# Supplementary material for: Modulating phosphatase DUSP22 with BML-260 ameliorates skeletal muscle wasting via Akt independent JNK-FOXO3a repression
Source: EMBO Mol Med. 2025 Apr 22;17(6):1259–88. doi: 10.1038/s44321-025-00234-2 (PMC12162873; doi:10.1038/s44321-025-00234-2)
Supplement: Supplementary file 1 — Appendix [file 44321_2025_234_MOESM1_ESM.pdf]

Appendix Figures and Tables: Contents

Page 3. Appendix Figure S1: Atrogin-1 expression in C2C12 murine myotubes treated with vehicle or dexamethasone to induce atrophy.

Page 4. Appendix Figure S2: Western blot analysis of DUSP22, MuRF-1, atrogin-1 and UBR2 levels C2C12 myoblasts transfected with a DUSP22 CRISPR activation plasmid or control plasmid.

Page 5. Appendix Figure S3: Western blot analysis of FOXO3a, FOXO3a phosphorylation, atrogin-1 and MuRF-1 in C2C12 myotubes treated with control or FOXO3a siRNA.

Page 6. Appendix Figure S4: Western blot analysis of atrogin-1 and DUSP22 in C2C12 myotubes treated with control or two distinct DUSP22 siRNAs.

Page 7. Appendix Figure S5: Phosphatase activity assay for DUSP22 and inhibition by BML 260.

Page 8. Appendix Figure S6: Fast myosin immunostaning and mean myotube diameter measurement of C2C12 myoblasts treated with Dex, SP600125 or BML-260.

Page 9. Appendix Figure S7: Type 1 myofiber staining, laminin staining, and cross sectional area in the tibialis anterior and gastrocnemius muscles of 5 months olds mice.

Page 10. Appendix Figure S8: Photographs and mass of the kidneys, liver, and heart after 6 weeks treatment with 5 mg/kg BML-260 in 15 months old mice.

Page 11. Appendix Figure S9: Expression changes for genes linked to PI3K-Akt signaling and gene set enrichment analysis (GSEA) combined with GO analysis of genes involved in the negative regulation of muscle hypertrophy and response to inactivity.

Page 12. Appendix Figure S10: qPCR analysis of atrogin-1, MuRF-1, and DUSP22 expression in dexamethasone-treated C2C12 myotubes treated with BML-260, DUSP22 siRNA, or DUSP22 siRNA plus BML-260.

Page 13. Appendix Table S1: Primary antibodies used in this study

Page 14. Appendix Table S2: Secondary antibodies used in this study

Page 15. Appendix Table S3: Primers used for qPCR – part 1

Page 16. Appendix Table S4: Primers used for qPCR – part 2

Page 17. Appendix Table S5: siRNAs used for gene knockdown

Appendix Figure S1

A

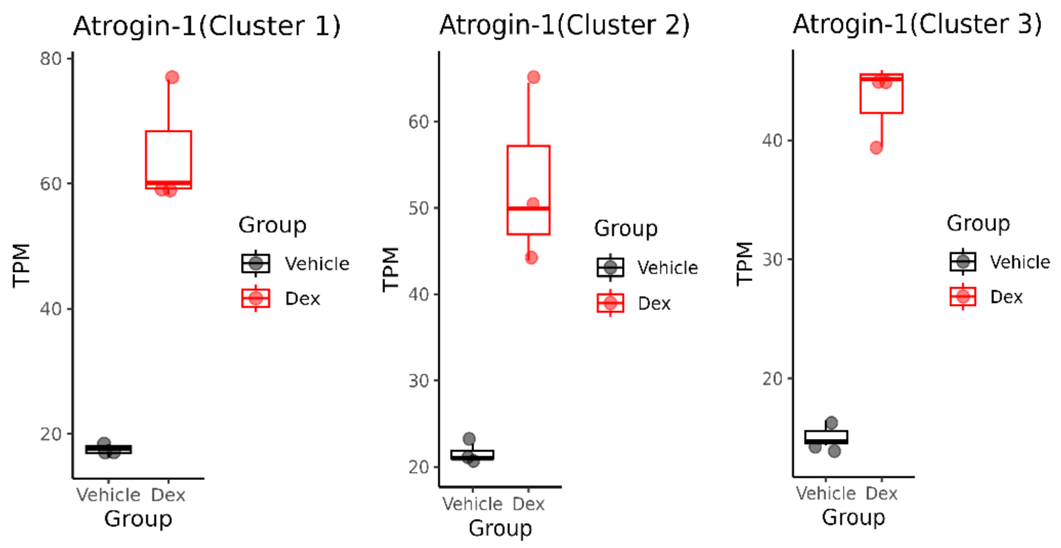

A) Atrogin-1 expression in C2C12 murine myotubes treated with vehicle or dexamethasone (Dex) to induce atrophy (n=3 each cluster, p=(Cluster 1=0.0012, Cluster 2= 0.007, Cluster 3= 0.0001)). Expression was measured using RNA Seq. TPM=transcript per million. Box plots represent the distribution of Atrogin-1 expression levels. The center line indicates the median (50th percentile, Q2), representing the middle value of the dataset. The box bounds correspond to the interquartile range (IQR), extending from the 25th percentile (Q1, lower bound) to the 75th percentile (Q3, upper bound). Whiskers extend to the smallest and largest values within 1.5 × IQR from Q1 and Q3, representing the minimum (lower whisker) and maximum (upper whisker) values within this range. Data points that fall beyond this range are considered outliers and are displayed as individual points outside the whiskers. n represents biological replicates

Appendix Figure S2

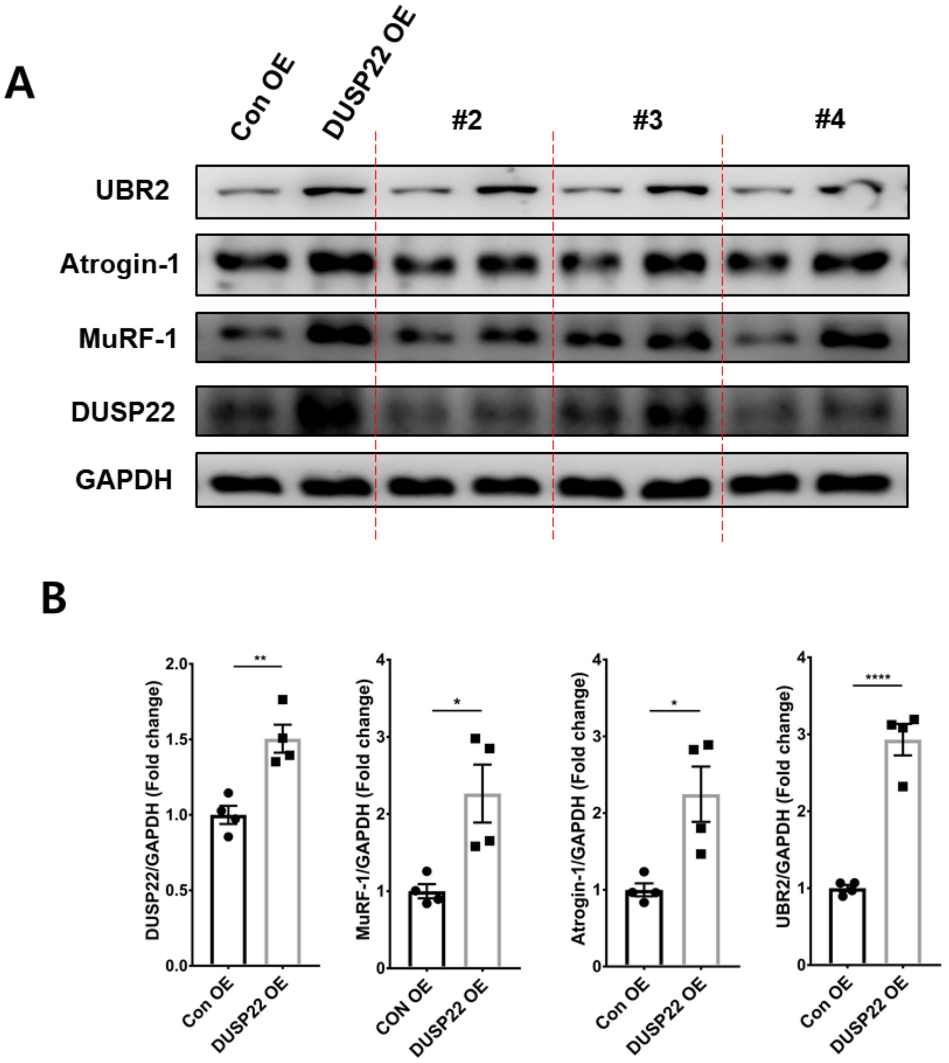

A) Western blot analysis of DUSP22(p=0.05), MuRF-1(p=0.017), atrogin-1(p=0.015) and UBR2(p=9E-05) levels C2C12 myoblasts transfected with a DUSP22 CRISPR activation plasmid (DUSP22endoOE) or control plasmid (CONendoOE) after 96 h culture in DM (n=4).B) Quantification of expression. GAPDH was used for the normalization of protein levels. *=p<0.05, **=p<0.01 ****=p<0.0001 indicate significantly increased or decreased. n represents biological replicates analyzed by Student's t test. Error bars represent the standard error of the mean (SEM).

Appendix Figure S3

A

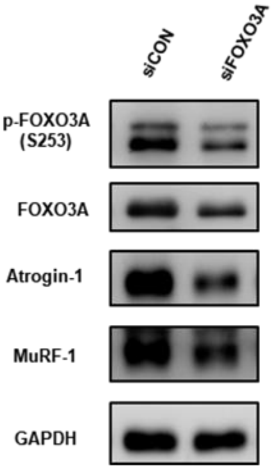

B

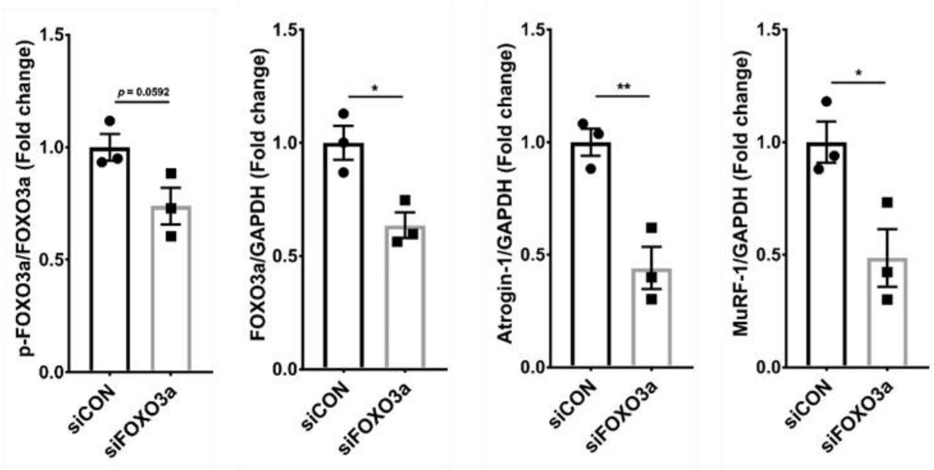

A) Western blot analysis of FOXO3a(p=0.0177), FOXO3a phosphorylation(p=0.0592), atrogin-1(p=0.0075) and MuRF-1(p=0.0309) in C2C12 myotubes treated with control or FOXO3a siRNA (n=3). B) Quantification of FOXO3a phosphorylation, FOXO3a, atrogin-1 and MuRF-1 protein levels compared to GAPDH. *=p<0.05, **=p<0.01 ****=p<0.0001 indicate significantly increased or decreased. n represents biological replicates analyzed by Student's t test. Error bars represent the standard error of the mean (SEM).

Appendix Figure S4

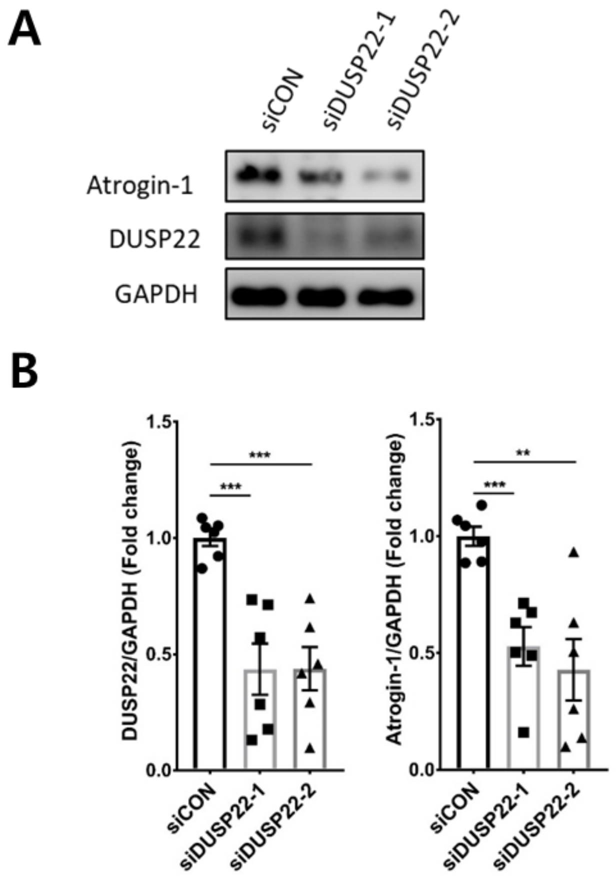

A) Western blot analysis of atrogin-1 and DUSP22 in C2C12 myotubes treated with control or two distinct DUSP22 siRNAs (termed siDUSP22-1 and siDUSP22-2) (n=6). B) Quantification of DUSP22(p=(siDUSP22-1=0.0006,siDUSP22-2=0.0002) and atrogin-1(p=(siDUSP22-1=0.0004,siDUSP22-2=0.002) protein levels compared to GAPDH. **=*p*<0.01 ****=*p*<0.0001 indicate significantly increased or decreased. n represents biological replicates analyzed by Student's t test. Error bars represent the standard error of the mean (SEM).

Appendix Figure S5

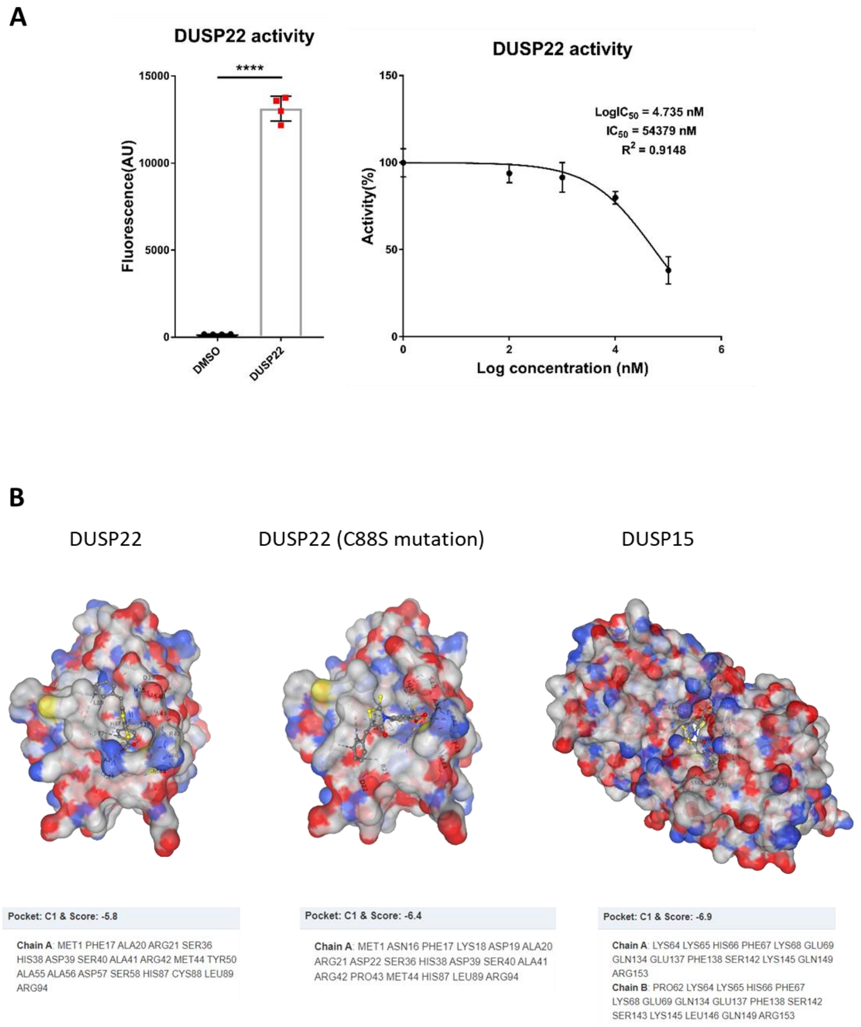

A) Phosphatase activity assay for DUSP22 and inhibition by BML-260. The assay is based on the 6,8-difluoro-4-methylumbelliferyl phosphate (DiFMUP) reagent and GST-tagged-DUSP22. (n=4) B) CB-Dock2 molecular docking analysis of BML-260 binding to the active site of DUSP22, DUSP22 with an active site mutation (at C88S), and DUSP15, which is a DUSP member with the highest homology with DUSP22 (as assessed by sequence alignment). DUSP2 (C88S mutation) and DUSP15 DUSP2 (C88S mutation) and DUSP15 showed altered BML-260 binding. BML-260 bound DUSP15 at the interface of the A and B chain, distinct from the predicted active site at position 85. n represents technical replicates analyzed by Student's t test. Data are presented as mean ± standard deviation (SD).

Appendix Figure S6

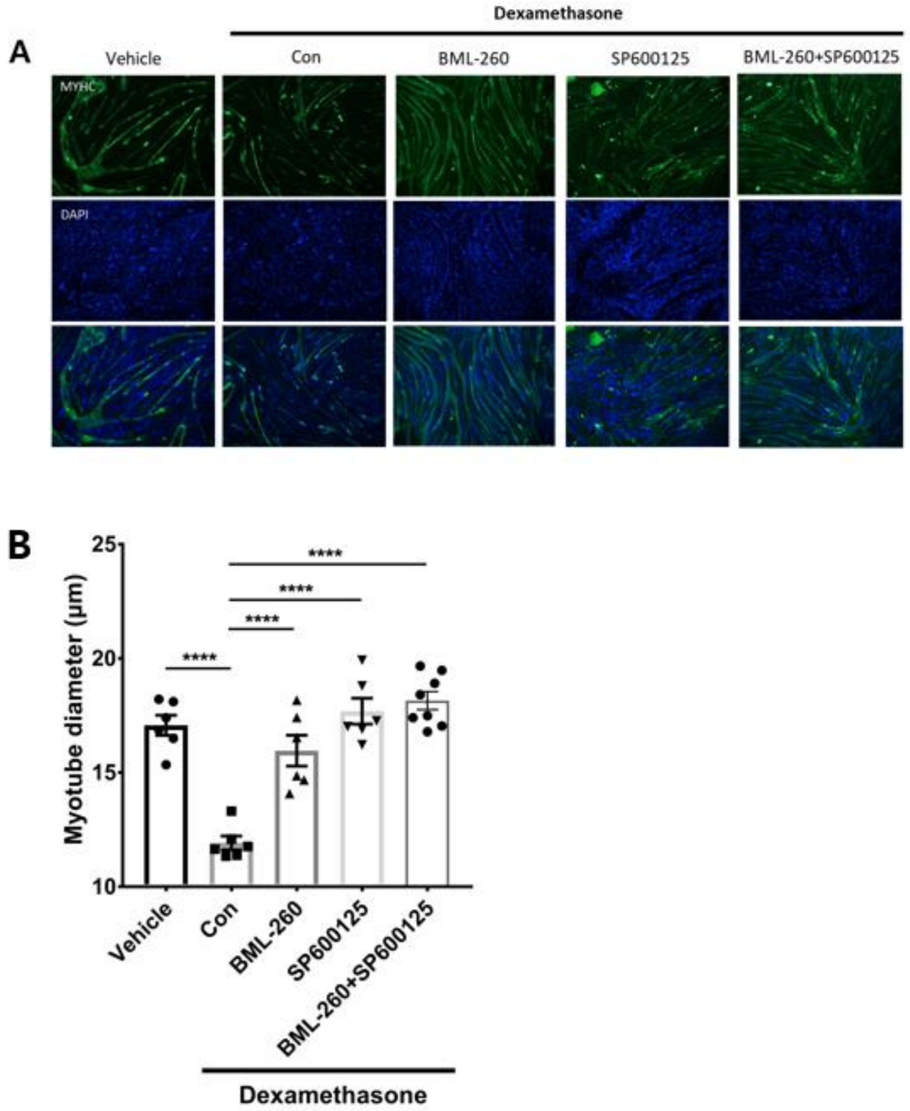

A) Fast myosin (MYH2) immunostaning of C2C12 myoblasts cultured as follows: (1) DM for 120 h (vehicle alone)(p=2.17E-06); (2) DM for 96 h and DM plus 10 μM Dex for 24 h; (3) DM for 96 h and DM plus 10 μM Dex and 12.5 μM BML-260 for 24 h(p=0.0002); (4) DM for 96 h and DM plus 10 μM Dex and 20 μM SP600125 for 24 h(p=4.11E-06); (5) DM for 96 h and DM plus 10 μM Dex and 12.5 μM BML-260 plus 20 μM SP600125 for 24 h(p=5.88E-08).(n=6) B) Mean myotube diameter. ****=*p*<0.0001 indicate significantly increased or decreased. n represents biological replicates analyzed by Student's t test. Error bars represent the standard error of the mean (SEM).

Appendix Figure S7

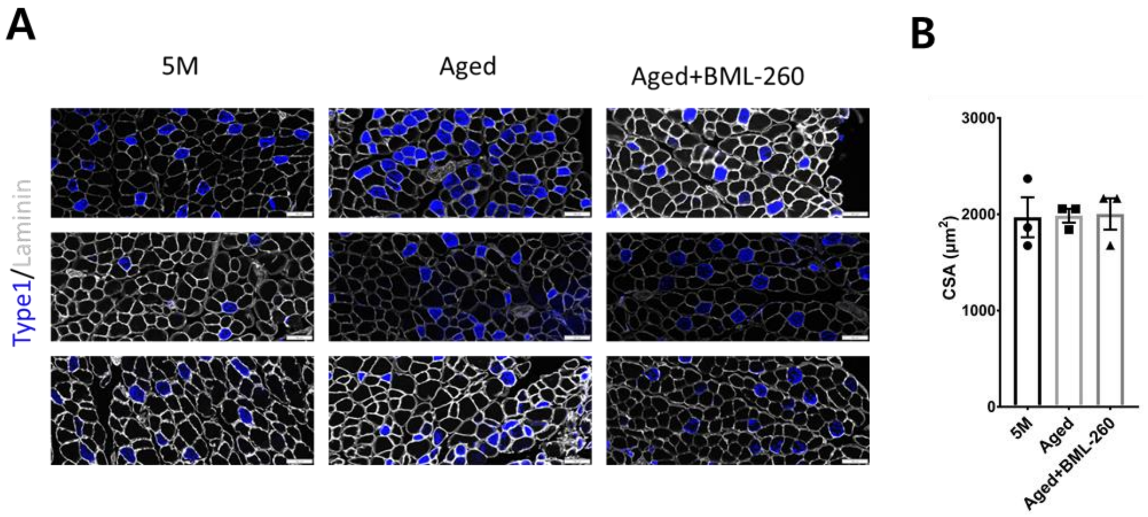

A) Type 1 myofiber and laminin staining in the TA and gastrocnemius muscles (5M=5 months-old)(p=(5M=0.9444,Aged+BML-260=0.9236)). Scale bar=100 μm (n=3). B) CSA of the type 1 myofibers. n represents biological replicates analyzed by Student's t test. Error bars represent the standard error of the mean (SEM).

Appendix Figure S8

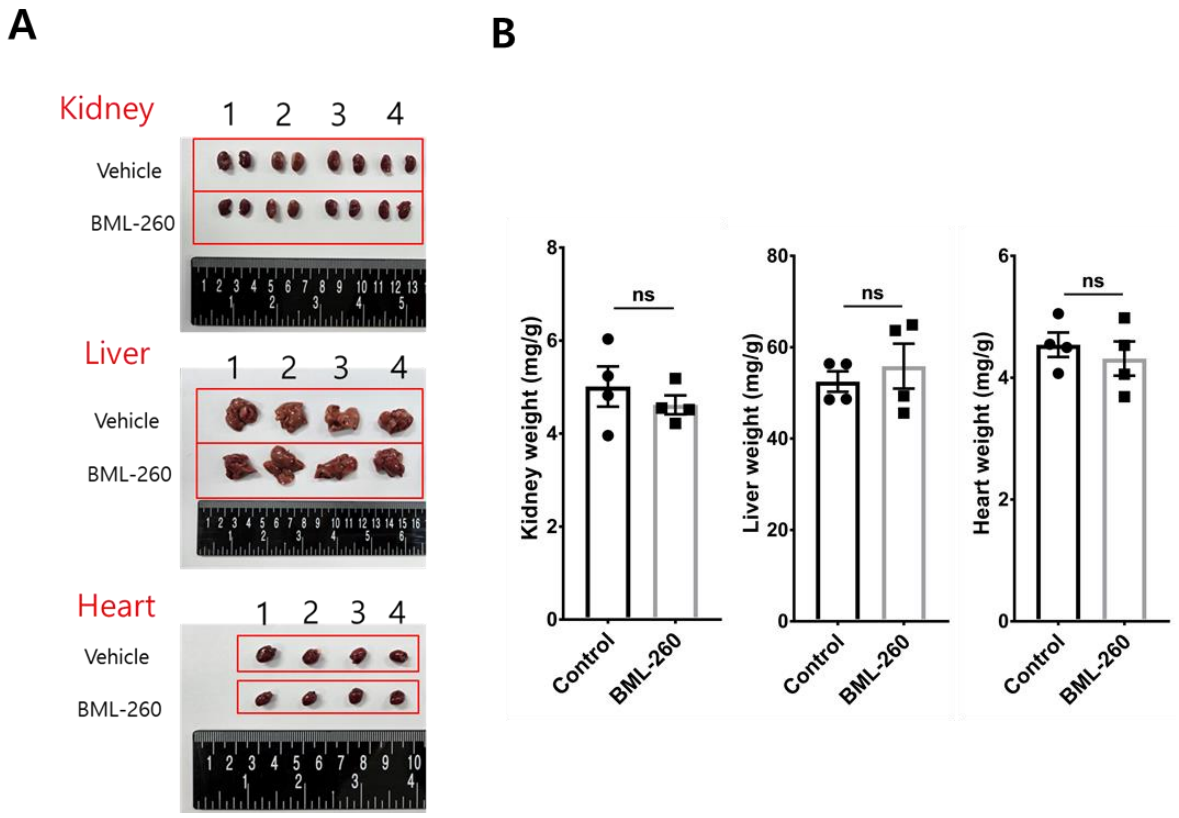

A) Photographs of the kidneys, liver, and heart after 6 weeks treatment with 5 mg/kg in 15 month-old mice. (n=4) B) Kidney(p=0.4403), liver(0.5545), and heart(0.5349) mass. ns=not statistically significant. n represents biological replicates analyzed by Student's t test. Error bars represent the standard error of the mean (SEM).

Appendix Figure S9

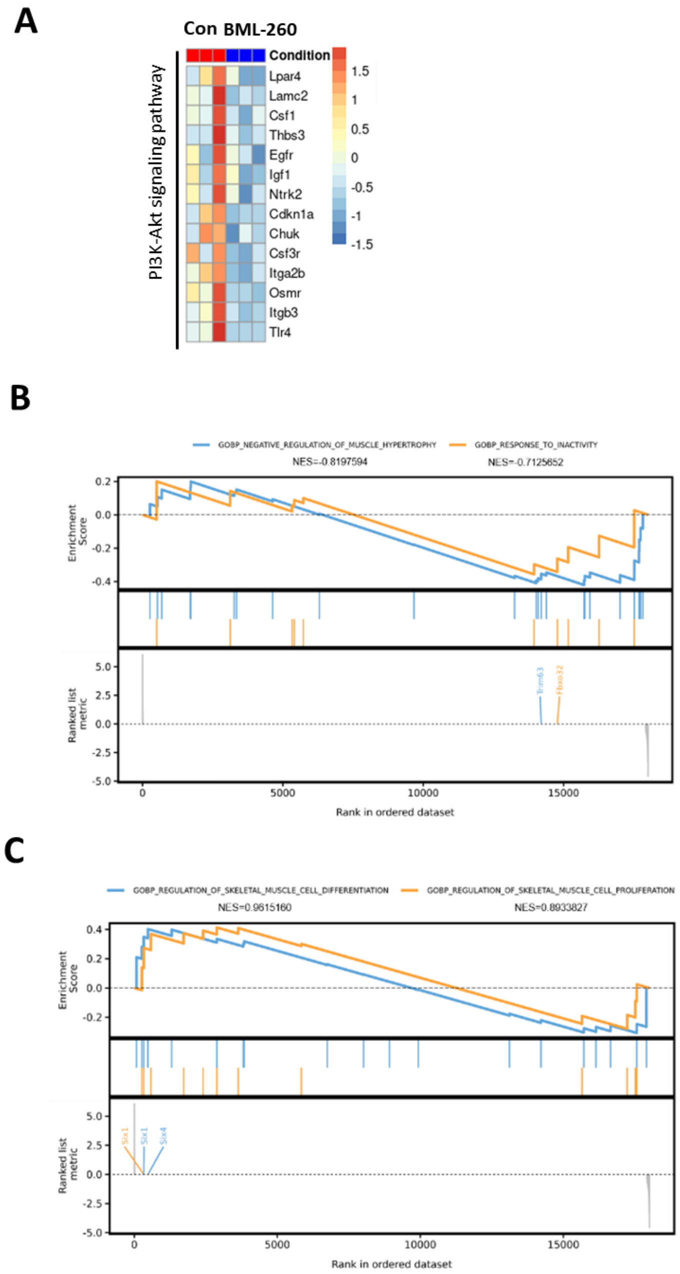

A) Expression changes for genes linked to PI3K-Akt signaling. B) Gene set enrichment analysis (GSEA) combined with GO analysis of genes involved in the negative regulation of muscle hypertrophy and response to inactivity. The rankings for atrogin-1 (Fbox32) and MuRF-1 (Trim63) are indicated. C) (GSEA) combined with GO analysis of genes involved in the regulation of skeletal muscle differentiation and proliferation. The rankings for Six1 and Six4 are indicated.

Appendix Figure S10

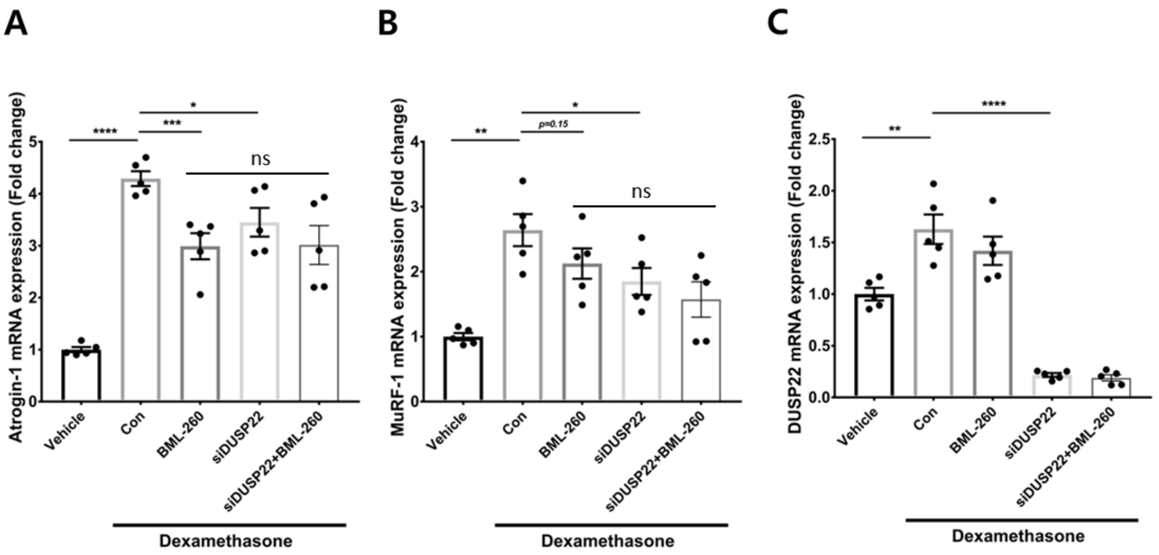

qPCR analysis of A) atrogin-1(p=(Vehicle=2.12E-08, BML-260=0.0001, siDUSP22=0.2716, siDUSP22+Dex=0.0127)), B) MuRF-1(p=(Vehicle=0.0001, BML-260=0.1671, si-DUSP22=0.0396, siDUSP22+Dex=0.0196)), and C) DUSP22 (p=(Vehicle=0.0036, BML-260=0.3213, siDUSP22=9.77E-06, siDUSP22+Dex=9.24E-06)) expression in dexamethasone-treated C2C12 myotubes treated with BML-260 alone, DUSP22 siRNA, or DUSP22 siRNA plus BML-260 (n=5). *=p<0.05, **=p<0.01, ***=p<0.001 and ****=p<0.0001 indicate significantly increased or decreased. ns=not significant. n represents biological replicates. Error bars represent the standard error of the mean (SEM).

Appendix Table S1

Primary antibodies used in this study

Primary antibody	Clone	Company	Catalog No.	Dilution
**α-Tubulin**	Polyclonal	INVITROGEN	PA5-29444	1:10000
**Atrogin-1/MAFbx**	Monoclonal	Abcam	Ab168372	1:1000
**MuRF-1/Trim63**	Monoclonal	SANTA CRUZ	SC-398608	1:500
**FoxO3a**	Monoclonal	Cell Signaling	#12829	1:1000
**UBR2**	Polyclonal	Abcam	Ab217069	1:1000
**Phospho-FoxO3a**	Polyclonal	Cell Signaling	#9466	1:1000
**AKT**	Monoclonal	Cell Signaling	#2983	1:1000
**Phospho-AKT**	Monoclonal	Cell Signaling	#2971	1:1000
**JNK**	Monoclonal	Cell Signaling	SC-7345	1:1000
**Phospho-JNK**	Monoclonal	Cell Signaling	#4668	1:1000
**c-Jun**	Monoclonal	Cell Signaling	#9165	1:1000
**Phospho-c-Jun**	Monoclonal	Cell Signaling	#2361	1:1000
**STAT3**	Monoclonal	SANTA CRUZ	SC-8019	1:1000
**Phospho-STAT3**	Monoclonal	SANTA CRUZ	SC-8059	1:1000
**Myosin heavy chain2**	Monoclonal	SANTA CRUZ	SC-53095	1:1000
**DUSP22**	Polyclonal	Abcam	Ab70124	1:1000
**Myosin Heavy Chain 2A**	Monoclonal	DSHB	SC-71	1:500
**Myosin Heavy Chain 1**	Monoclonal	DSHB	BA-D5	1:50
**Myosin Heavy Chain 2B**	Monoclonal	DSHB	BF-F3	1:100
**Laminin**	Polyclonal	sigma	L9393	1:50
**Fast-type skeletal muscle**	monoclonal	SANTA CRUZ	sc-32732	1:100

Appendix Table S2

Secondary antibodies used in this study

Primary antibody	Conjugated use	Company	Catalog No.	Dilution
Goat anti-Mouse IgG (H+L)	HRP	Abcam	ab6789	1:10000
Goat anti-Rabbit IgG HRP	HRP	Cell signaling	#7074S	1:10000
Alexa Fluor™ 488 Goat anti-mouse IgG(H+L)	Alexa Fluor 488	INVITROGEN	A11001	ICC:1:2000
Alexa Fluor™ 555 Goat anti-mouse IgG(H+L)	Alexa Fluor 555	INVITROGEN	A21422	IHC 1:500
Alexa Fluor™ 488 Goat anti-mouse IgG(H+L)	Alexa Fluor 488	INVITROGEN	A28175	IHC 1:500
Alexa Fluor™ 350 Goat anti-mouse IgG(H+L)	Alexa Fluor 350	INVITROGEN	A11045	IHC 1:500

Appendix Table S3

Primers used for qPCR – part 1

Primer name	Primer sequence	Size (bp)	Accession Number
**GAPDH**	F : CTCCACTCACGGCAAATTCA<br>R : GCCTCACCCCATTTGATGTT	120	NM_001289726
**Atrogin-1**	F : CAGAGAGCTGCTCCGTCTCA<br>R : ACGTATCCCCCGCAGTTTC	178	NM_026346
**MuRF-1**	F : CCGAGTGCAGACGATCATCTC<br>R : TGGAGGATCAGAGCCTCGAT	198	NM_001039048
**Myh2**	F :GATCACCACGAACCCATATGATT<br>R : TTCATGTTCCCATAATGCATCAC	183	NM_011039
**Pax7**	F : CACAGAGGCAGAGCTGATTGC<br>R : CCAATTGAGGAGAGTGACAGGTT	157	NM_011039
**Myf5**	F : AGCTGGGCAGAATACGTGCTT<br>R : AGAACAGGCAGAGGAGAATCCA	112	NM_008656
**Myogenin**	F : AGCGCAGGCTCAAGAAAGTG<br>R : CCGCCTCTGTAGCGGAGAT	181	NM_031189
**MyoD1**	F : TGTCCTTTCGAAGCCGTTCT<br>R : TGCAGCCAGAGTGCAAGTG	169	NM_010866
**DUSP22**	F : GATGCCTTGCACACTGTTCGT<br>R : ACTGGTGCACTTCATGTTTCTCA	100	NM_001037955.4
**DUSP22 human**	F : GGTTTCTGTACCTCGCTTGGAT<br>R : AGGCGTTCACAGGAAGCA A	100	NM_001286555.3
**Atrogin-1 human**	F :GGAACTACTCCAGACCCTCTACACA<br>R : CTCCATCCGATACACCCACAT	103	NM_148177
**MuRF-1 human**	F : TTGACTTTGGGACAGATGAGGAA<br>R : CCAGCTCCTTACTGGTGTCCTT	102	NM_032588
**GAPDH human**	F : CTGCACCACCAACTGCTTAGC<br>R : TCTTCTGGGTGGCAGTGATG	107	NM_002046
**PGC-1α**	F : CAG GGT GCA TGG CAG TTG T<br>R : CAG AGG CCA TGC TAG TGA AAG A	100	NM_008904
**UCP-3**	F : GAT GTG GTG AAG GTC CGA TTT C<br>R : CCC TGG CGA TGG TTC TGT AG	100	NM_009464
**Acly**	F : ATG CCA AGA CCA TCC TCT CAC T<br>R : GCG GCC ACA TTG GTG AAG	100	NM_134037
**LC-3B**	F :CGT CCT GGA CAA GAC CAA GT<br>R :ATT GCT GTC CCG AAT GTC TC	100	NM_026160

Appendix Table S4

Primers used for qPCR – part 2

Primer name	Primer sequence	Size (bp)	Accession Number
**Cathepsin L**	F : CAG GGT CCG TGA AGC TGT CT<br>R : CTG CAG TGC TGC CAG CTT T	100	NM_001257971
**Psmd11**	F : TTT CTT ACG CCA AGC ATT GGA<br>R : CTC CCG AAG CAG CTG AGA AC	100	NM_178616
**UBR2**	F : TAT TCT CCT CCT TAC CTT G<br>R : CGA AAC CGC TCT TGG CAT A	100	NM_001177374
**MYH7**	F : TGT TTT TGT GCC CGA TGA<br>R : CAG TCA CCG TCT TGC CAT T	100	NM_080728
**MYH1**	F : CGG GAG AAC CAG TCT ATT TTG ATC<br>R : CTC CCC AGT GAC TGC AAT TGT	100	NM_030679
**MYH4**	F : GTC GGC AAT GAG TAT GTC A<br>R : TGA CCA TCC ATA GGA ACA TC	100	NM_010855
**FoxO3a**	F : TGGAGTCCATCATCCGTAGTGA<br>R : CTGGTACCCAGCTTTGAGATGAG	147	NM_019740
**P62**	F : CCC AGT GTC TTG GCA TTC TT<br>R : AGG GAA AGC AGA GGA AGC TC	100	NM_001290769
**TGIF**	F : TTT CCT CAT CAG CAG CCT CT<br>R : CTT TGC CAT CCT TTC TCA GC	100	NM_001164074
**ATF4**	F : TCC TGA ACA GCG AAG TGT TG<br>R : ACC CAT GAG GTT TCA AGT GC	100	NM_001287180
**Bnip3**	F : TTC CAC TAG CAC CTT CTG ATG A<br>R : GAA CAC CGC ATT TAC AGA ACA A	100	NM_009760
**Gadd45a**	F : GAA AGT CGC TAC ATG GAT CAG T<br>R : AAA CTT CAG TGC AAT TTG GTT C	100	NM_007836
**SMART**	F : TCA ATA ACC TCA AGG CGT TC<br>R : GTT TTG CAC ACA AGC TCC A	PMID: 33927785	
**MUSA1**	F : TCG TGG AAT GGT AAT CTT GC<br>R : CCT CCC GTT TCT CTA TCA CG	PMID: 33927785	

Appendix Table S5

siRNAs used for gene knockdown

Target	Sequence	Accession Number
**DUSP22-1(mouse)**	Sense(5>3) : CCUGACAAGACAUUUCAAAtt<br>Antisense(5>3) : UUUGAAAUGUCUUGUCAGGtt	NM_001037955
**DUSP22-1(human)**	Sense(5>3) : CCUGACAAGACAUUUCAAAtt<br>Antisense(5>3) : UUUGAAAUGUCUUGUCAGGtt	NM_001286555
**DUSP22-2(mouse)**	Sense(5>3) : GGGAGUUAAAUACCUGUGUtt<br>Antisense(5>3) : ACACAGGUAUUUAACUCCCtc	NM_001037955
**FOXO3a**	Sense(5>3) : GAGCUCUUGGUGGAUCAUCtt<br>Antisense(5>3) : GAUGAUCCACCAAGAGCUCtt	NM_019740.3
