# Supplementary figures and images for: Modulating phosphatase DUSP22 with BML-260 ameliorates skeletal muscle wasting via Akt independent JNK-FOXO3a repression (part 1 of 3)
Source: EMBO Mol Med. 2025 Apr 22;17(6):1259–88. doi: 10.1038/s44321-025-00234-2 (PMC12162873; doi:10.1038/s44321-025-00234-2)

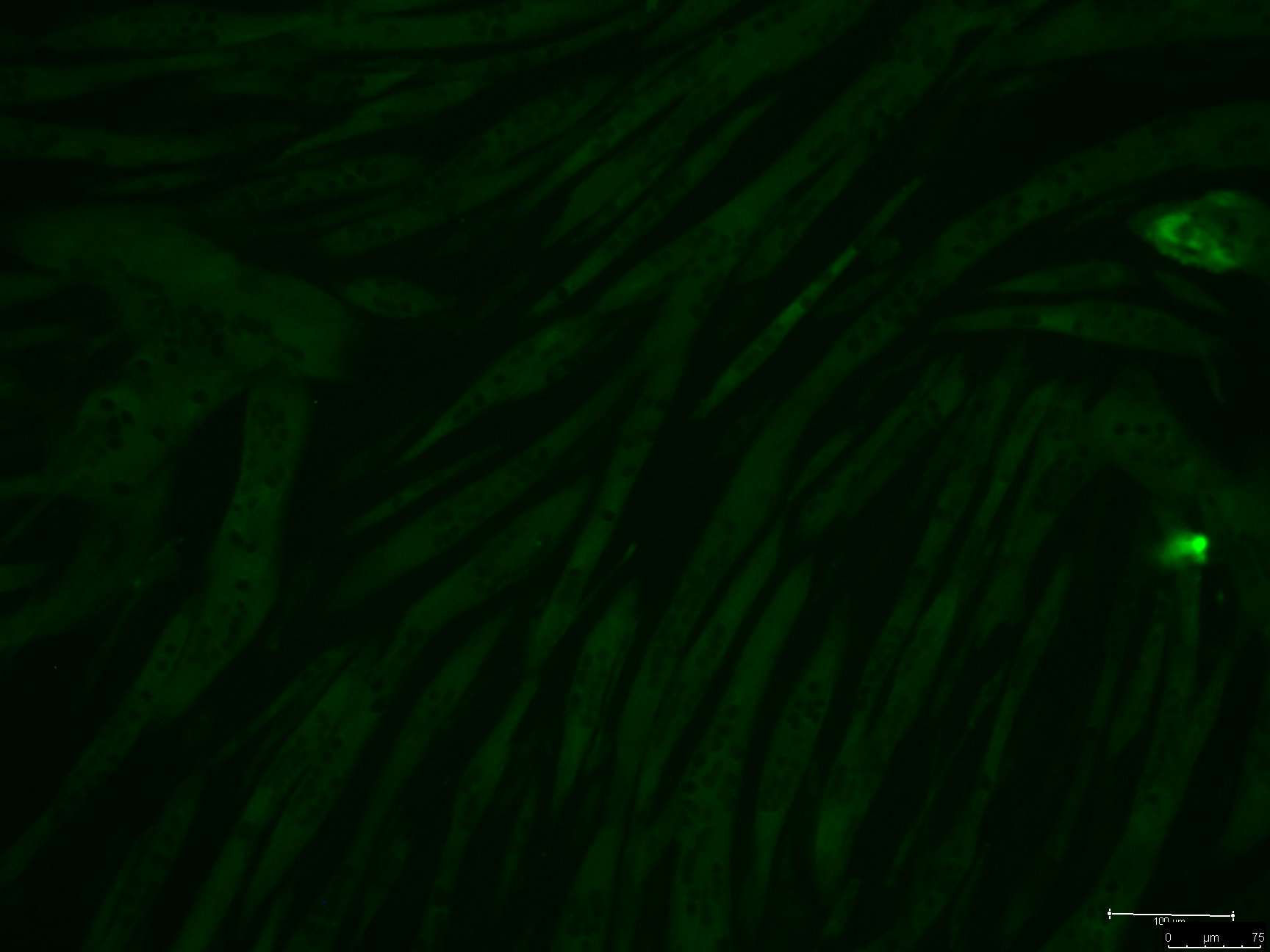

Supplement: Supplementary file 3 — Source data Fig. 1 [file 44321_2025_234_MOESM3_ESM.zip › Figure 1E/Con OE/dmso_Image001_ch00.tif]

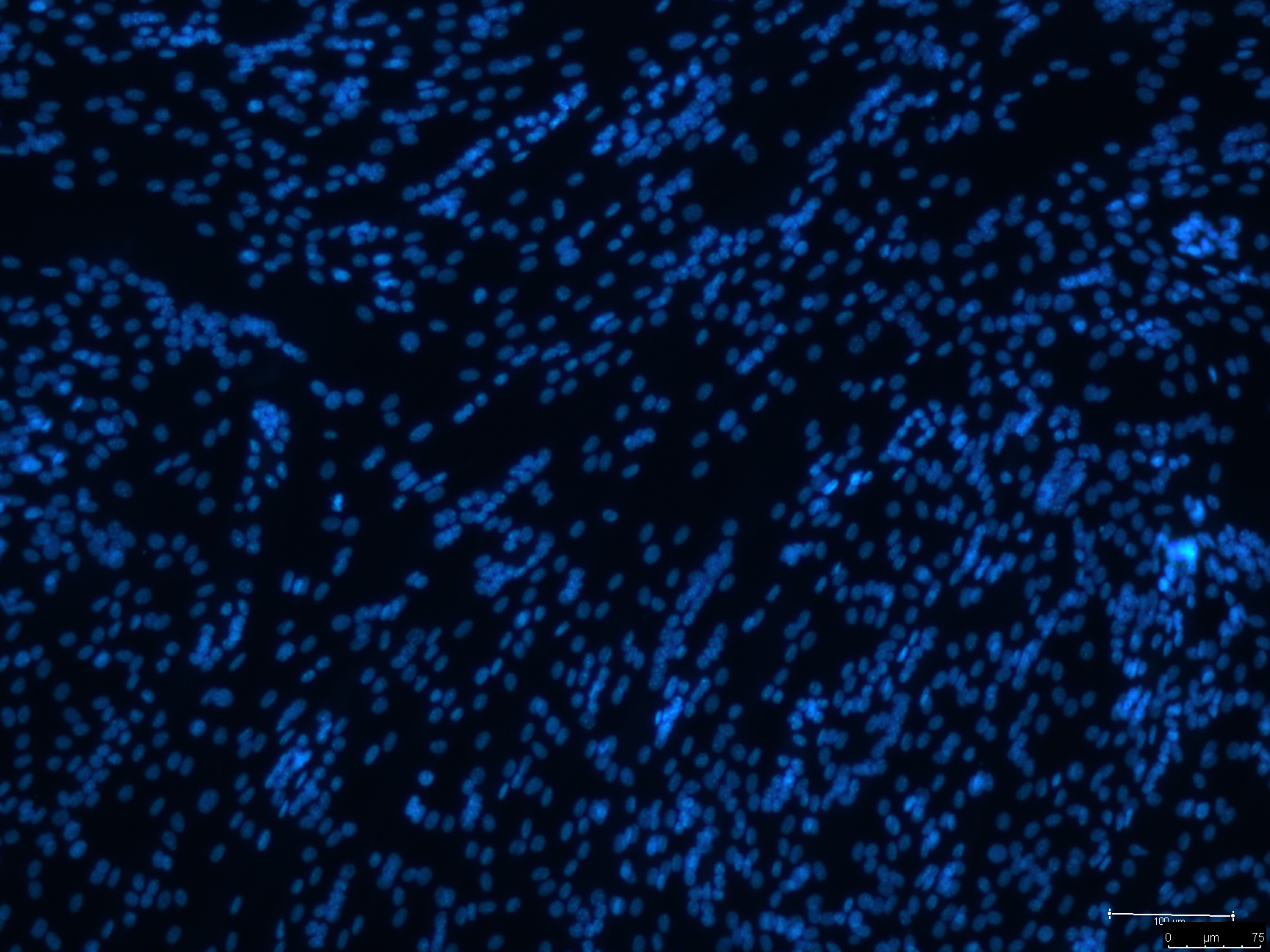

Supplement: Supplementary file 3 — Source data Fig. 1 [file 44321_2025_234_MOESM3_ESM.zip › Figure 1E/Con OE/dmso_Image002_ch00.tif]

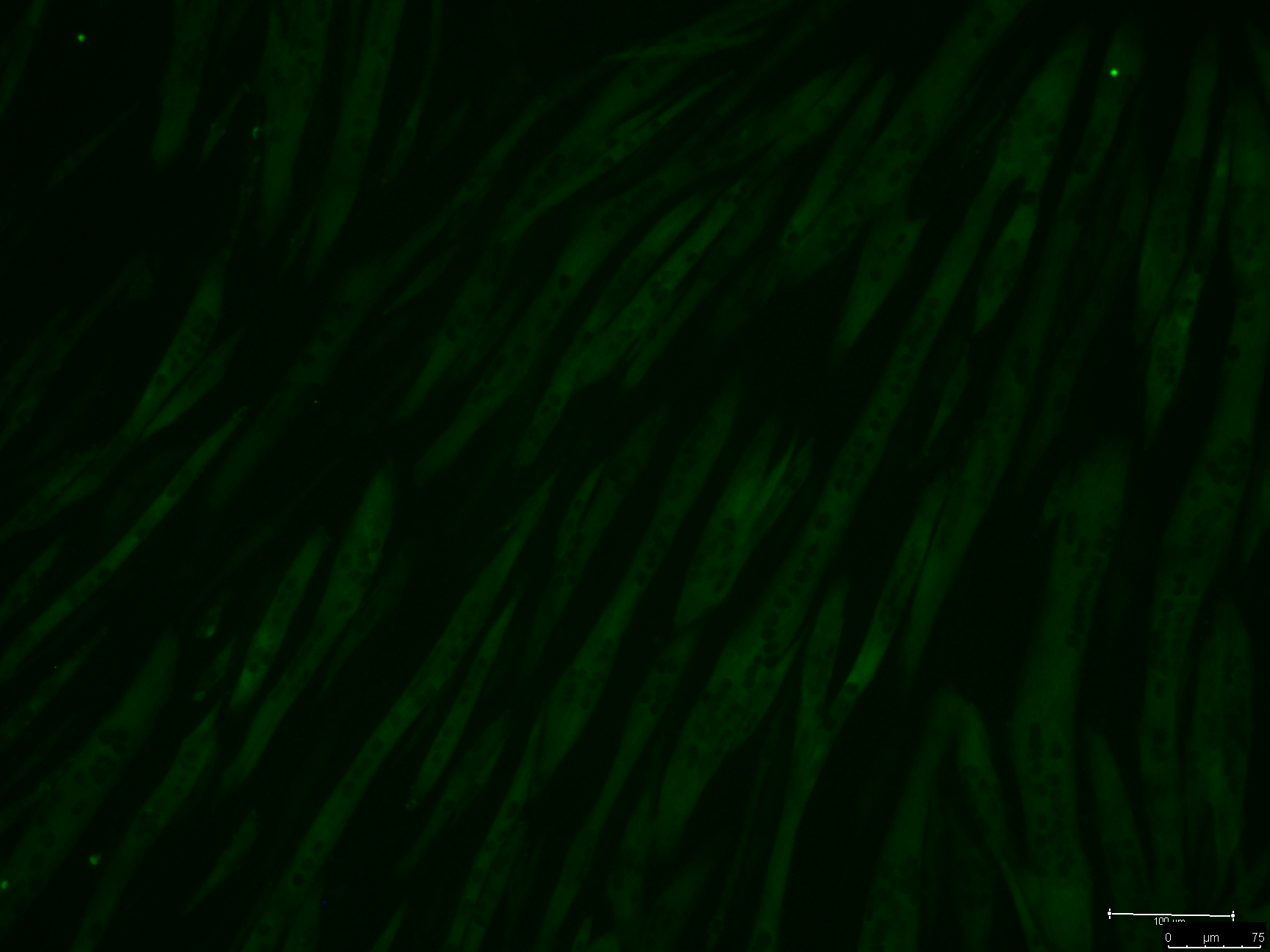

Supplement: Supplementary file 3 — Source data Fig. 1 [file 44321_2025_234_MOESM3_ESM.zip › Figure 1E/Con OE/dmso_Image003_ch00.tif]

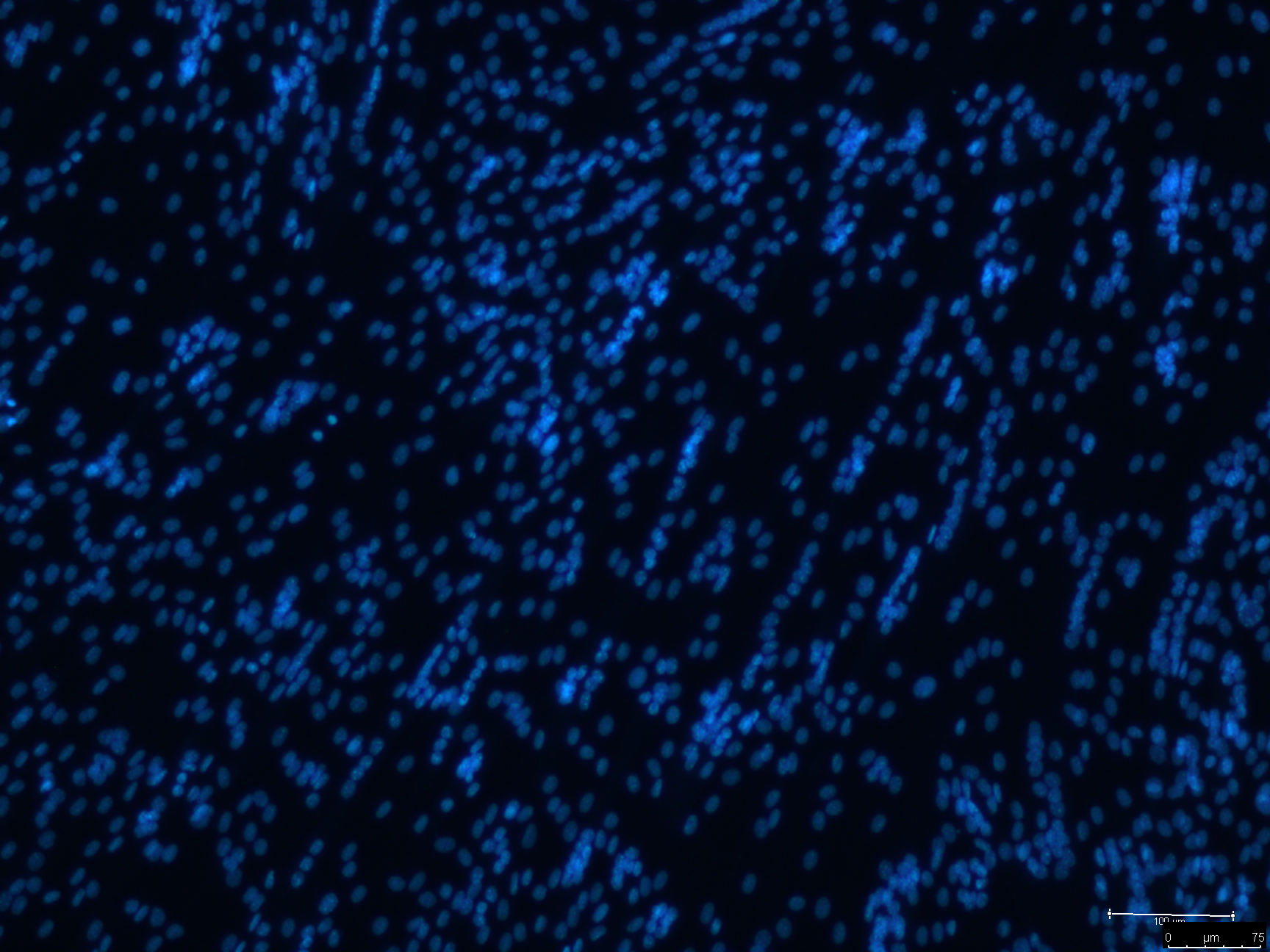

Supplement: Supplementary file 3 — Source data Fig. 1 [file 44321_2025_234_MOESM3_ESM.zip › Figure 1E/Con OE/dmso_Image004_ch00.tif]

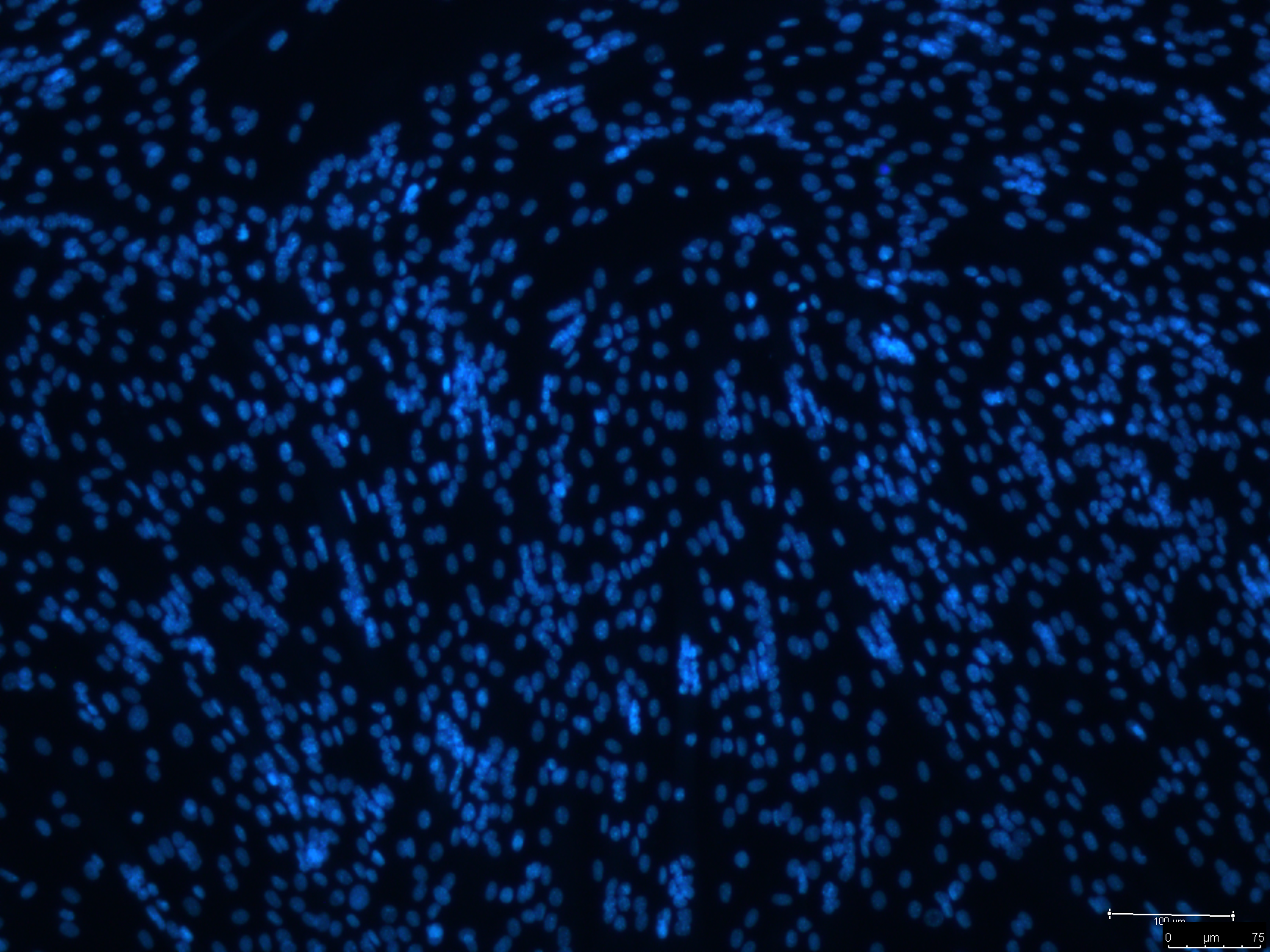

Supplement: Supplementary file 3 — Source data Fig. 1 [file 44321_2025_234_MOESM3_ESM.zip › Figure 1E/Con OE/dmso_Image005_ch00.tif]

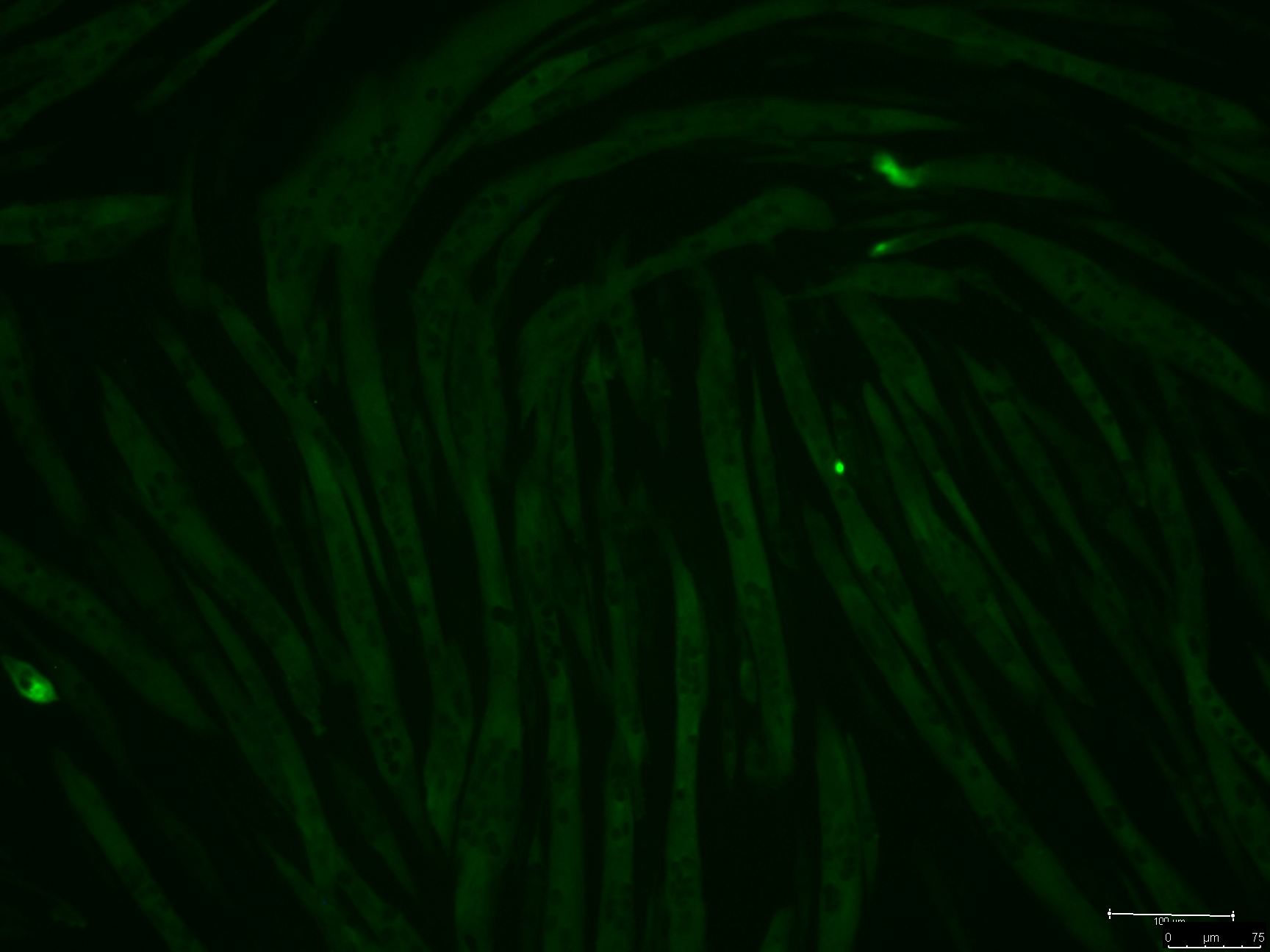

Supplement: Supplementary file 3 — Source data Fig. 1 [file 44321_2025_234_MOESM3_ESM.zip › Figure 1E/Con OE/dmso_Image006_ch00.tif]

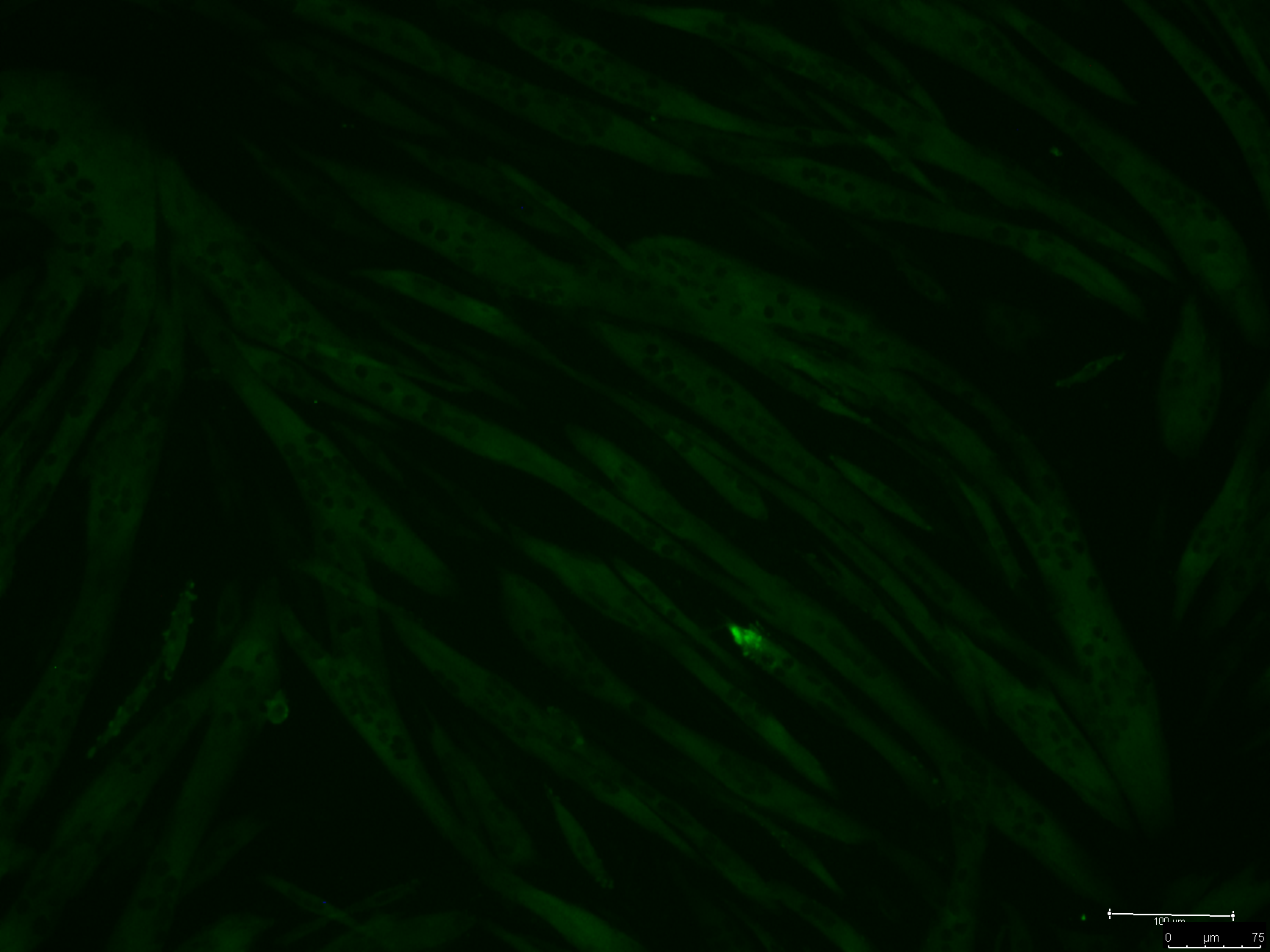

Supplement: Supplementary file 3 — Source data Fig. 1 [file 44321_2025_234_MOESM3_ESM.zip › Figure 1E/Con OE/dmso_Image007_ch00.tif]

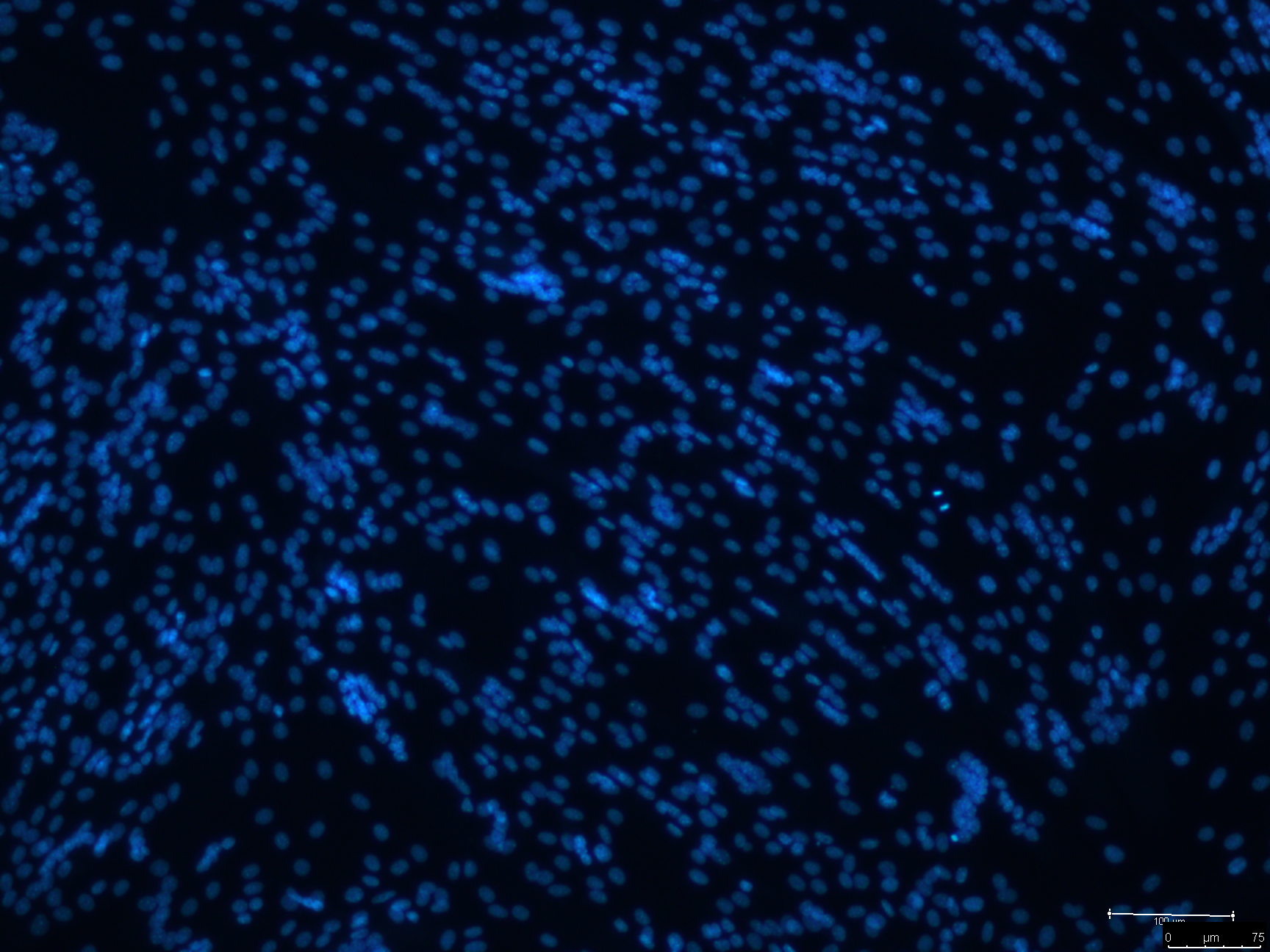

Supplement: Supplementary file 3 — Source data Fig. 1 [file 44321_2025_234_MOESM3_ESM.zip › Figure 1E/Con OE/dmso_Image008_ch00.tif]

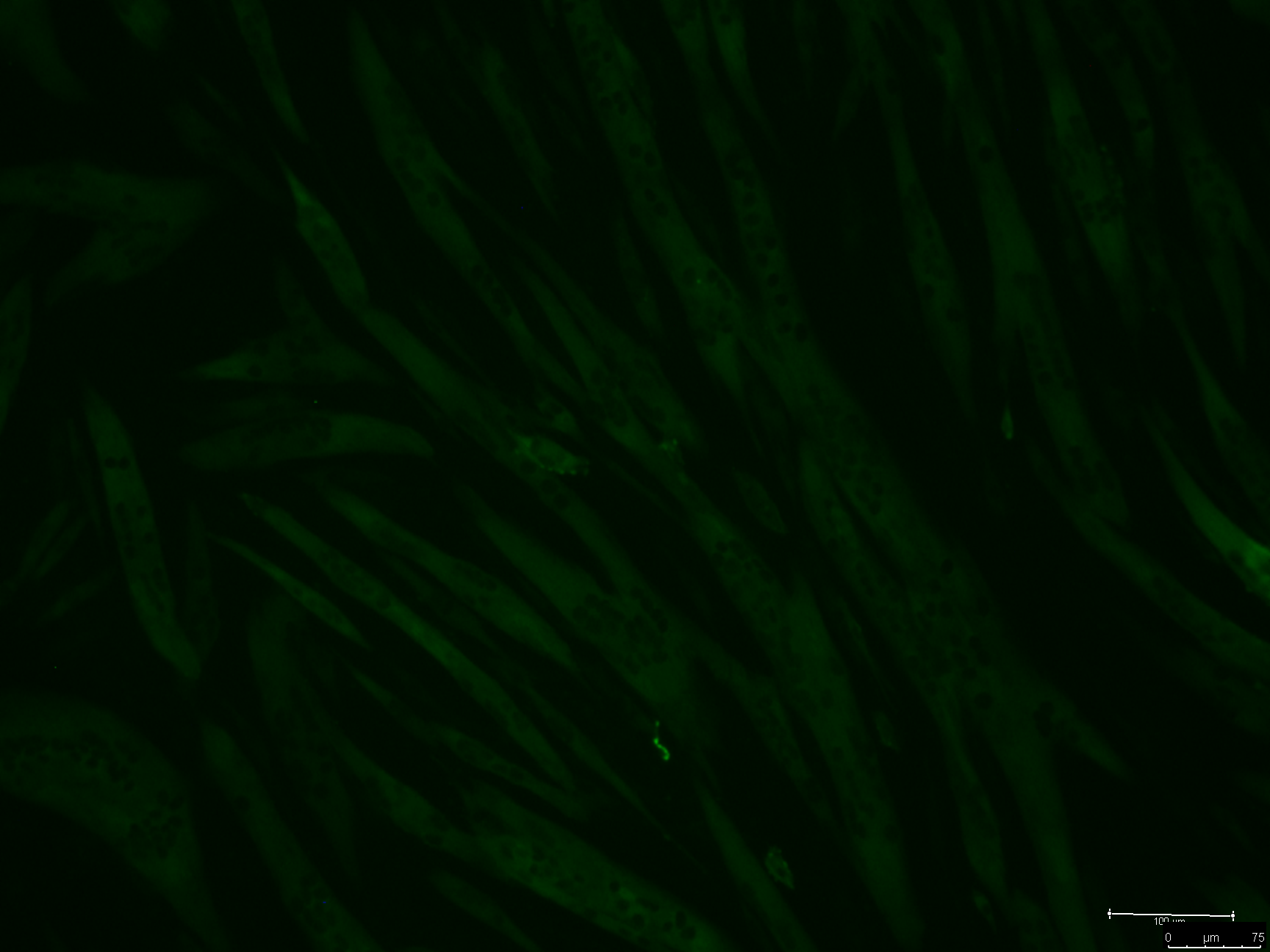

Supplement: Supplementary file 3 — Source data Fig. 1 [file 44321_2025_234_MOESM3_ESM.zip › Figure 1E/Con OE/dmso_Image009_ch00.tif]

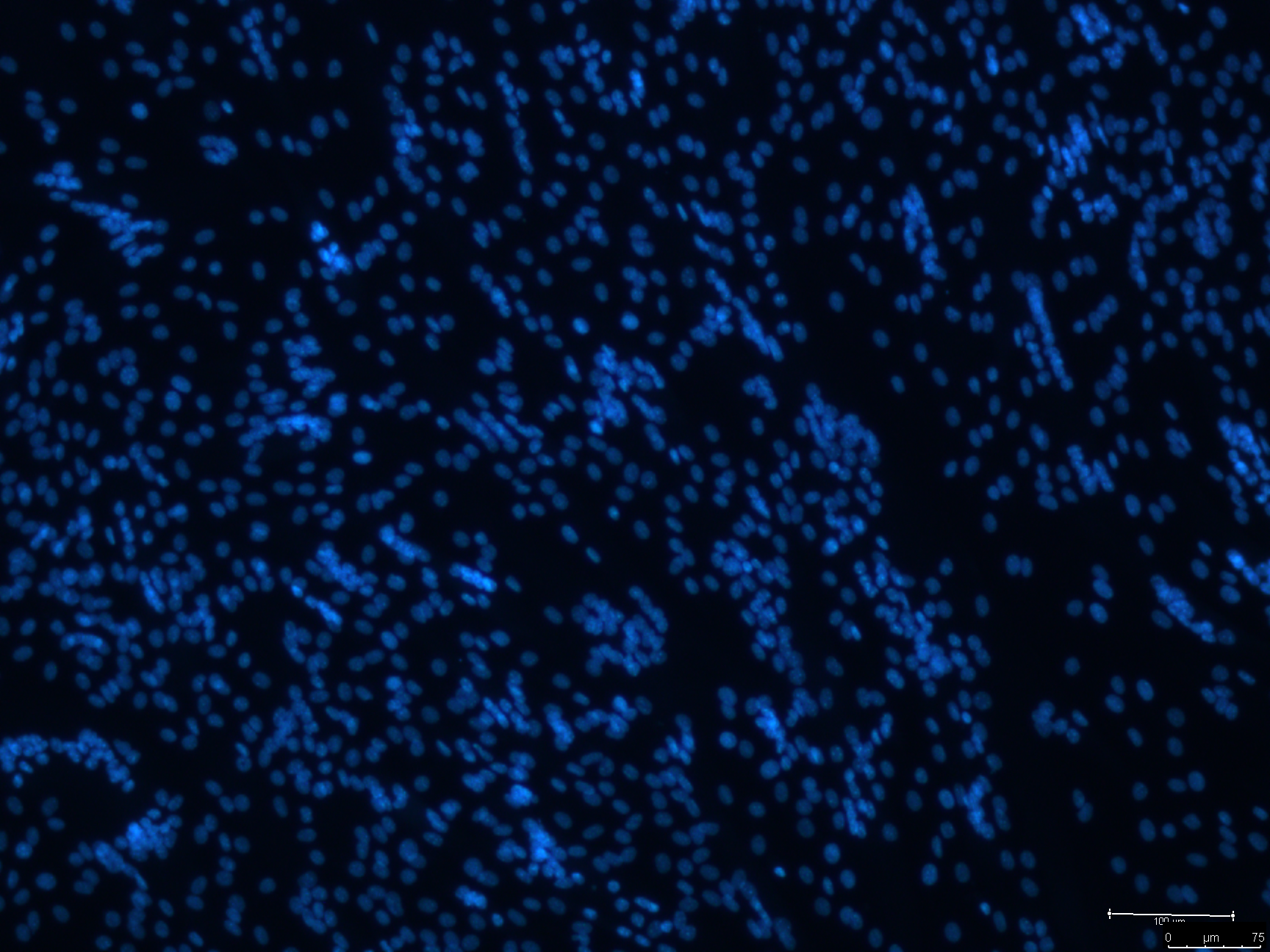

Supplement: Supplementary file 3 — Source data Fig. 1 [file 44321_2025_234_MOESM3_ESM.zip › Figure 1E/Con OE/dmso_Image010_ch00.tif]

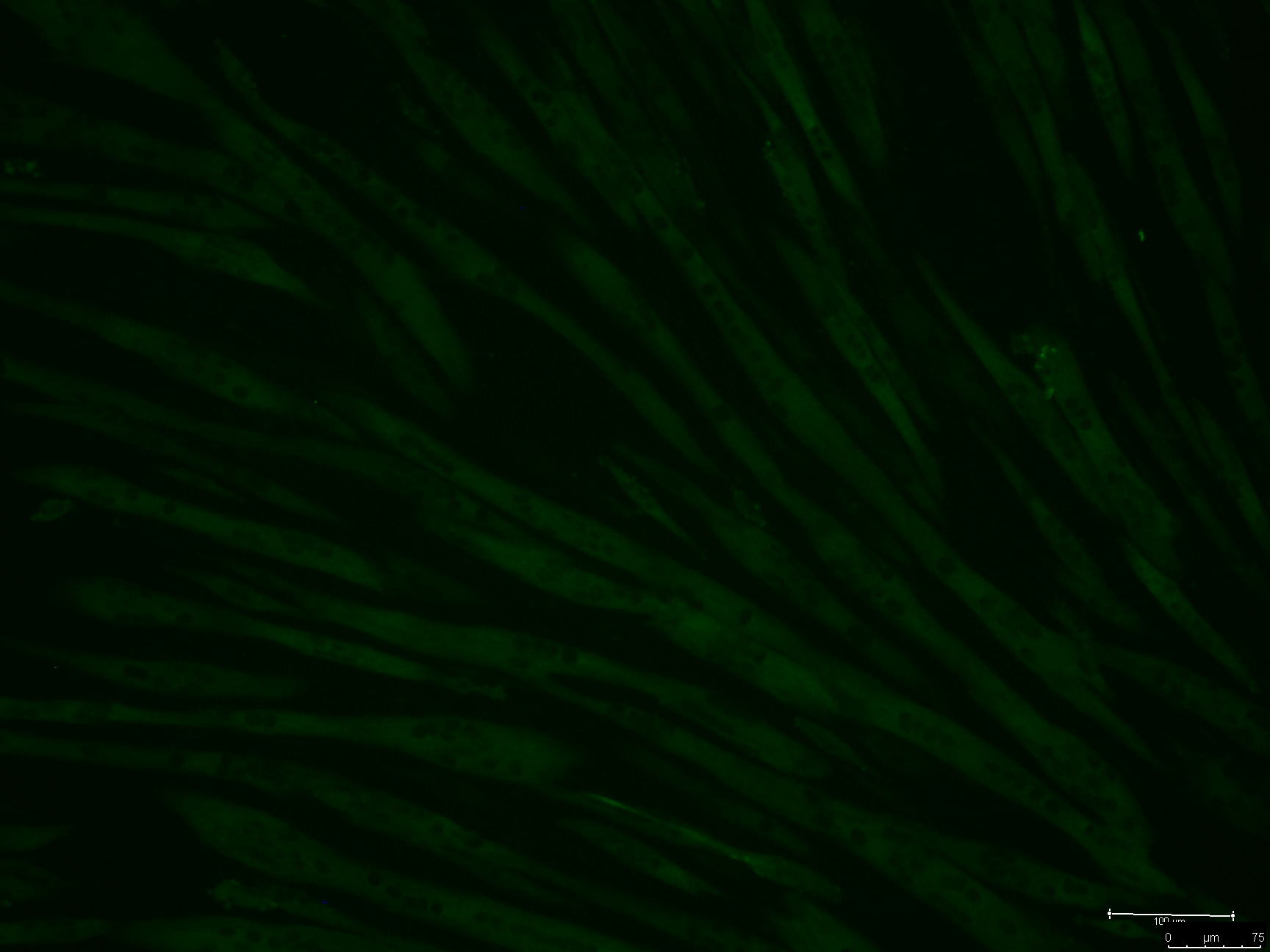

Supplement: Supplementary file 3 — Source data Fig. 1 [file 44321_2025_234_MOESM3_ESM.zip › Figure 1E/Con OE/dmso_Image011_ch00.tif]

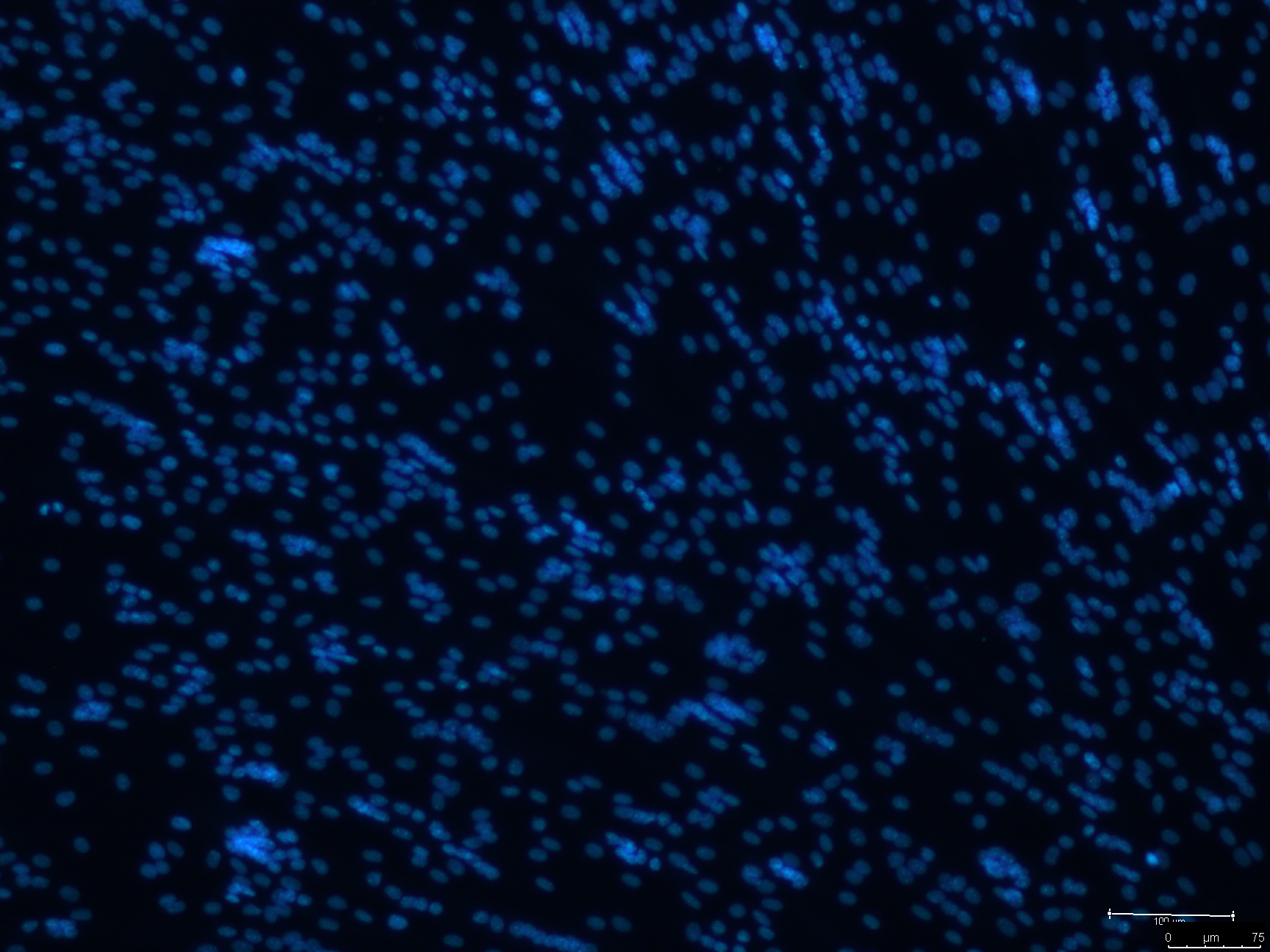

Supplement: Supplementary file 3 — Source data Fig. 1 [file 44321_2025_234_MOESM3_ESM.zip › Figure 1E/Con OE/dmso_Image012_ch00.tif]

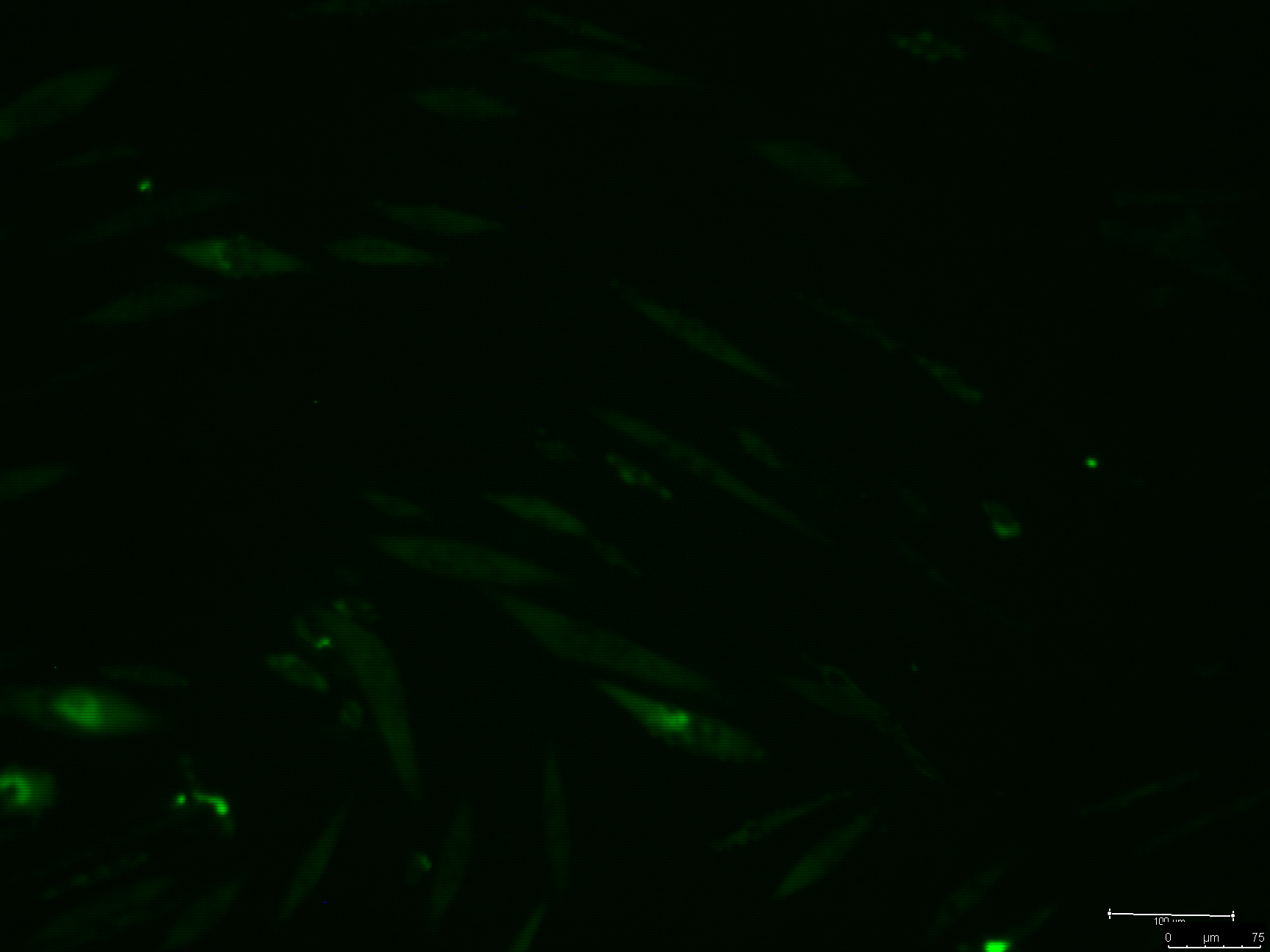

Supplement: Supplementary file 3 — Source data Fig. 1 [file 44321_2025_234_MOESM3_ESM.zip › Figure 1E/DUSP22 OE/dmso_Image013_ch00.tif]

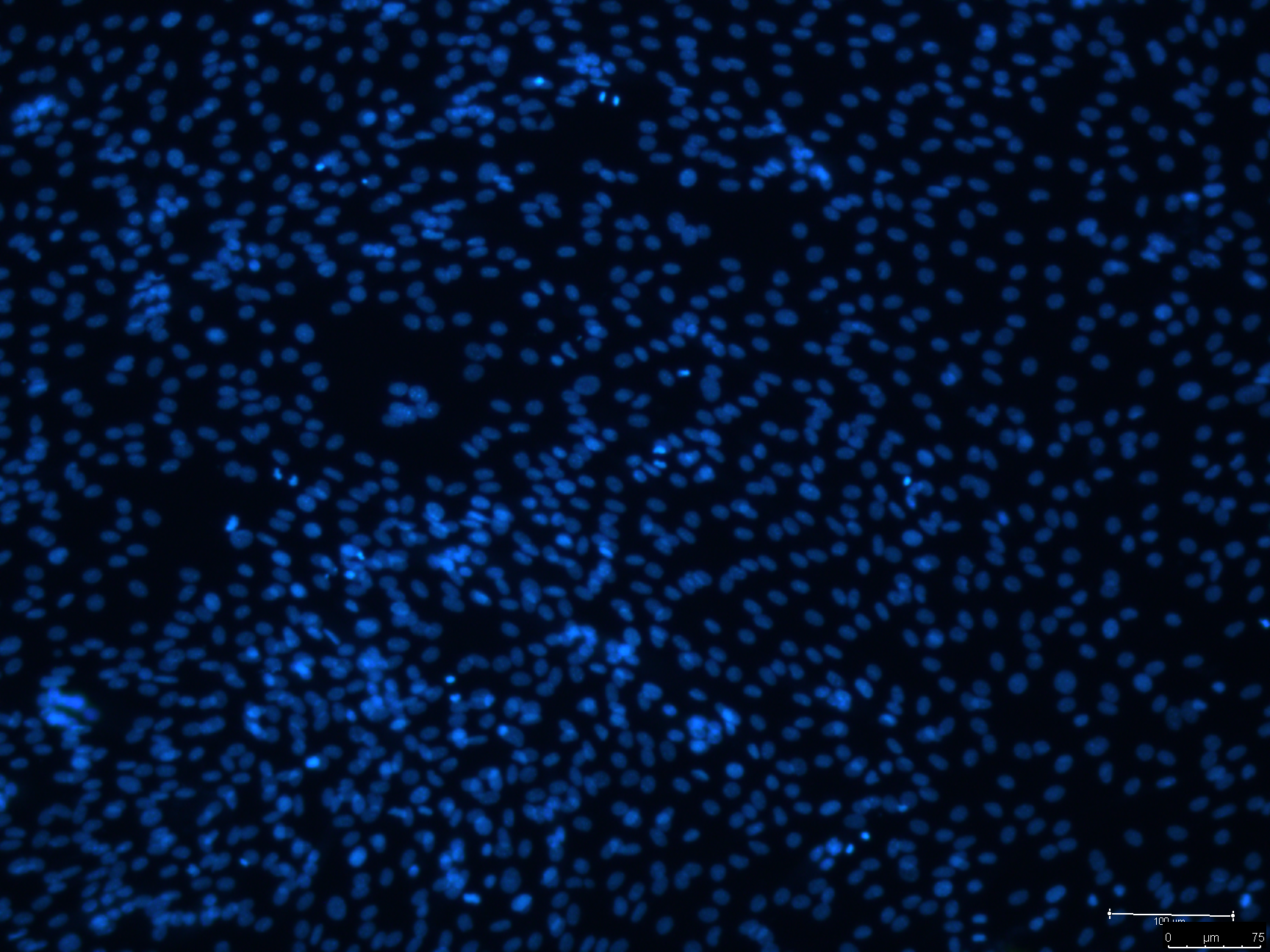

Supplement: Supplementary file 3 — Source data Fig. 1 [file 44321_2025_234_MOESM3_ESM.zip › Figure 1E/DUSP22 OE/dmso_Image014_ch00.tif]

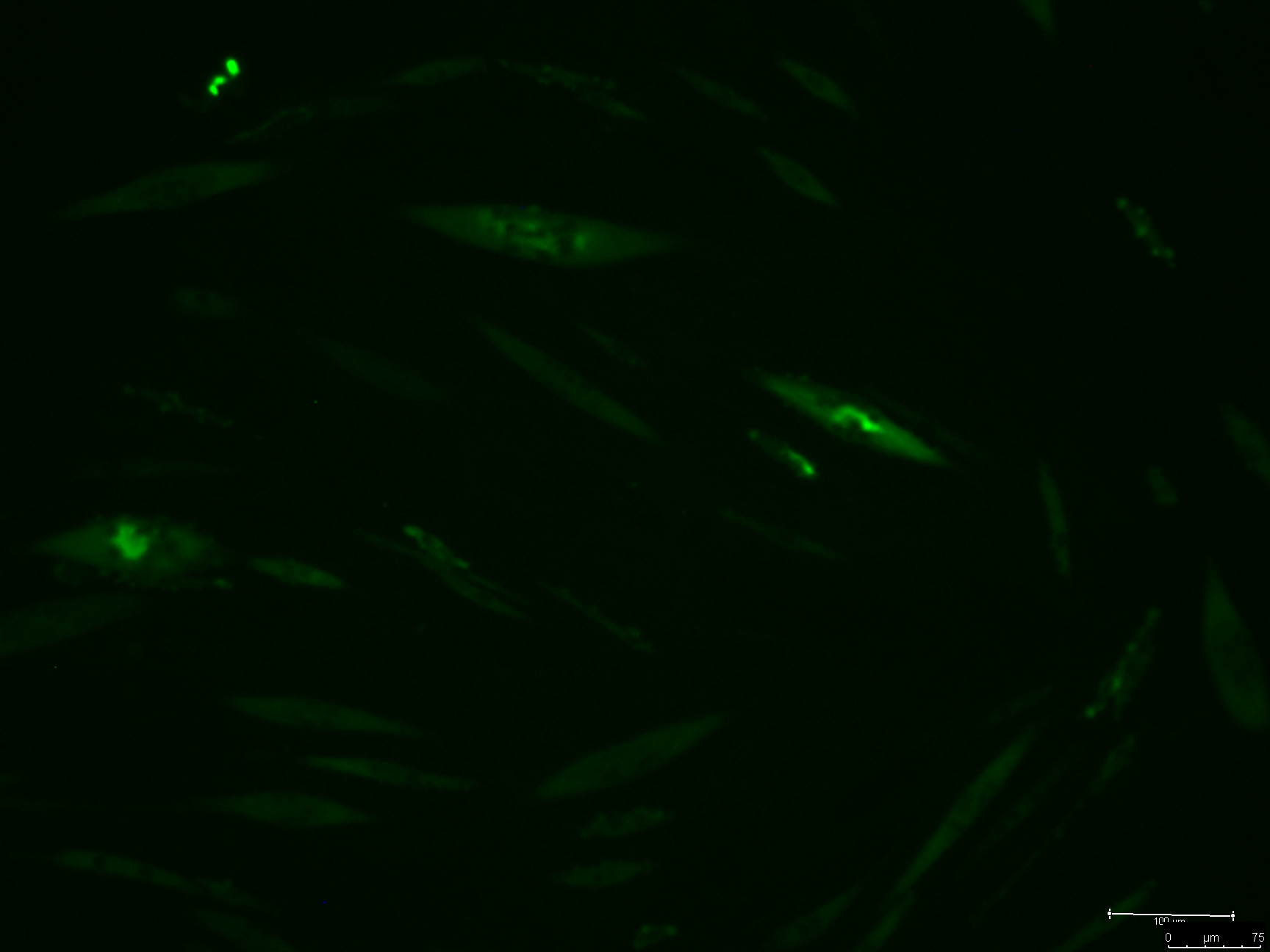

Supplement: Supplementary file 3 — Source data Fig. 1 [file 44321_2025_234_MOESM3_ESM.zip › Figure 1E/DUSP22 OE/dmso_Image015_ch00.tif]

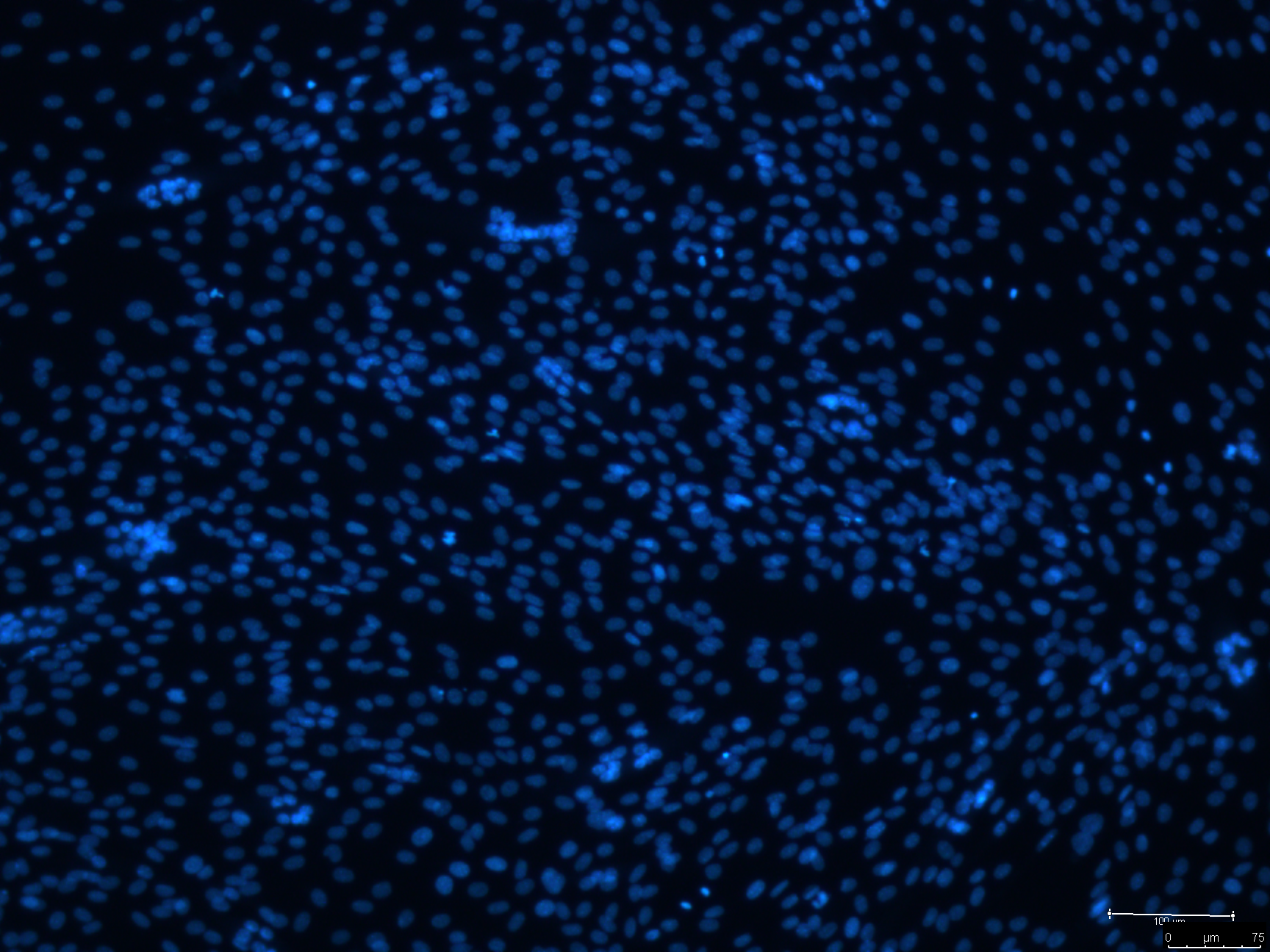

Supplement: Supplementary file 3 — Source data Fig. 1 [file 44321_2025_234_MOESM3_ESM.zip › Figure 1E/DUSP22 OE/dmso_Image016_ch00.tif]

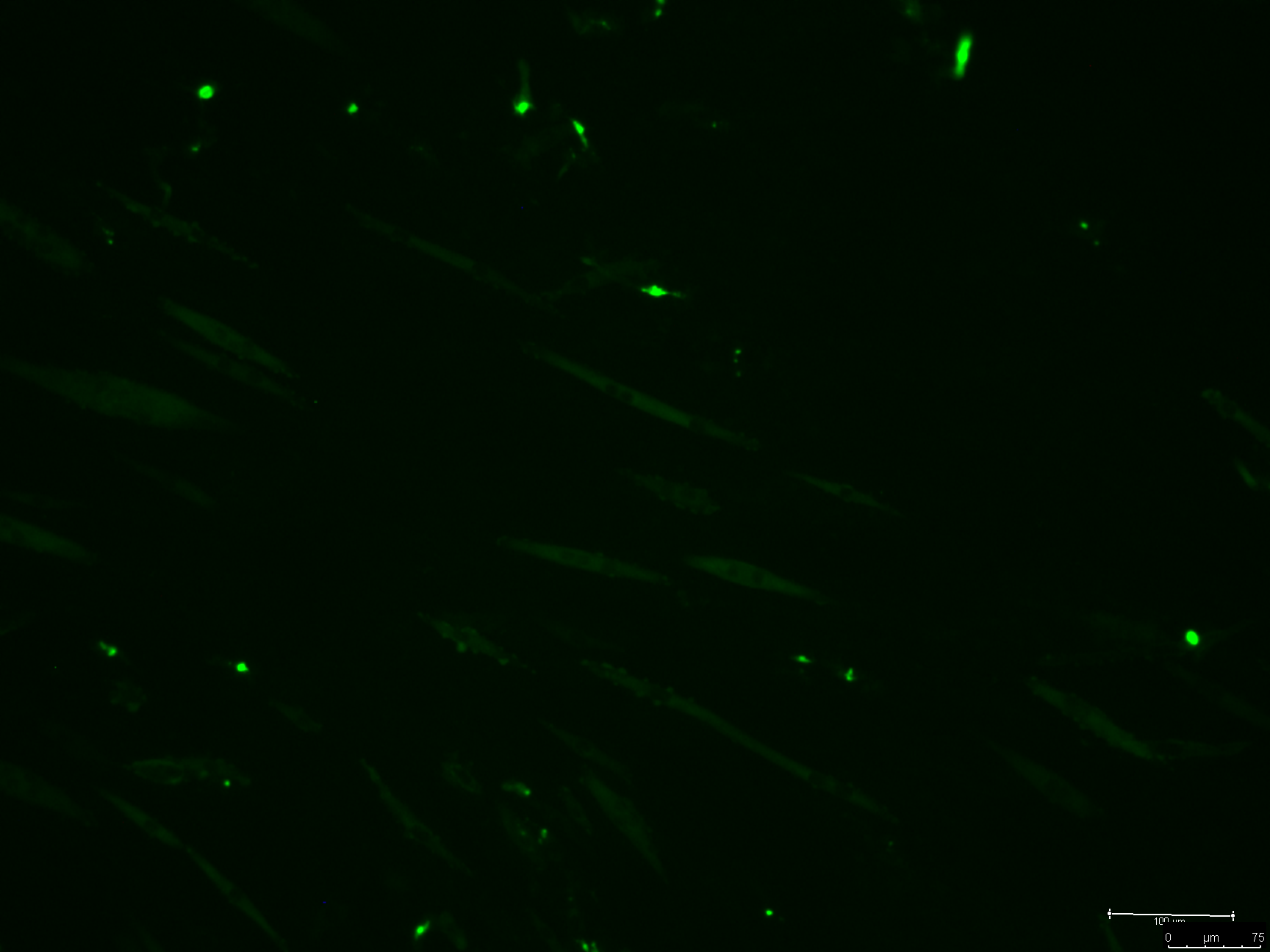

Supplement: Supplementary file 3 — Source data Fig. 1 [file 44321_2025_234_MOESM3_ESM.zip › Figure 1E/DUSP22 OE/dmso_Image017_ch00.tif]

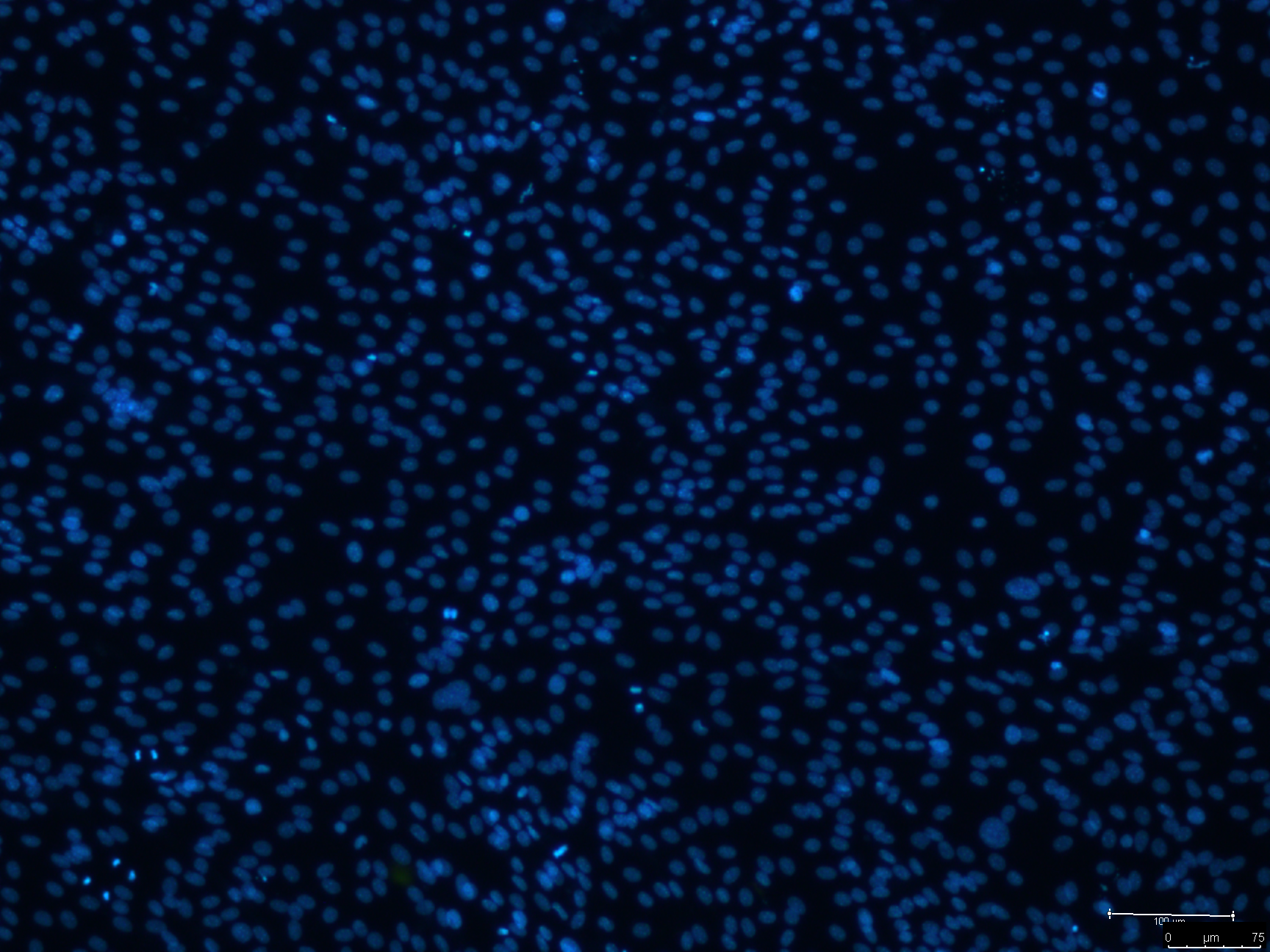

Supplement: Supplementary file 3 — Source data Fig. 1 [file 44321_2025_234_MOESM3_ESM.zip › Figure 1E/DUSP22 OE/dmso_Image018_ch00.tif]

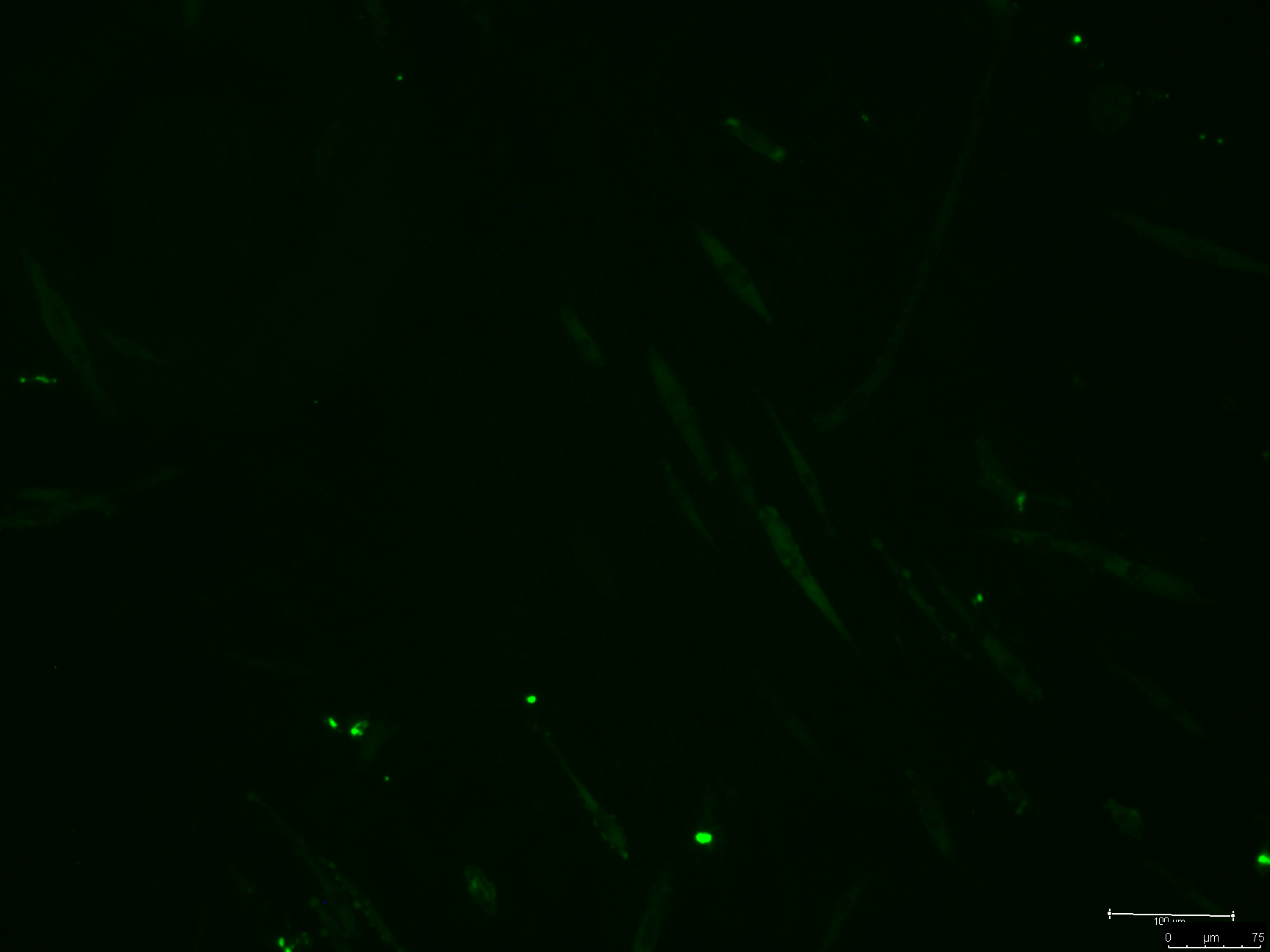

Supplement: Supplementary file 3 — Source data Fig. 1 [file 44321_2025_234_MOESM3_ESM.zip › Figure 1E/DUSP22 OE/dmso_Image019_ch00.tif]

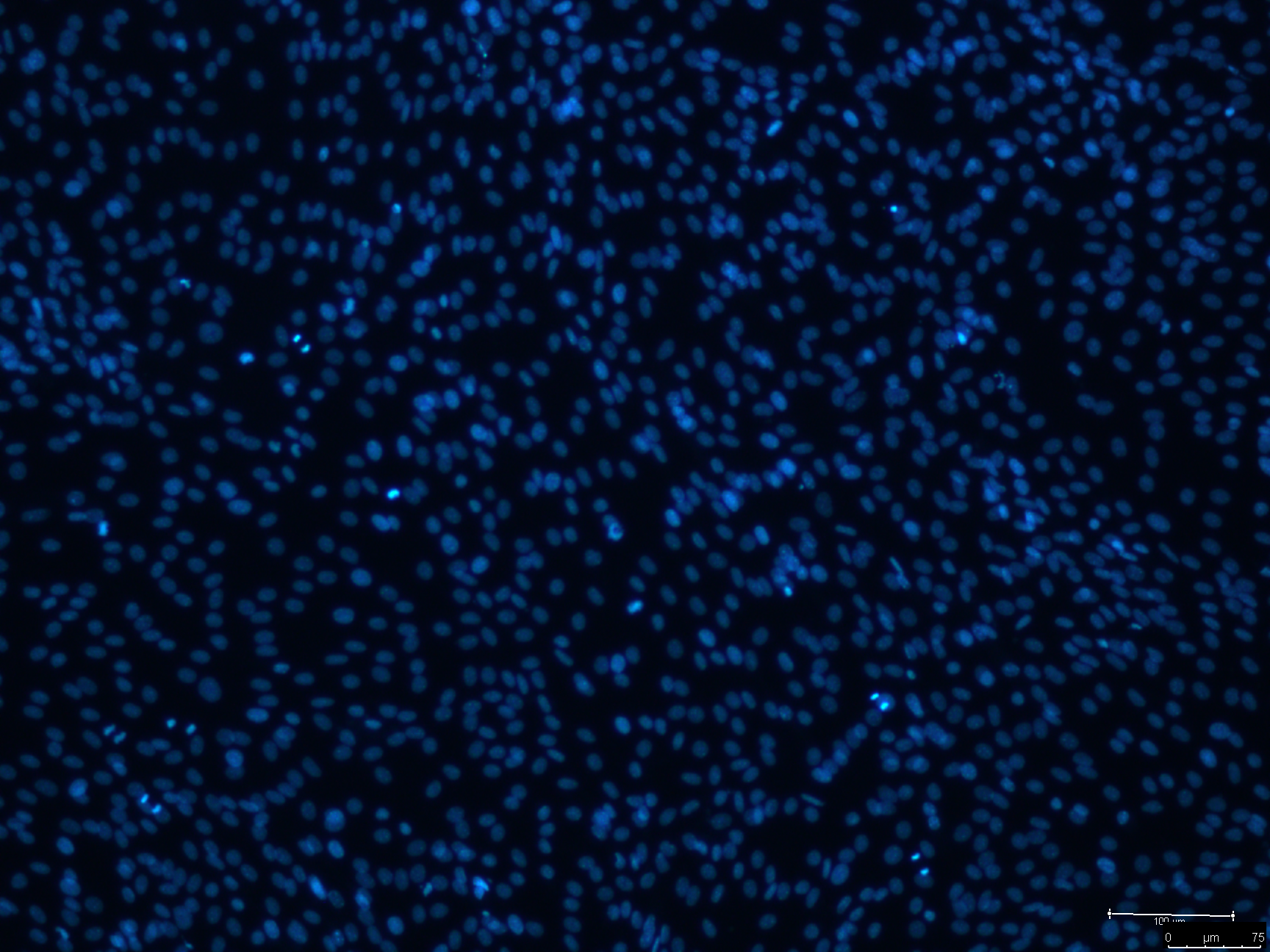

Supplement: Supplementary file 3 — Source data Fig. 1 [file 44321_2025_234_MOESM3_ESM.zip › Figure 1E/DUSP22 OE/dmso_Image020_ch00.tif]

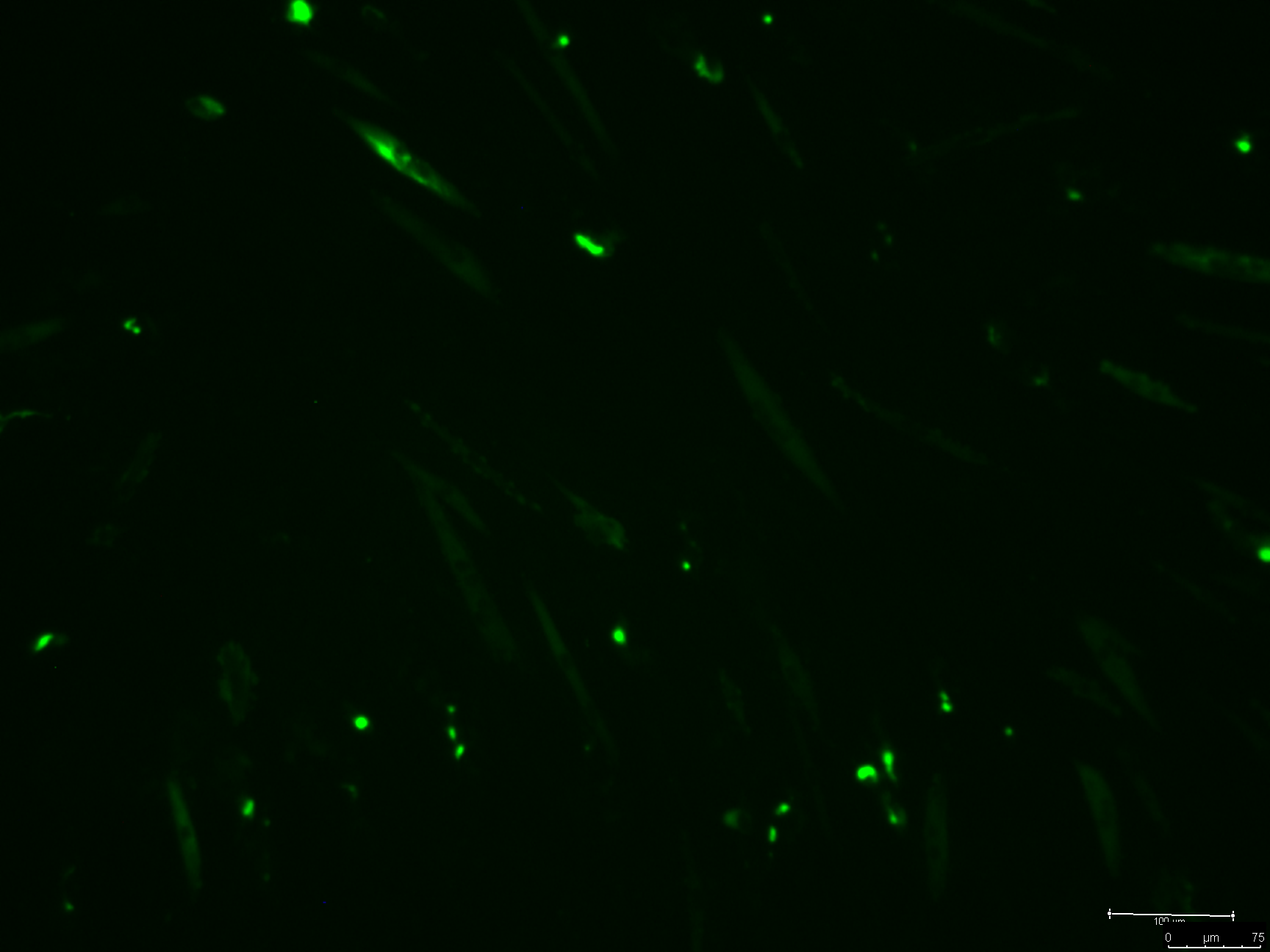

Supplement: Supplementary file 3 — Source data Fig. 1 [file 44321_2025_234_MOESM3_ESM.zip › Figure 1E/DUSP22 OE/dmso_Image021_ch00.tif]

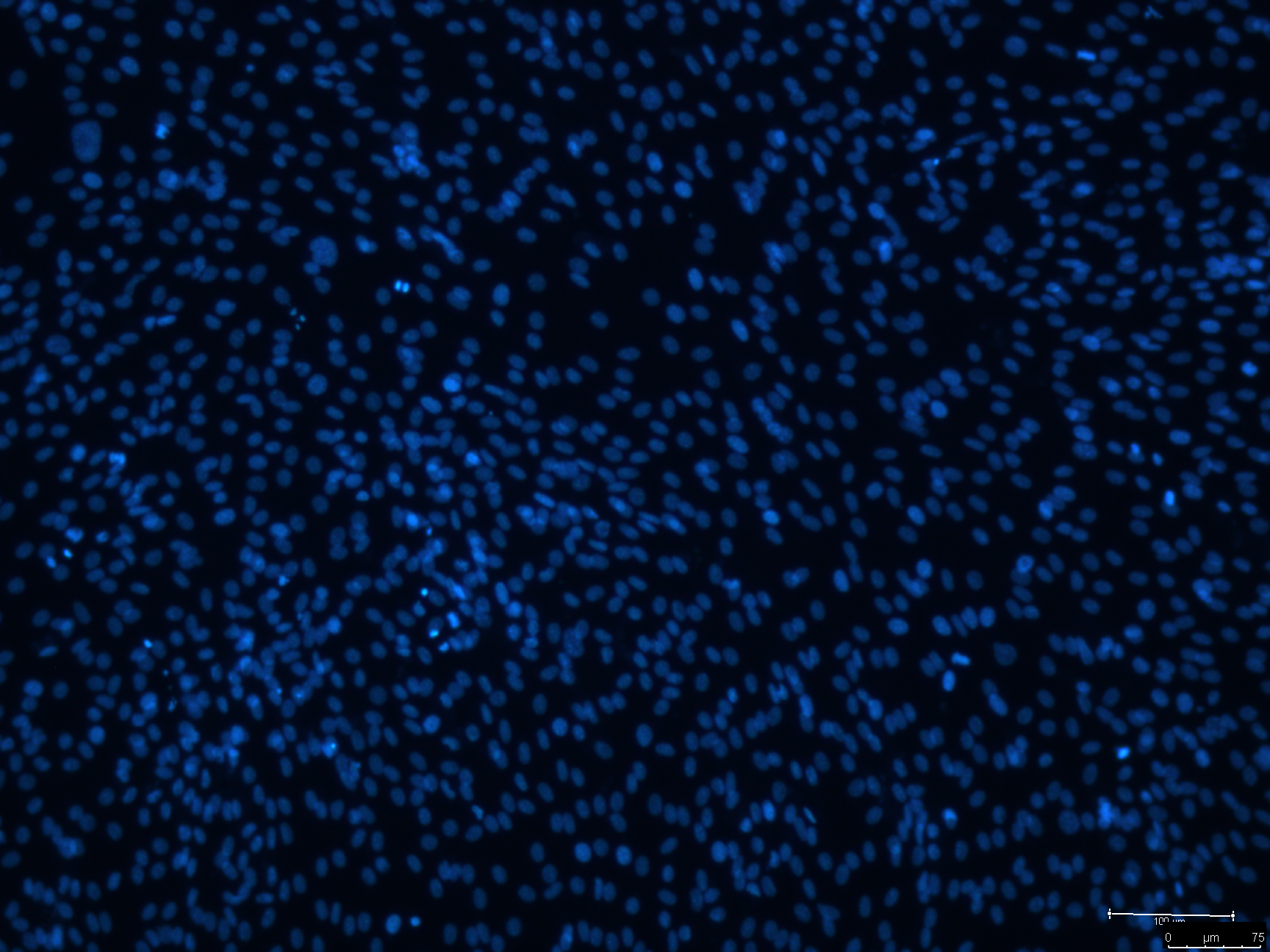

Supplement: Supplementary file 3 — Source data Fig. 1 [file 44321_2025_234_MOESM3_ESM.zip › Figure 1E/DUSP22 OE/dmso_Image022_ch00.tif]

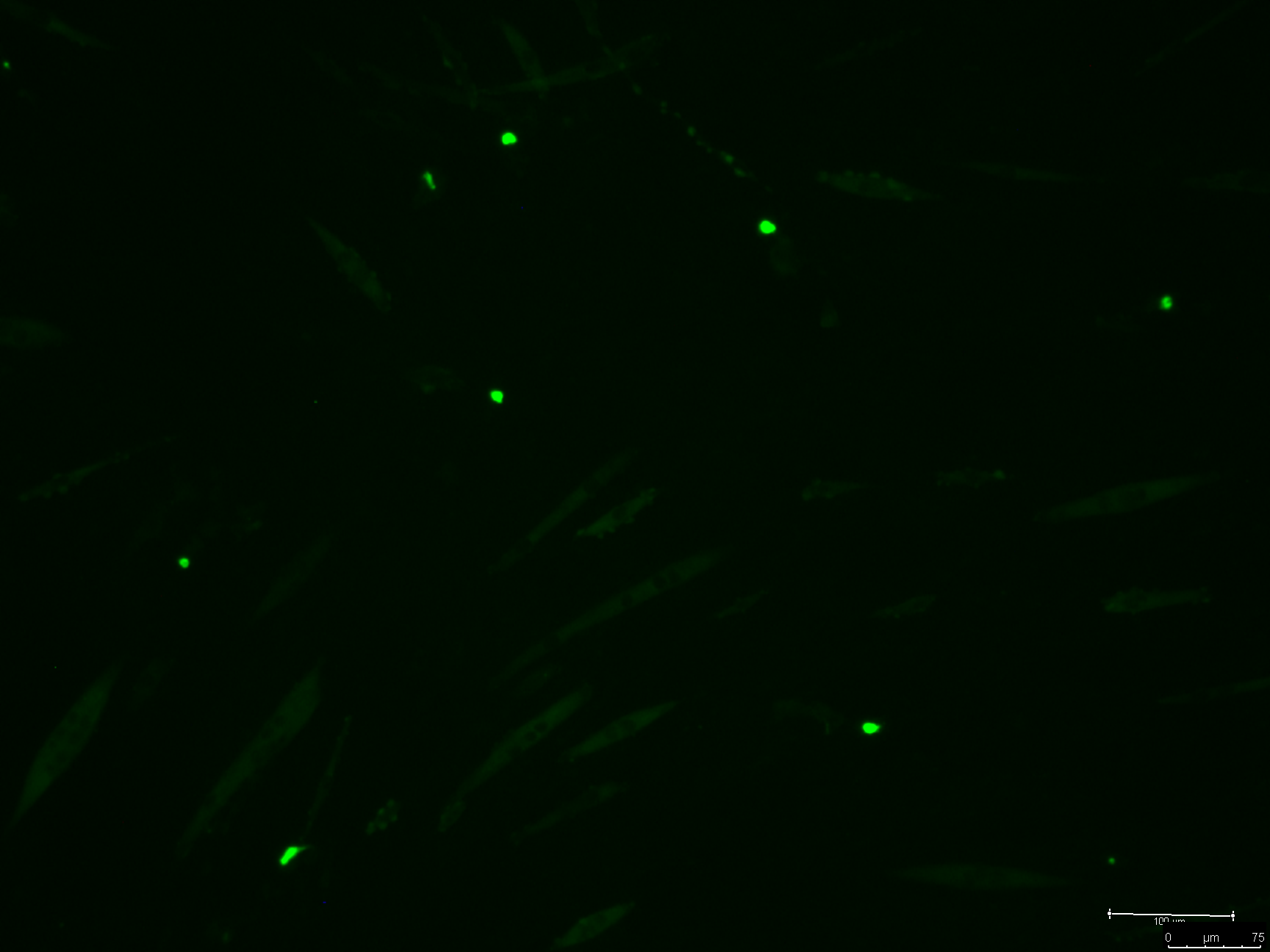

Supplement: Supplementary file 3 — Source data Fig. 1 [file 44321_2025_234_MOESM3_ESM.zip › Figure 1E/DUSP22 OE/dmso_Image023_ch00.tif]

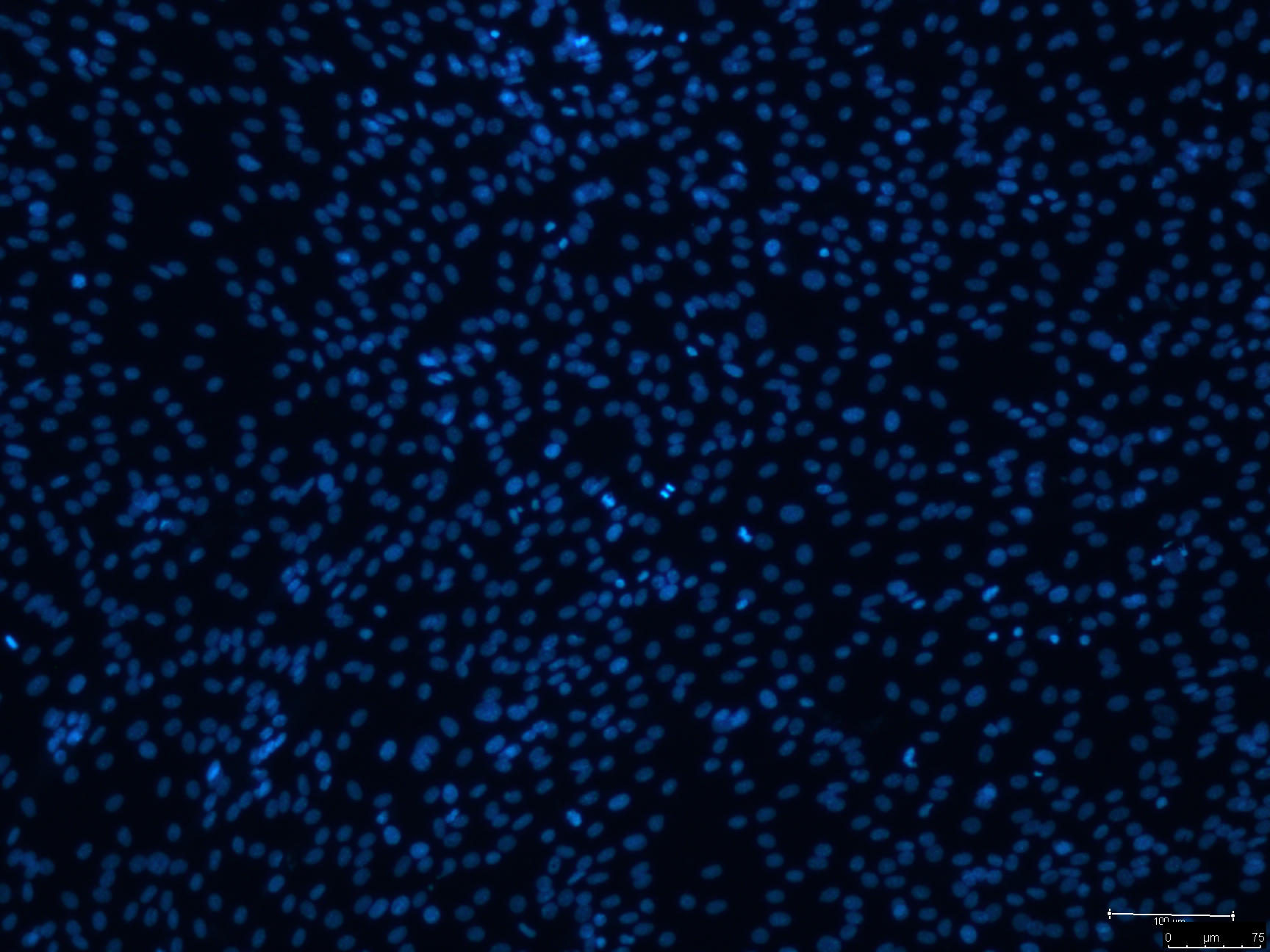

Supplement: Supplementary file 3 — Source data Fig. 1 [file 44321_2025_234_MOESM3_ESM.zip › Figure 1E/DUSP22 OE/dmso_Image024_ch00.tif]

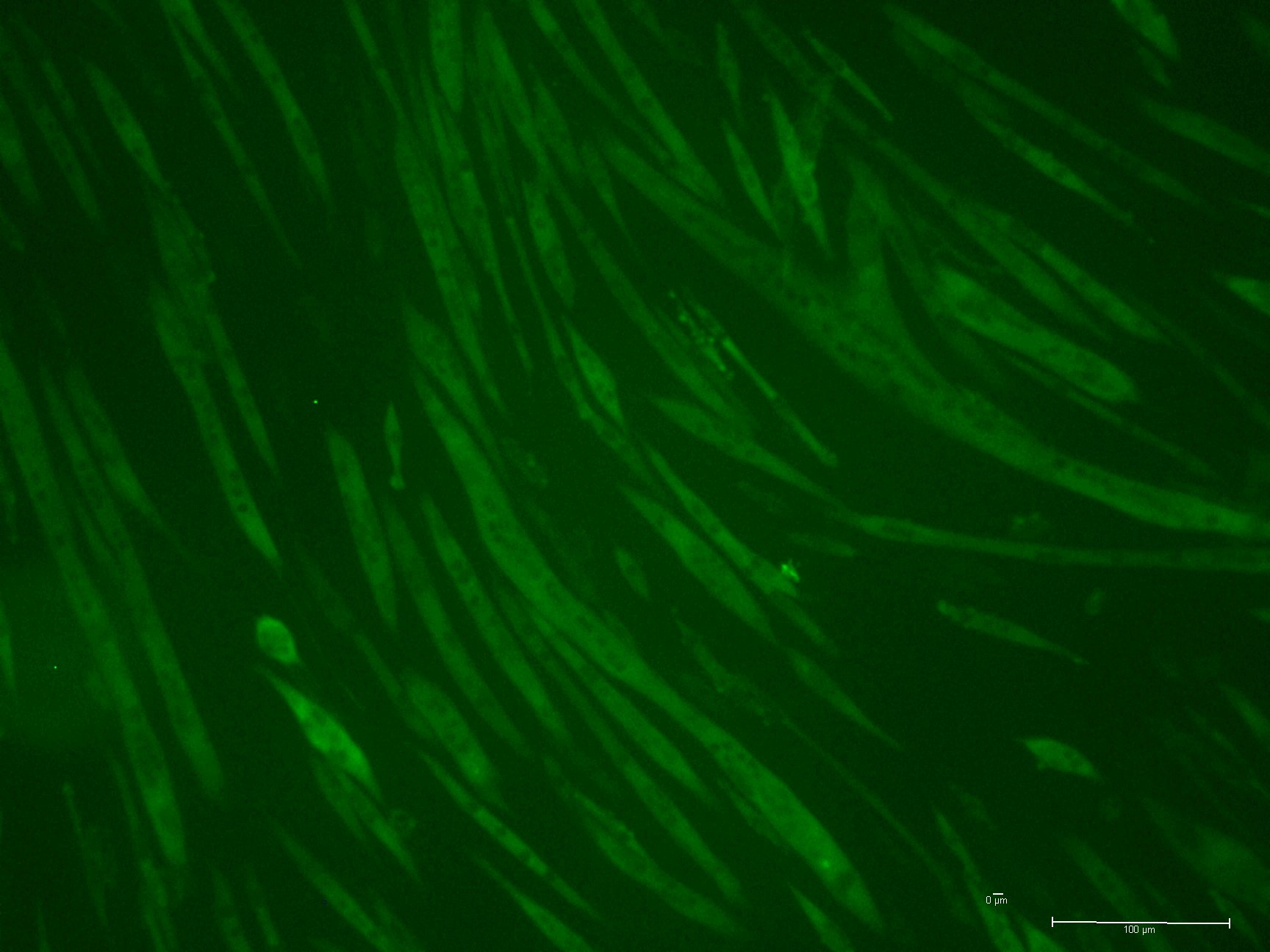

Supplement: Supplementary file 4 — Source data Fig. 2 [file 44321_2025_234_MOESM4_ESM.zip › Figure 2D/siCON/siCON1.jpg]

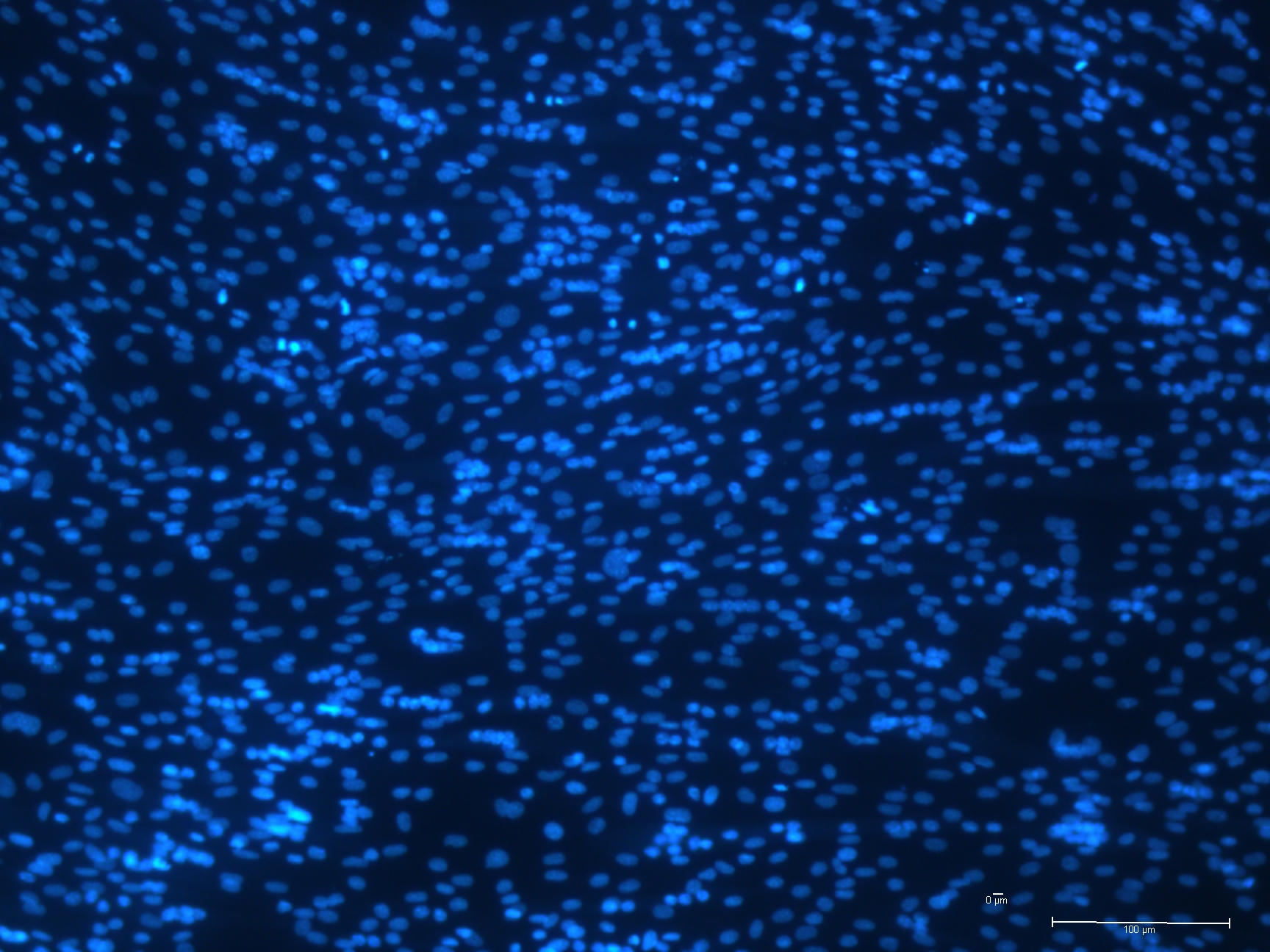

Supplement: Supplementary file 4 — Source data Fig. 2 [file 44321_2025_234_MOESM4_ESM.zip › Figure 2D/siCON/siCON2 DAPI.jpg]

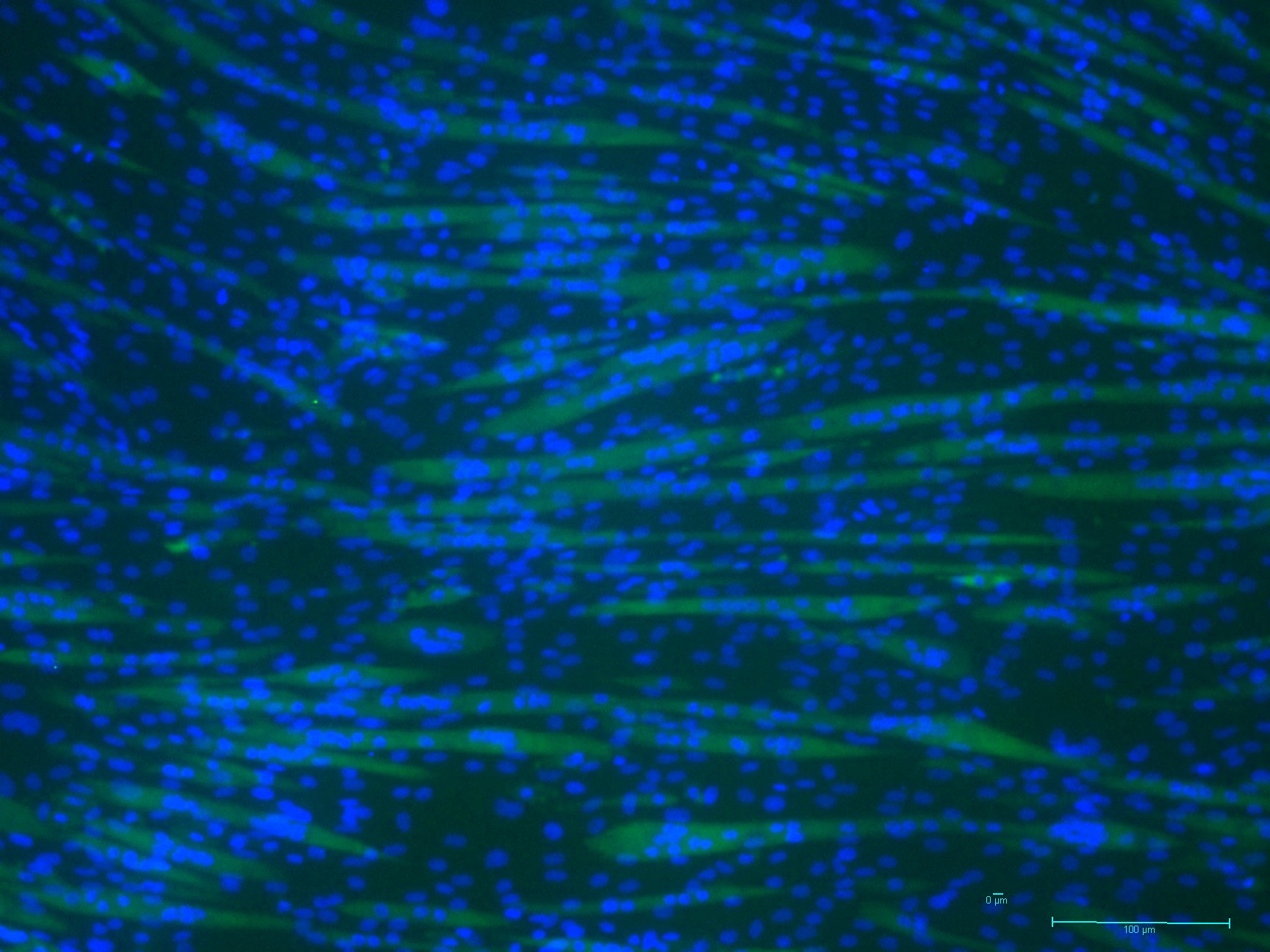

Supplement: Supplementary file 4 — Source data Fig. 2 [file 44321_2025_234_MOESM4_ESM.zip › Figure 2D/siCON/siCON2 merge.jpg]

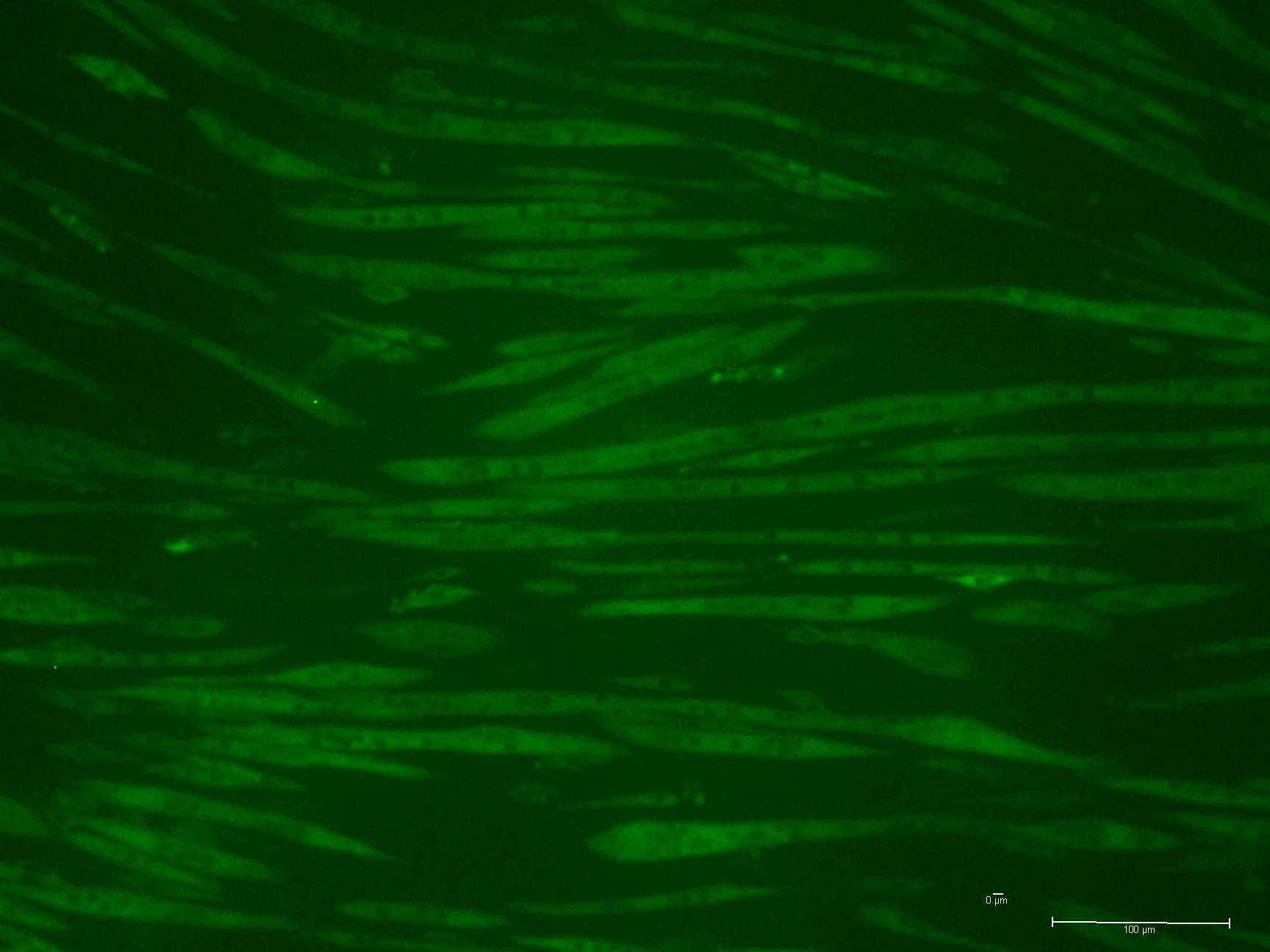

Supplement: Supplementary file 4 — Source data Fig. 2 [file 44321_2025_234_MOESM4_ESM.zip › Figure 2D/siCON/siCON2.jpg]

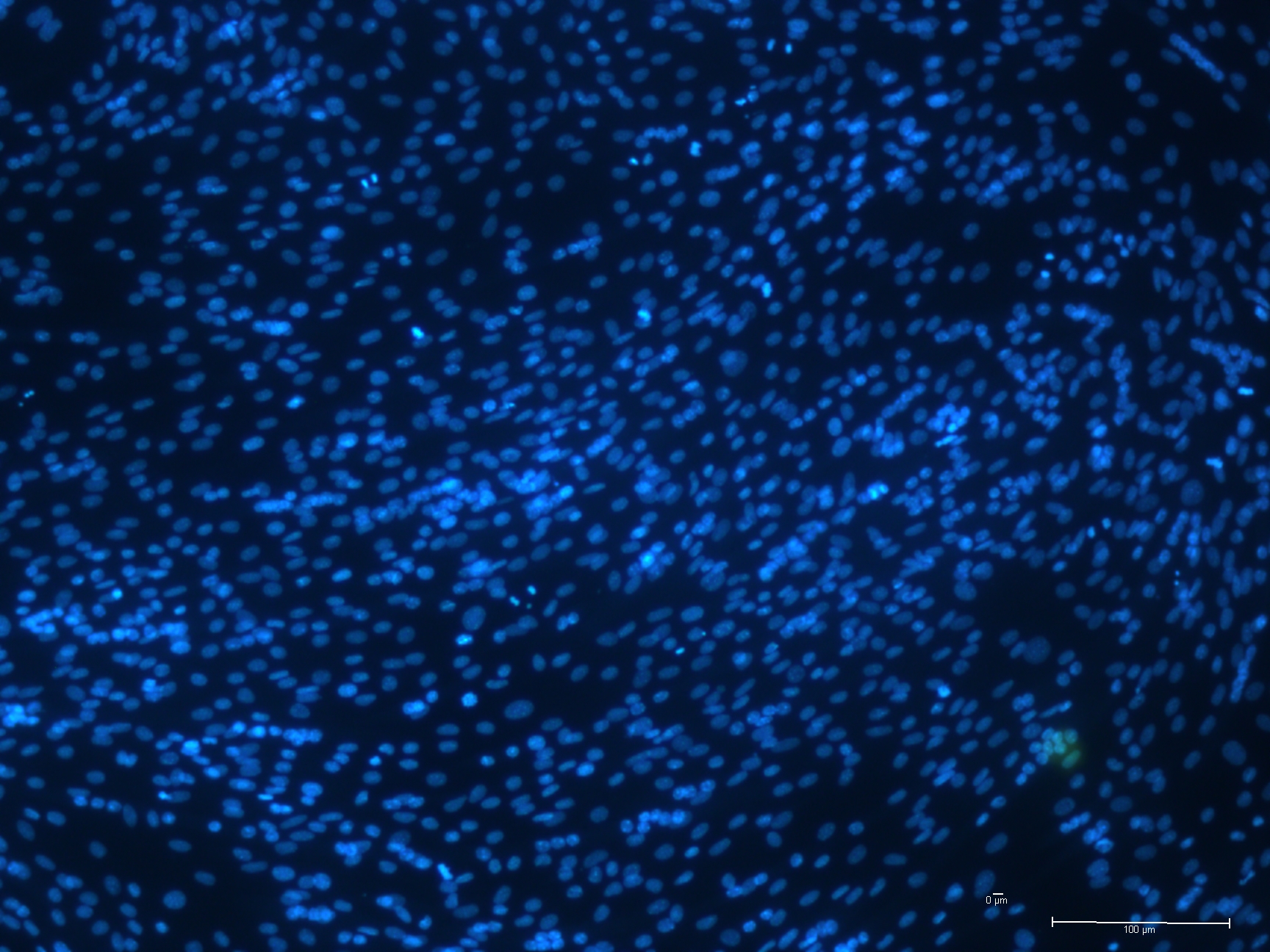

Supplement: Supplementary file 4 — Source data Fig. 2 [file 44321_2025_234_MOESM4_ESM.zip › Figure 2D/siCON/siCON3 DAPI.jpg]

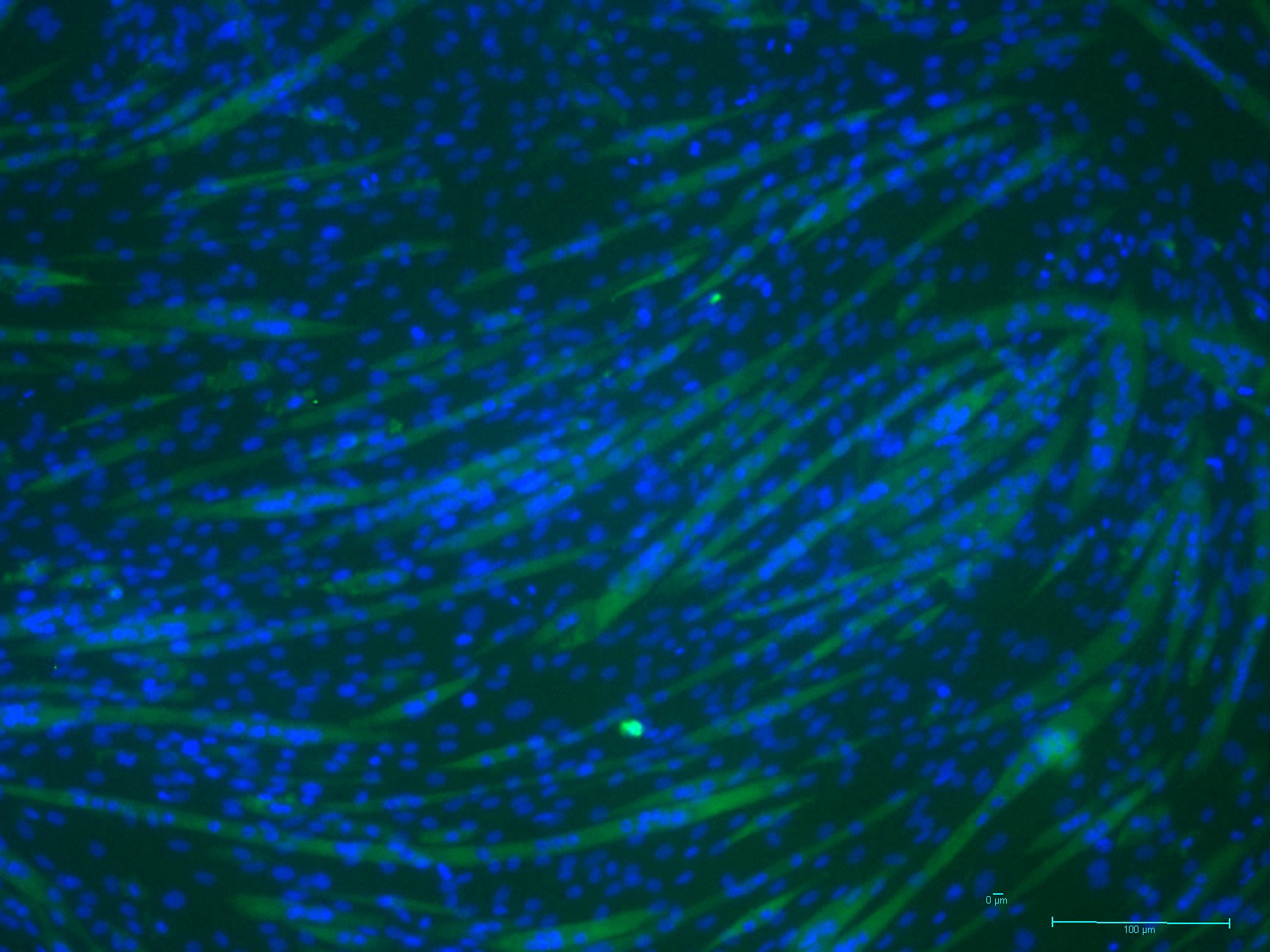

Supplement: Supplementary file 4 — Source data Fig. 2 [file 44321_2025_234_MOESM4_ESM.zip › Figure 2D/siCON/siCON3 merge.jpg]

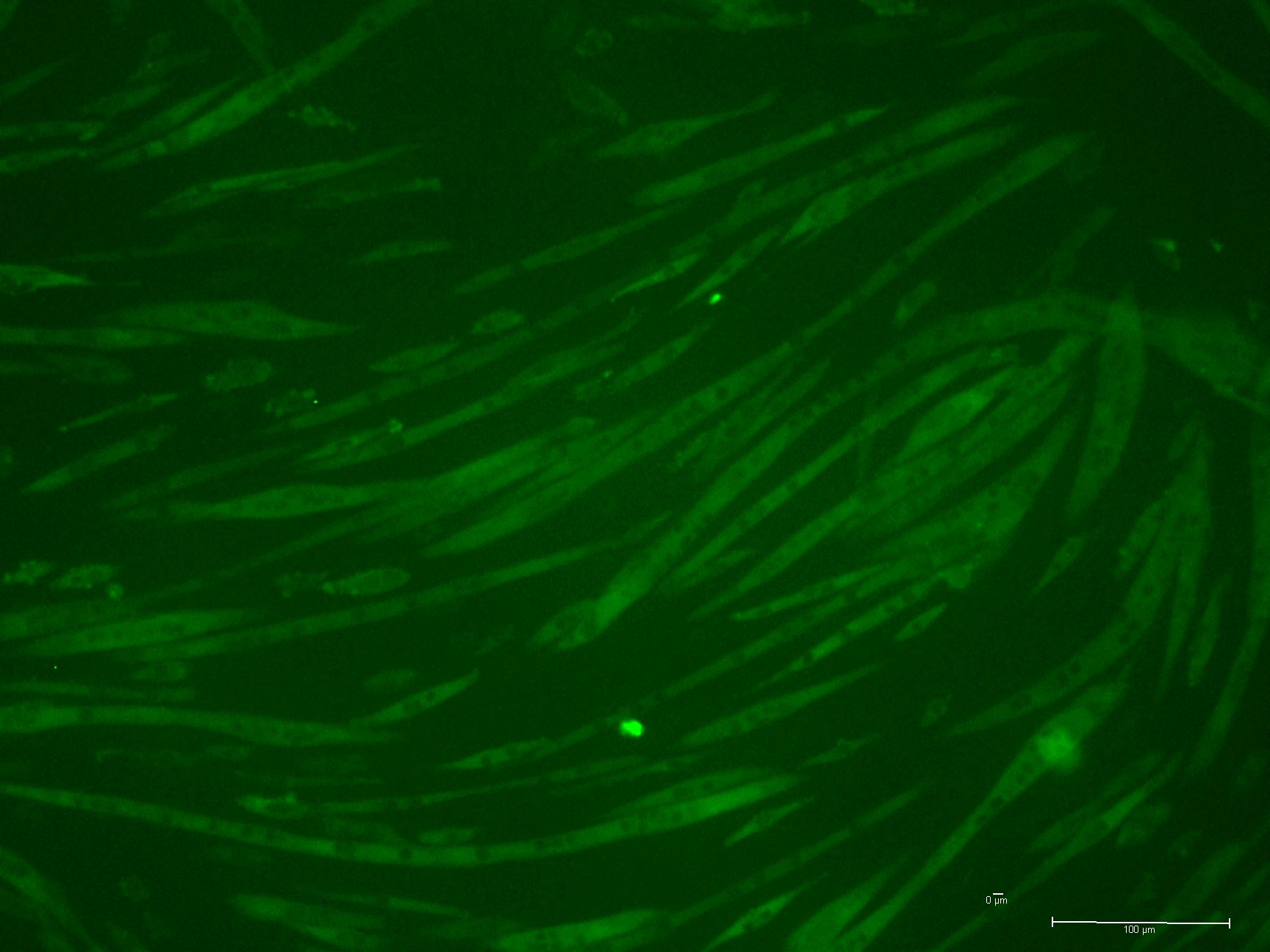

Supplement: Supplementary file 4 — Source data Fig. 2 [file 44321_2025_234_MOESM4_ESM.zip › Figure 2D/siCON/siCON3.jpg]

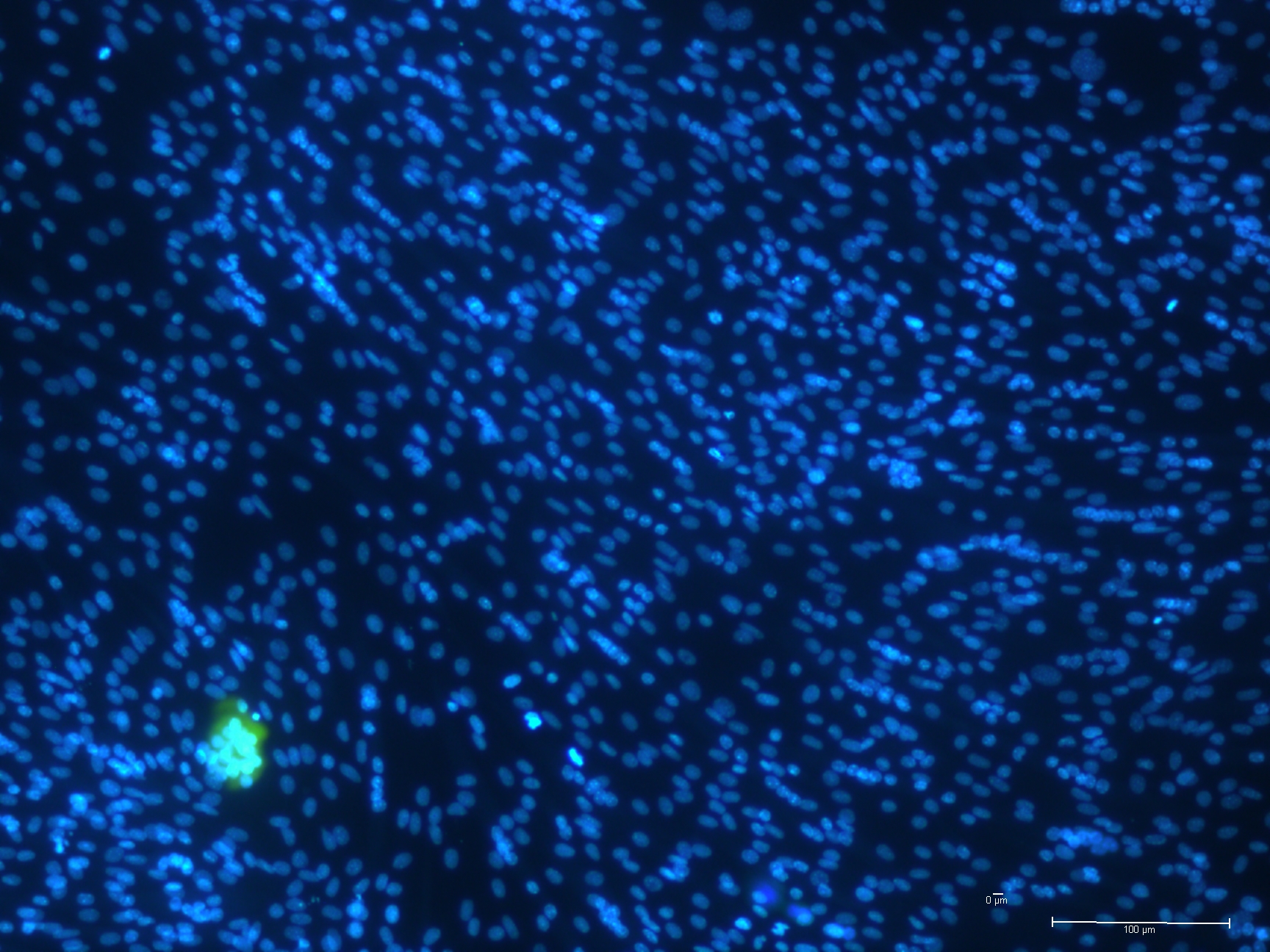

Supplement: Supplementary file 4 — Source data Fig. 2 [file 44321_2025_234_MOESM4_ESM.zip › Figure 2D/siCON/siCON4 DAPI.jpg]

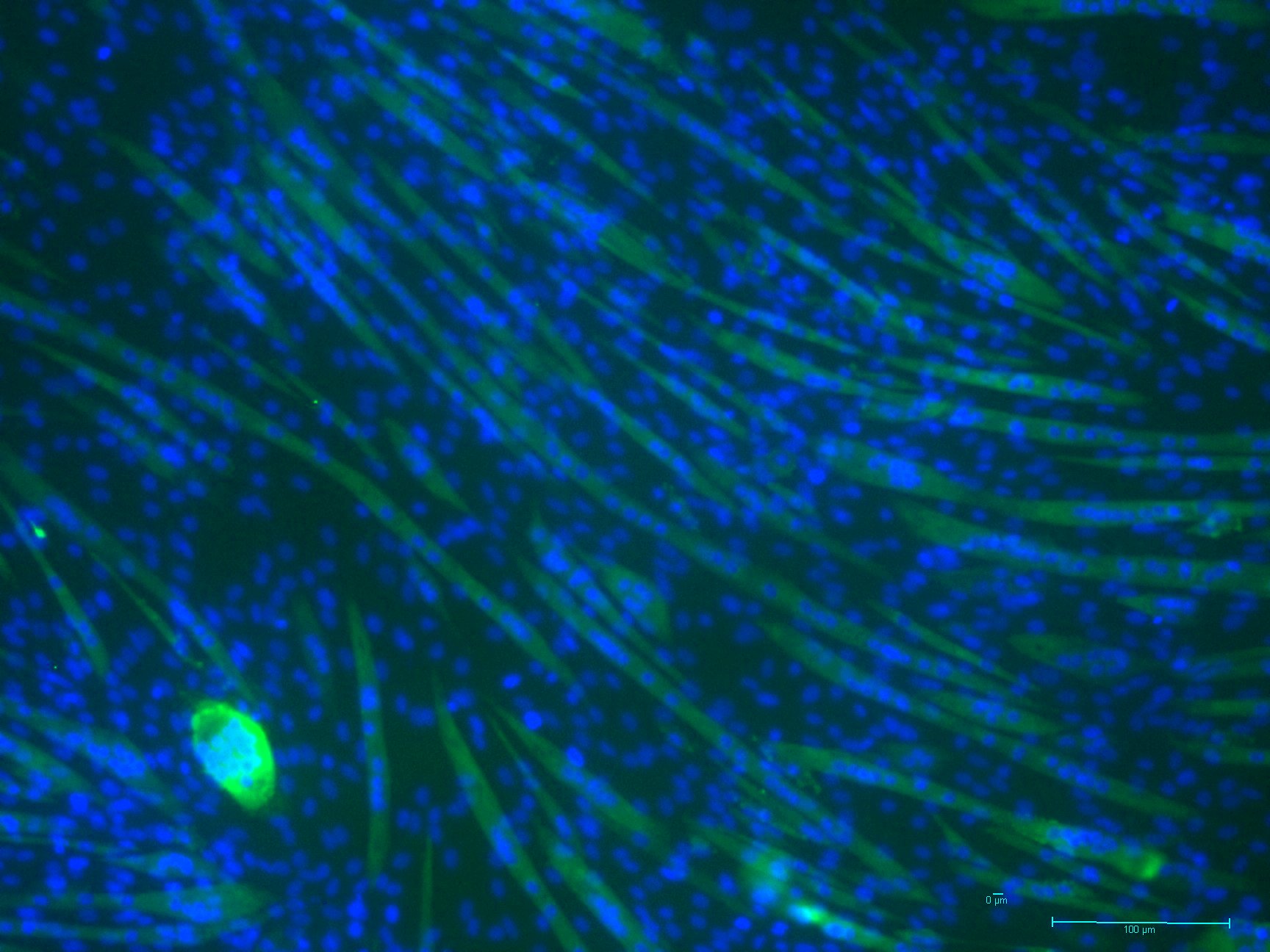

Supplement: Supplementary file 4 — Source data Fig. 2 [file 44321_2025_234_MOESM4_ESM.zip › Figure 2D/siCON/siCON4 merge.jpg]

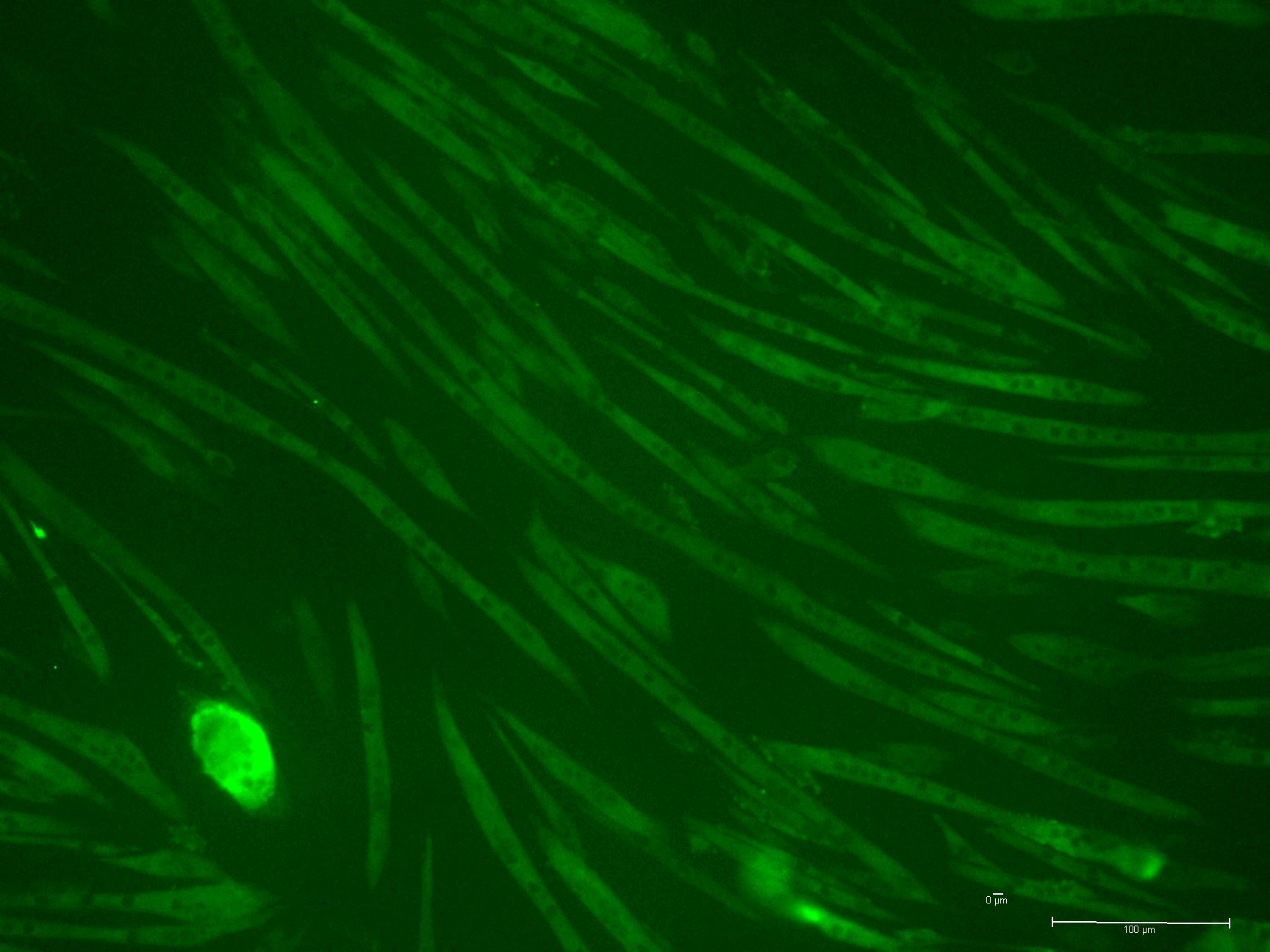

Supplement: Supplementary file 4 — Source data Fig. 2 [file 44321_2025_234_MOESM4_ESM.zip › Figure 2D/siCON/siCON4.jpg]

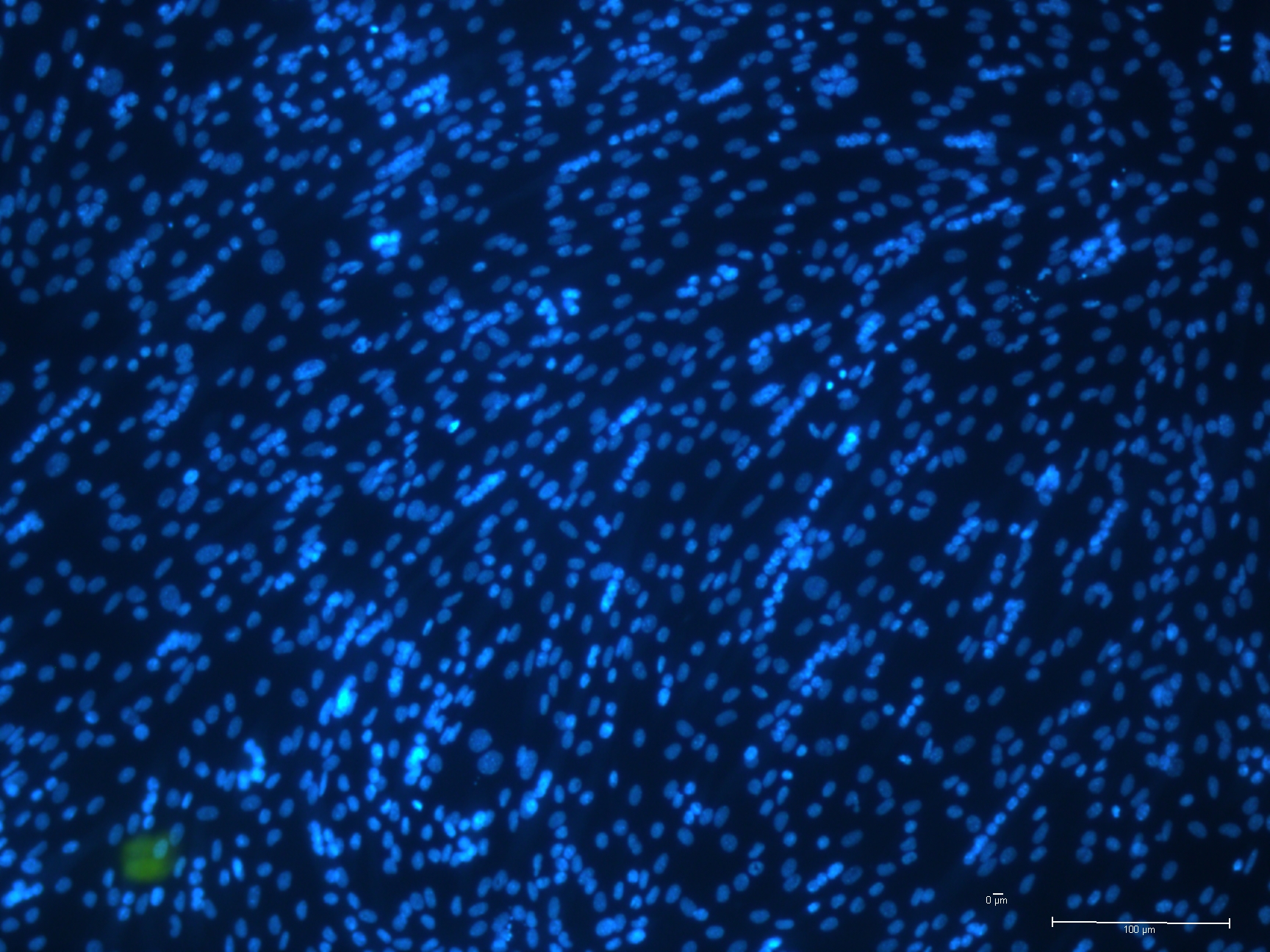

Supplement: Supplementary file 4 — Source data Fig. 2 [file 44321_2025_234_MOESM4_ESM.zip › Figure 2D/siCON+Dex/siCON+Dex1 DAPI.jpg]

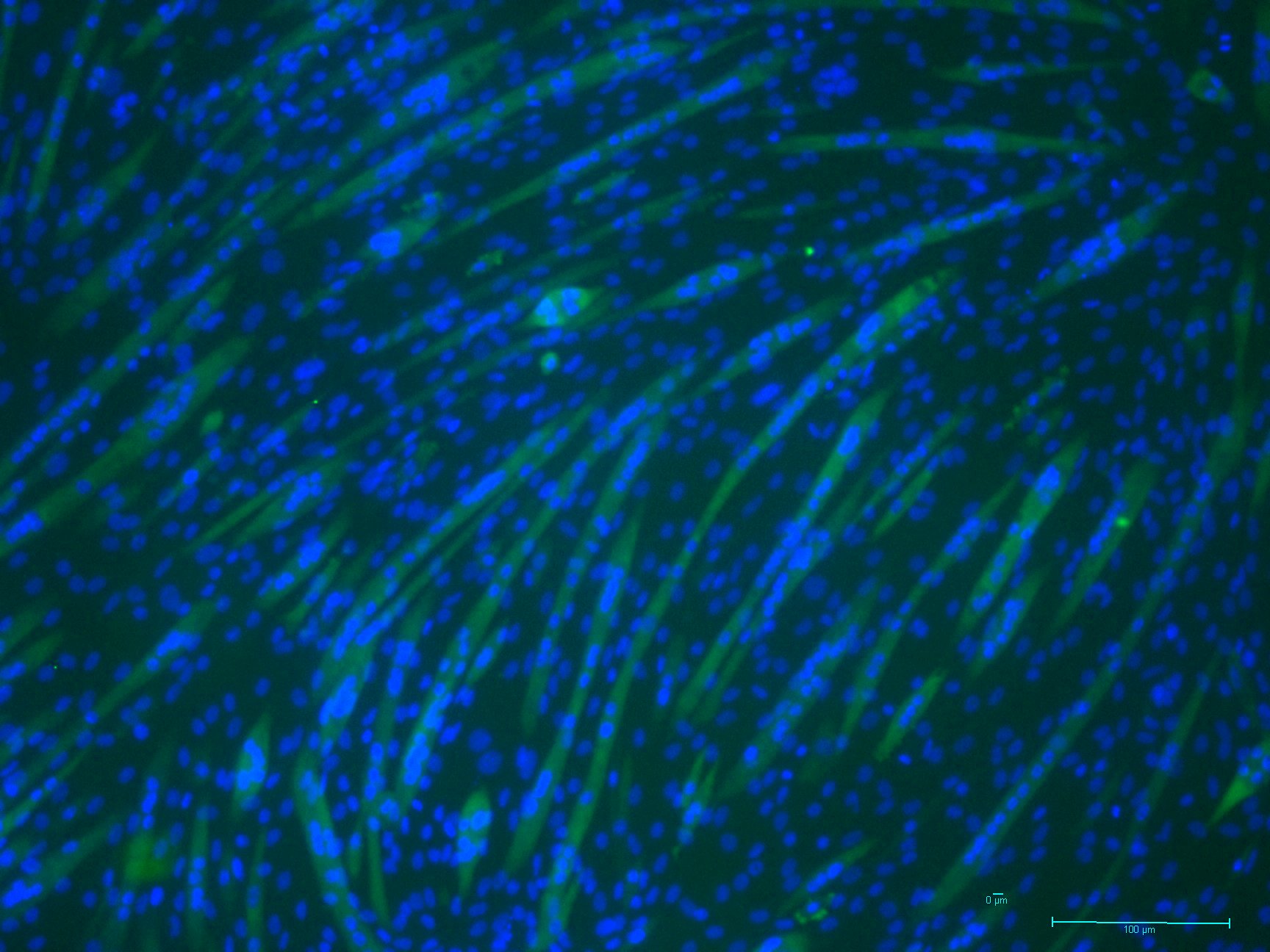

Supplement: Supplementary file 4 — Source data Fig. 2 [file 44321_2025_234_MOESM4_ESM.zip › Figure 2D/siCON+Dex/siCON+Dex1 merge.jpg]

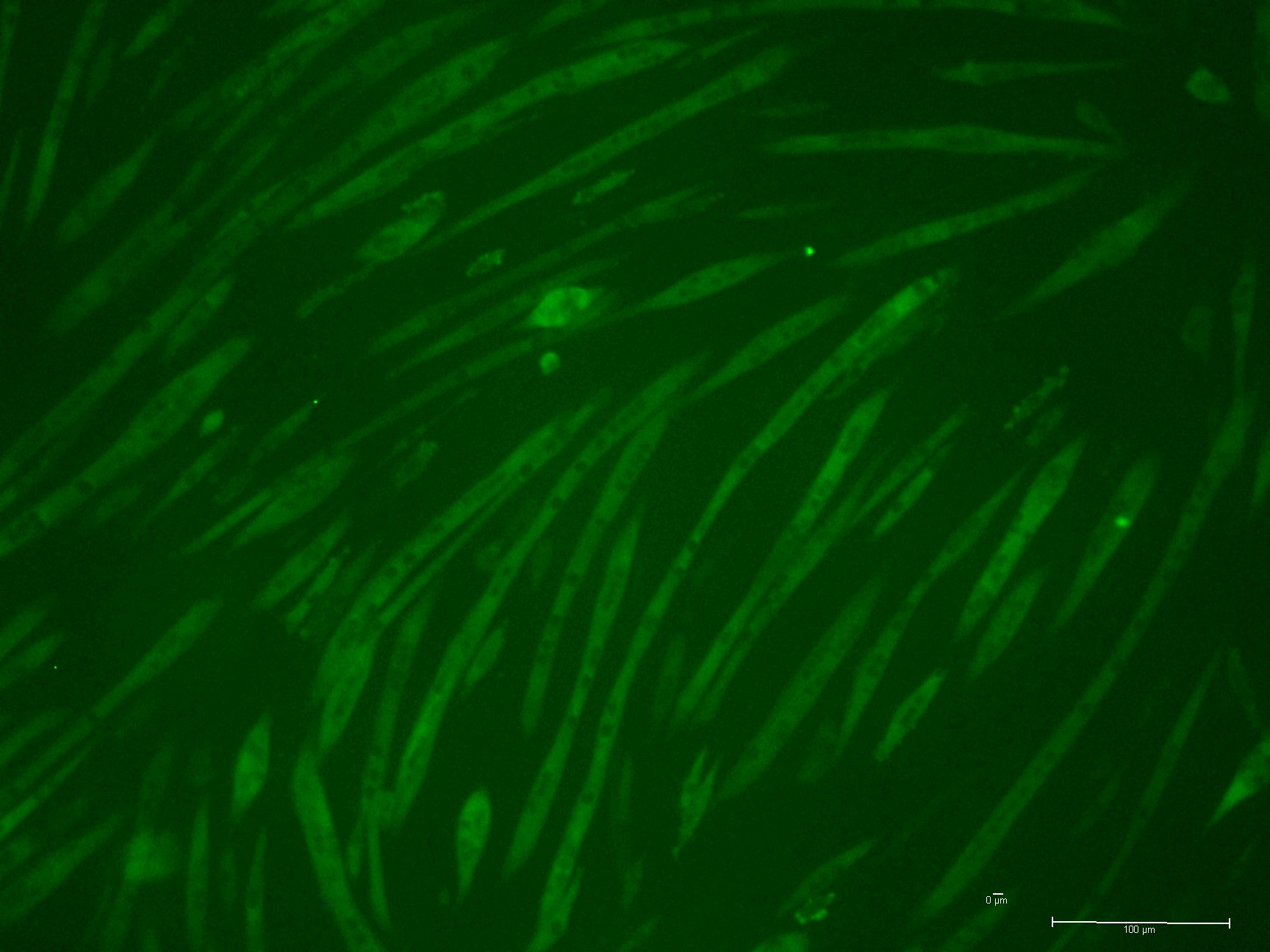

Supplement: Supplementary file 4 — Source data Fig. 2 [file 44321_2025_234_MOESM4_ESM.zip › Figure 2D/siCON+Dex/siCON+Dex1.jpg]

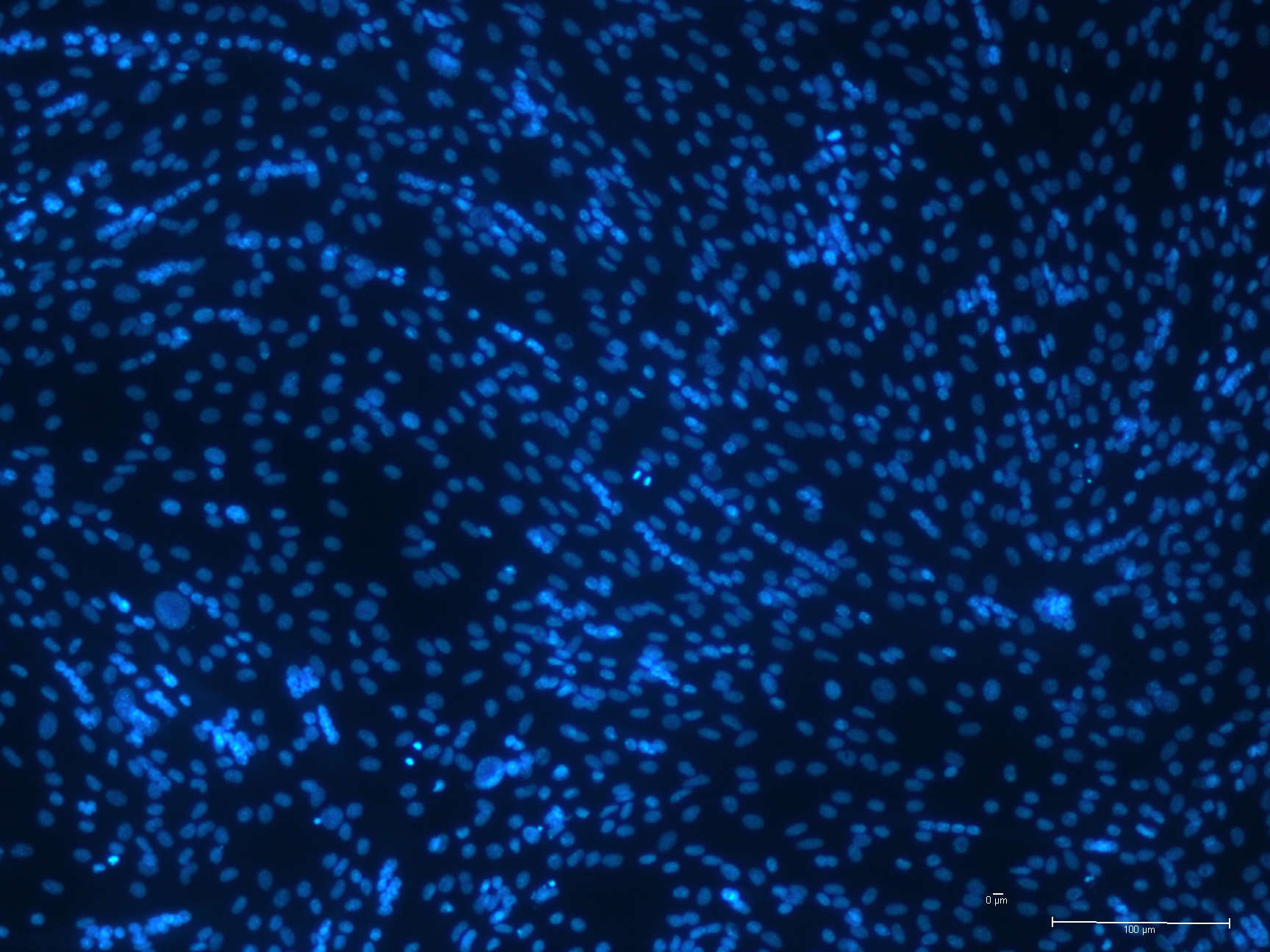

Supplement: Supplementary file 4 — Source data Fig. 2 [file 44321_2025_234_MOESM4_ESM.zip › Figure 2D/siCON+Dex/siCON+Dex2 DAPI.jpg]

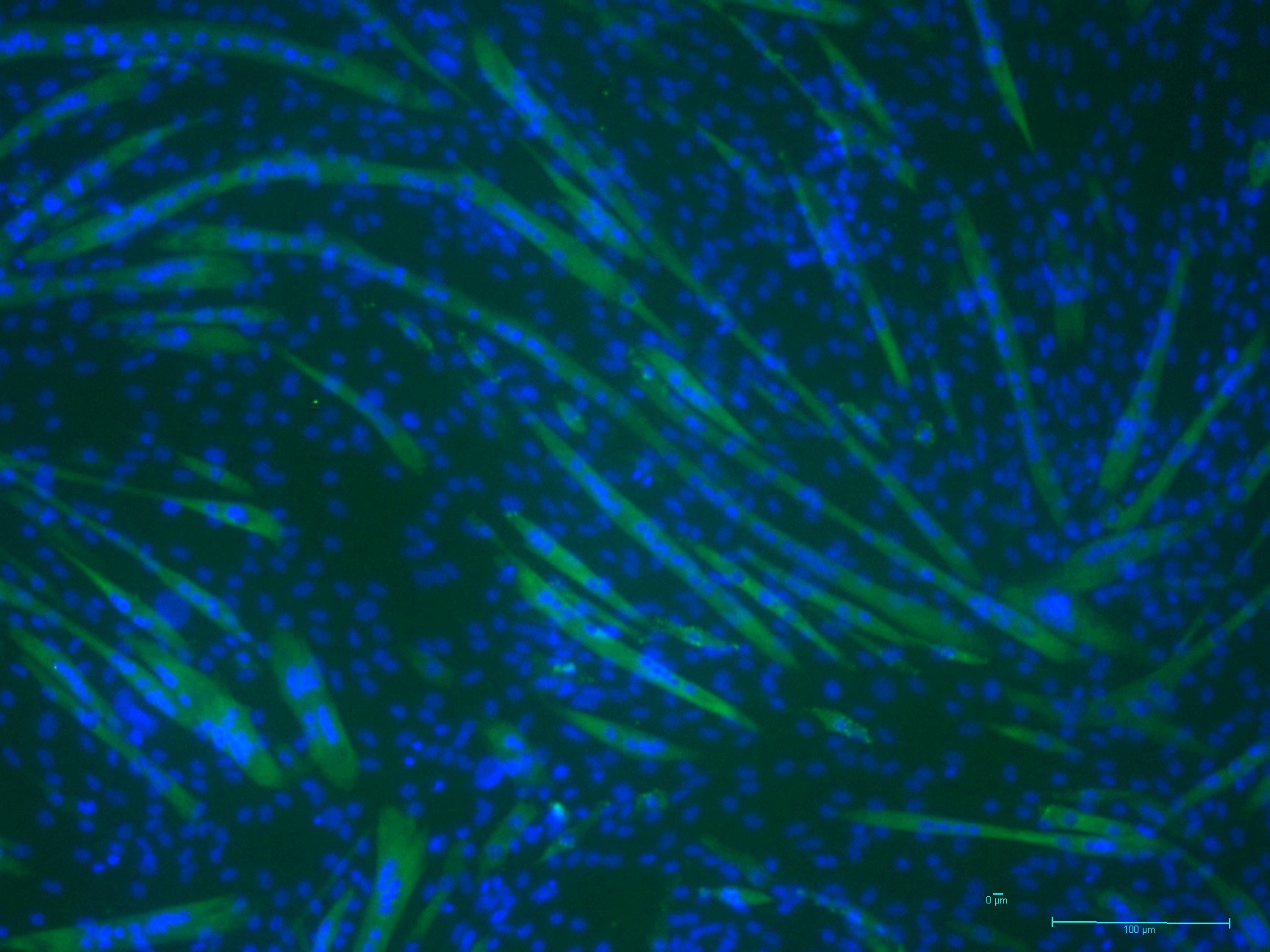

Supplement: Supplementary file 4 — Source data Fig. 2 [file 44321_2025_234_MOESM4_ESM.zip › Figure 2D/siCON+Dex/siCON+Dex2 merge.jpg]

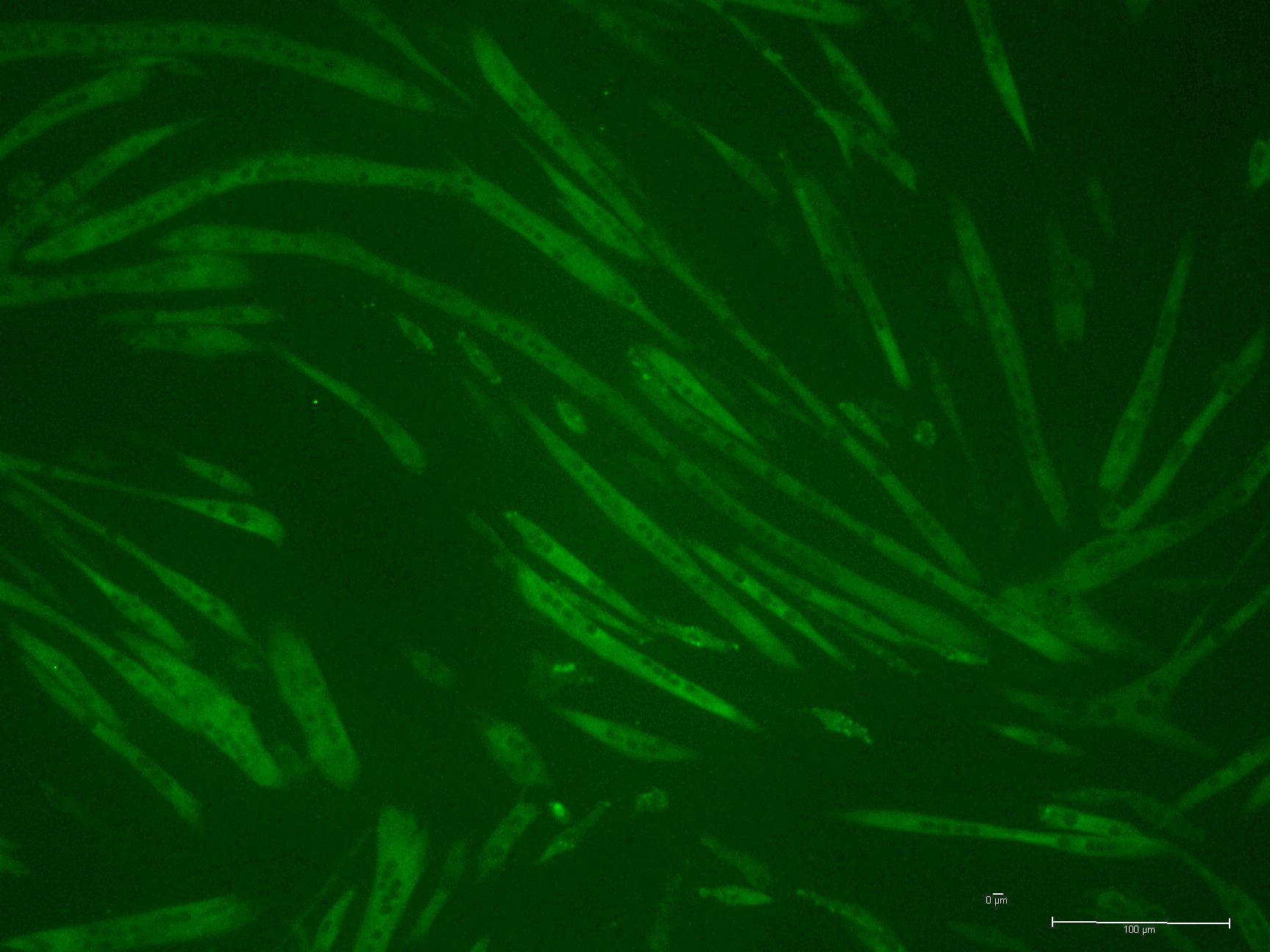

Supplement: Supplementary file 4 — Source data Fig. 2 [file 44321_2025_234_MOESM4_ESM.zip › Figure 2D/siCON+Dex/siCON+Dex2.jpg]

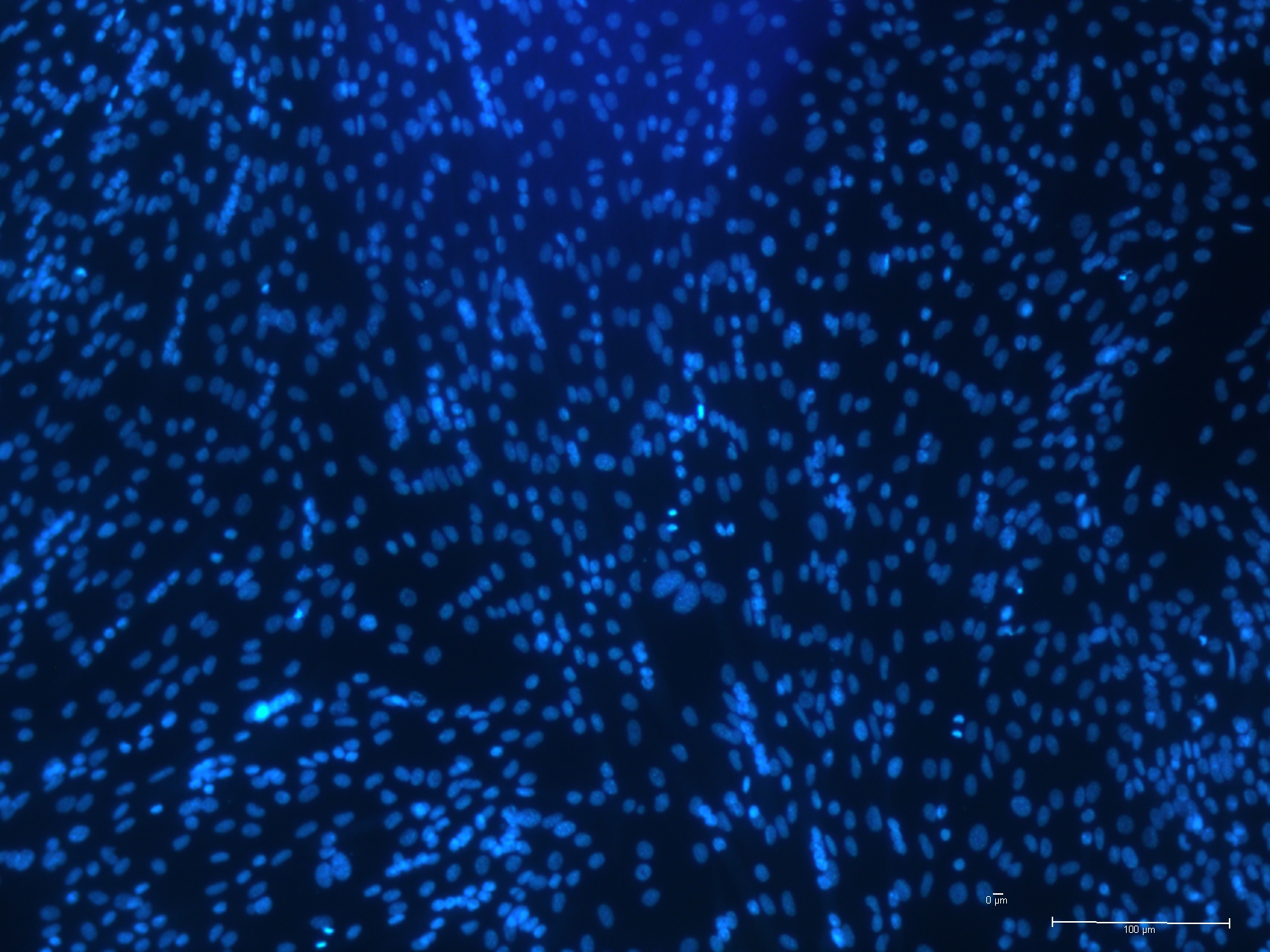

Supplement: Supplementary file 4 — Source data Fig. 2 [file 44321_2025_234_MOESM4_ESM.zip › Figure 2D/siCON+Dex/siCON+Dex3 DAPI.jpg]

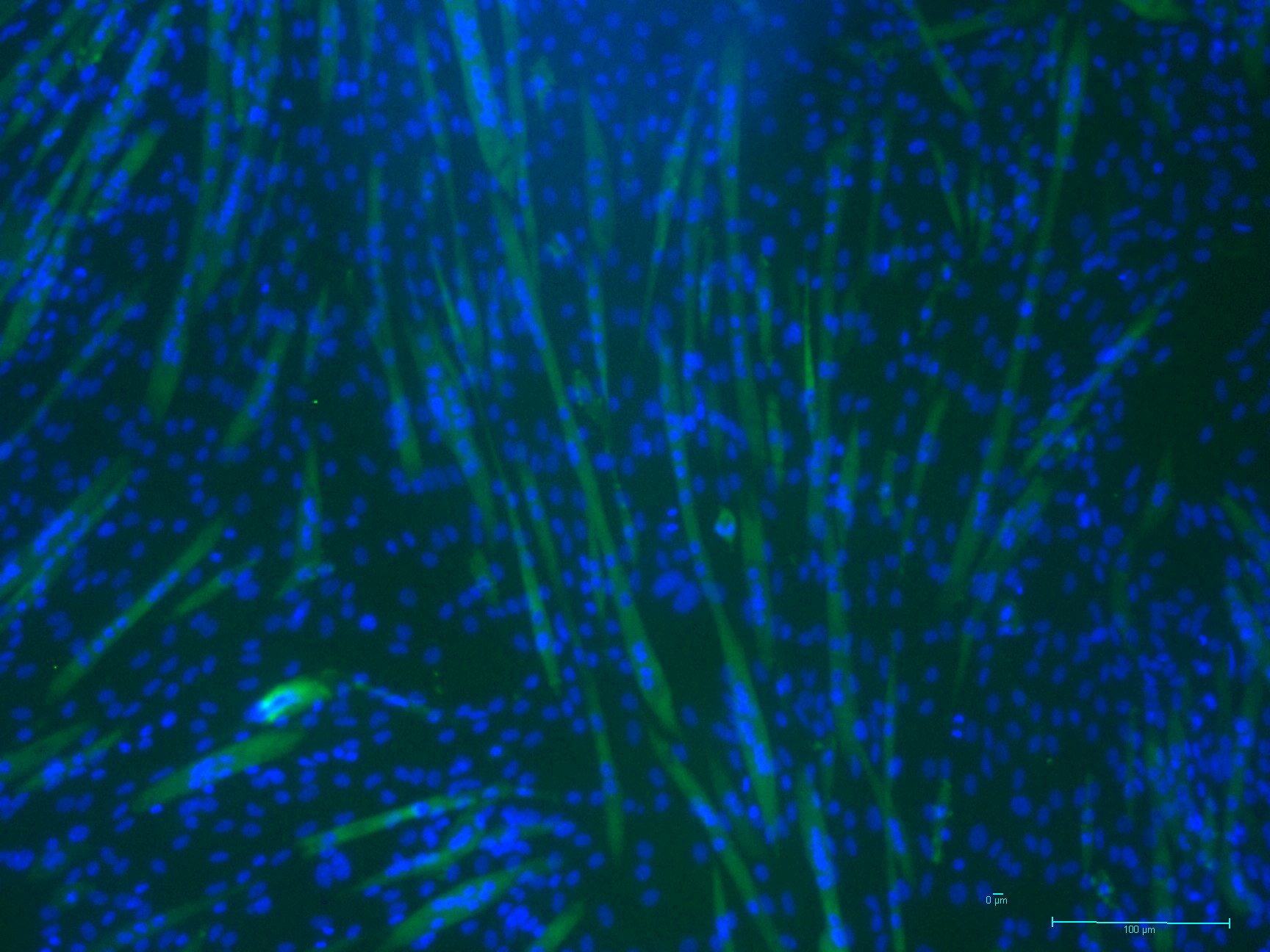

Supplement: Supplementary file 4 — Source data Fig. 2 [file 44321_2025_234_MOESM4_ESM.zip › Figure 2D/siCON+Dex/siCON+Dex3 merge.jpg]

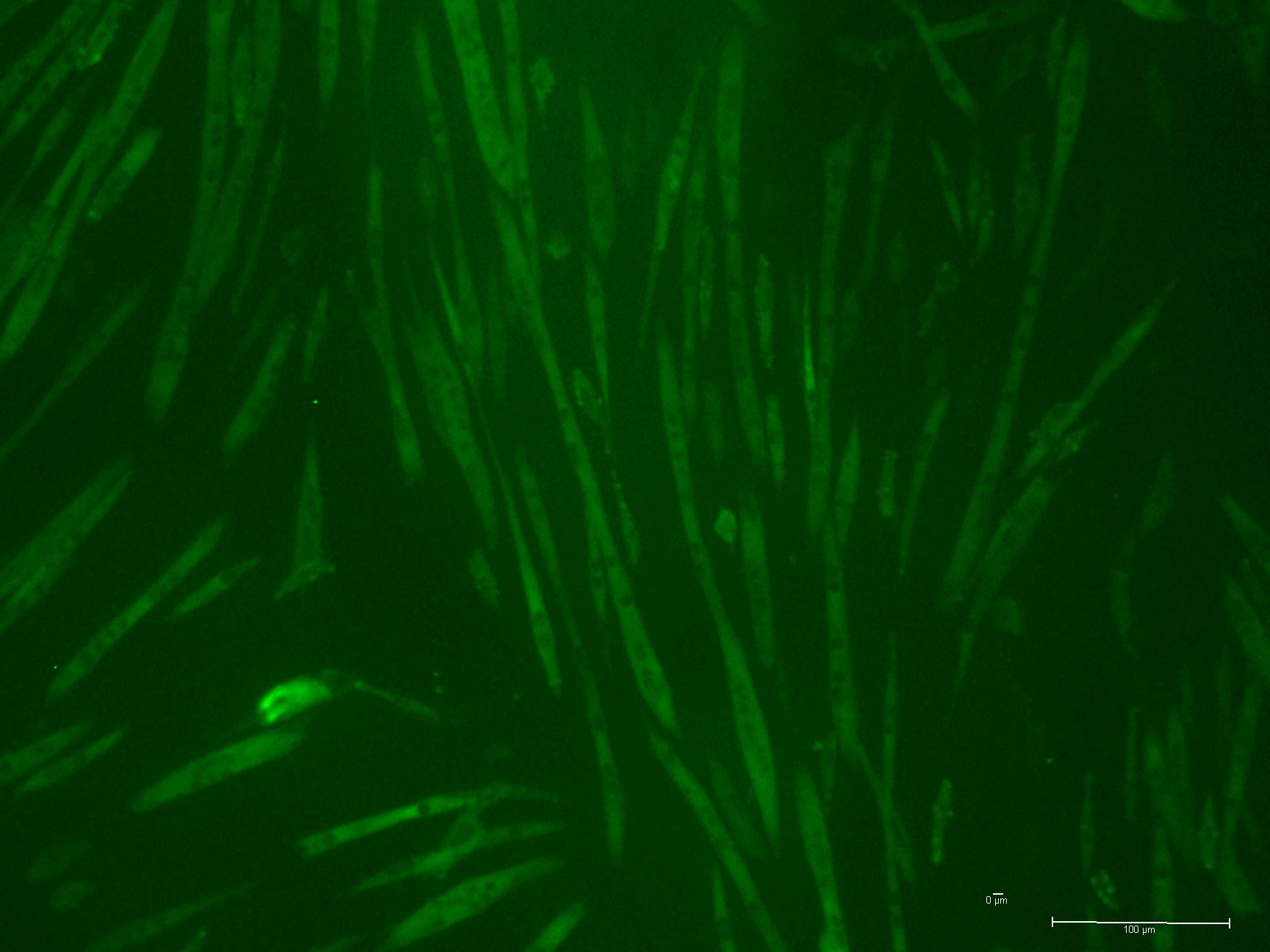

Supplement: Supplementary file 4 — Source data Fig. 2 [file 44321_2025_234_MOESM4_ESM.zip › Figure 2D/siCON+Dex/siCON+Dex3.jpg]

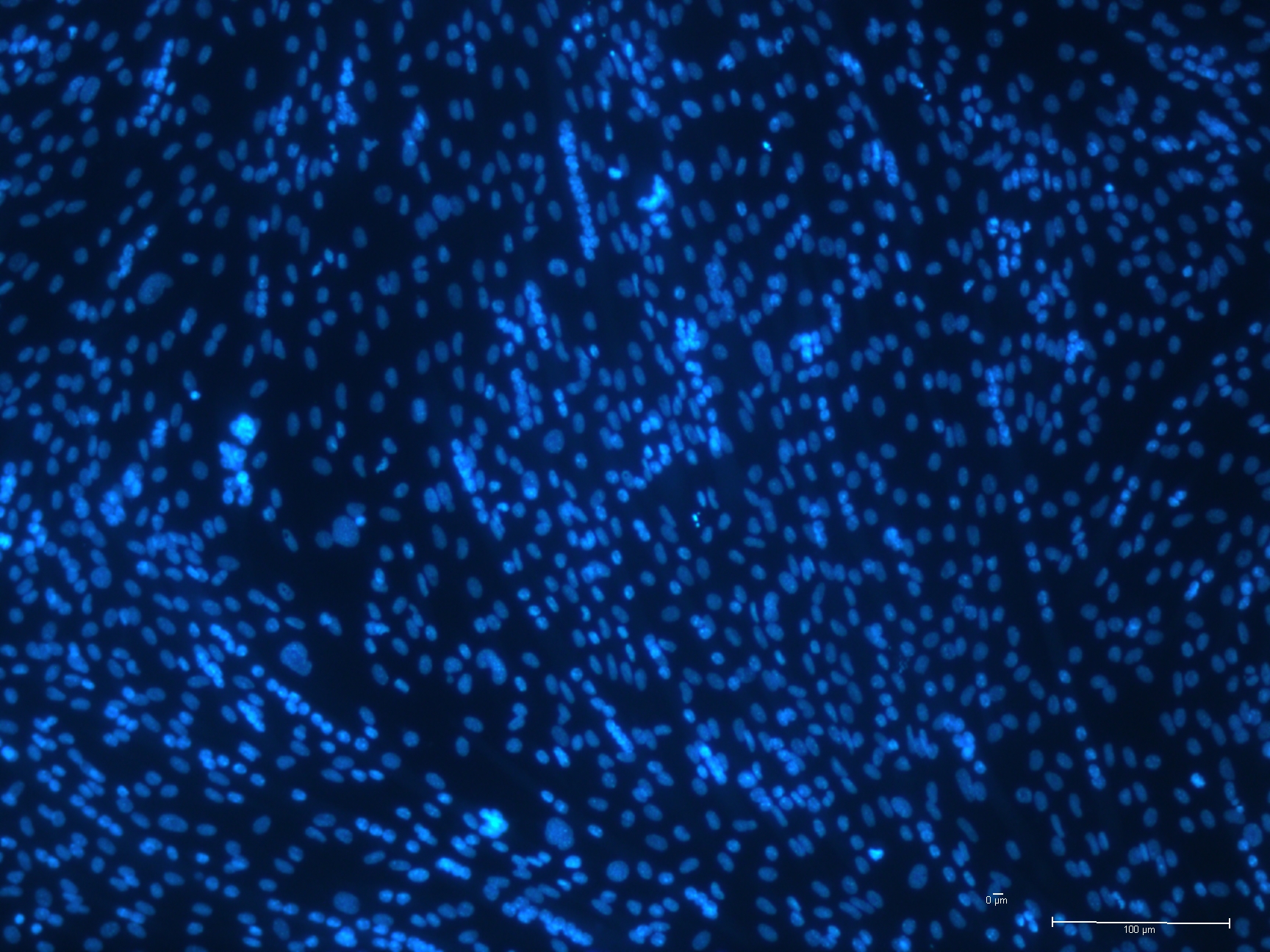

Supplement: Supplementary file 4 — Source data Fig. 2 [file 44321_2025_234_MOESM4_ESM.zip › Figure 2D/siCON+Dex/siCON+Dex4 DAPI.jpg]

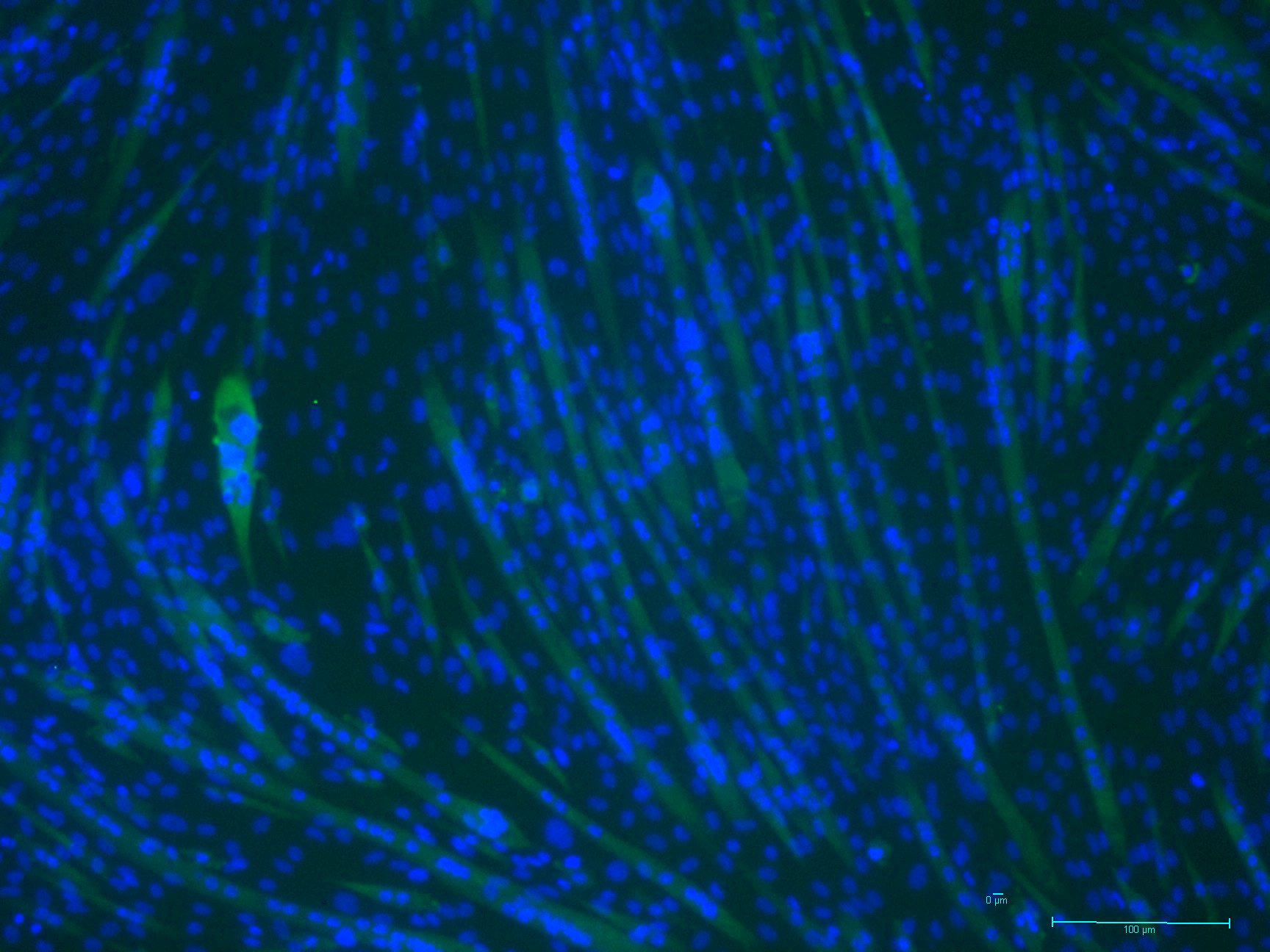

Supplement: Supplementary file 4 — Source data Fig. 2 [file 44321_2025_234_MOESM4_ESM.zip › Figure 2D/siCON+Dex/siCON+Dex4 merge.jpg]

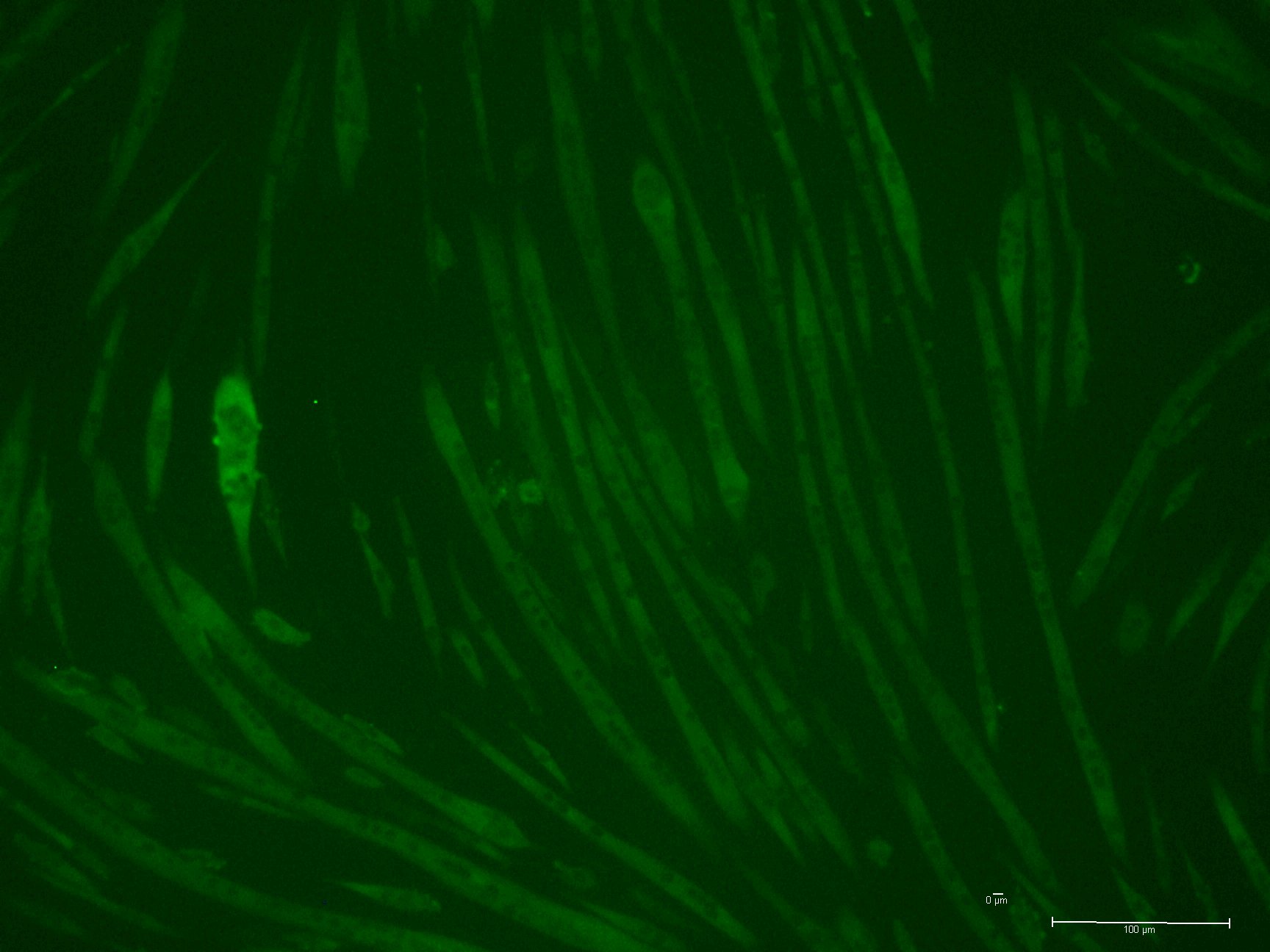

Supplement: Supplementary file 4 — Source data Fig. 2 [file 44321_2025_234_MOESM4_ESM.zip › Figure 2D/siCON+Dex/siCON+Dex4.jpg]

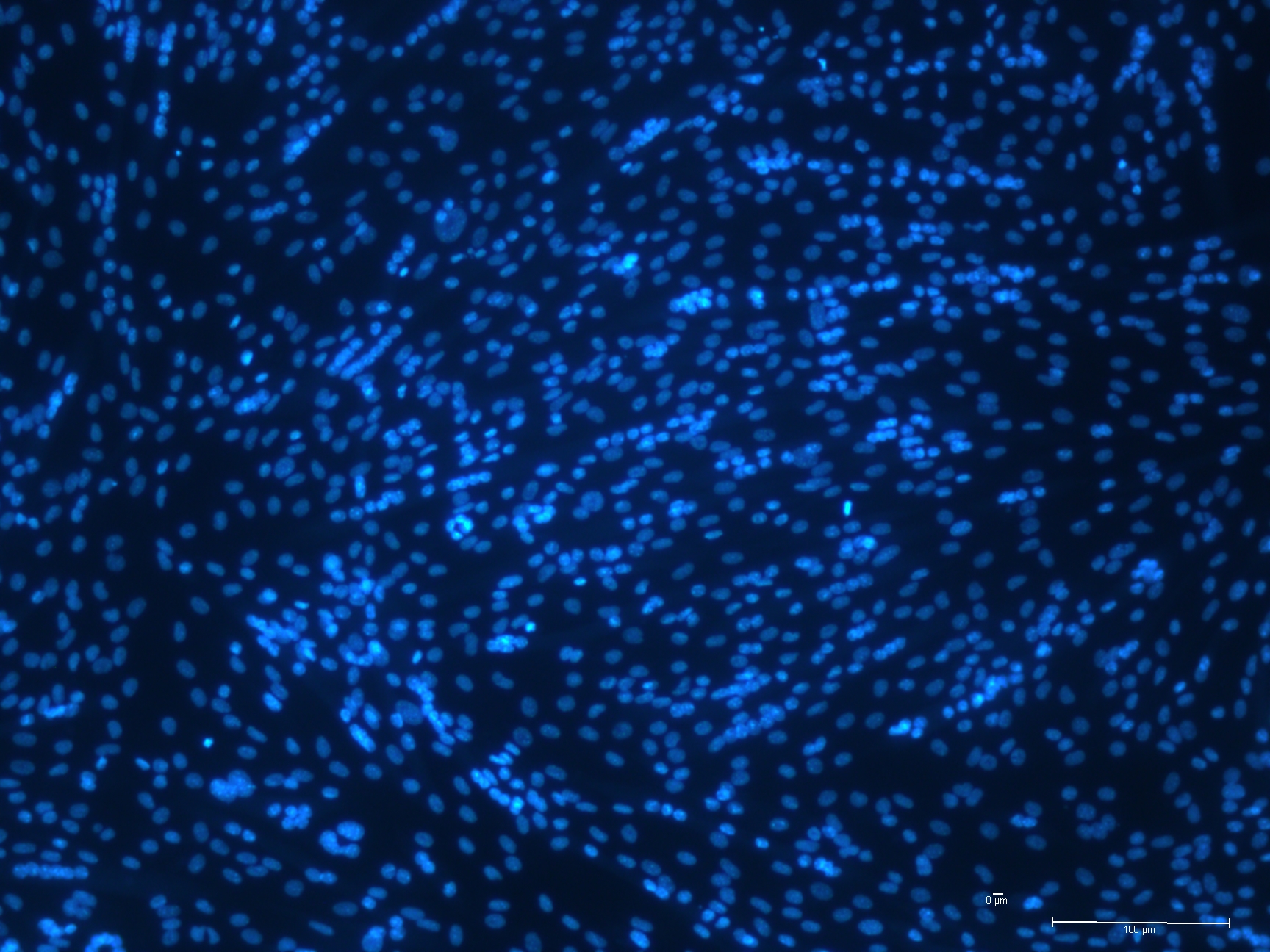

Supplement: Supplementary file 4 — Source data Fig. 2 [file 44321_2025_234_MOESM4_ESM.zip › Figure 2D/siDUSP22+Dex/siDUSP22+Dex1 DAPI.jpg]

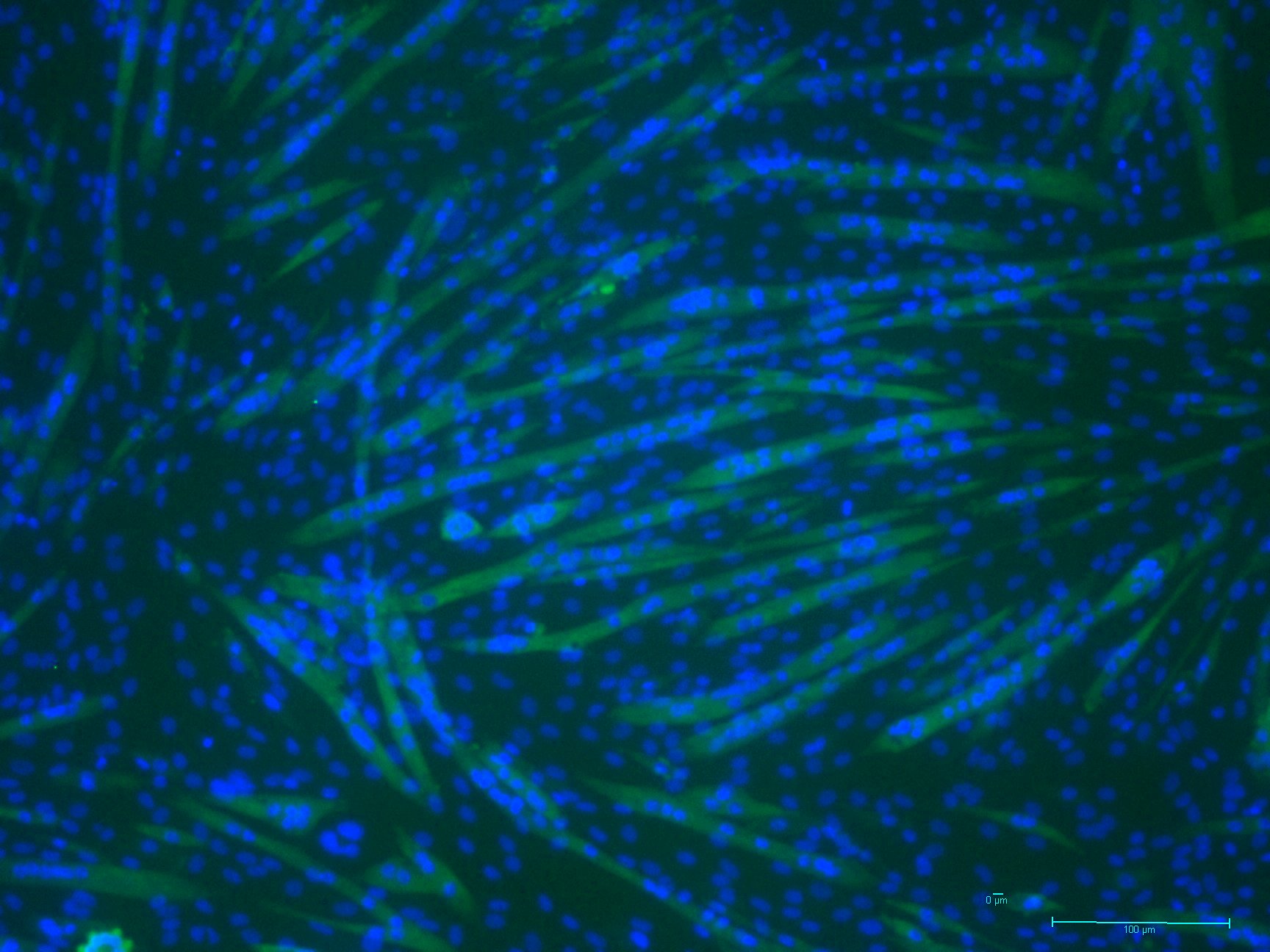

Supplement: Supplementary file 4 — Source data Fig. 2 [file 44321_2025_234_MOESM4_ESM.zip › Figure 2D/siDUSP22+Dex/siDUSP22+Dex1 merge.jpg]

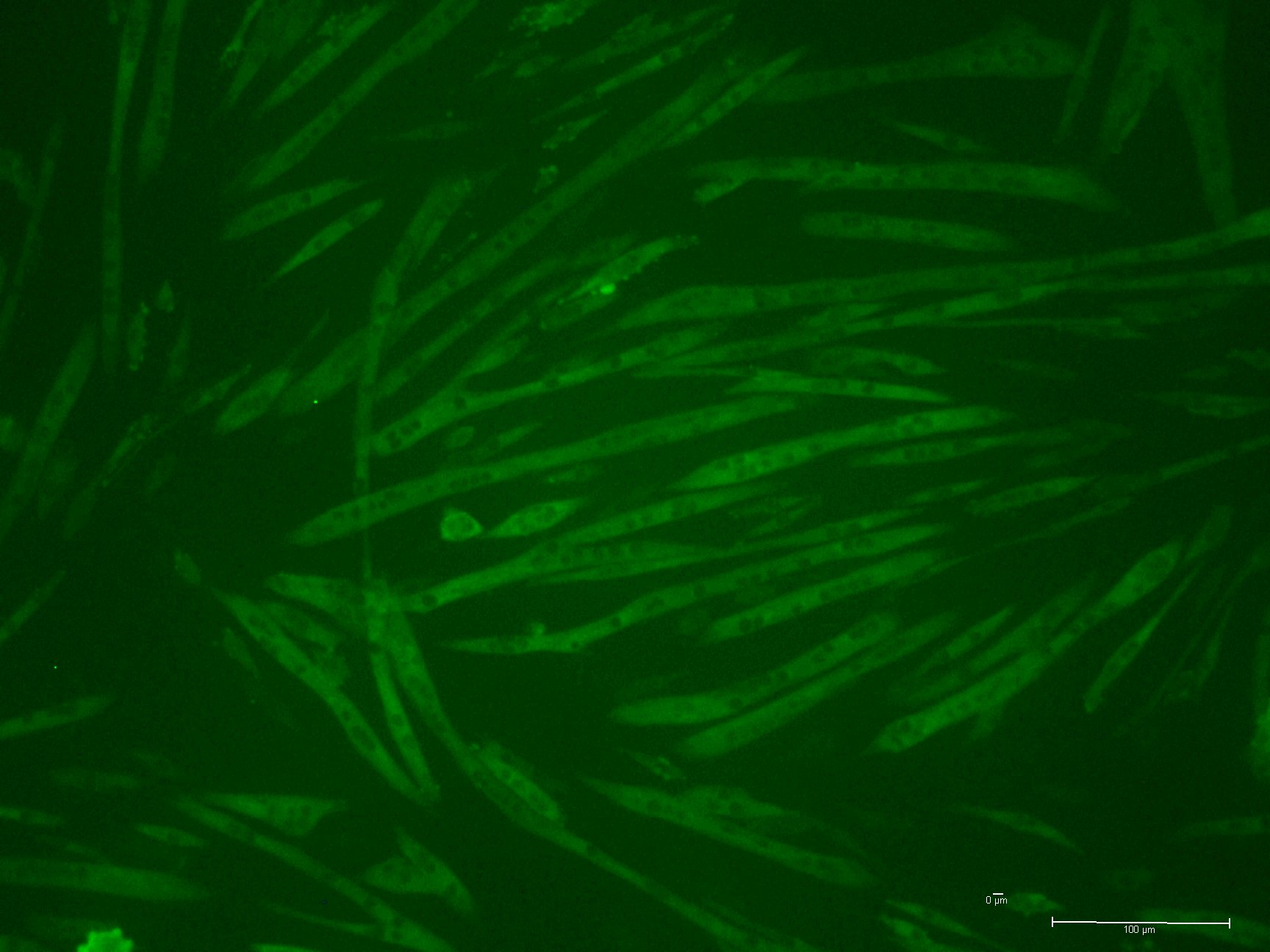

Supplement: Supplementary file 4 — Source data Fig. 2 [file 44321_2025_234_MOESM4_ESM.zip › Figure 2D/siDUSP22+Dex/siDUSP22+Dex1.jpg]

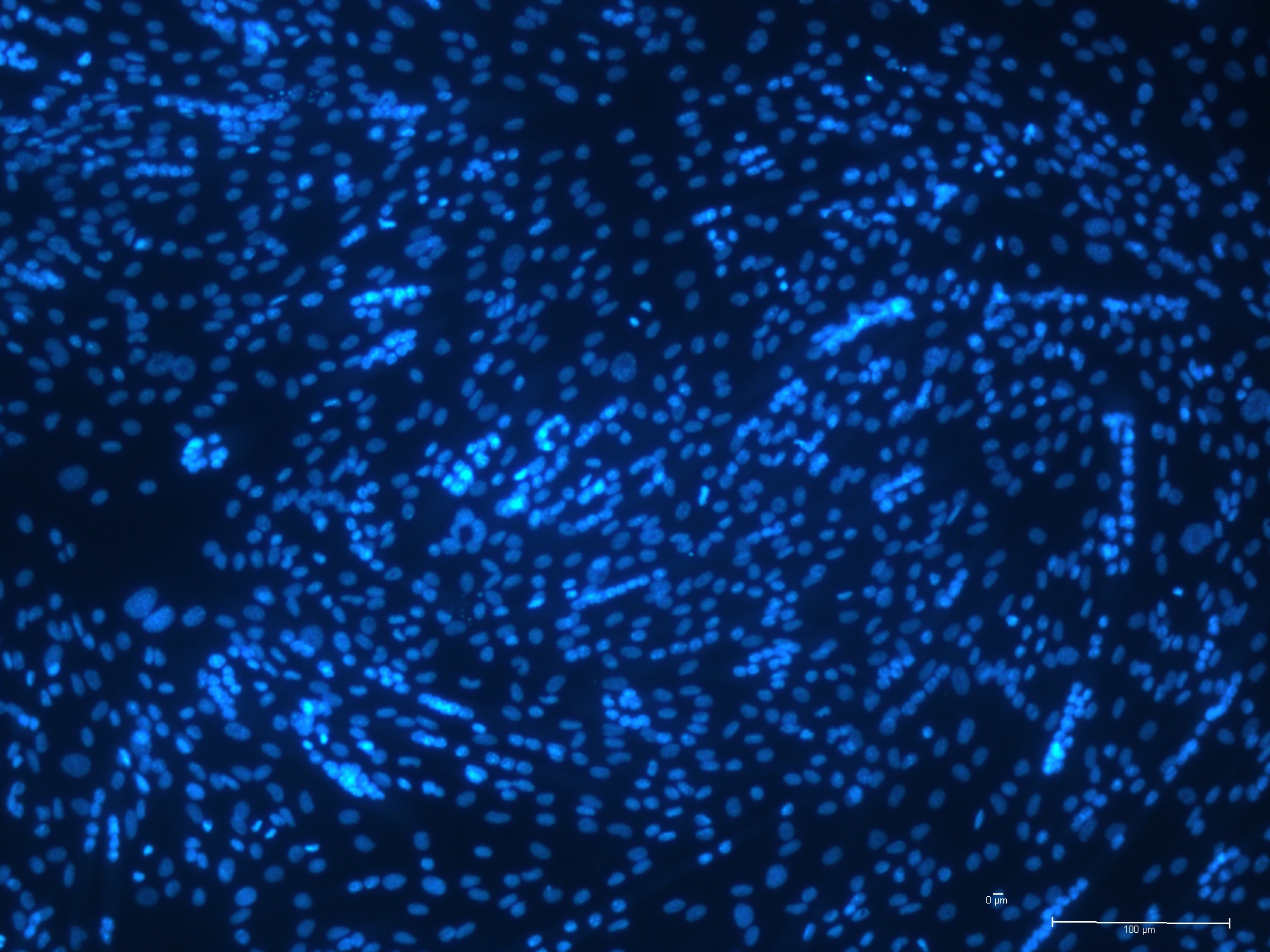

Supplement: Supplementary file 4 — Source data Fig. 2 [file 44321_2025_234_MOESM4_ESM.zip › Figure 2D/siDUSP22+Dex/siDUSP22+Dex2 DAPI.jpg]

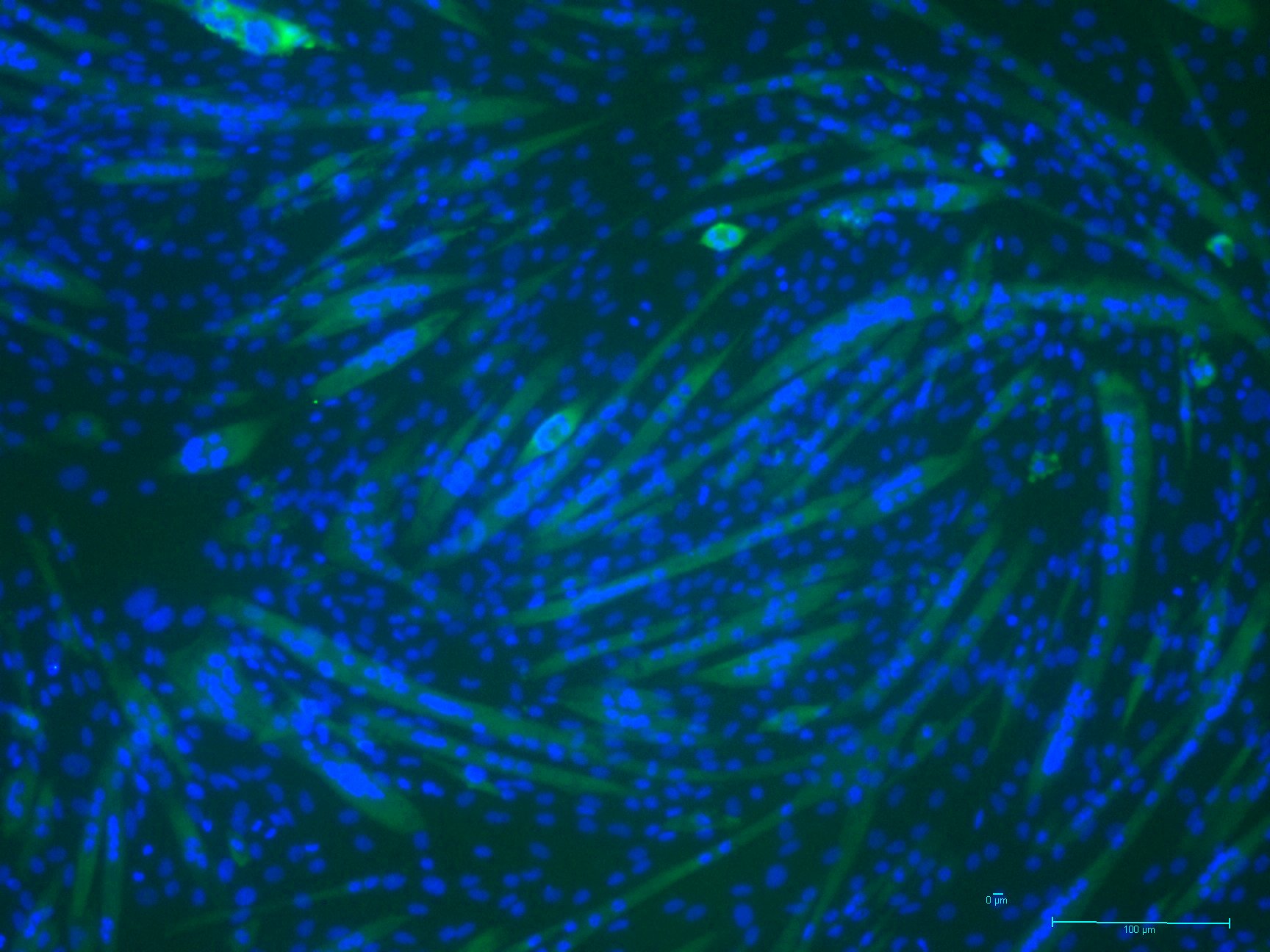

Supplement: Supplementary file 4 — Source data Fig. 2 [file 44321_2025_234_MOESM4_ESM.zip › Figure 2D/siDUSP22+Dex/siDUSP22+Dex2 merge.jpg]

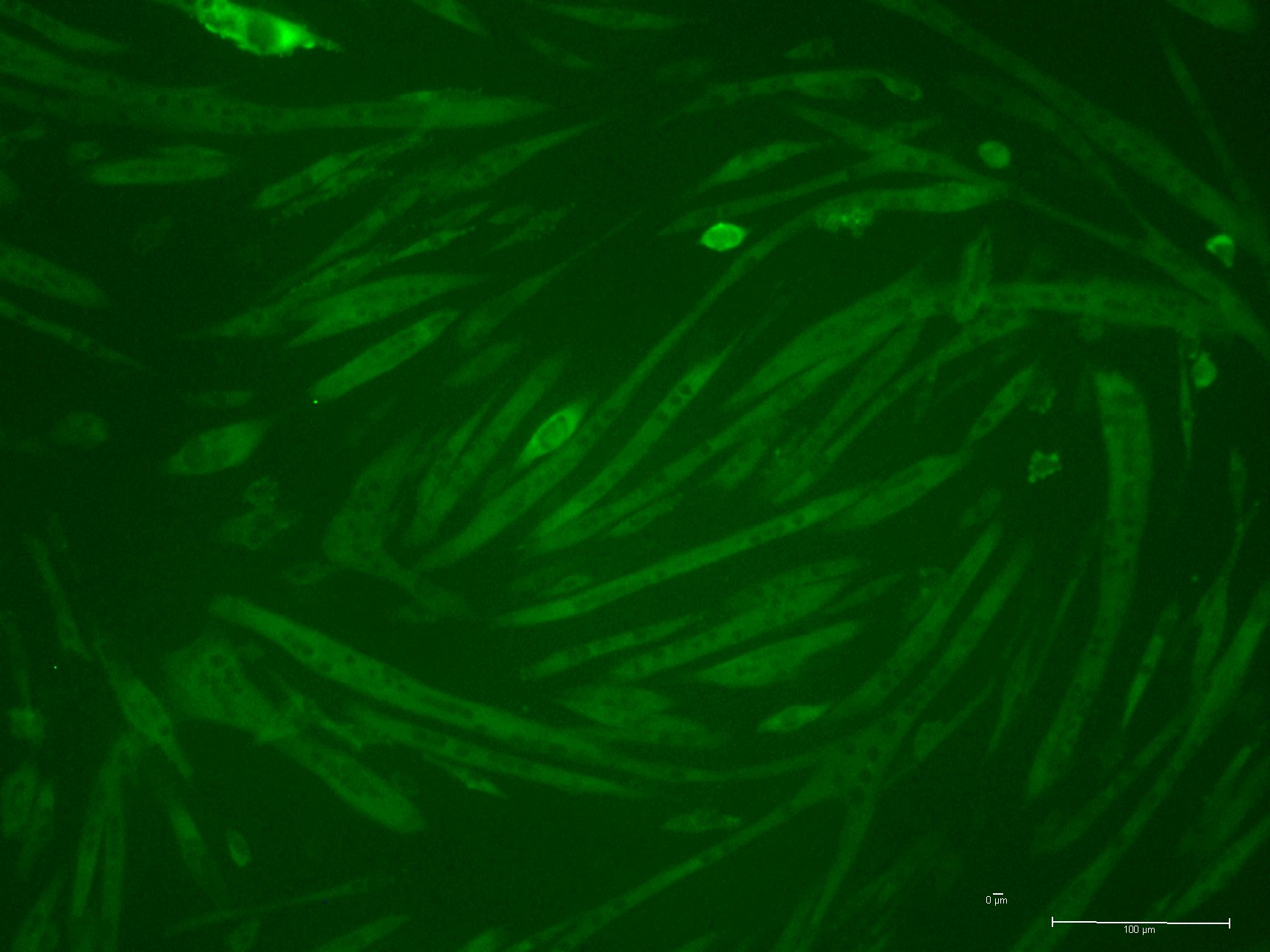

Supplement: Supplementary file 4 — Source data Fig. 2 [file 44321_2025_234_MOESM4_ESM.zip › Figure 2D/siDUSP22+Dex/siDUSP22+Dex2.jpg]

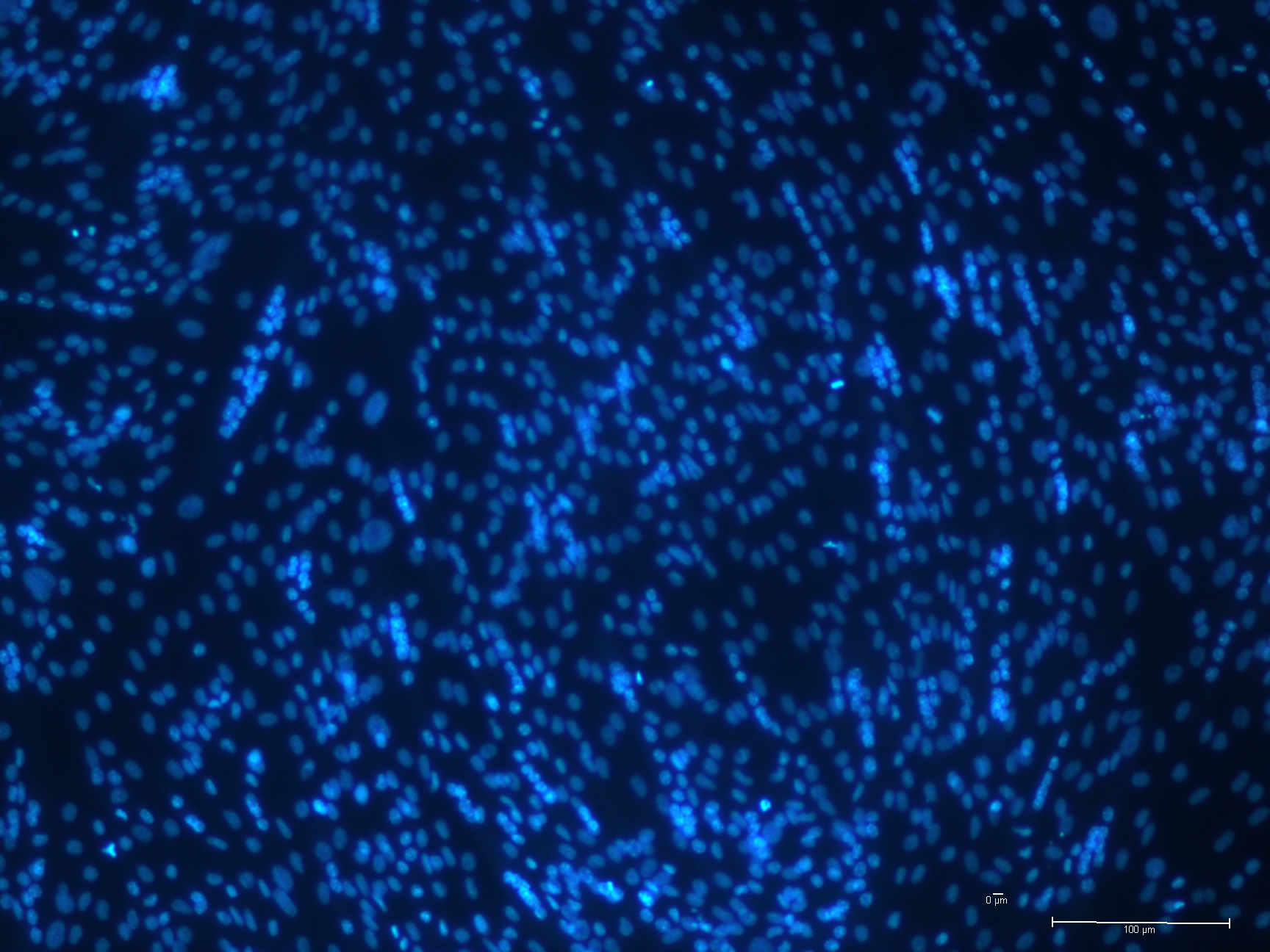

Supplement: Supplementary file 4 — Source data Fig. 2 [file 44321_2025_234_MOESM4_ESM.zip › Figure 2D/siDUSP22+Dex/siDUSP22+Dex3 DAPI.jpg]

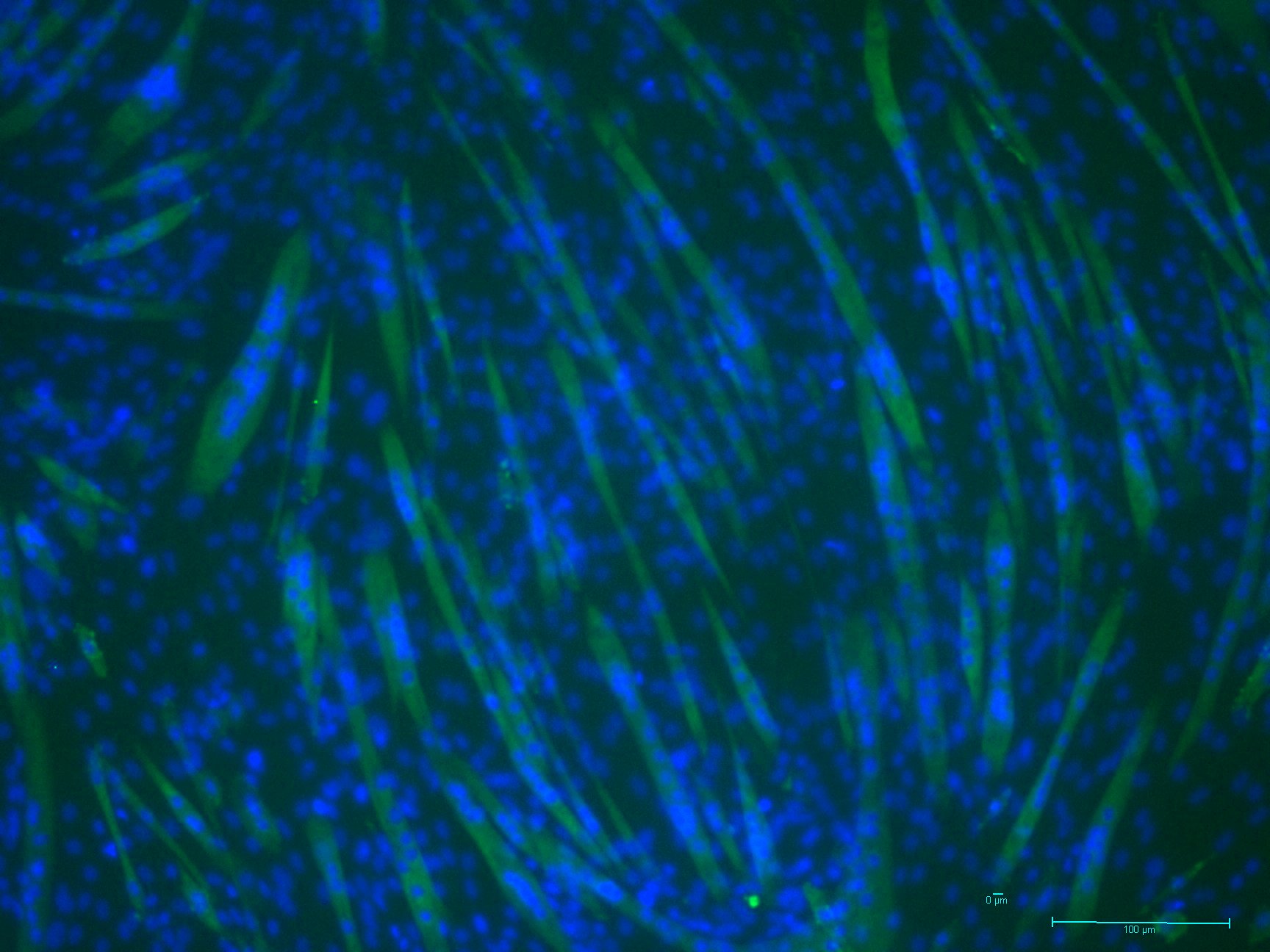

Supplement: Supplementary file 4 — Source data Fig. 2 [file 44321_2025_234_MOESM4_ESM.zip › Figure 2D/siDUSP22+Dex/siDUSP22+Dex3 merge.jpg]

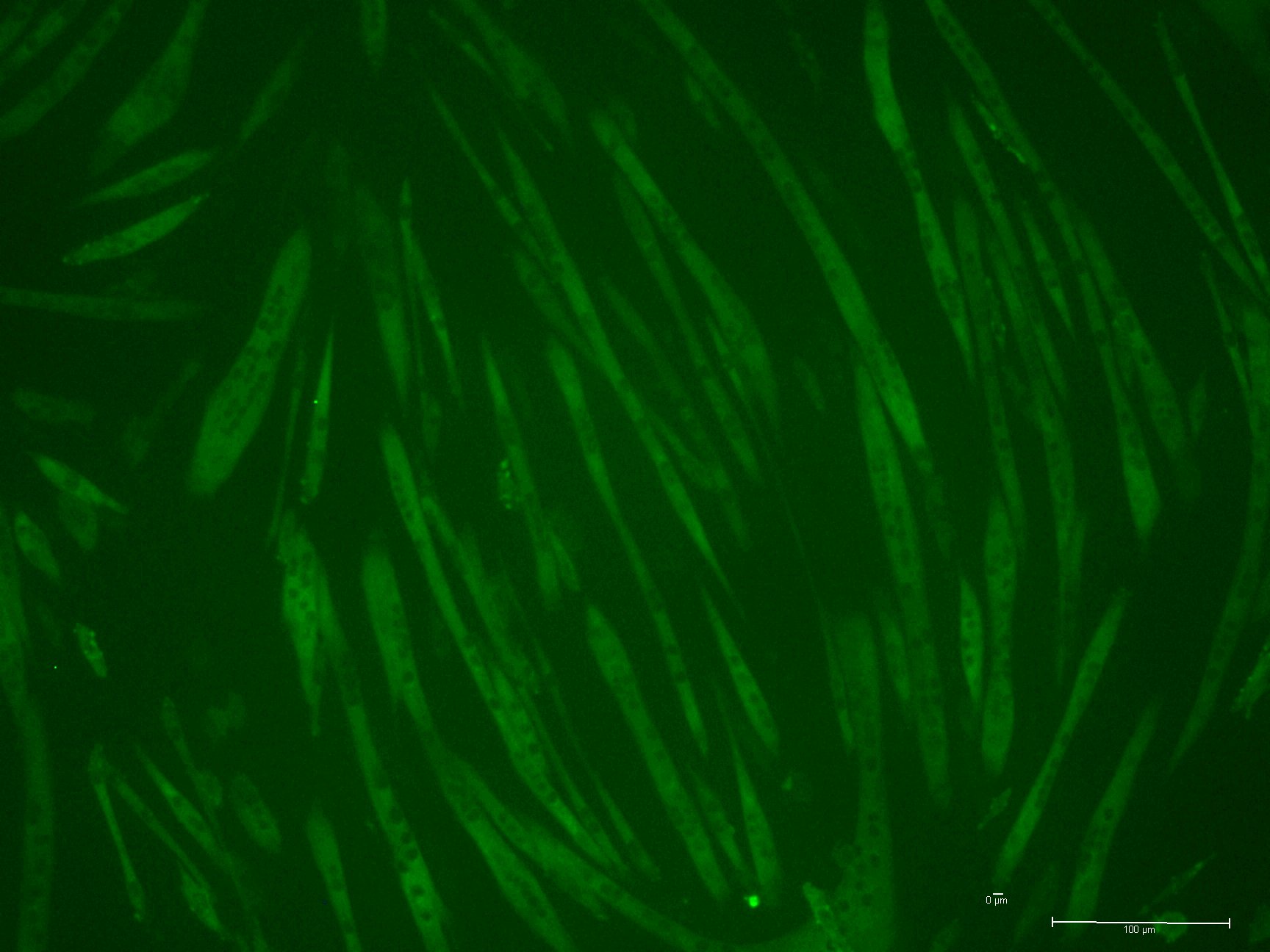

Supplement: Supplementary file 4 — Source data Fig. 2 [file 44321_2025_234_MOESM4_ESM.zip › Figure 2D/siDUSP22+Dex/siDUSP22+Dex3.jpg]

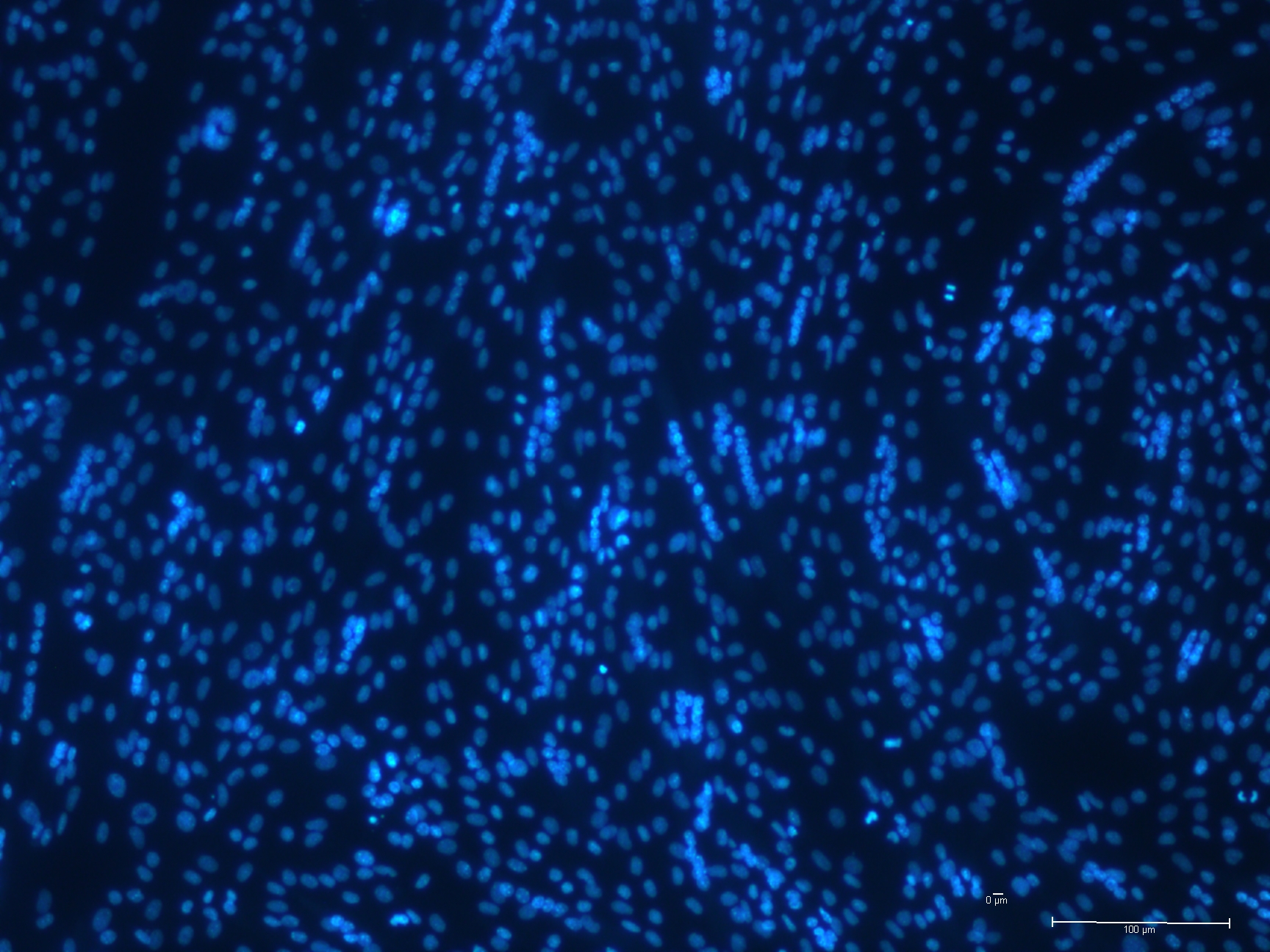

Supplement: Supplementary file 4 — Source data Fig. 2 [file 44321_2025_234_MOESM4_ESM.zip › Figure 2D/siDUSP22+Dex/siDUSP22+Dex4 DAPI.jpg]

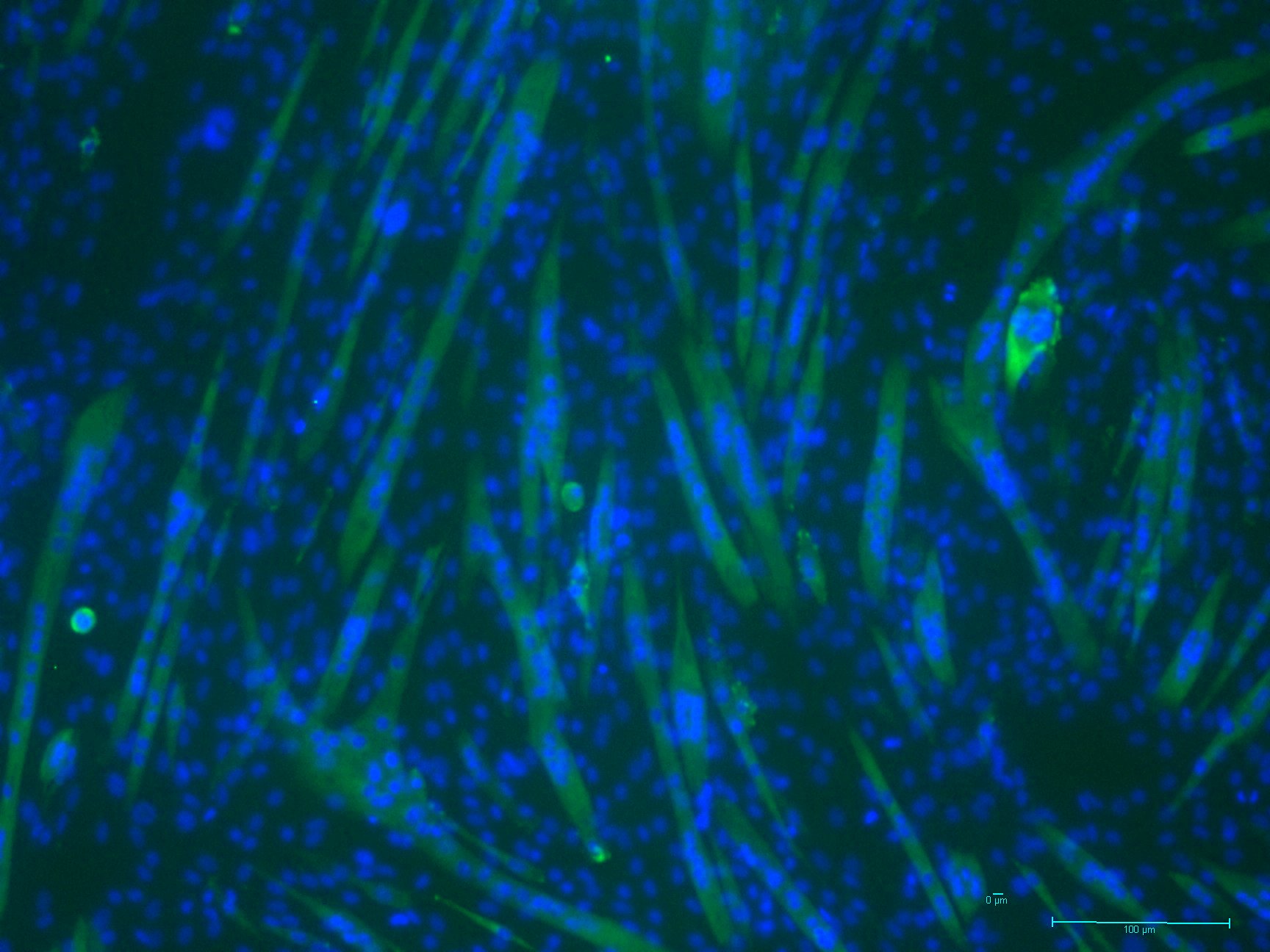

Supplement: Supplementary file 4 — Source data Fig. 2 [file 44321_2025_234_MOESM4_ESM.zip › Figure 2D/siDUSP22+Dex/siDUSP22+Dex4 merge.jpg]

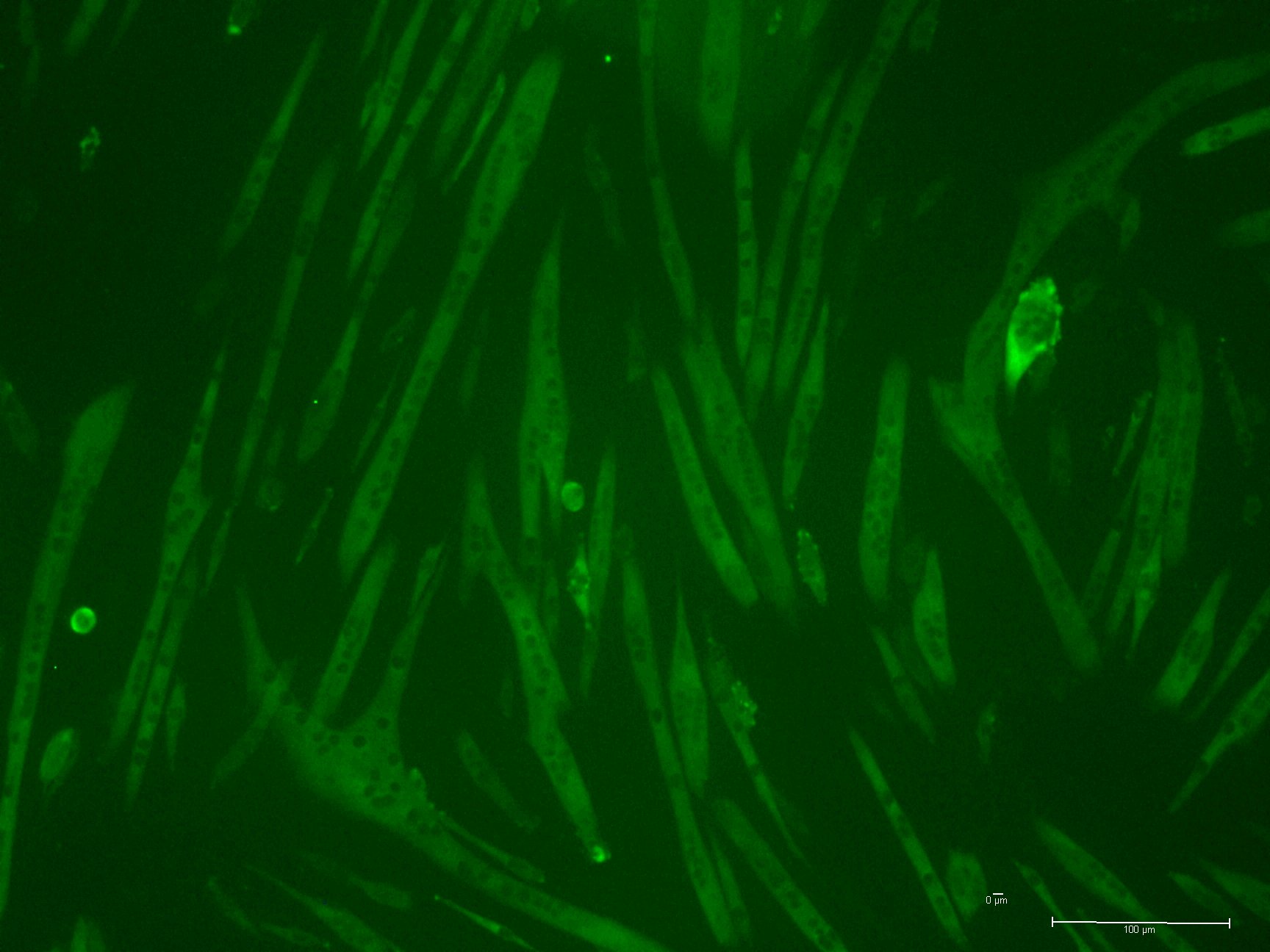

Supplement: Supplementary file 4 — Source data Fig. 2 [file 44321_2025_234_MOESM4_ESM.zip › Figure 2D/siDUSP22+Dex/siDUSP22+Dex4.jpg]

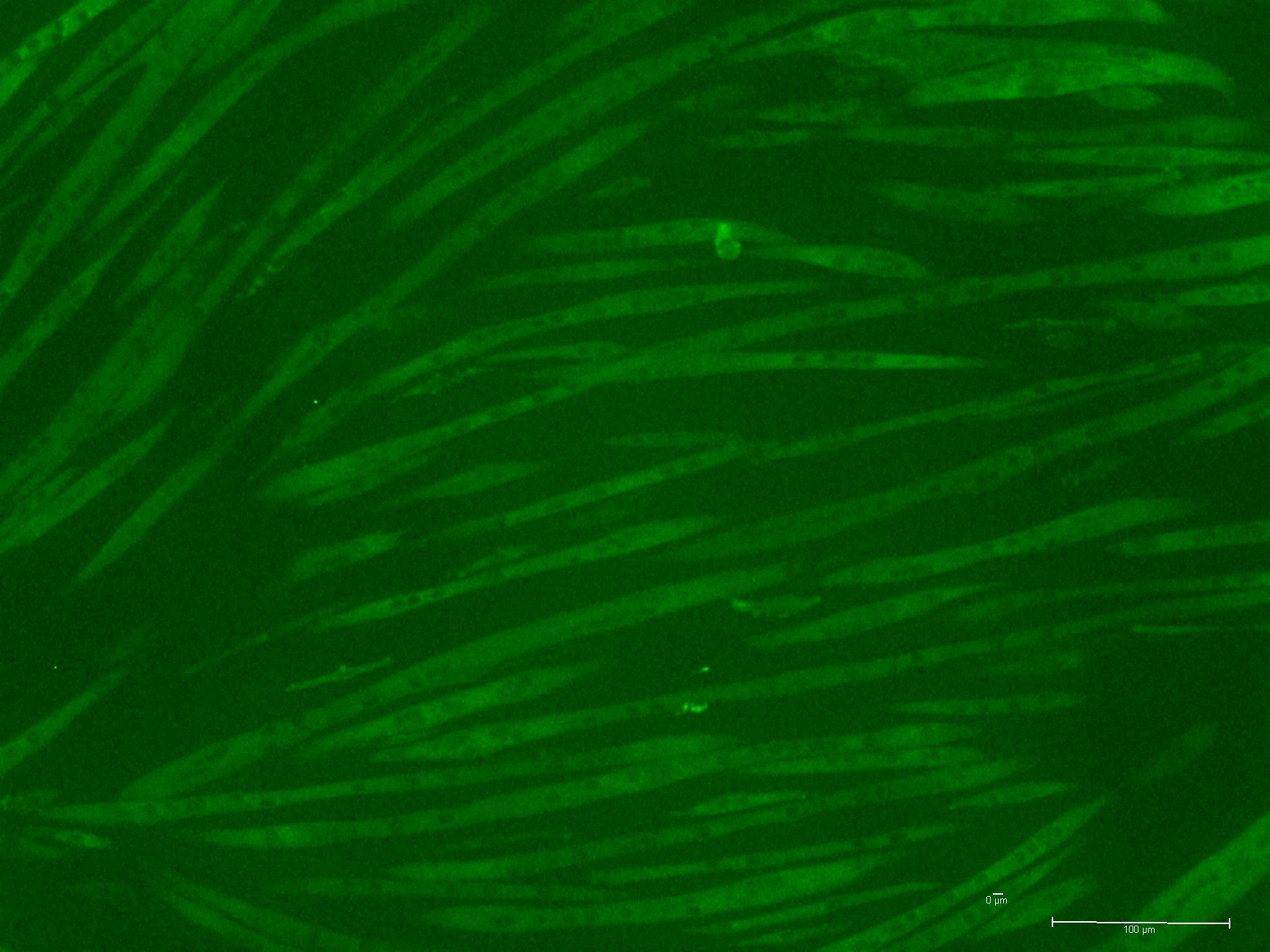

Supplement: Supplementary file 4 — Source data Fig. 2 [file 44321_2025_234_MOESM4_ESM.zip › Figure 2D/untreated/untreated1.jpg]

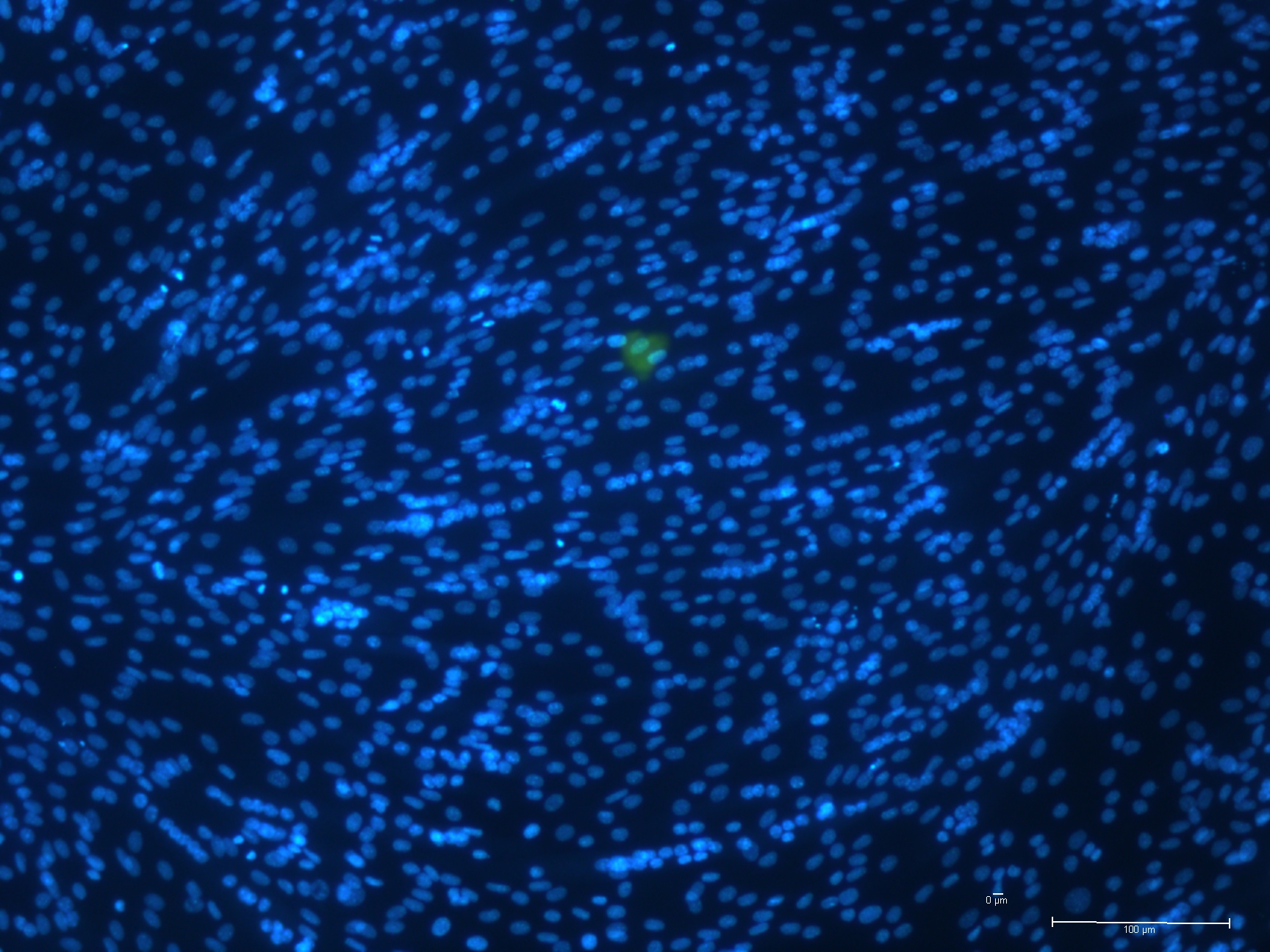

Supplement: Supplementary file 4 — Source data Fig. 2 [file 44321_2025_234_MOESM4_ESM.zip › Figure 2D/untreated/untreated2 DAPI.jpg]

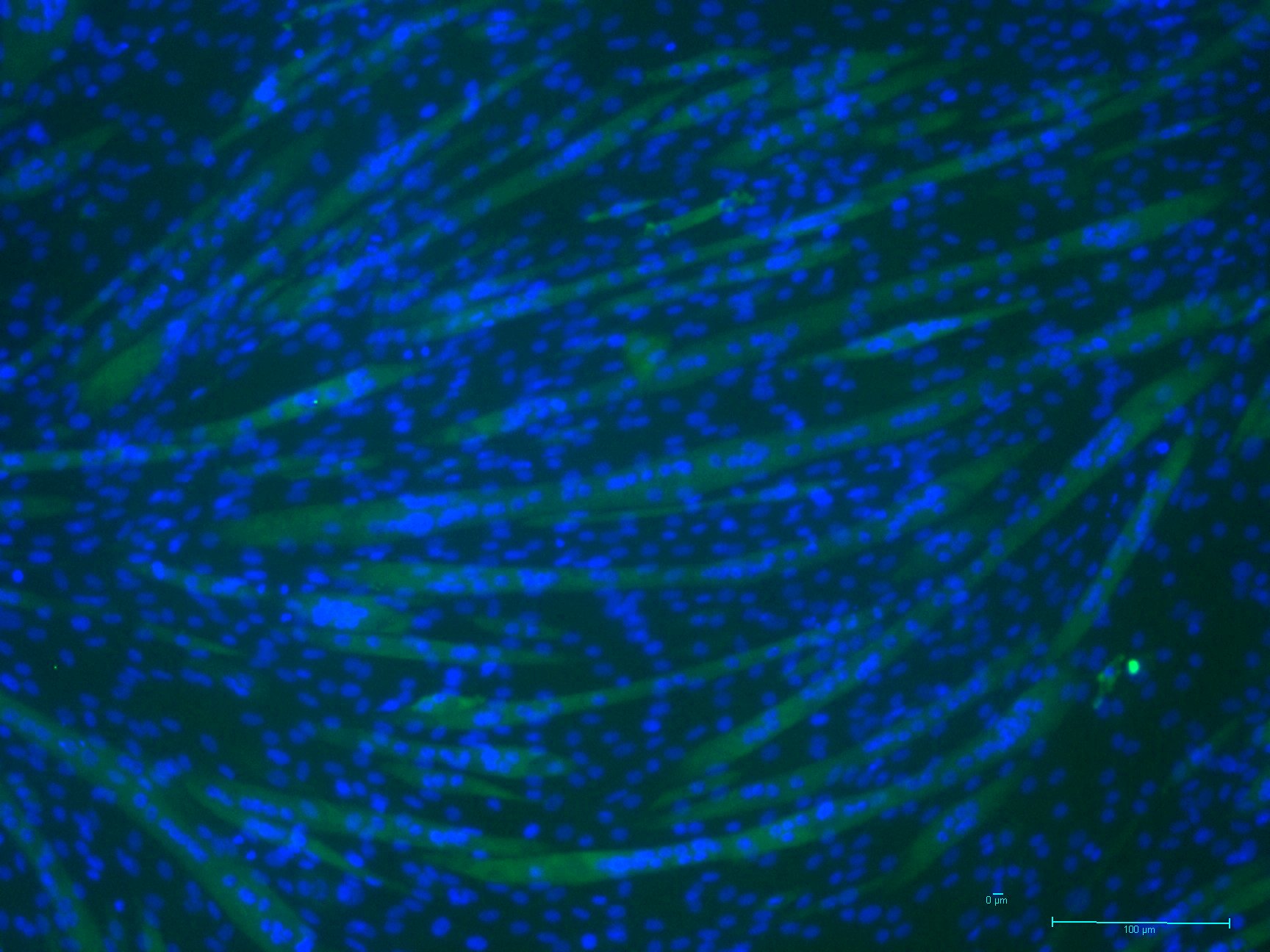

Supplement: Supplementary file 4 — Source data Fig. 2 [file 44321_2025_234_MOESM4_ESM.zip › Figure 2D/untreated/untreated2 merge.jpg]

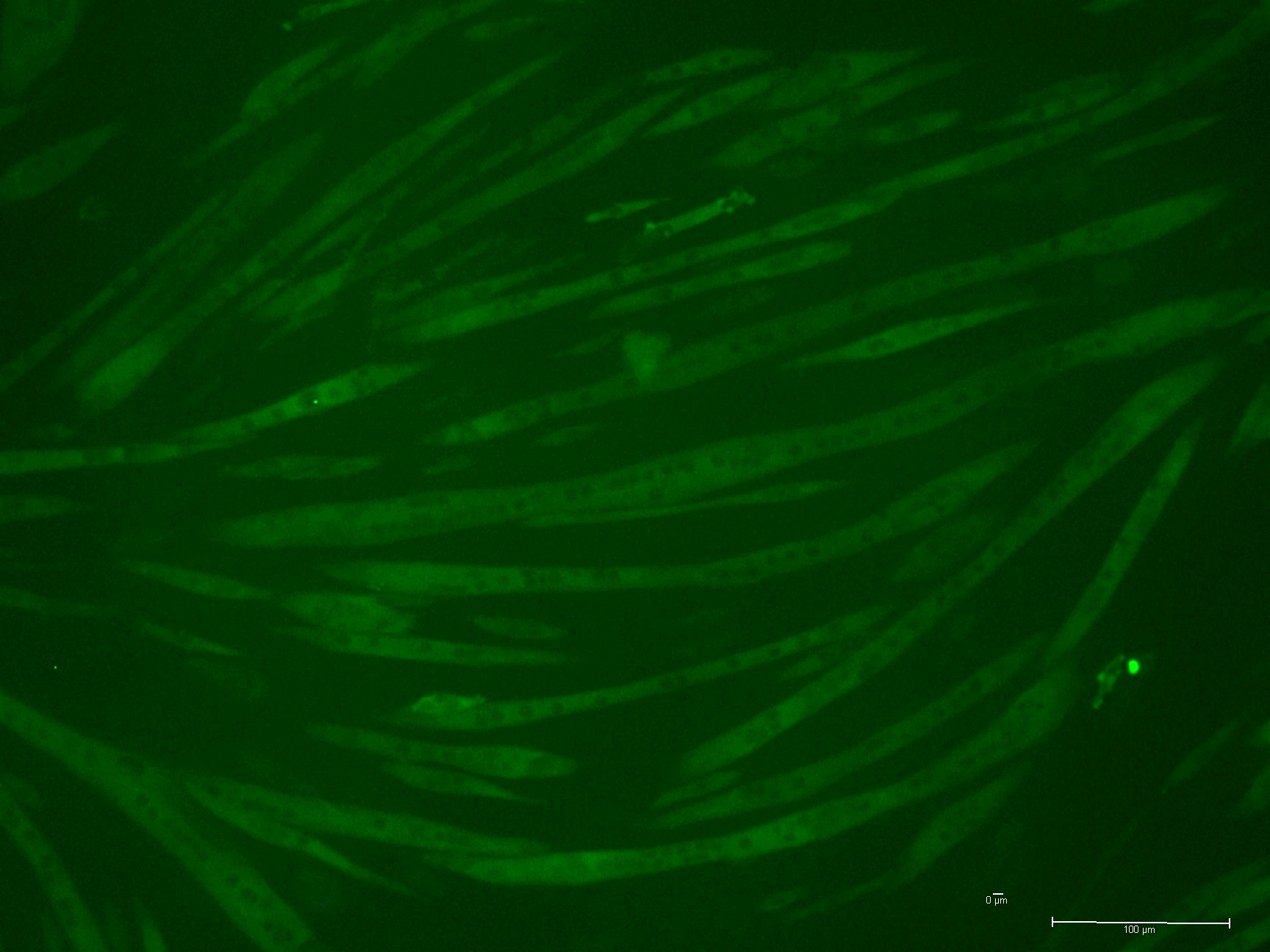

Supplement: Supplementary file 4 — Source data Fig. 2 [file 44321_2025_234_MOESM4_ESM.zip › Figure 2D/untreated/untreated2.jpg]

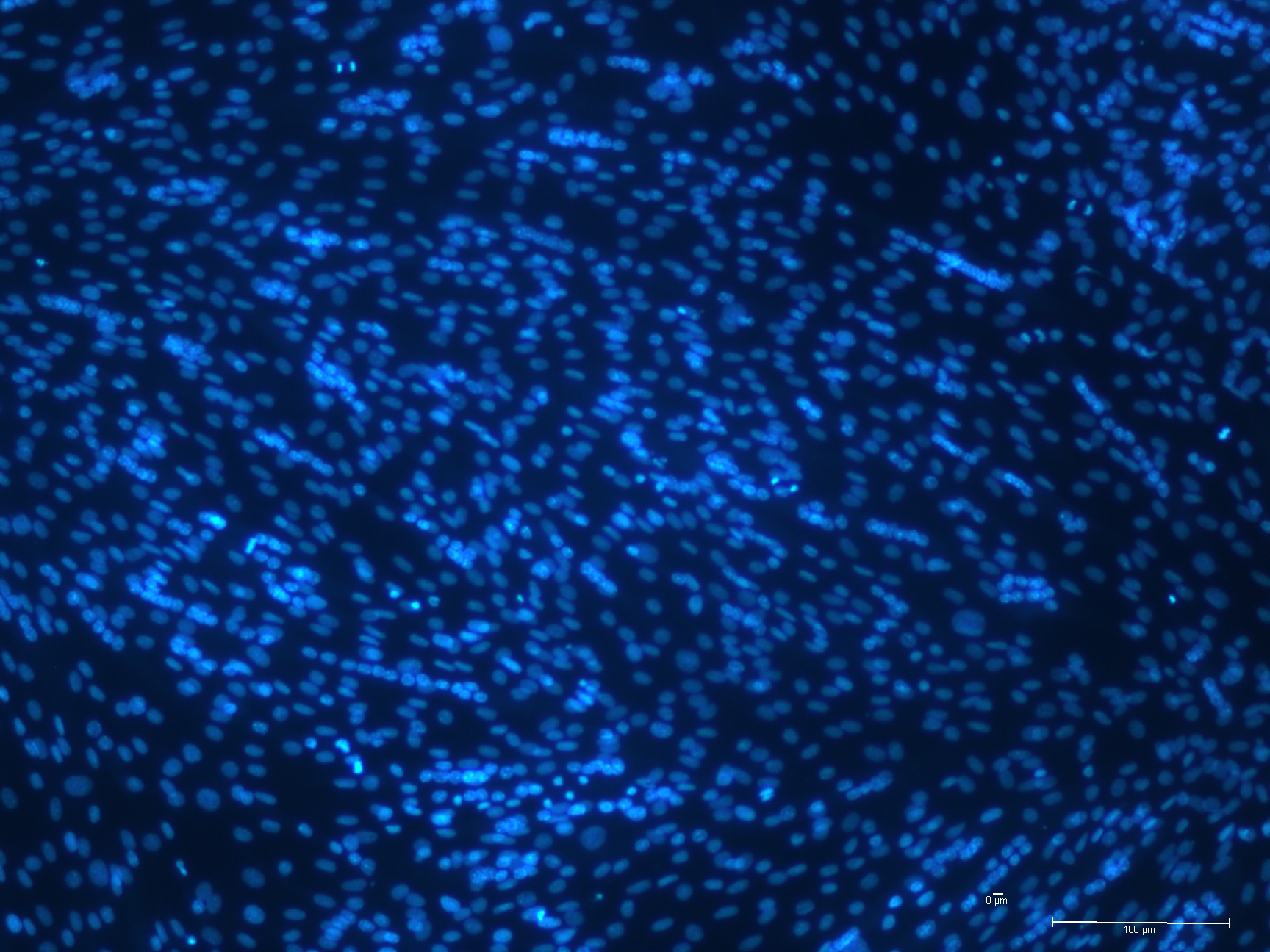

Supplement: Supplementary file 4 — Source data Fig. 2 [file 44321_2025_234_MOESM4_ESM.zip › Figure 2D/untreated/untreated3 DAPI.jpg]

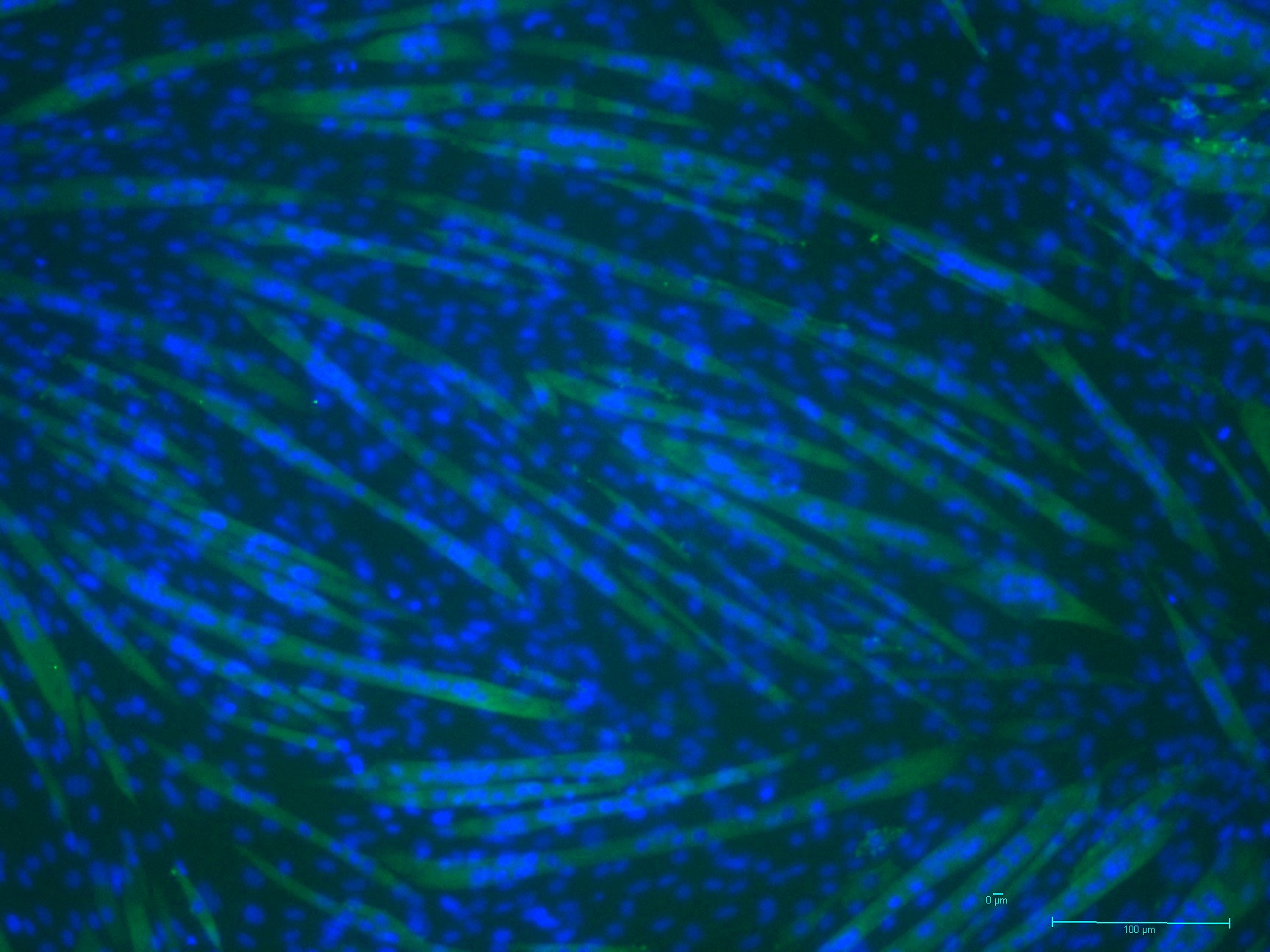

Supplement: Supplementary file 4 — Source data Fig. 2 [file 44321_2025_234_MOESM4_ESM.zip › Figure 2D/untreated/untreated3 merge.jpg]

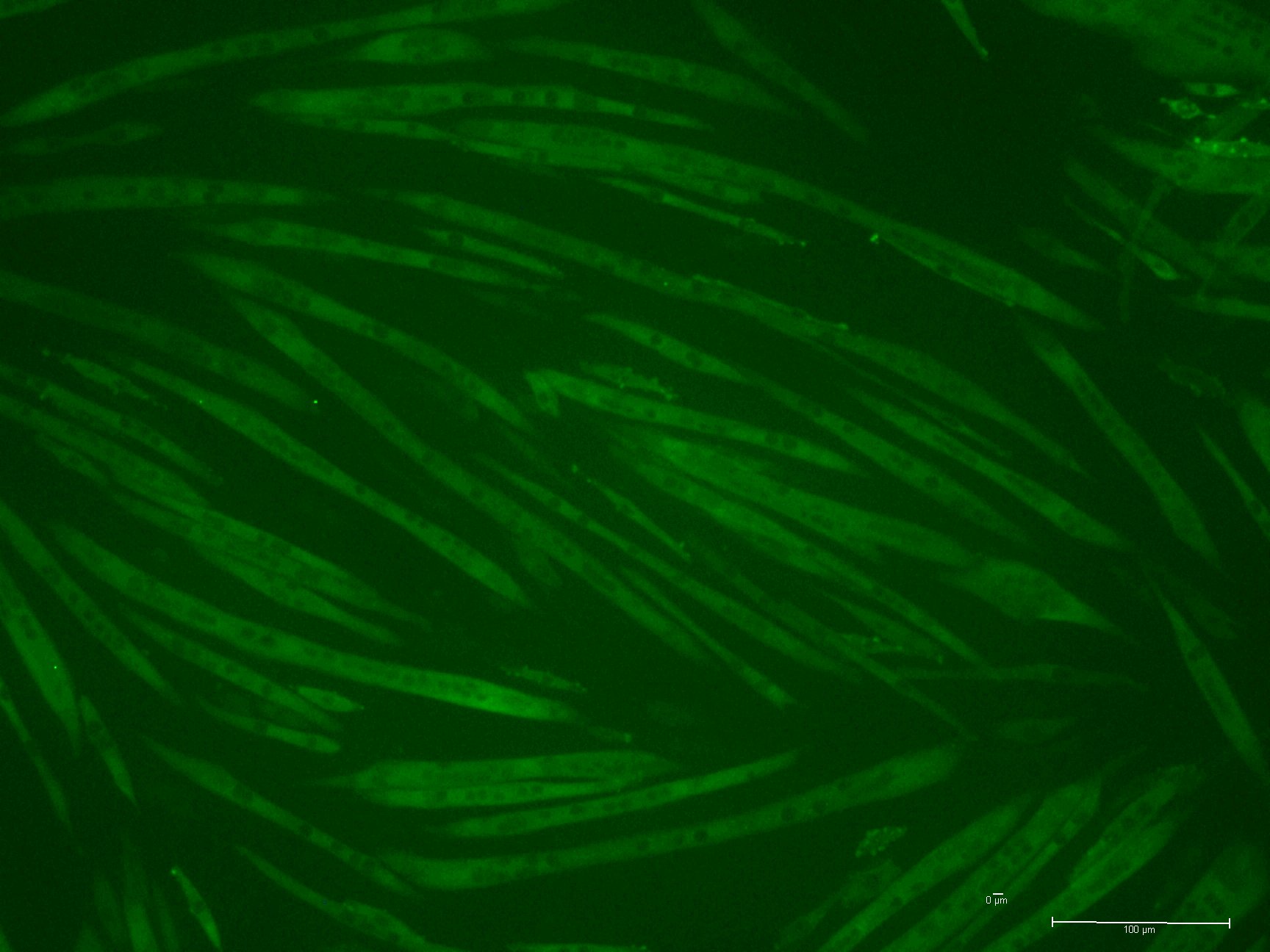

Supplement: Supplementary file 4 — Source data Fig. 2 [file 44321_2025_234_MOESM4_ESM.zip › Figure 2D/untreated/untreated3.jpg]

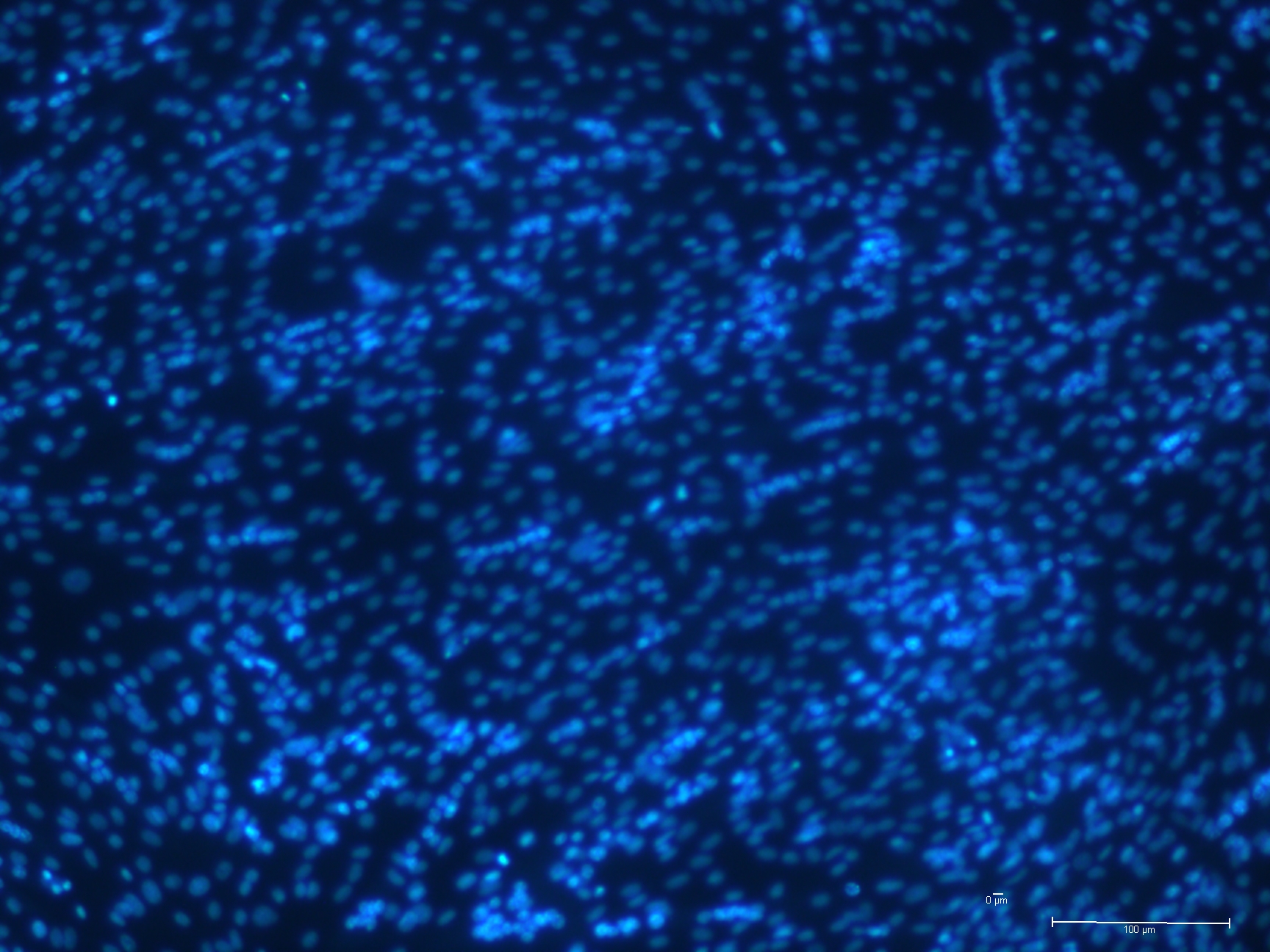

Supplement: Supplementary file 4 — Source data Fig. 2 [file 44321_2025_234_MOESM4_ESM.zip › Figure 2D/untreated/untreated4 DAPI.jpg]

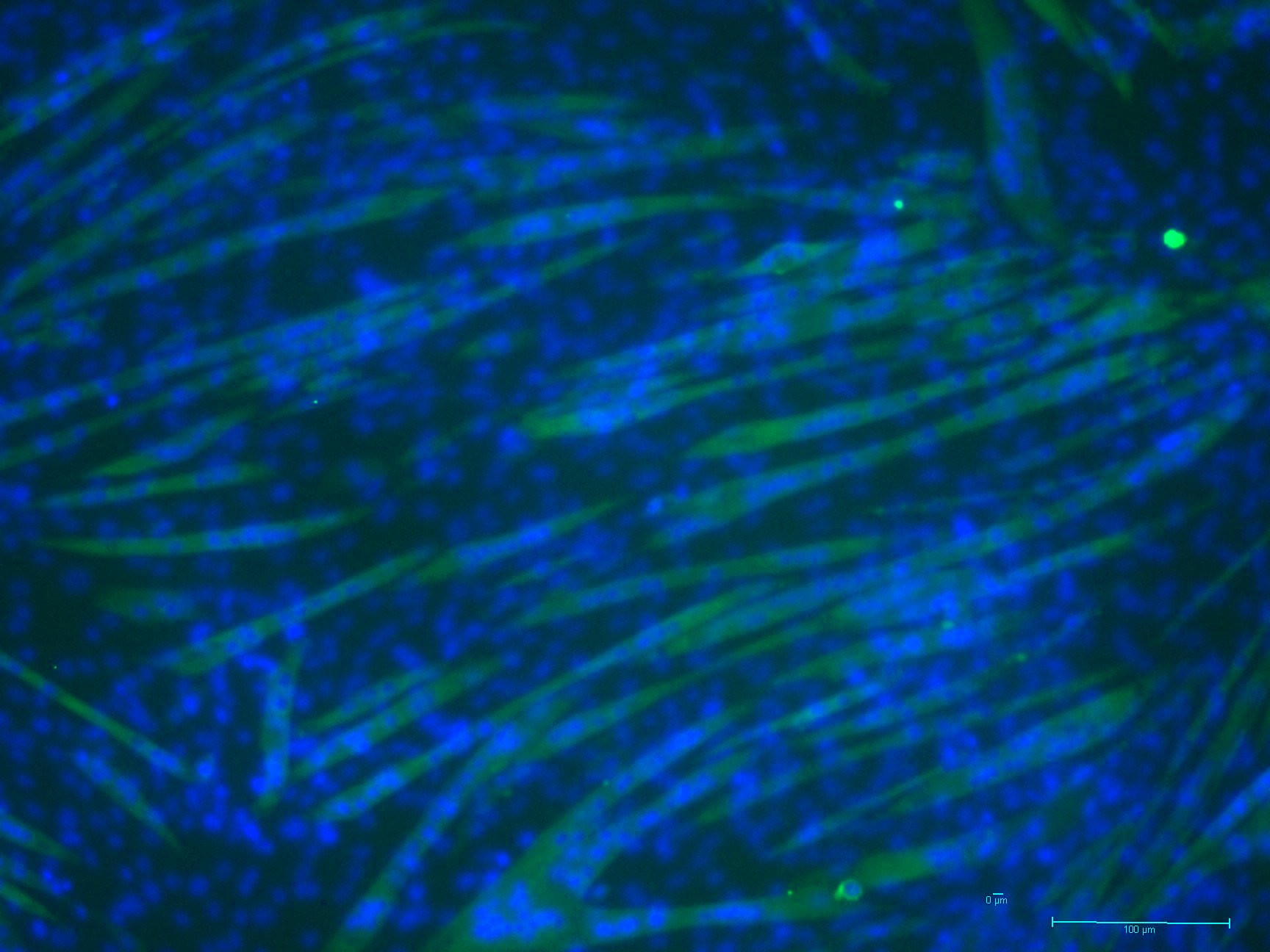

Supplement: Supplementary file 4 — Source data Fig. 2 [file 44321_2025_234_MOESM4_ESM.zip › Figure 2D/untreated/untreated4 merge.jpg]

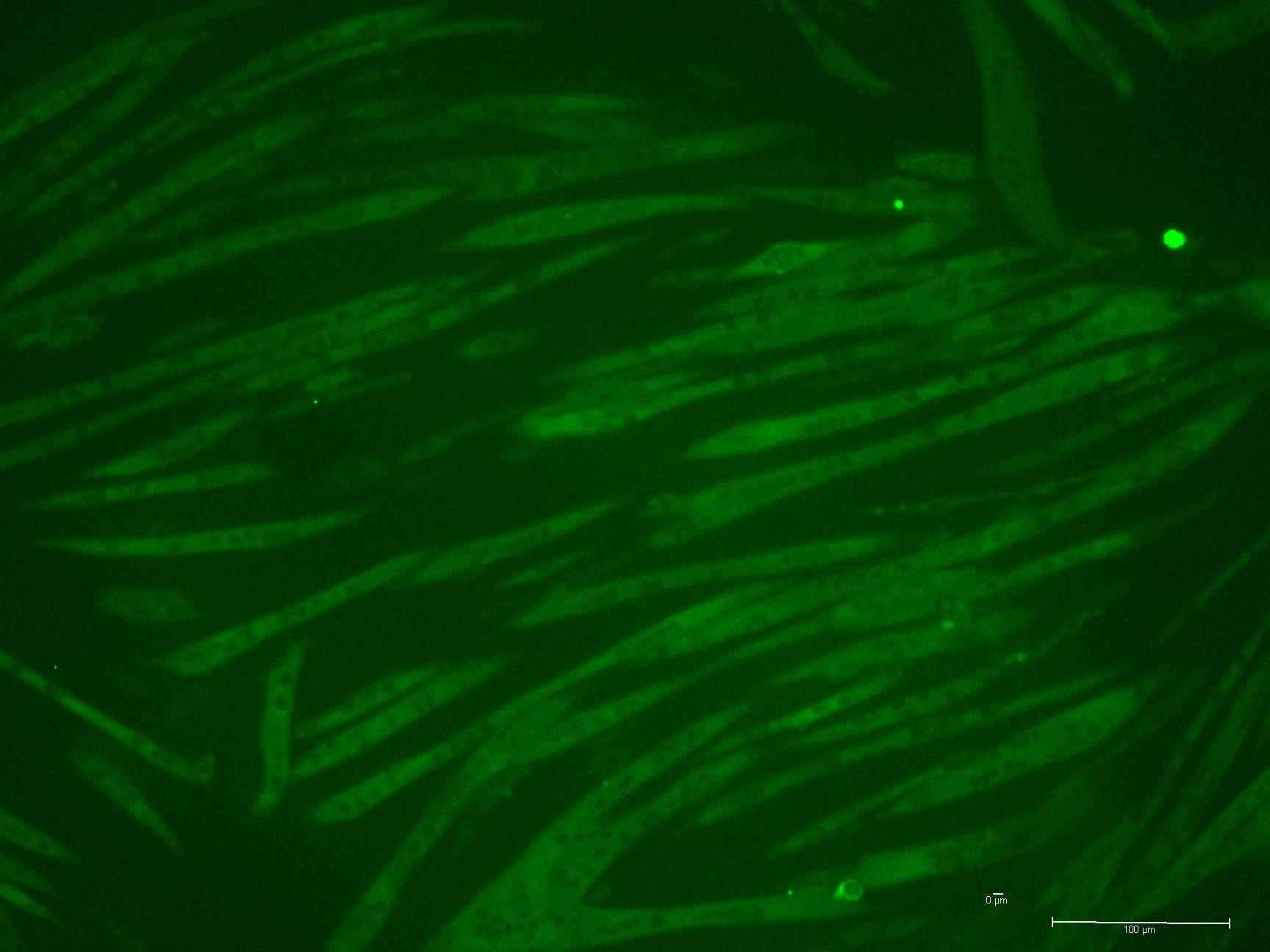

Supplement: Supplementary file 4 — Source data Fig. 2 [file 44321_2025_234_MOESM4_ESM.zip › Figure 2D/untreated/untreated4.jpg]

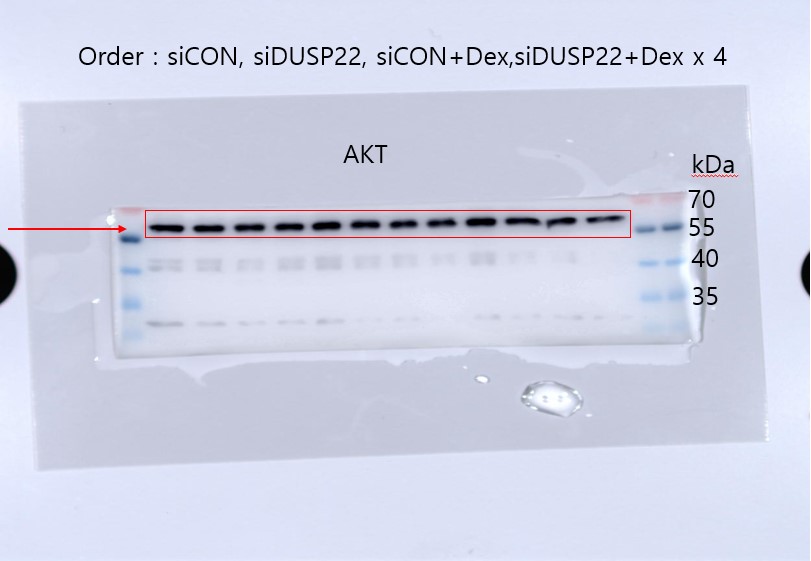

Supplement: Supplementary file 5 — Source data Fig. 3 [file 44321_2025_234_MOESM5_ESM.zip › Figure 3A/Figure 3A AKT 3.jpg]

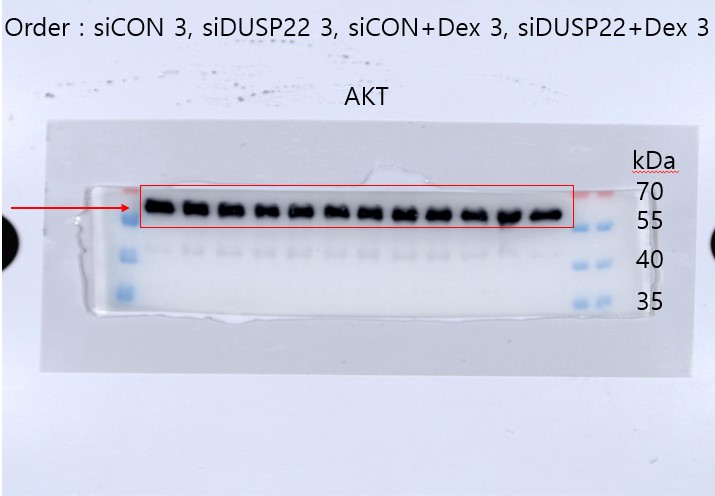

Supplement: Supplementary file 5 — Source data Fig. 3 [file 44321_2025_234_MOESM5_ESM.zip › Figure 3A/Figure 3A AKT.jpg]

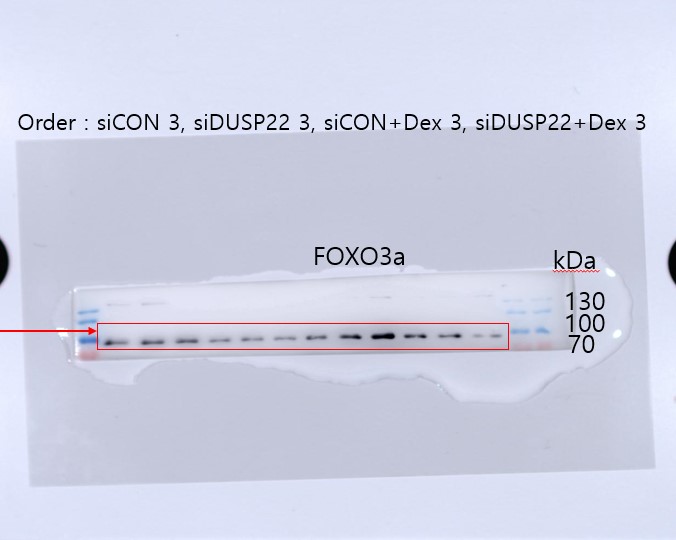

Supplement: Supplementary file 5 — Source data Fig. 3 [file 44321_2025_234_MOESM5_ESM.zip › Figure 3A/Figure 3A FOXO3a 2.jpg]

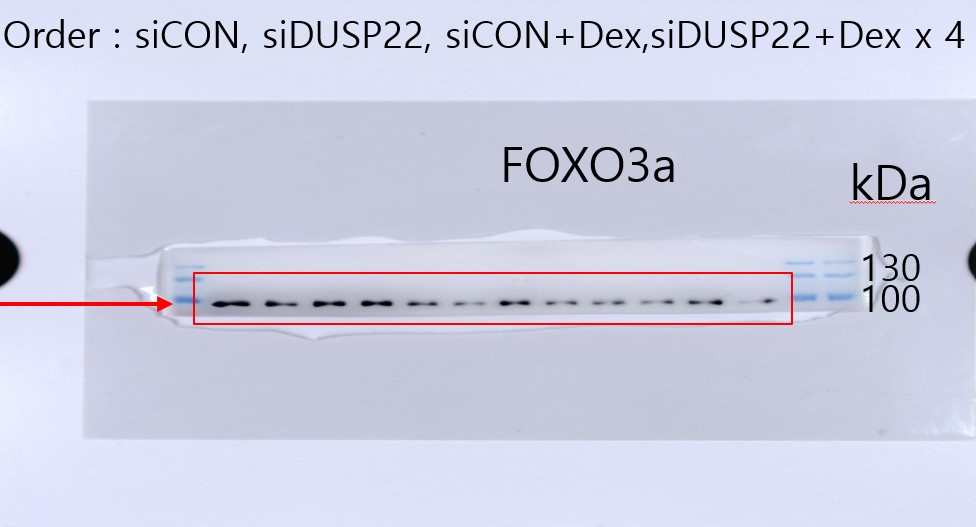

Supplement: Supplementary file 5 — Source data Fig. 3 [file 44321_2025_234_MOESM5_ESM.zip › Figure 3A/Figure 3A FOXO3a 3.jpg]

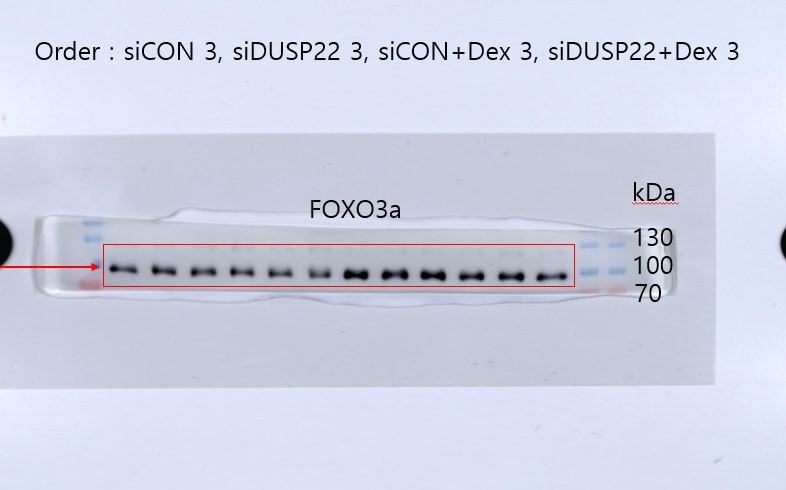

Supplement: Supplementary file 5 — Source data Fig. 3 [file 44321_2025_234_MOESM5_ESM.zip › Figure 3A/Figure 3A FOXO3a.jpg]

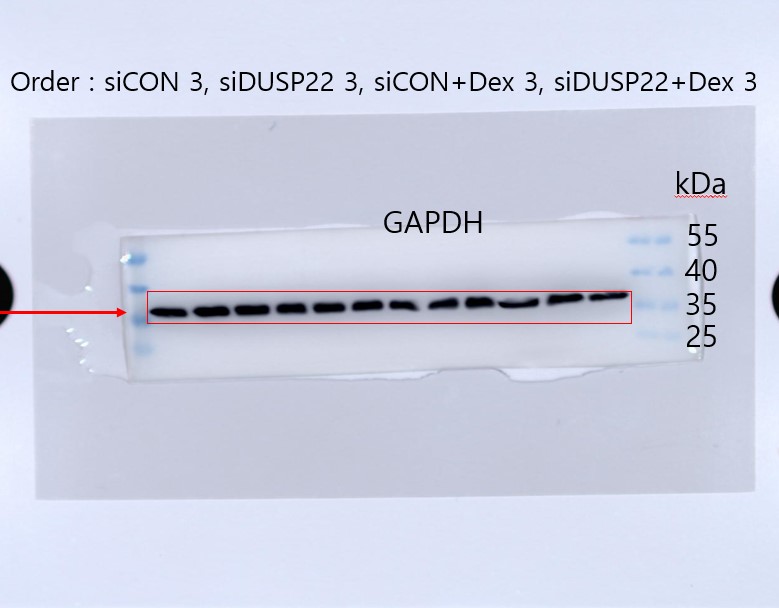

Supplement: Supplementary file 5 — Source data Fig. 3 [file 44321_2025_234_MOESM5_ESM.zip › Figure 3A/Figure 3A GAPDH 2.jpg]

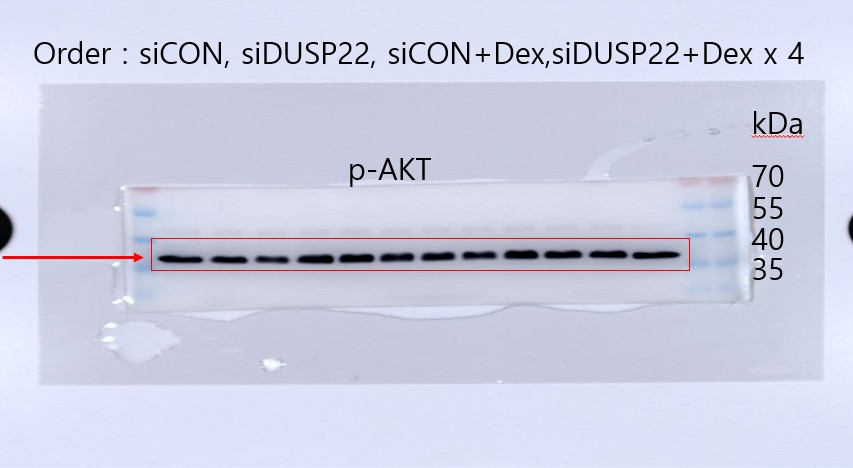

Supplement: Supplementary file 5 — Source data Fig. 3 [file 44321_2025_234_MOESM5_ESM.zip › Figure 3A/Figure 3A GAPDH 3.jpg]

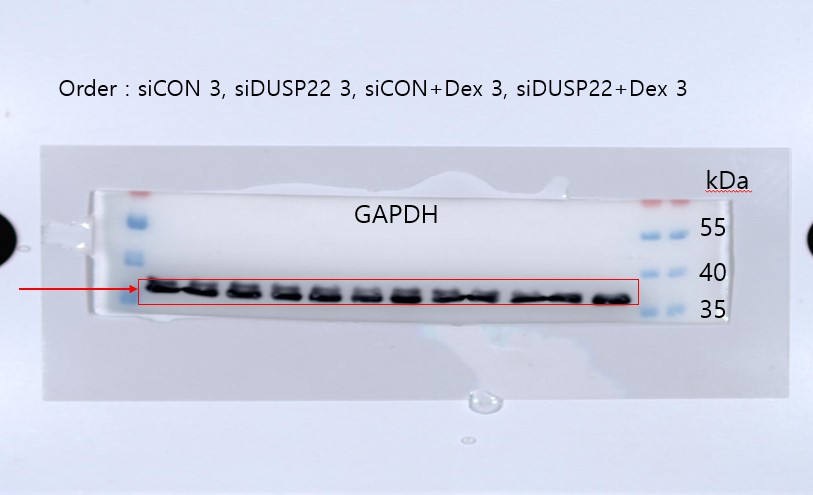

Supplement: Supplementary file 5 — Source data Fig. 3 [file 44321_2025_234_MOESM5_ESM.zip › Figure 3A/Figure 3A GAPDH.jpg]

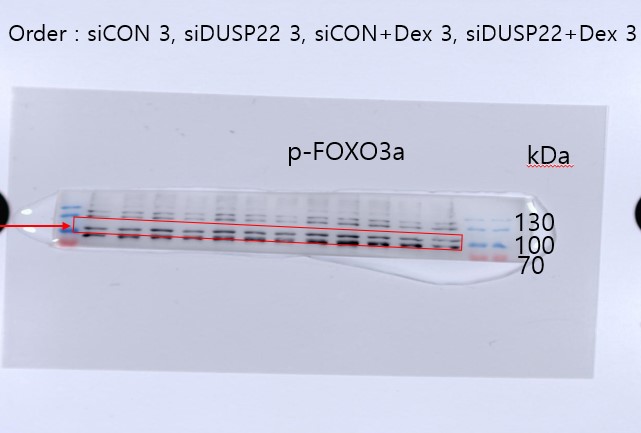

Supplement: Supplementary file 5 — Source data Fig. 3 [file 44321_2025_234_MOESM5_ESM.zip › Figure 3A/Figure 3A p-FOXO3a 2.jpg]

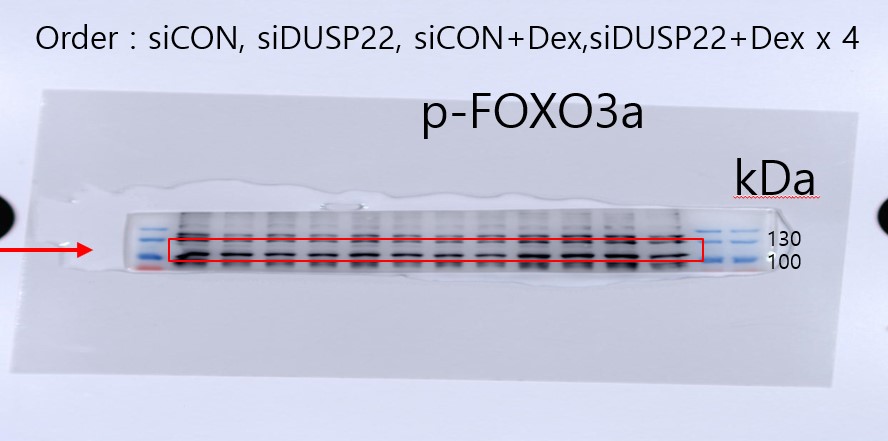

Supplement: Supplementary file 5 — Source data Fig. 3 [file 44321_2025_234_MOESM5_ESM.zip › Figure 3A/Figure 3A p-FOXO3a 3.jpg]

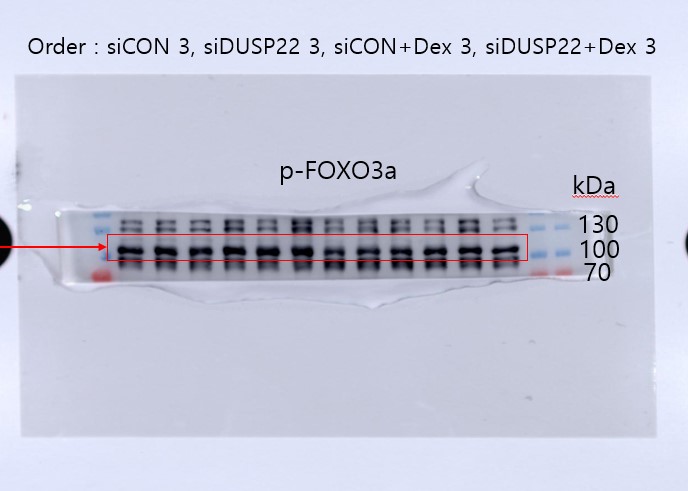

Supplement: Supplementary file 5 — Source data Fig. 3 [file 44321_2025_234_MOESM5_ESM.zip › Figure 3A/Figure 3A p-FOXO3a.jpg]

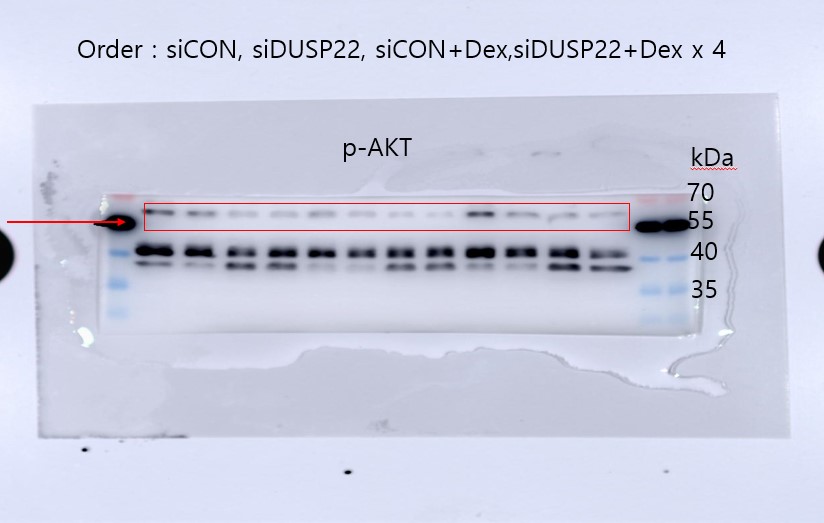

Supplement: Supplementary file 5 — Source data Fig. 3 [file 44321_2025_234_MOESM5_ESM.zip › Figure 3A/Figure 3A p-akt 3.jpg]

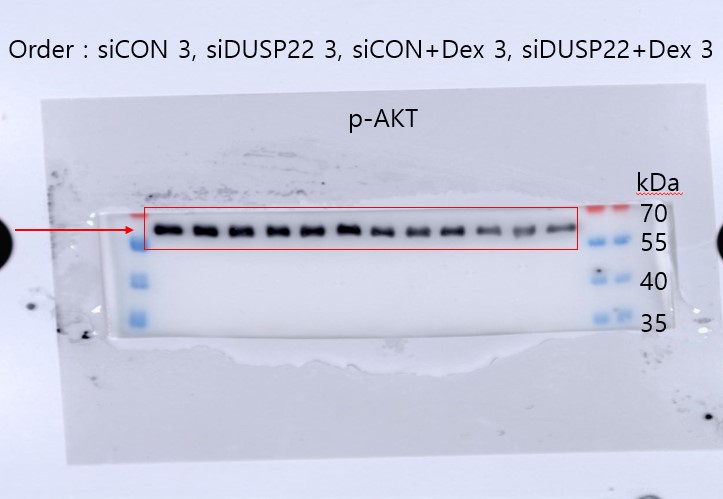

Supplement: Supplementary file 5 — Source data Fig. 3 [file 44321_2025_234_MOESM5_ESM.zip › Figure 3A/Figure 3A p-akt.jpg]

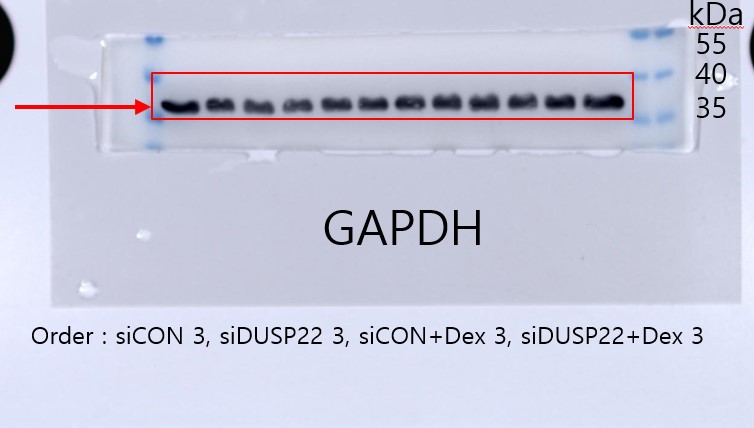

Supplement: Supplementary file 5 — Source data Fig. 3 [file 44321_2025_234_MOESM5_ESM.zip › Figure 3E/FIgure 3E GAPDH.jpg]

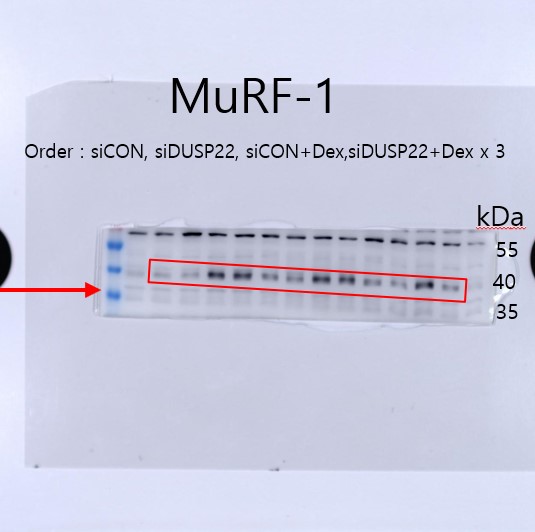

Supplement: Supplementary file 5 — Source data Fig. 3 [file 44321_2025_234_MOESM5_ESM.zip › Figure 3E/FIgure 3E MuRF-1 2.jpg]

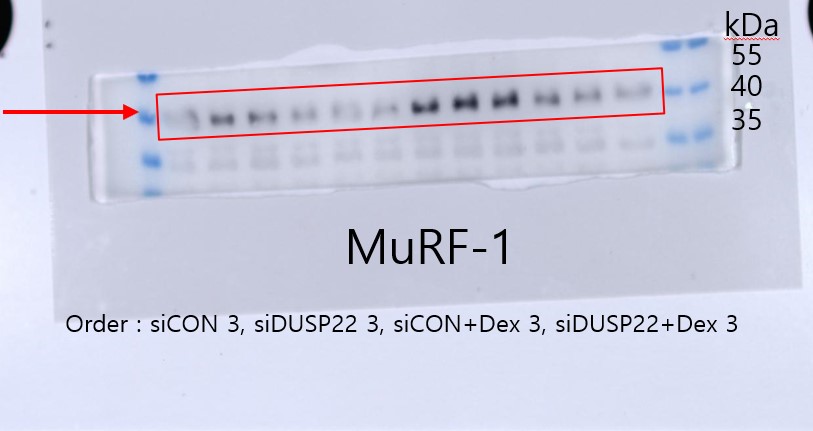

Supplement: Supplementary file 5 — Source data Fig. 3 [file 44321_2025_234_MOESM5_ESM.zip › Figure 3E/FIgure 3E MuRF-1.jpg]

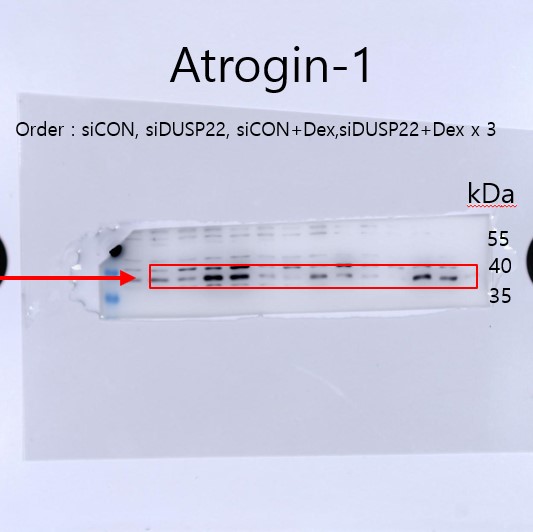

Supplement: Supplementary file 5 — Source data Fig. 3 [file 44321_2025_234_MOESM5_ESM.zip › Figure 3E/Figure 3E Atrogin-1 2.jpg]

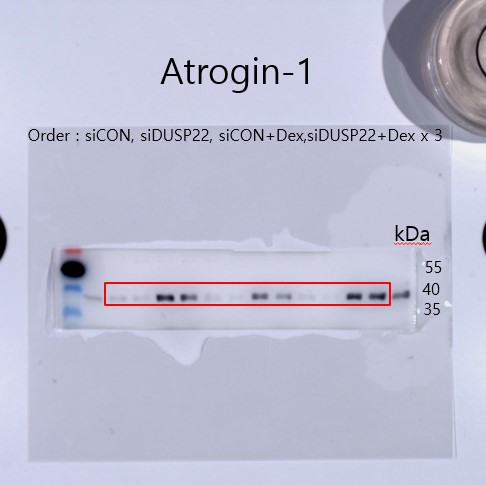

Supplement: Supplementary file 5 — Source data Fig. 3 [file 44321_2025_234_MOESM5_ESM.zip › Figure 3E/Figure 3E Atrogin-1 3.jpg]

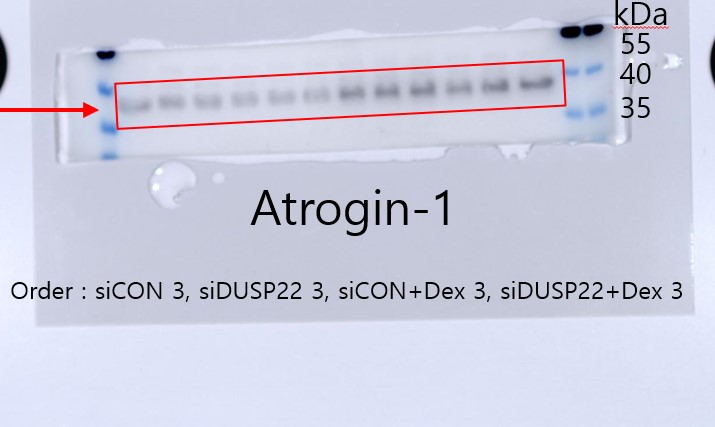

Supplement: Supplementary file 5 — Source data Fig. 3 [file 44321_2025_234_MOESM5_ESM.zip › Figure 3E/Figure 3E Atrogin-1.jpg]

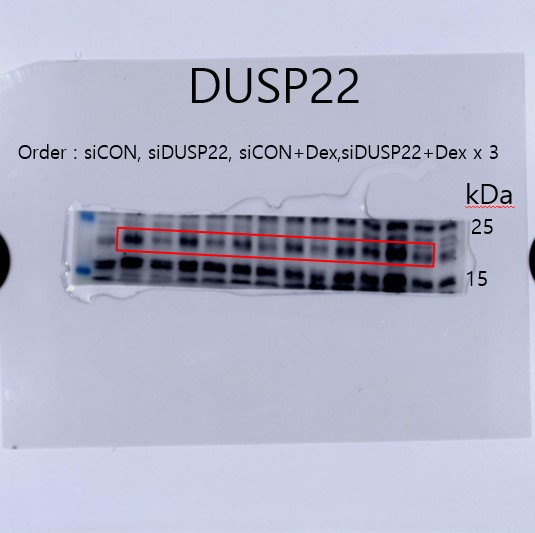

Supplement: Supplementary file 5 — Source data Fig. 3 [file 44321_2025_234_MOESM5_ESM.zip › Figure 3E/Figure 3E DUSP22 2.jpg]

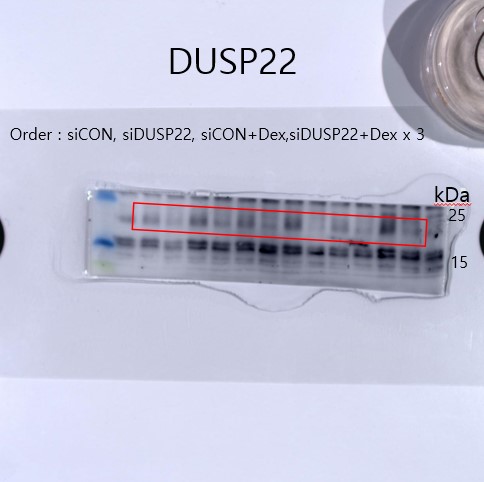

Supplement: Supplementary file 5 — Source data Fig. 3 [file 44321_2025_234_MOESM5_ESM.zip › Figure 3E/Figure 3E DUSP22 3.jpg]

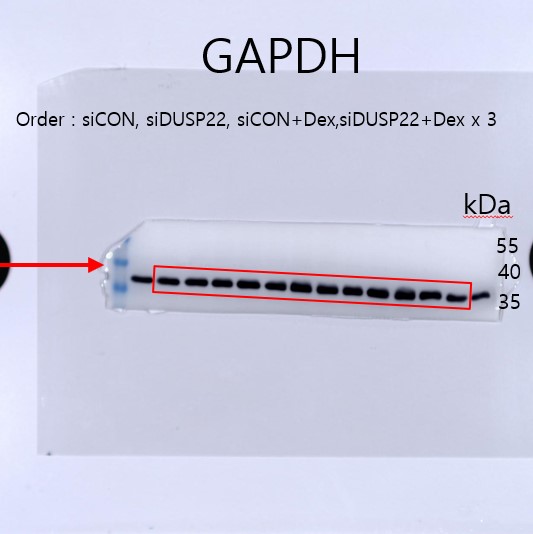

Supplement: Supplementary file 5 — Source data Fig. 3 [file 44321_2025_234_MOESM5_ESM.zip › Figure 3E/Figure 3E GAPDH 2.jpg]

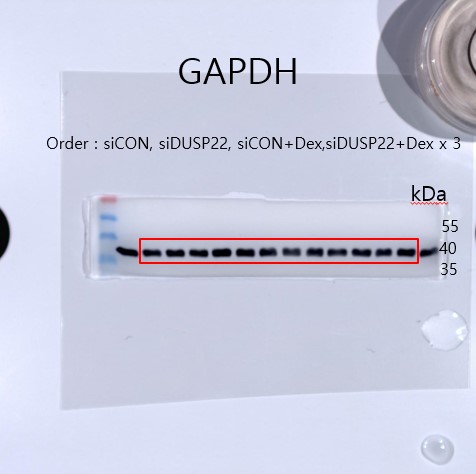

Supplement: Supplementary file 5 — Source data Fig. 3 [file 44321_2025_234_MOESM5_ESM.zip › Figure 3E/Figure 3E GAPDH 3.jpg]

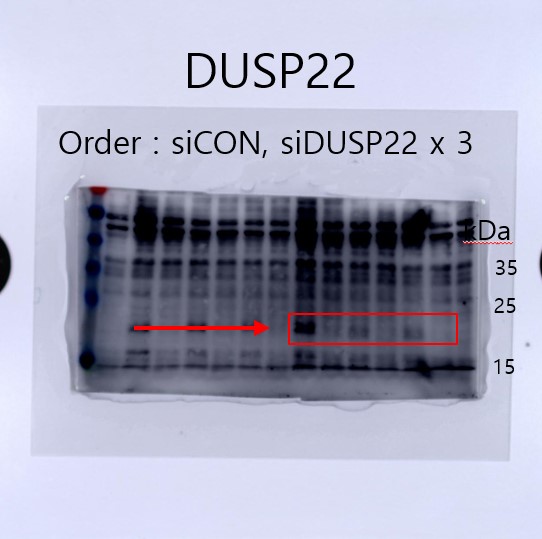

Supplement: Supplementary file 5 — Source data Fig. 3 [file 44321_2025_234_MOESM5_ESM.zip › Figure 3I/FIgure 3I DUSP22 .jpg]

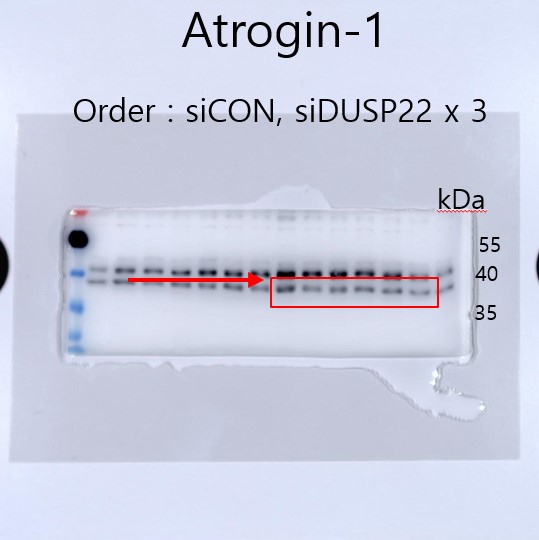

Supplement: Supplementary file 5 — Source data Fig. 3 [file 44321_2025_234_MOESM5_ESM.zip › Figure 3I/Figure 3I Atrogin-1.jpg]

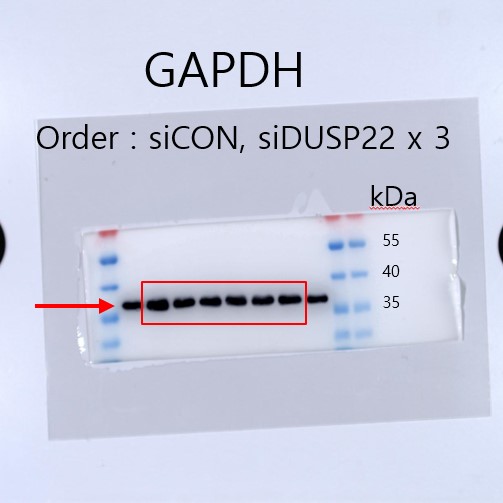

Supplement: Supplementary file 5 — Source data Fig. 3 [file 44321_2025_234_MOESM5_ESM.zip › Figure 3I/Figure 3I GAPDH 2.jpg]

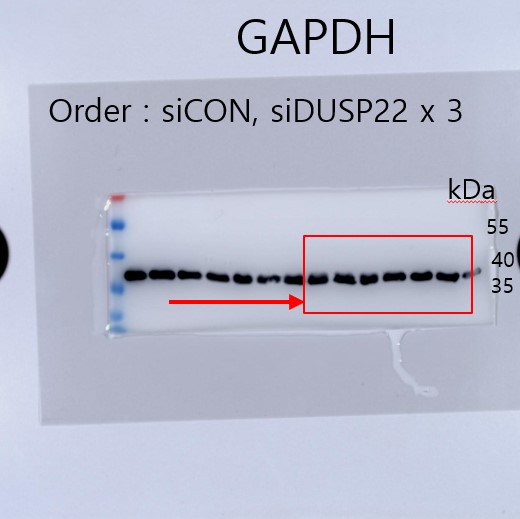

Supplement: Supplementary file 5 — Source data Fig. 3 [file 44321_2025_234_MOESM5_ESM.zip › Figure 3I/Figure 3I GAPDH.jpg]

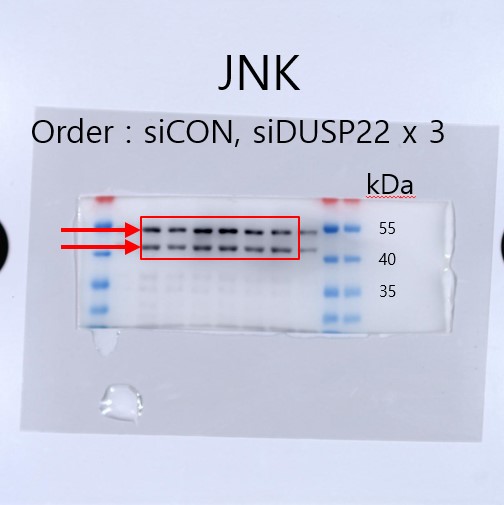

Supplement: Supplementary file 5 — Source data Fig. 3 [file 44321_2025_234_MOESM5_ESM.zip › Figure 3I/Figure 3I JNK 2.jpg]

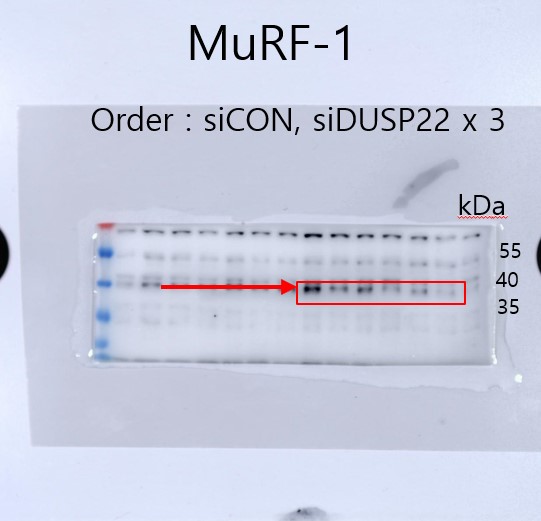

Supplement: Supplementary file 5 — Source data Fig. 3 [file 44321_2025_234_MOESM5_ESM.zip › Figure 3I/Figure 3I MuRF-1.jpg]

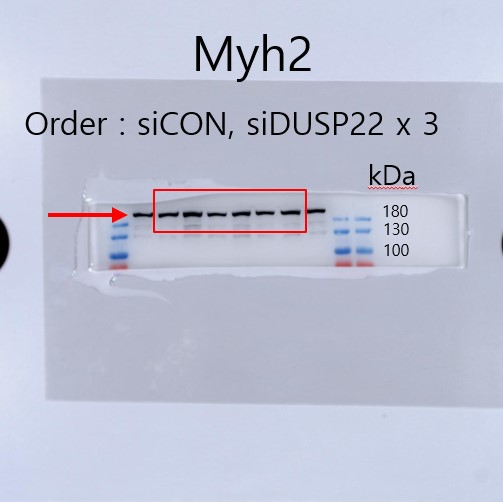

Supplement: Supplementary file 5 — Source data Fig. 3 [file 44321_2025_234_MOESM5_ESM.zip › Figure 3I/Figure 3I Myh2 2.jpg]

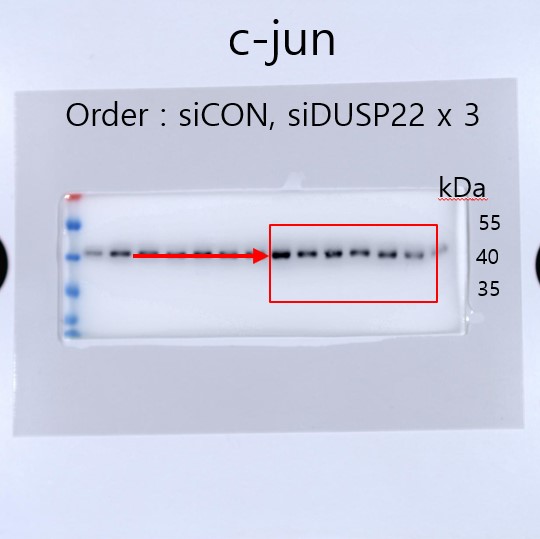

Supplement: Supplementary file 5 — Source data Fig. 3 [file 44321_2025_234_MOESM5_ESM.zip › Figure 3I/Figure 3I c-jun.jpg]

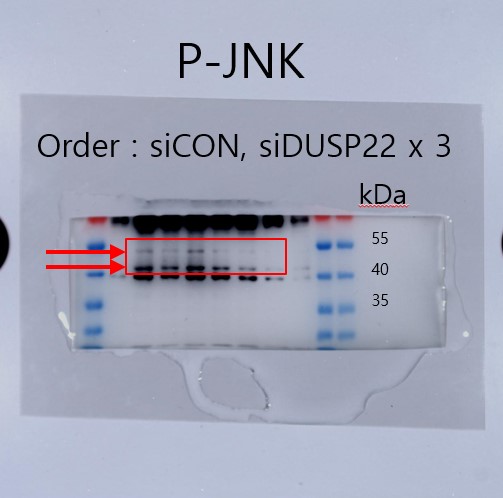

Supplement: Supplementary file 5 — Source data Fig. 3 [file 44321_2025_234_MOESM5_ESM.zip › Figure 3I/Figure 3I p-JNK 2.jpg]
