# Supplementary figures and images for: Modulating phosphatase DUSP22 with BML-260 ameliorates skeletal muscle wasting via Akt independent JNK-FOXO3a repression (part 2 of 3)
Source: EMBO Mol Med. 2025 Apr 22;17(6):1259–88. doi: 10.1038/s44321-025-00234-2 (PMC12162873; doi:10.1038/s44321-025-00234-2)

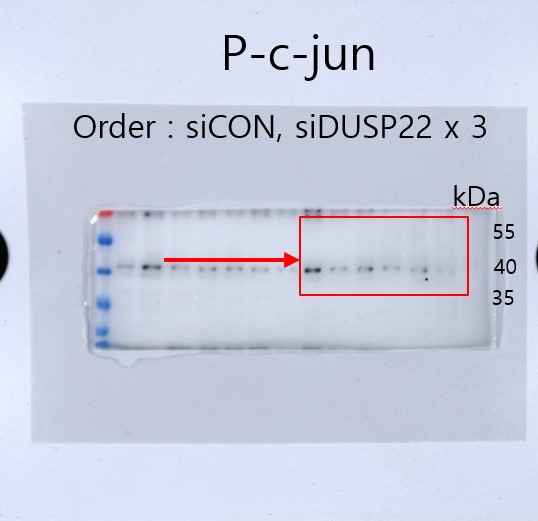

Supplement: Supplementary file 5 — Source data Fig. 3 [file 44321_2025_234_MOESM5_ESM.zip › Figure 3I/Figure 3I p-c-jun.jpg]

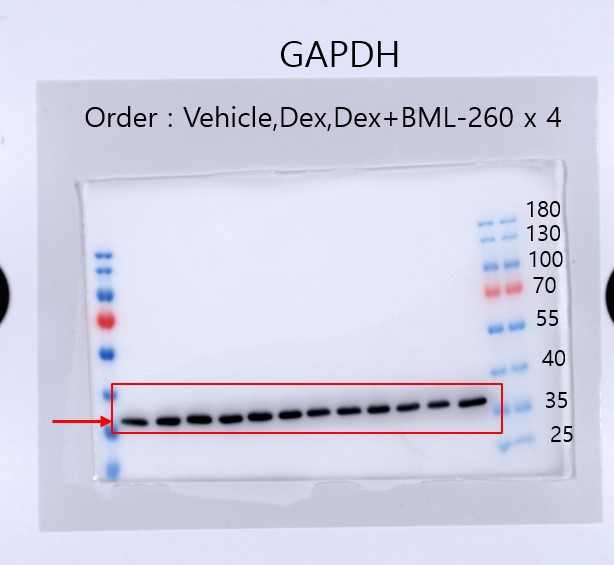

Supplement: Supplementary file 6 — Source data Fig. 4 [file 44321_2025_234_MOESM6_ESM.zip › Figure 4G/Figure 4G GAPDH.jpg]

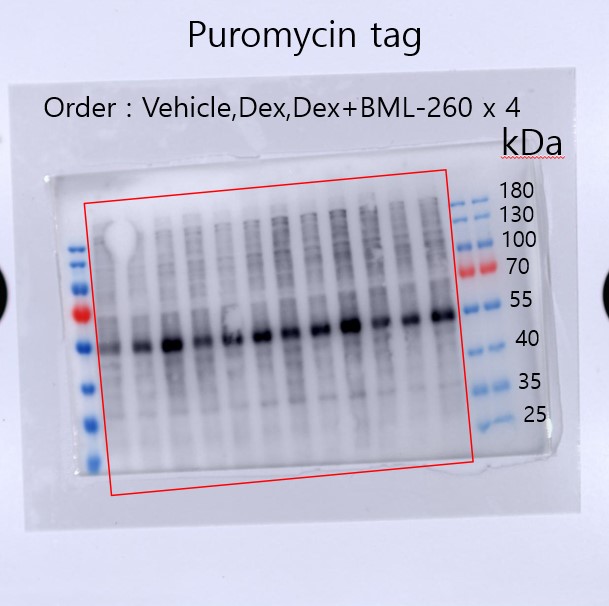

Supplement: Supplementary file 6 — Source data Fig. 4 [file 44321_2025_234_MOESM6_ESM.zip › Figure 4G/Figure 4G puromycin.jpg]

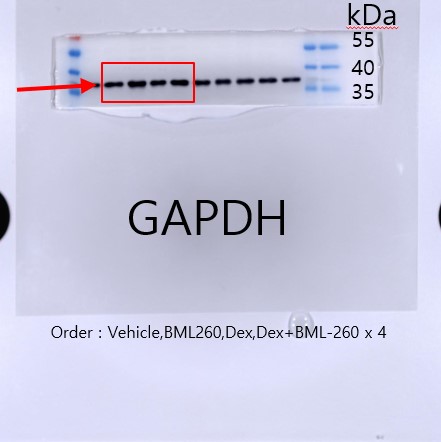

Supplement: Supplementary file 6 — Source data Fig. 4 [file 44321_2025_234_MOESM6_ESM.zip › Figure 4J/Figuer 4J GAPDH DUSP22 2.jpg]

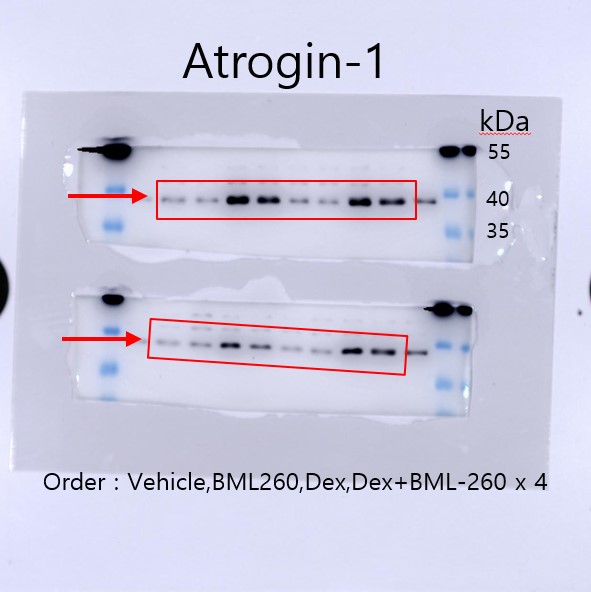

Supplement: Supplementary file 6 — Source data Fig. 4 [file 44321_2025_234_MOESM6_ESM.zip › Figure 4J/Figure 4J Atrogin-1 2.jpg]

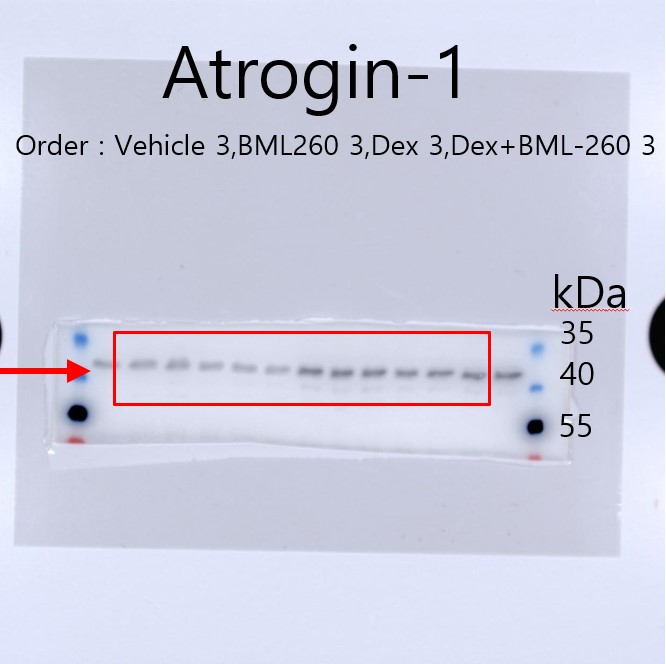

Supplement: Supplementary file 6 — Source data Fig. 4 [file 44321_2025_234_MOESM6_ESM.zip › Figure 4J/Figure 4J Atrogin-1.jpg]

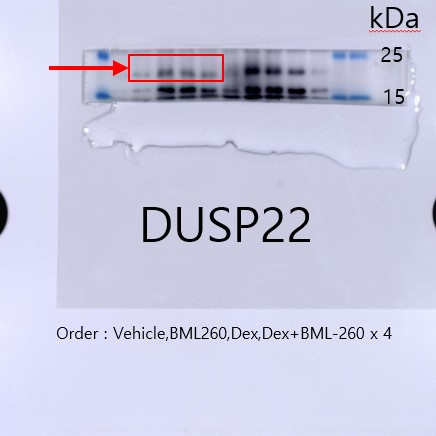

Supplement: Supplementary file 6 — Source data Fig. 4 [file 44321_2025_234_MOESM6_ESM.zip › Figure 4J/Figure 4J DUSP22 2.jpg]

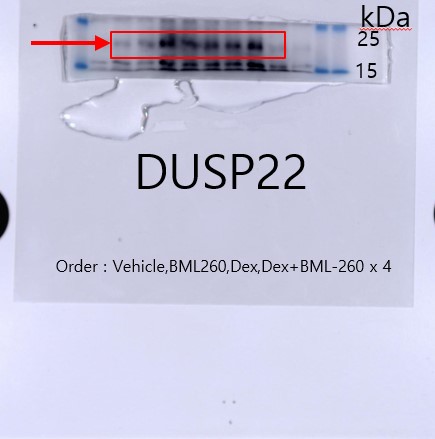

Supplement: Supplementary file 6 — Source data Fig. 4 [file 44321_2025_234_MOESM6_ESM.zip › Figure 4J/Figure 4J DUSP22.jpg]

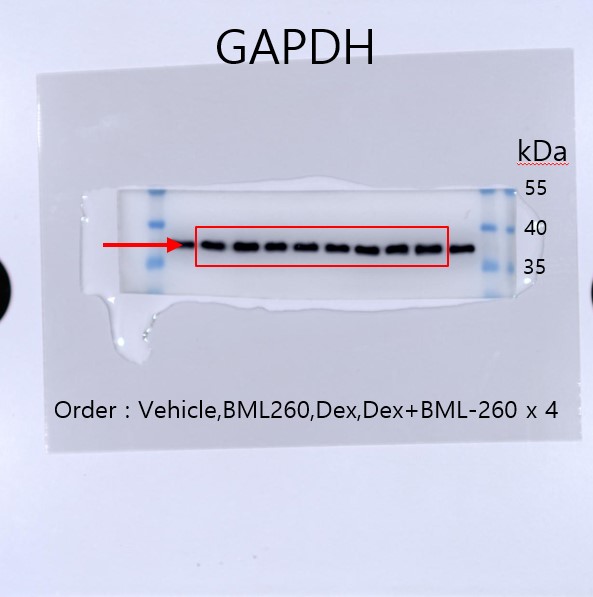

Supplement: Supplementary file 6 — Source data Fig. 4 [file 44321_2025_234_MOESM6_ESM.zip › Figure 4J/Figure 4J GAPDH 2,.jpg]

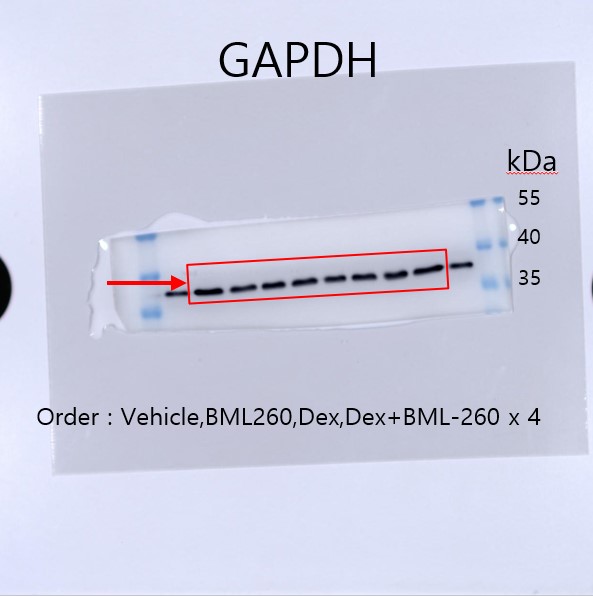

Supplement: Supplementary file 6 — Source data Fig. 4 [file 44321_2025_234_MOESM6_ESM.zip › Figure 4J/Figure 4J GAPDH 2.jpg]

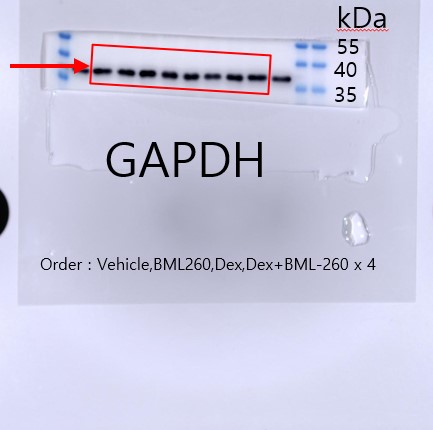

Supplement: Supplementary file 6 — Source data Fig. 4 [file 44321_2025_234_MOESM6_ESM.zip › Figure 4J/Figure 4J GAPDH DUSP.jpg]

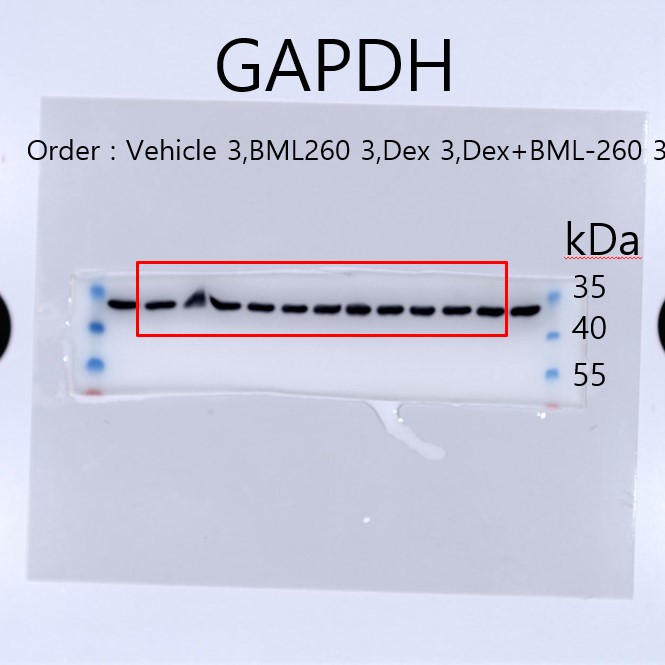

Supplement: Supplementary file 6 — Source data Fig. 4 [file 44321_2025_234_MOESM6_ESM.zip › Figure 4J/Figure 4J GAPDH.jpg]

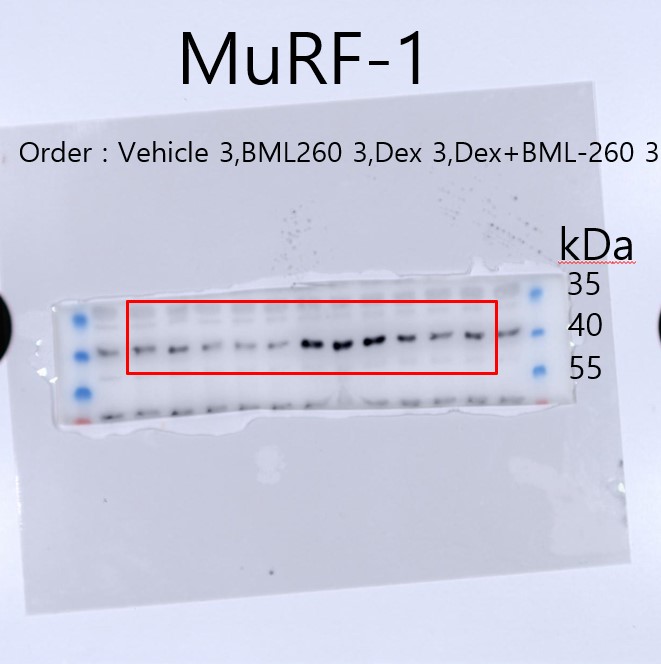

Supplement: Supplementary file 6 — Source data Fig. 4 [file 44321_2025_234_MOESM6_ESM.zip › Figure 4J/Figure 4J MuRF-1.jpg]

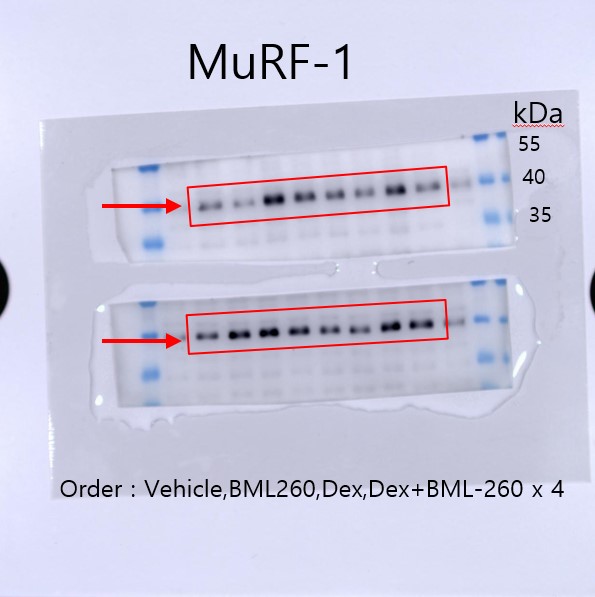

Supplement: Supplementary file 6 — Source data Fig. 4 [file 44321_2025_234_MOESM6_ESM.zip › Figure 4J/Figure 4J Murf-1 2.jpg]

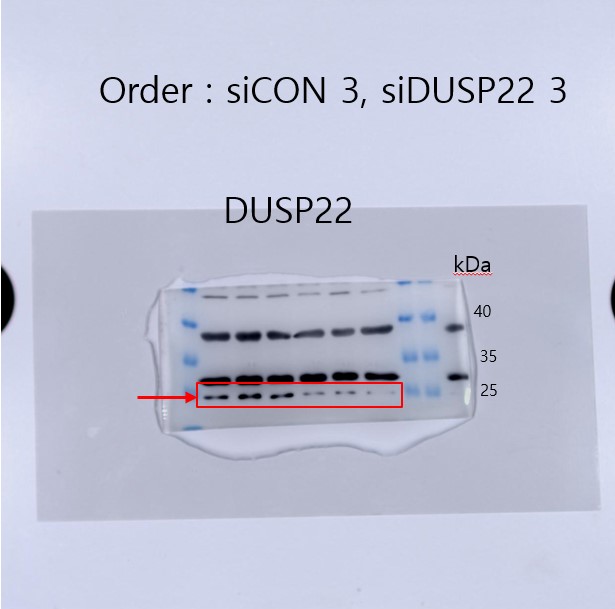

Supplement: Supplementary file 7 — Source data Fig. 5 [file 44321_2025_234_MOESM7_ESM.zip › Figure 5B/Figure 5B DUSP22.jpg]

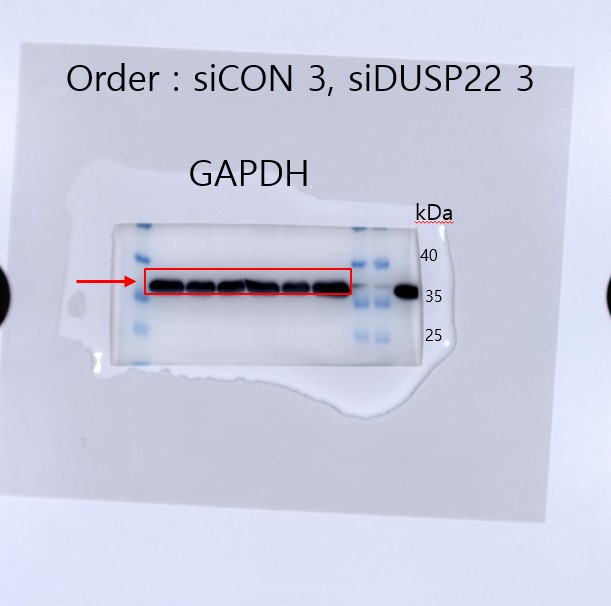

Supplement: Supplementary file 7 — Source data Fig. 5 [file 44321_2025_234_MOESM7_ESM.zip › Figure 5B/Figure 5B GAPDH.jpg]

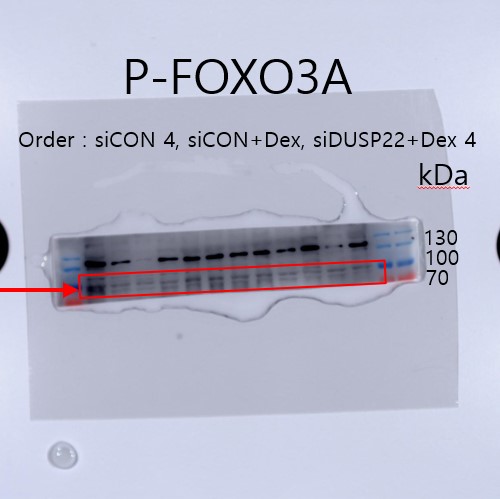

Supplement: Supplementary file 7 — Source data Fig. 5 [file 44321_2025_234_MOESM7_ESM.zip › Figure 5J/FIgure 5J p-FOXO3a.jpg]

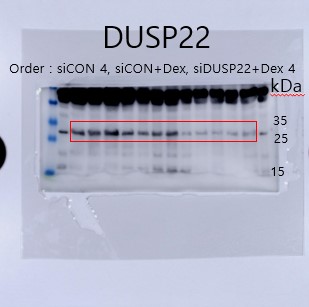

Supplement: Supplementary file 7 — Source data Fig. 5 [file 44321_2025_234_MOESM7_ESM.zip › Figure 5J/Figure 5J DUSP22.jpg]

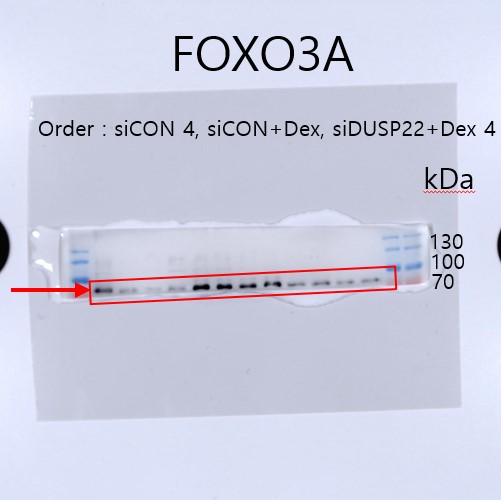

Supplement: Supplementary file 7 — Source data Fig. 5 [file 44321_2025_234_MOESM7_ESM.zip › Figure 5J/Figure 5J FOXO3a.jpg]

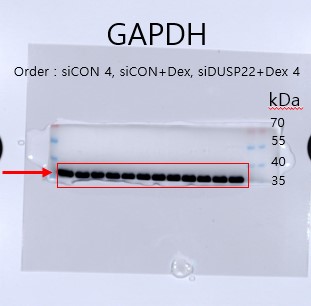

Supplement: Supplementary file 7 — Source data Fig. 5 [file 44321_2025_234_MOESM7_ESM.zip › Figure 5J/Figure 5J GAPDH 1.jpg]

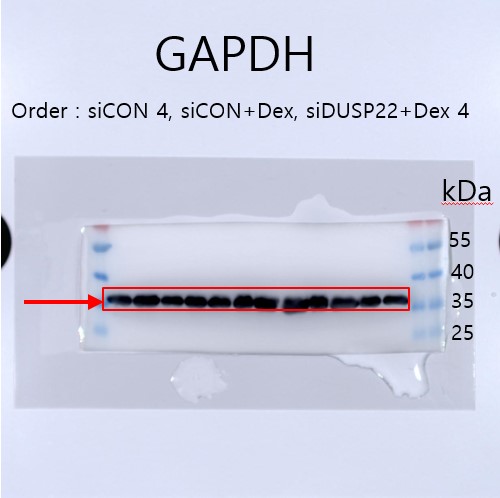

Supplement: Supplementary file 7 — Source data Fig. 5 [file 44321_2025_234_MOESM7_ESM.zip › Figure 5J/Figure 5J GAPDH 2.jpg]

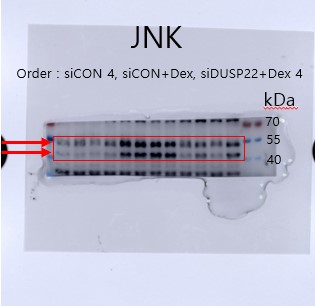

Supplement: Supplementary file 7 — Source data Fig. 5 [file 44321_2025_234_MOESM7_ESM.zip › Figure 5J/Figure 5J JNK.jpg]

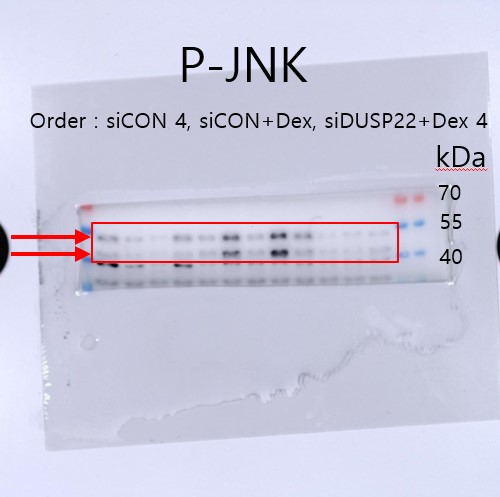

Supplement: Supplementary file 7 — Source data Fig. 5 [file 44321_2025_234_MOESM7_ESM.zip › Figure 5J/Figure 5J P-JNK.jpg]

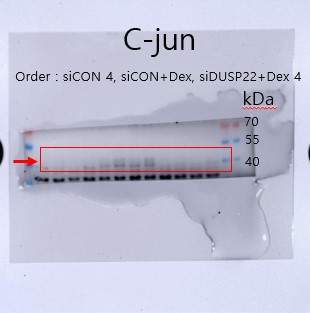

Supplement: Supplementary file 7 — Source data Fig. 5 [file 44321_2025_234_MOESM7_ESM.zip › Figure 5J/Figure 5J c-jun.jpg]

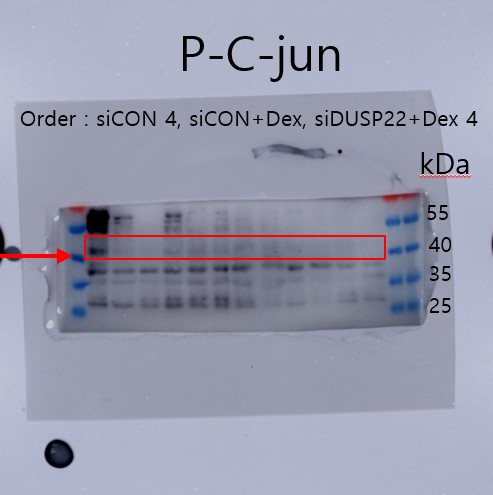

Supplement: Supplementary file 7 — Source data Fig. 5 [file 44321_2025_234_MOESM7_ESM.zip › Figure 5J/Figure 5J p-c-jun.jpg]

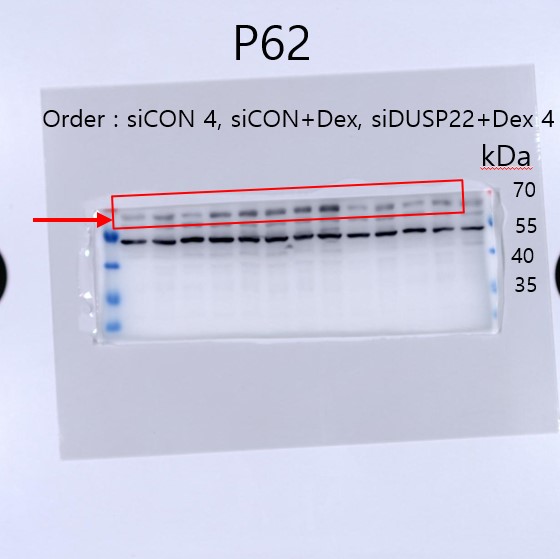

Supplement: Supplementary file 7 — Source data Fig. 5 [file 44321_2025_234_MOESM7_ESM.zip › Figure 5M/FIgure 5M P62.jpg]

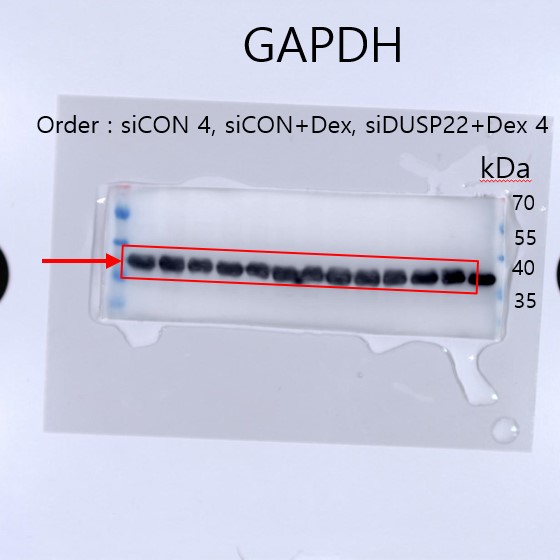

Supplement: Supplementary file 7 — Source data Fig. 5 [file 44321_2025_234_MOESM7_ESM.zip › Figure 5M/Figure 5M GAPDH.jpg]

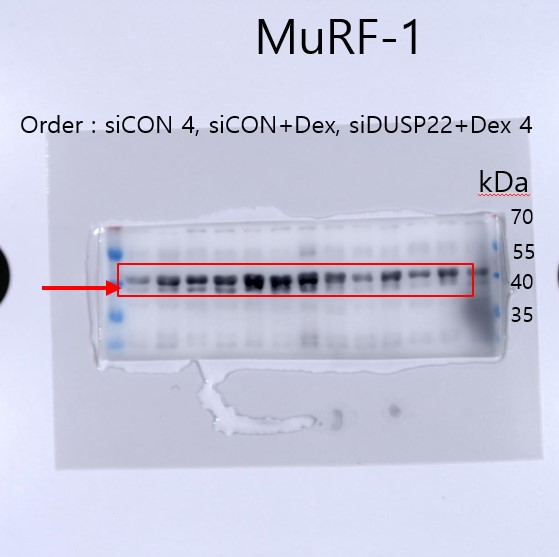

Supplement: Supplementary file 7 — Source data Fig. 5 [file 44321_2025_234_MOESM7_ESM.zip › Figure 5M/Figure 5M MuRF-1.jpg]

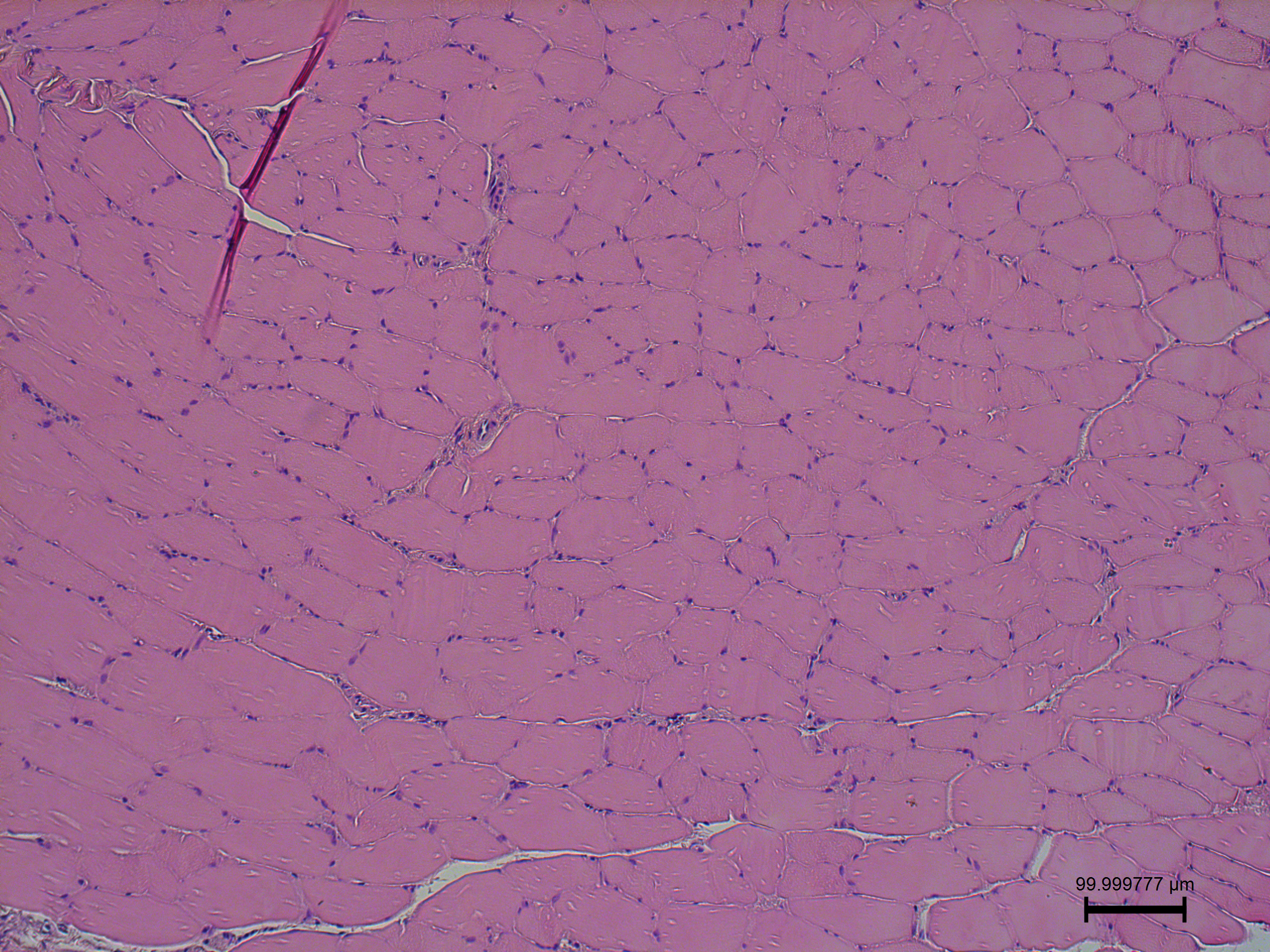

Supplement: Supplementary file 8 — Source data Fig. 6 [file 44321_2025_234_MOESM8_ESM.zip › Figure 6E/Dex/Figure'/Dex0004.tif]

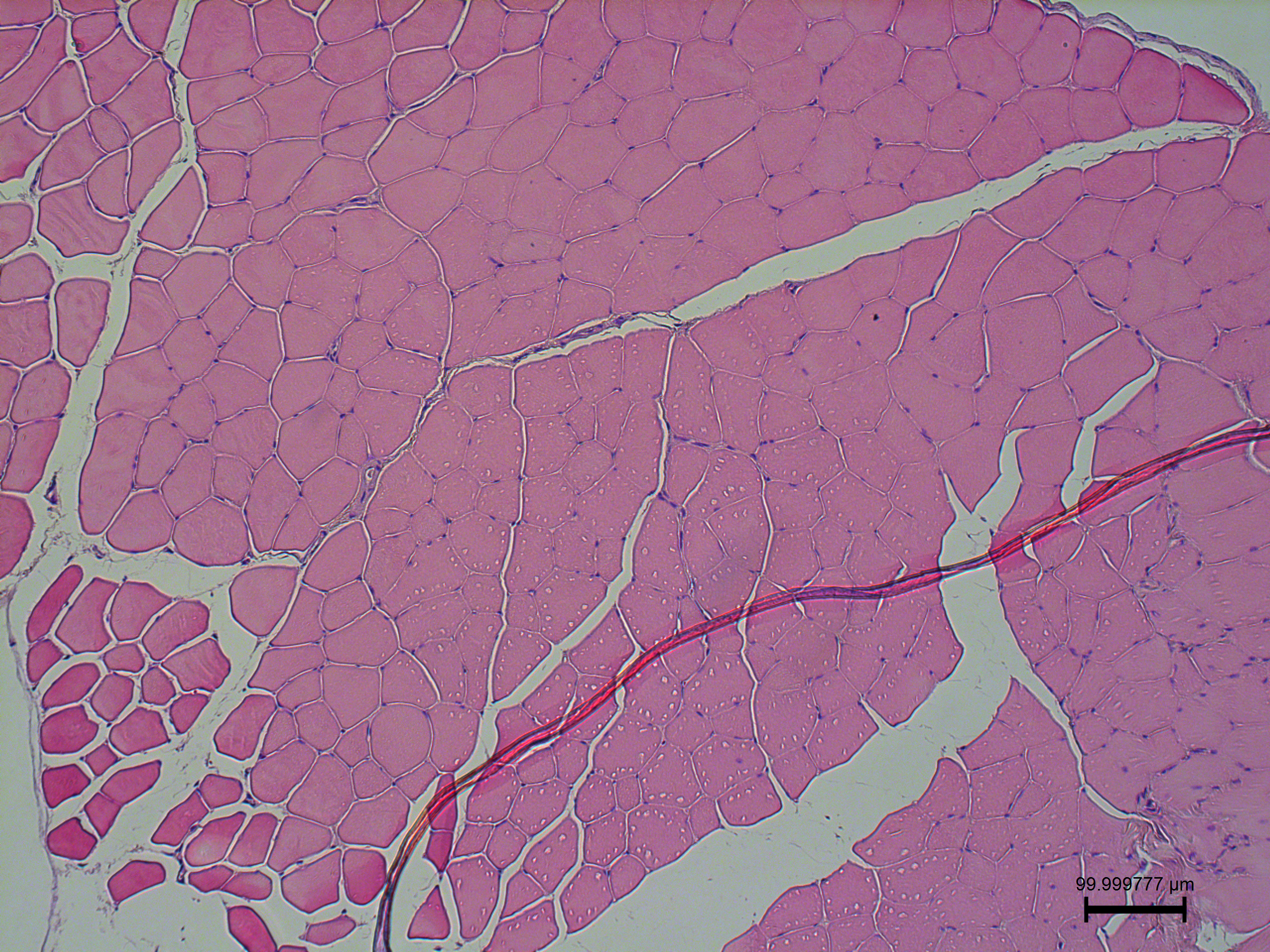

Supplement: Supplementary file 8 — Source data Fig. 6 [file 44321_2025_234_MOESM8_ESM.zip › Figure 6E/Dex/Figure'/Dex0014.tif]

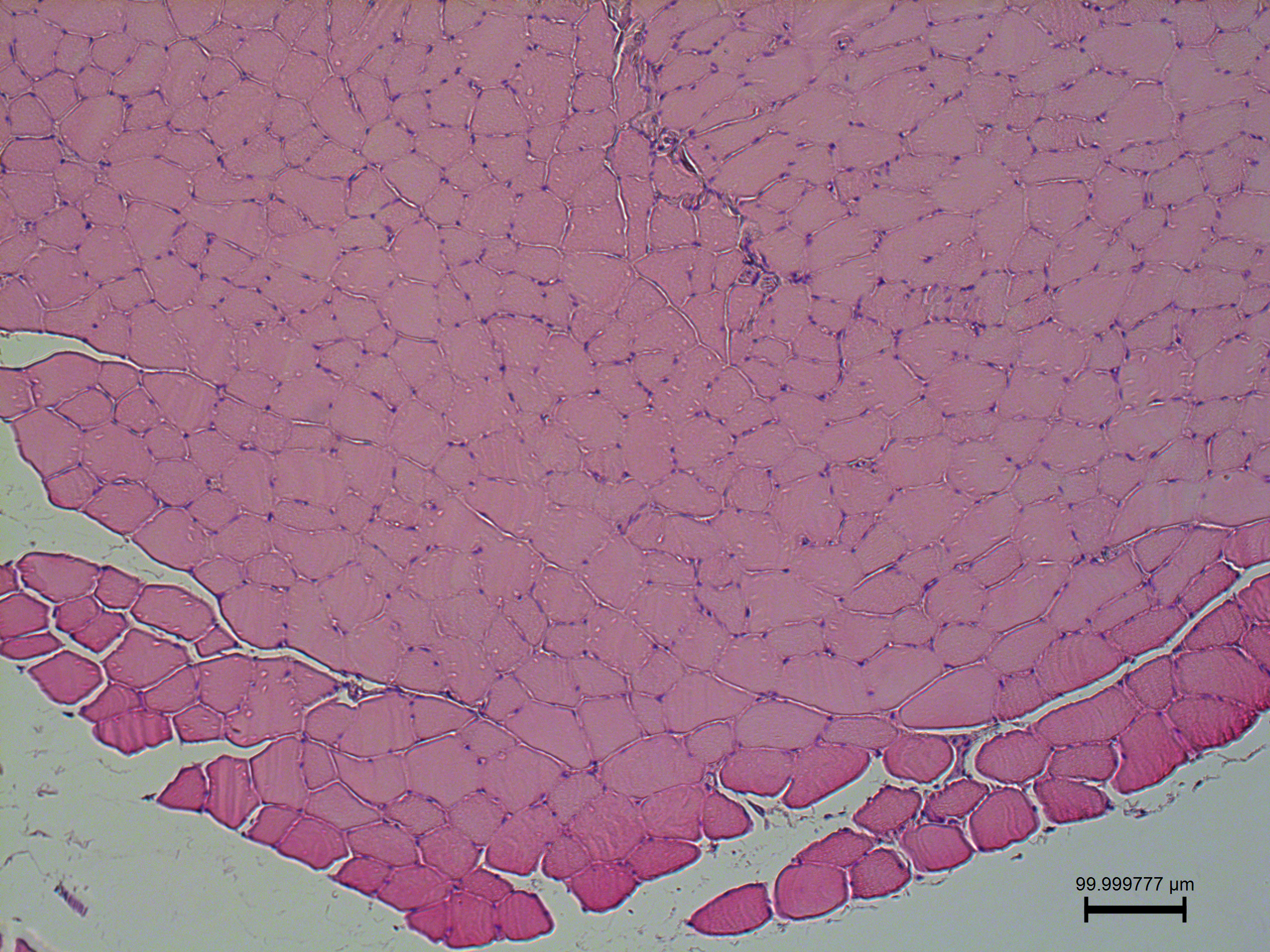

Supplement: Supplementary file 8 — Source data Fig. 6 [file 44321_2025_234_MOESM8_ESM.zip › Figure 6E/Dex/Figure'/Dex0017.tif]

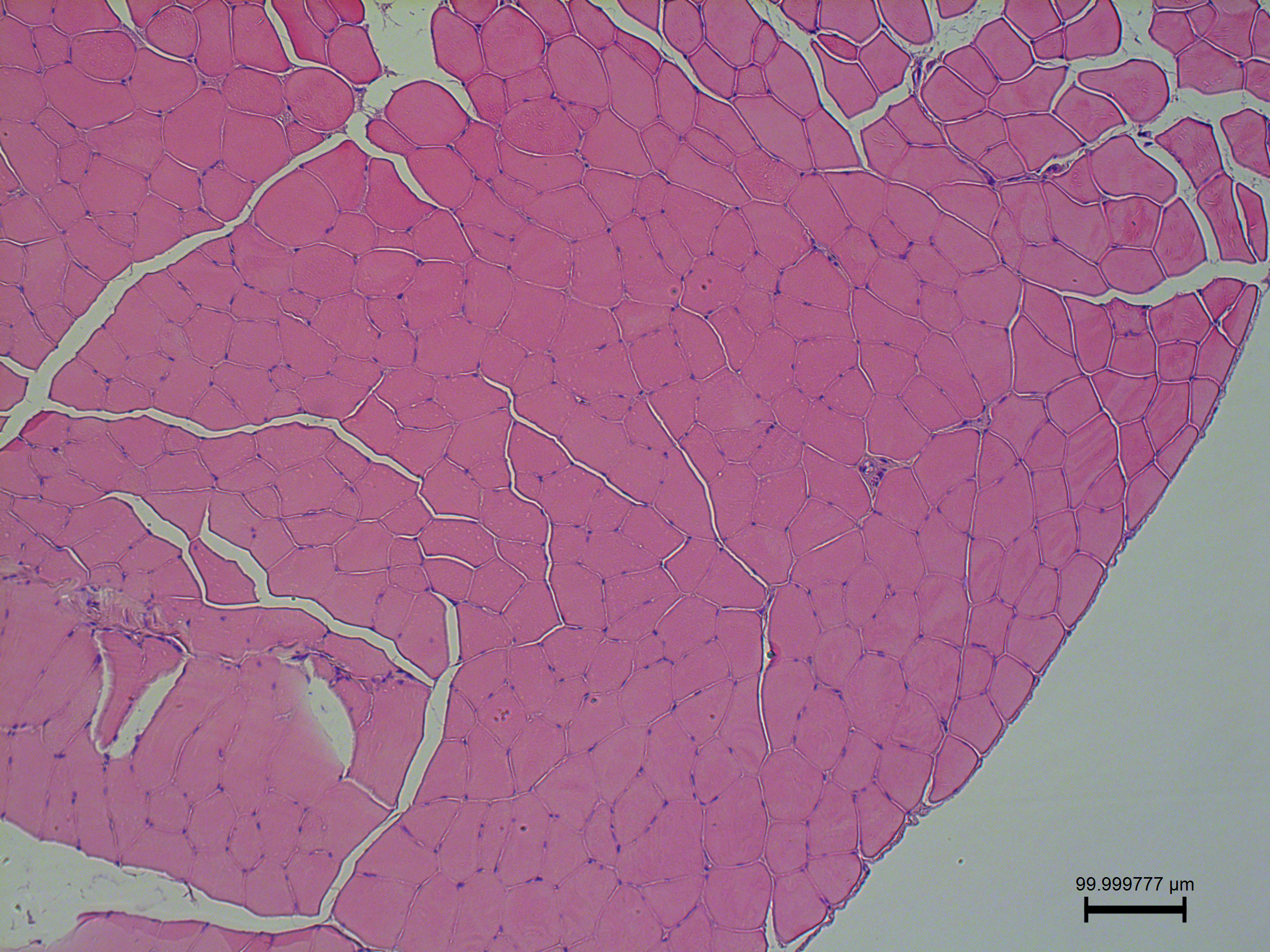

Supplement: Supplementary file 8 — Source data Fig. 6 [file 44321_2025_234_MOESM8_ESM.zip › Figure 6E/Dex/Figure'/Dex0022.tif]

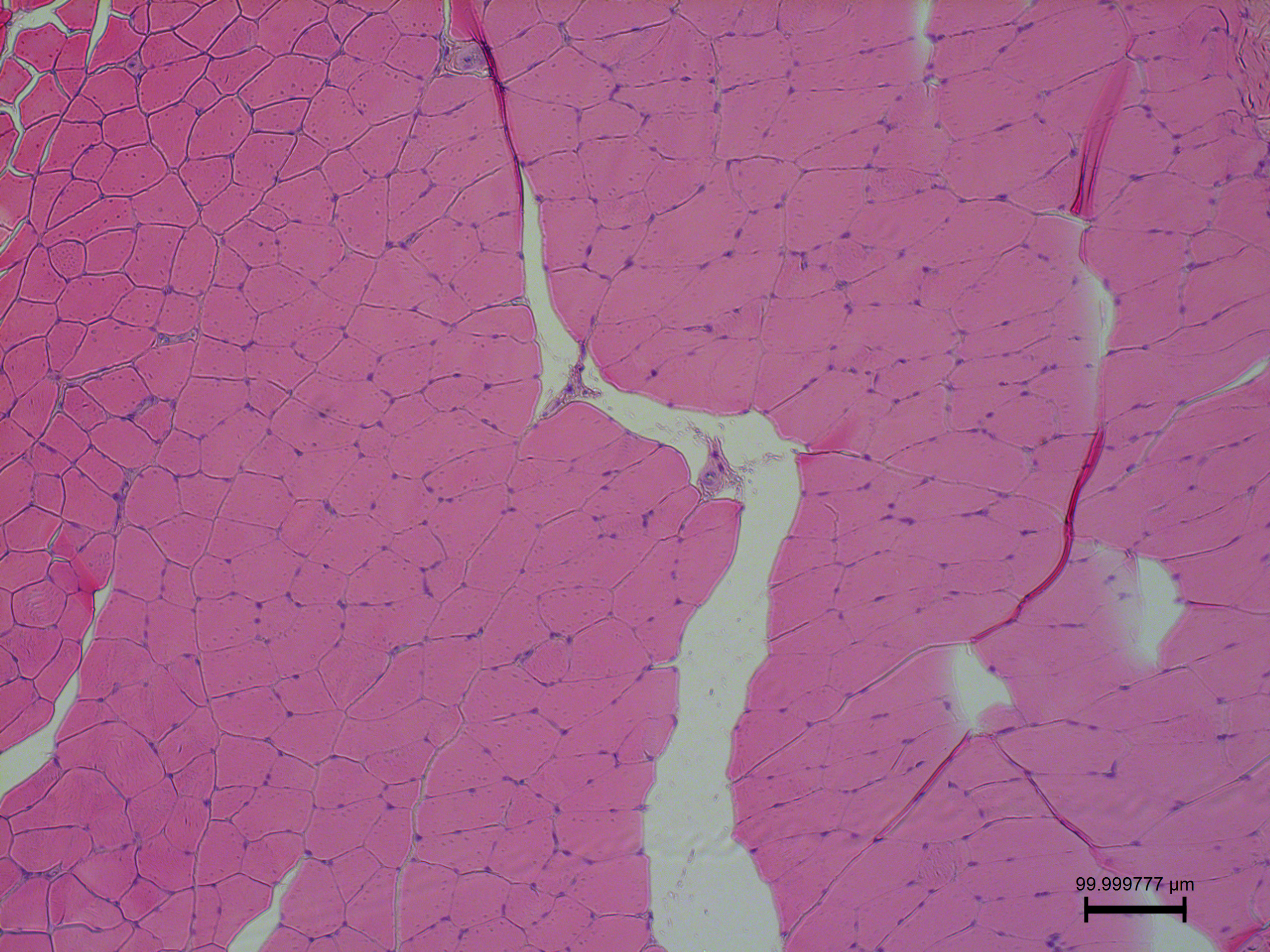

Supplement: Supplementary file 8 — Source data Fig. 6 [file 44321_2025_234_MOESM8_ESM.zip › Figure 6E/Dex+BML 5/Figure/BML (2).tif]

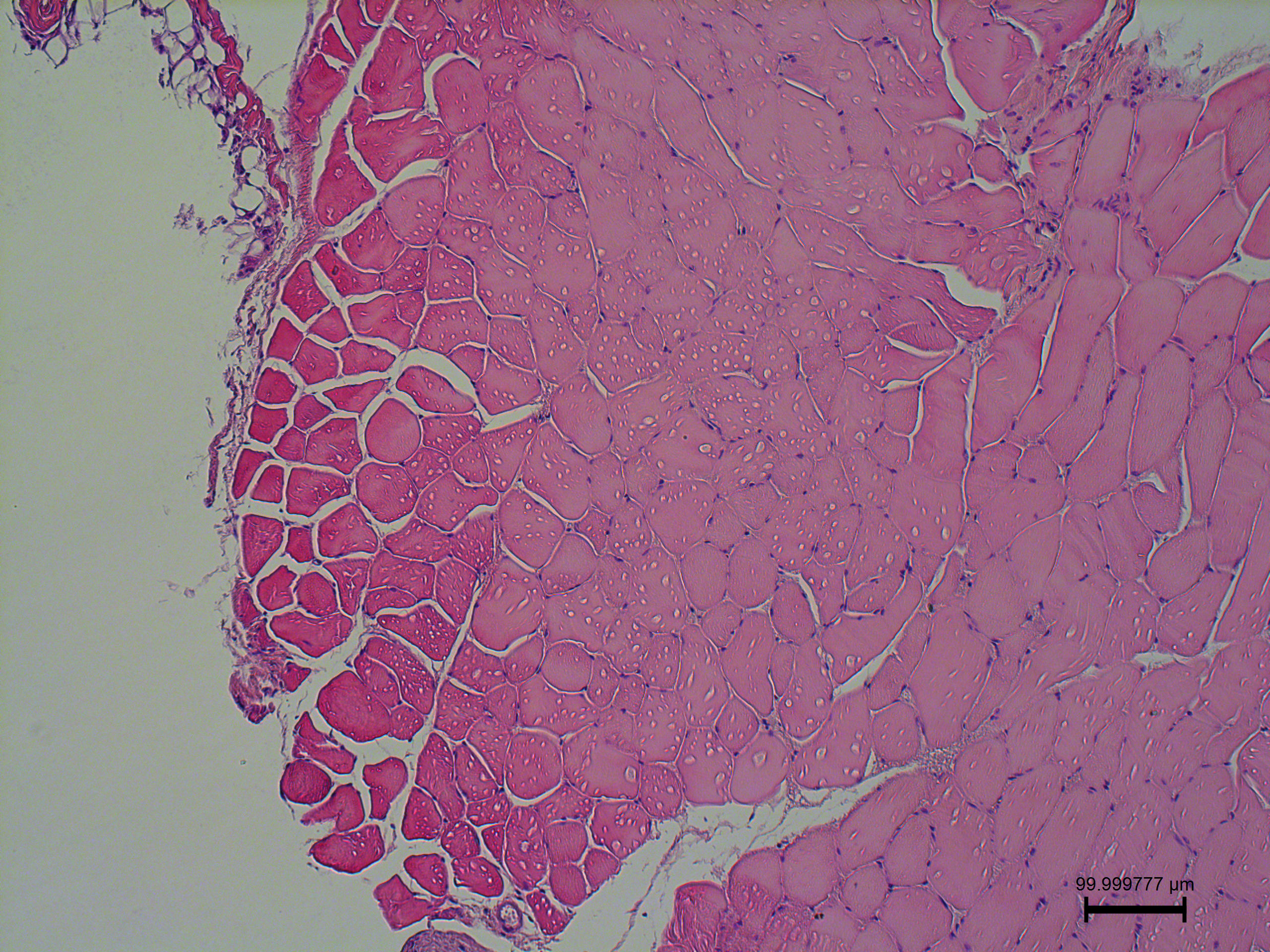

Supplement: Supplementary file 8 — Source data Fig. 6 [file 44321_2025_234_MOESM8_ESM.zip › Figure 6E/Dex+BML 5/Figure/BML (3).tif]

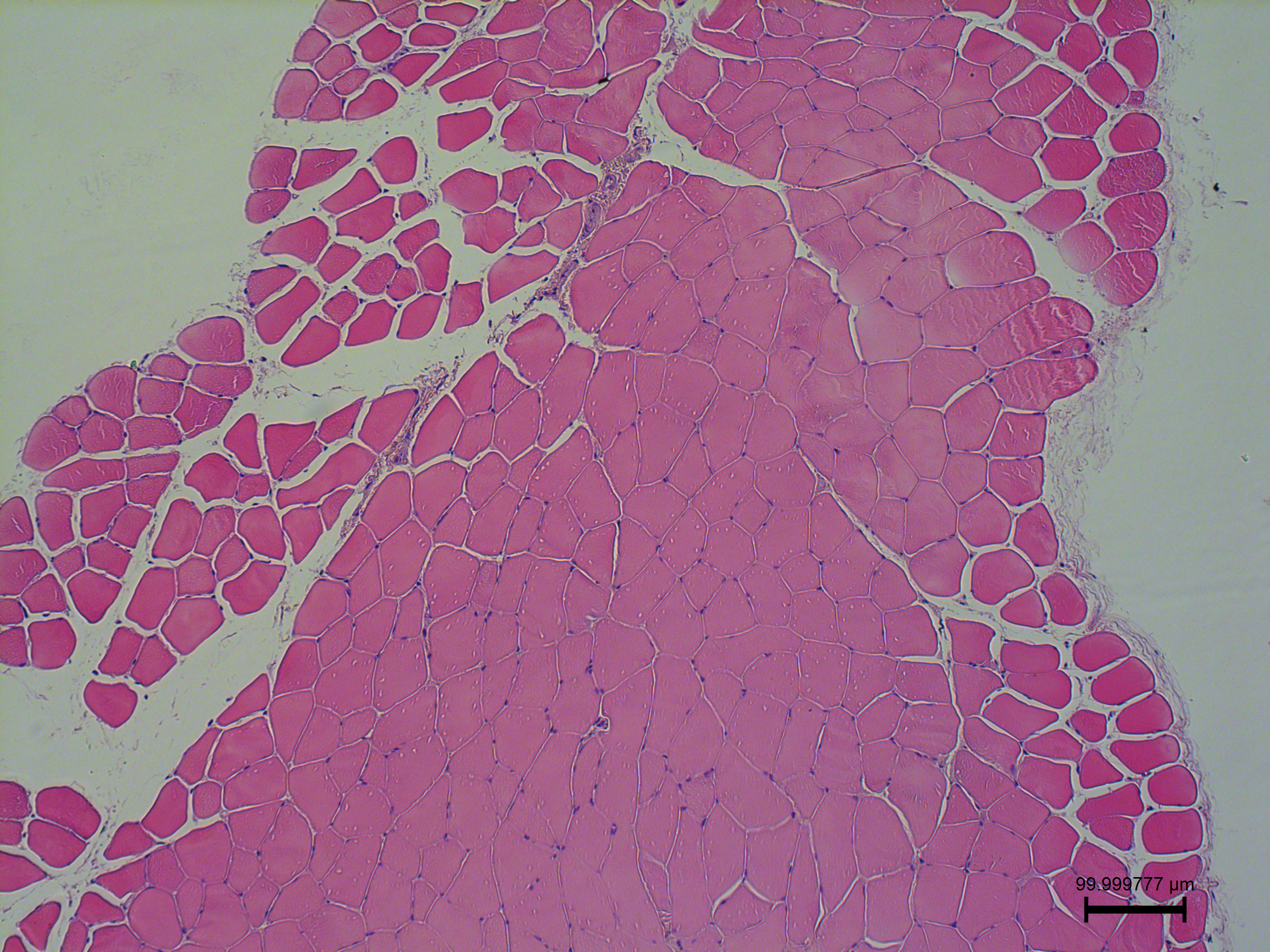

Supplement: Supplementary file 8 — Source data Fig. 6 [file 44321_2025_234_MOESM8_ESM.zip › Figure 6E/Dex+BML 5/Figure/BML (4).tif]

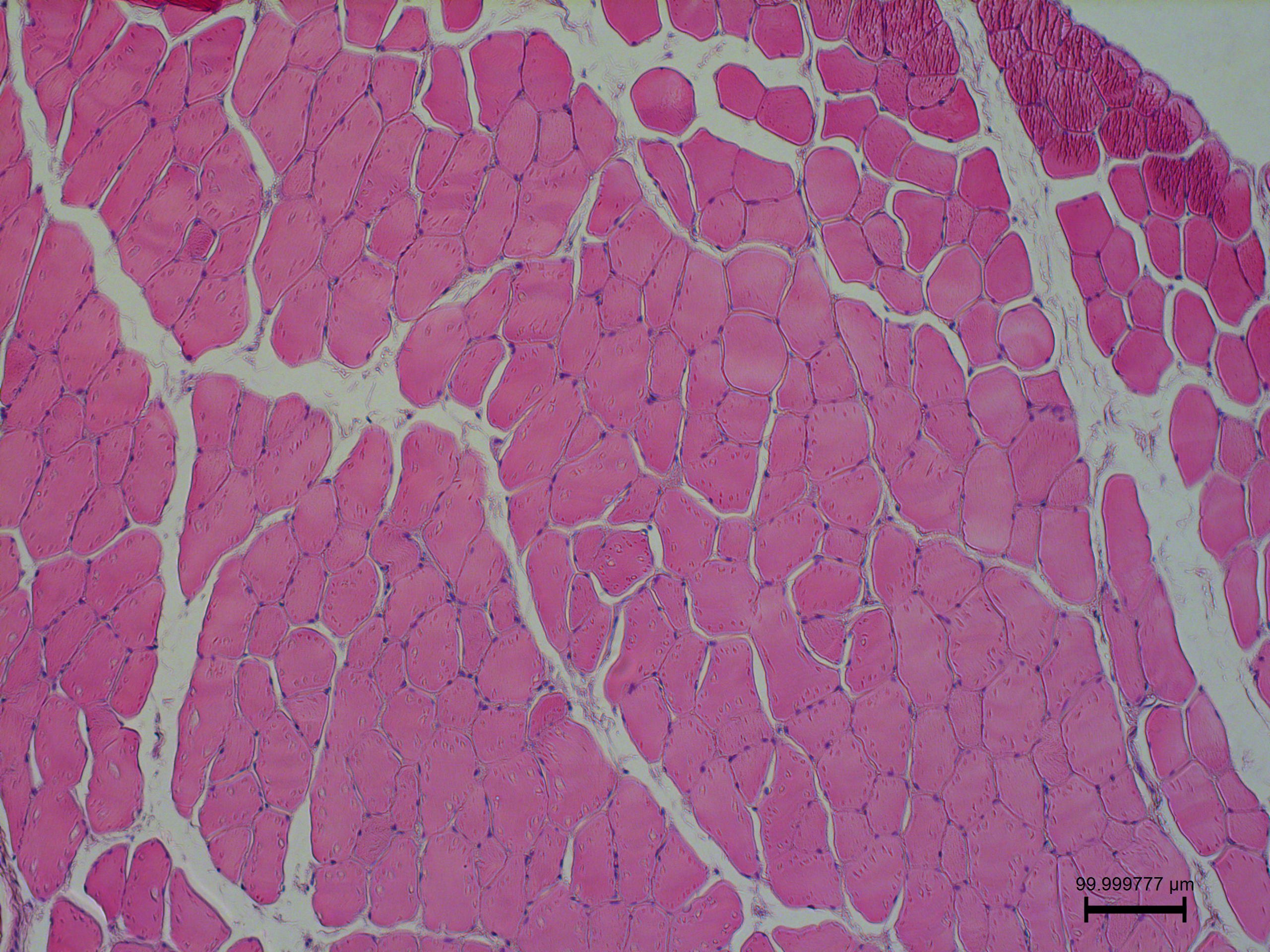

Supplement: Supplementary file 8 — Source data Fig. 6 [file 44321_2025_234_MOESM8_ESM.zip › Figure 6E/Dex+BML 5/Figure/BML.tif]

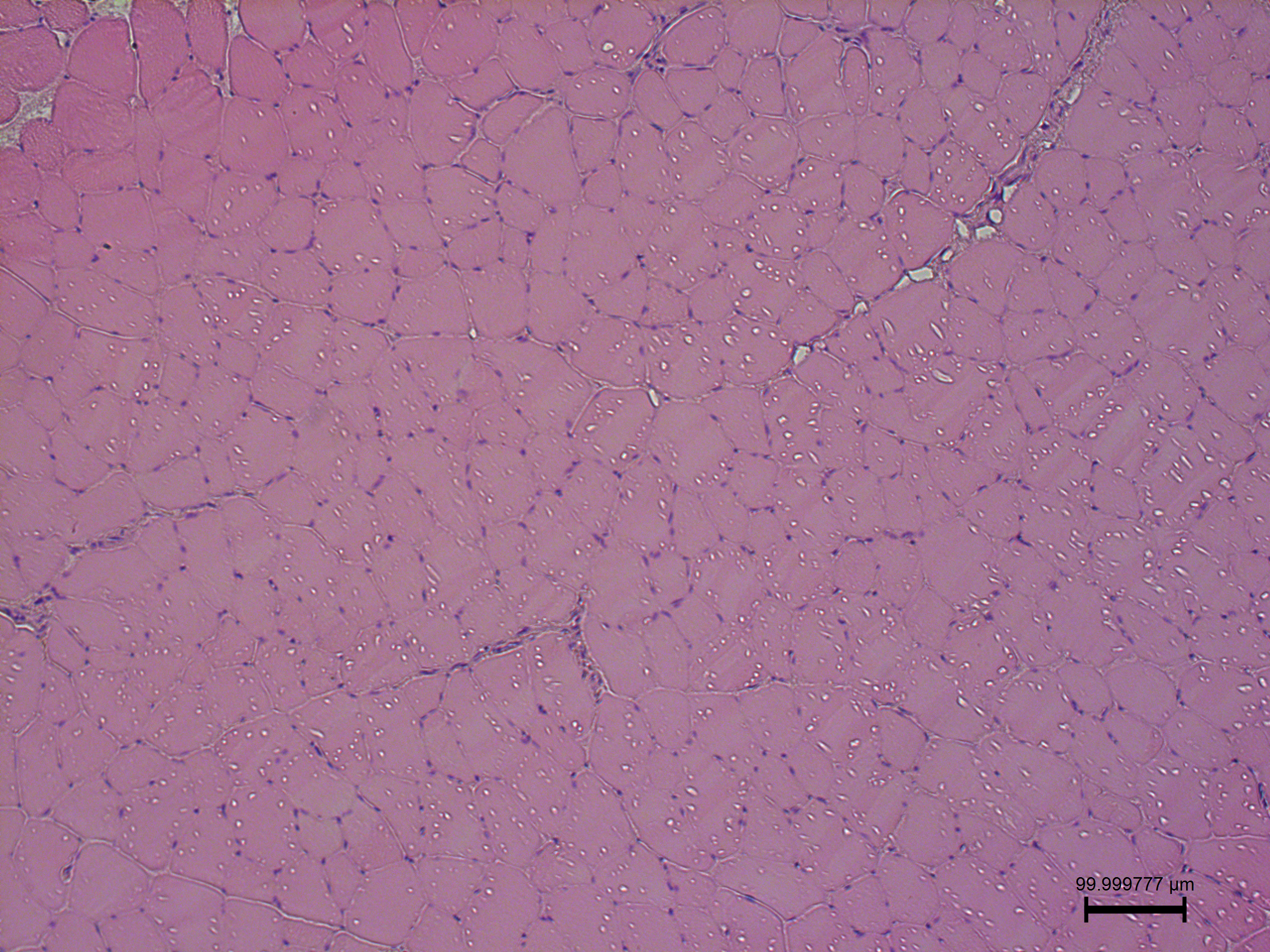

Supplement: Supplementary file 8 — Source data Fig. 6 [file 44321_2025_234_MOESM8_ESM.zip › Figure 6E/Vehicle/Figure/con0008.tif]

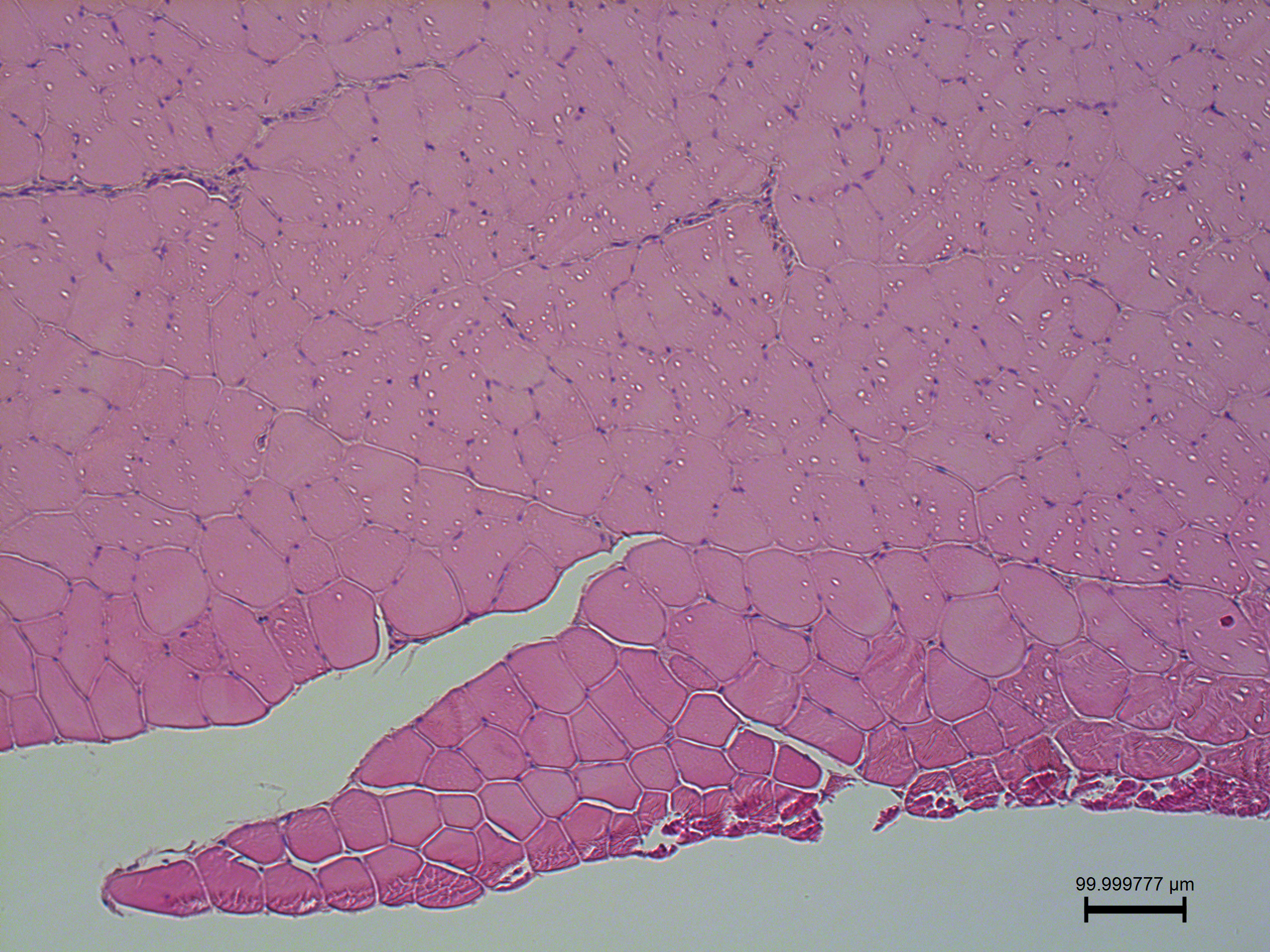

Supplement: Supplementary file 8 — Source data Fig. 6 [file 44321_2025_234_MOESM8_ESM.zip › Figure 6E/Vehicle/Figure/con0010.tif]

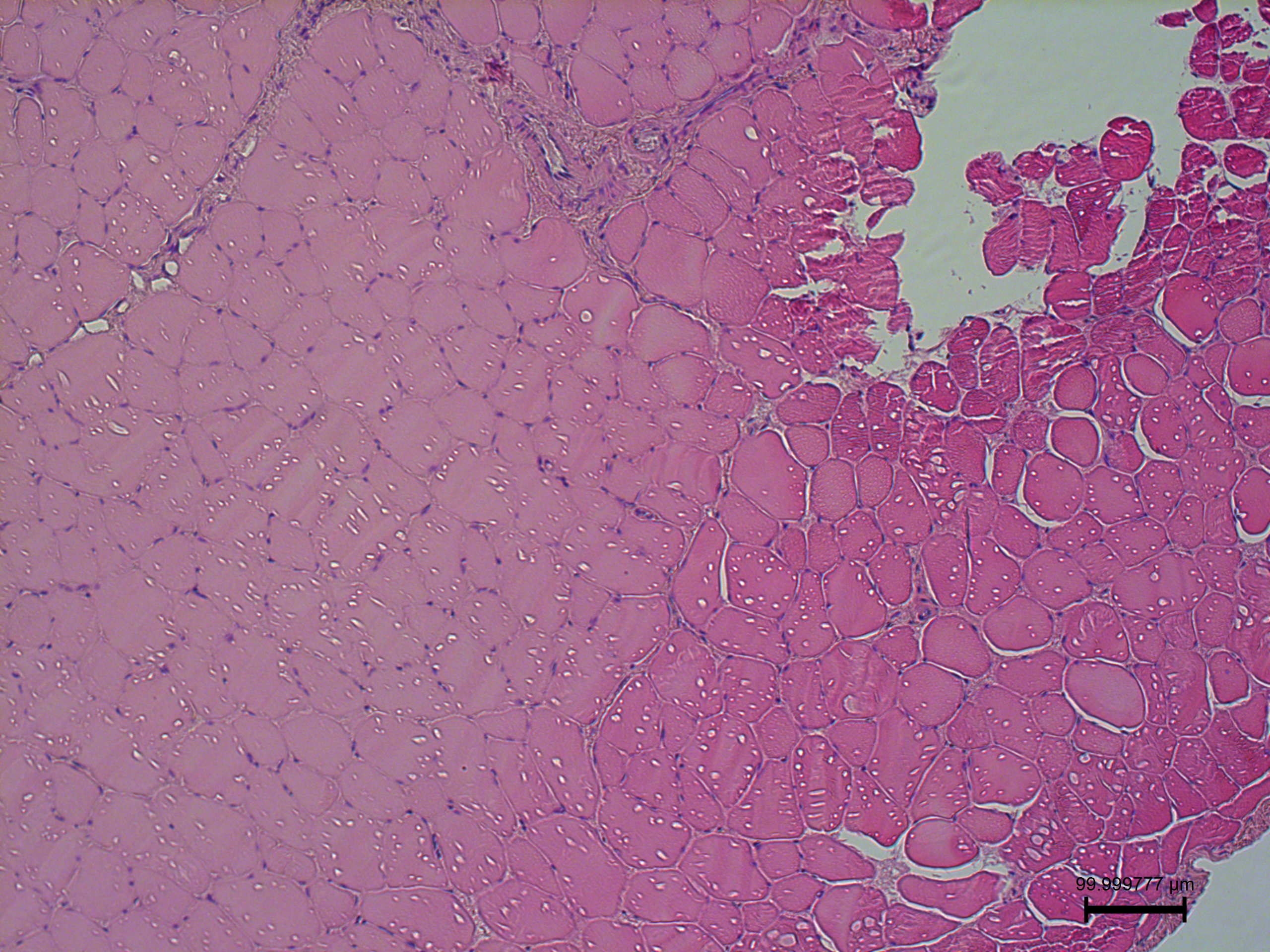

Supplement: Supplementary file 8 — Source data Fig. 6 [file 44321_2025_234_MOESM8_ESM.zip › Figure 6E/Vehicle/Figure/con0011.tif]

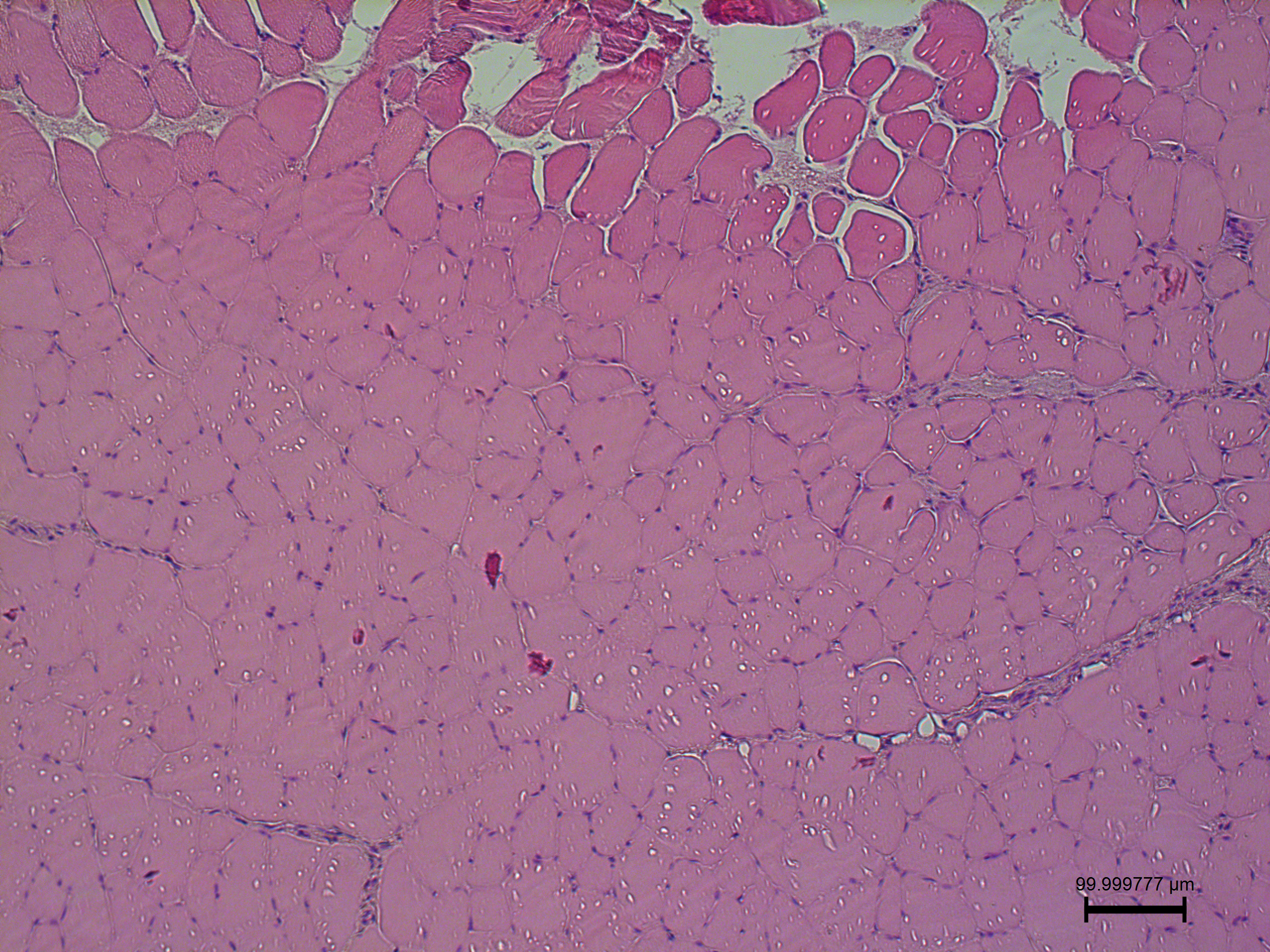

Supplement: Supplementary file 8 — Source data Fig. 6 [file 44321_2025_234_MOESM8_ESM.zip › Figure 6E/Vehicle/Figure/con0014.tif]

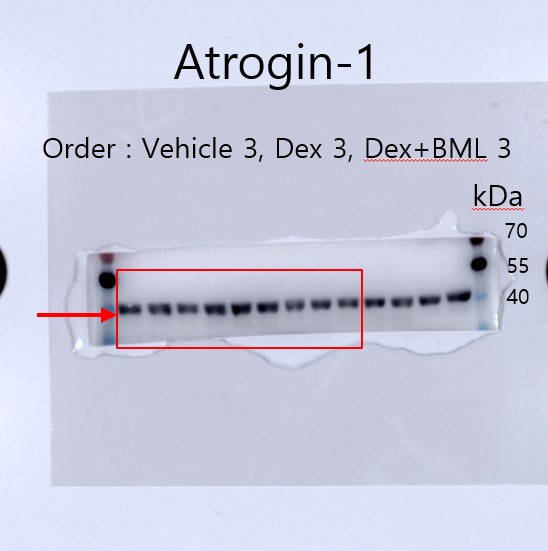

Supplement: Supplementary file 8 — Source data Fig. 6 [file 44321_2025_234_MOESM8_ESM.zip › Figure 6I/Figure 6I Atrogin-1.jpg]

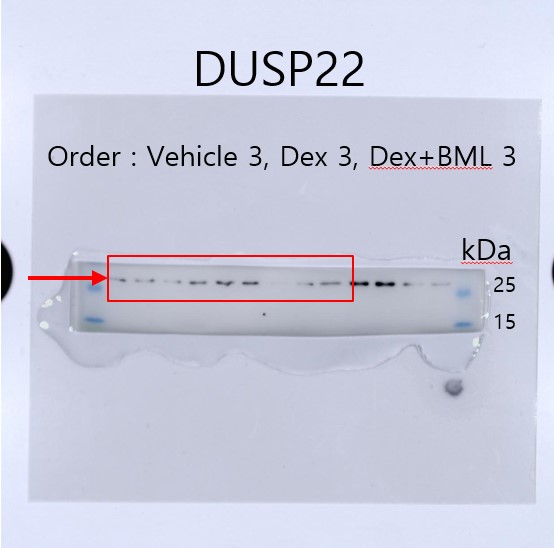

Supplement: Supplementary file 8 — Source data Fig. 6 [file 44321_2025_234_MOESM8_ESM.zip › Figure 6I/Figure 6I DUSP22.jpg]

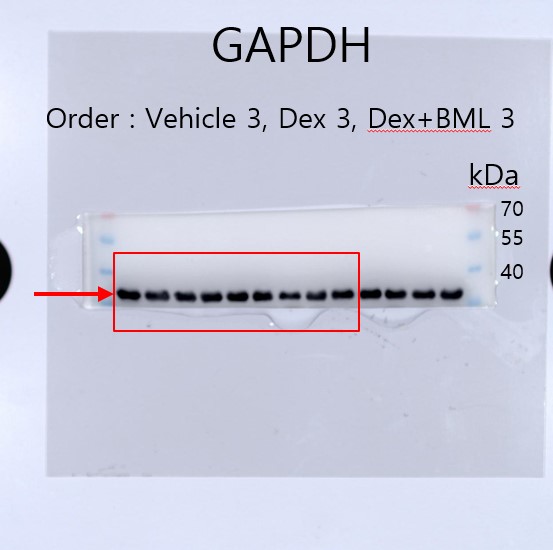

Supplement: Supplementary file 8 — Source data Fig. 6 [file 44321_2025_234_MOESM8_ESM.zip › Figure 6I/Figure 6I GAPDH.jpg]

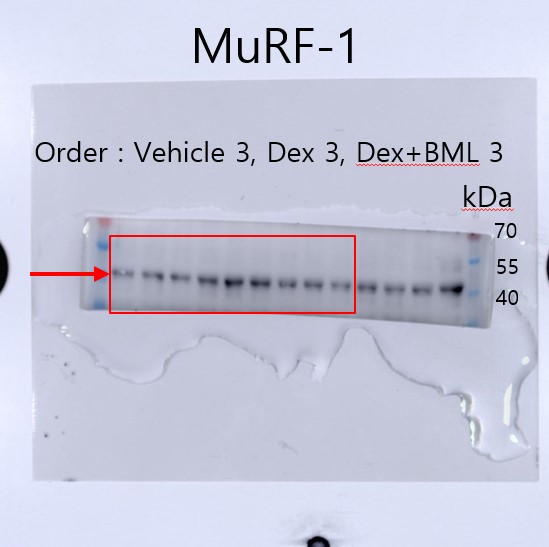

Supplement: Supplementary file 8 — Source data Fig. 6 [file 44321_2025_234_MOESM8_ESM.zip › Figure 6I/Figure 6I MuRF-1.jpg]

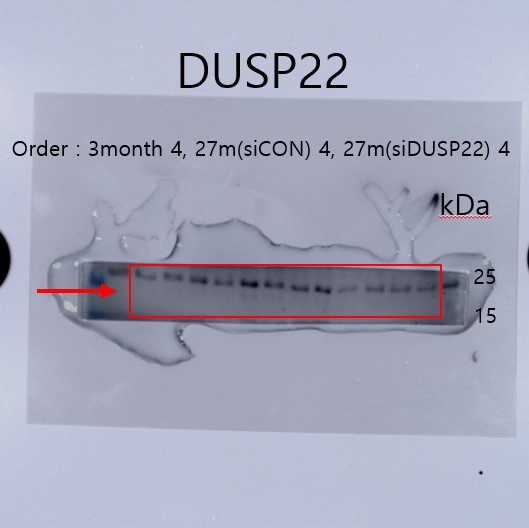

Supplement: Supplementary file 9 — Source data Fig. 7 [file 44321_2025_234_MOESM9_ESM.zip › Figure 7B/Figure 7B DUSP22.jpg]

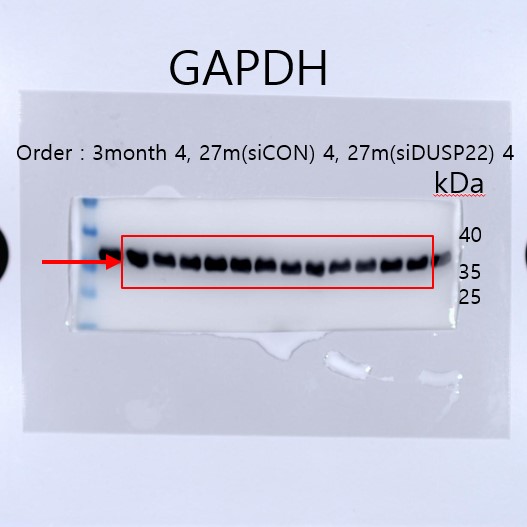

Supplement: Supplementary file 9 — Source data Fig. 7 [file 44321_2025_234_MOESM9_ESM.zip › Figure 7B/Figure 7B GAPDH.jpg]

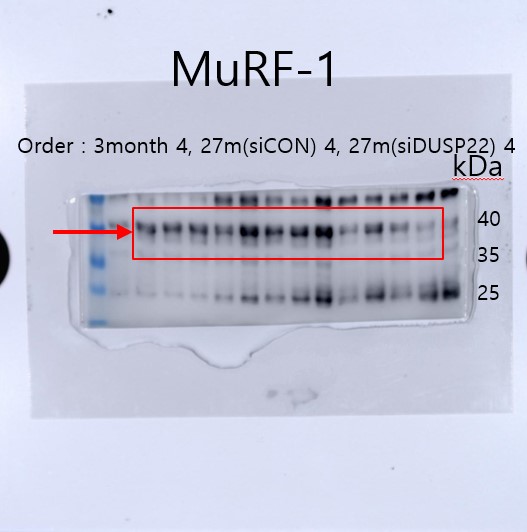

Supplement: Supplementary file 9 — Source data Fig. 7 [file 44321_2025_234_MOESM9_ESM.zip › Figure 7B/Figure 7B MuRF-1.jpg]

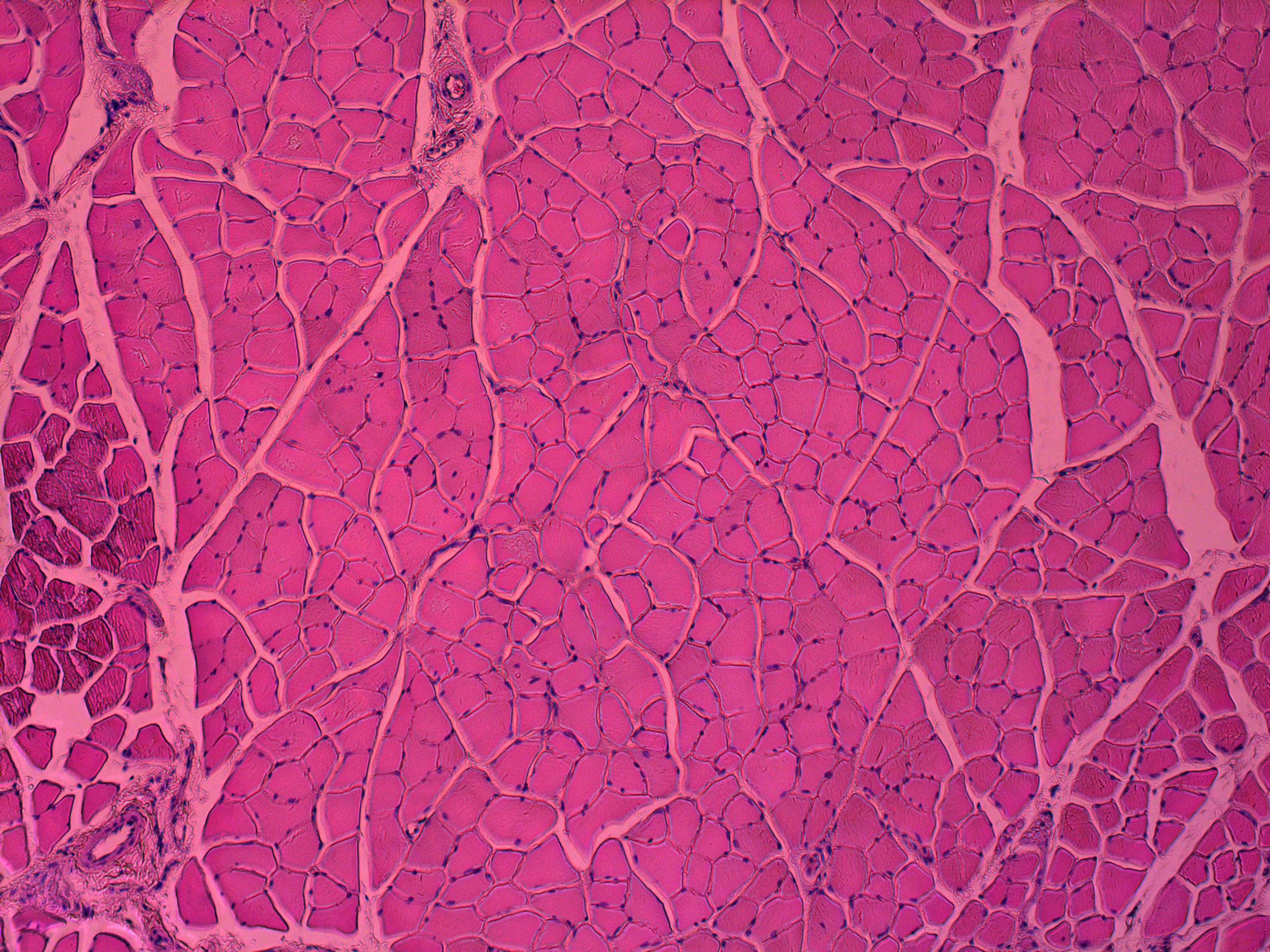

Supplement: Supplementary file 9 — Source data Fig. 7 [file 44321_2025_234_MOESM9_ESM.zip › Figure 7E/siCON1-1.tif]

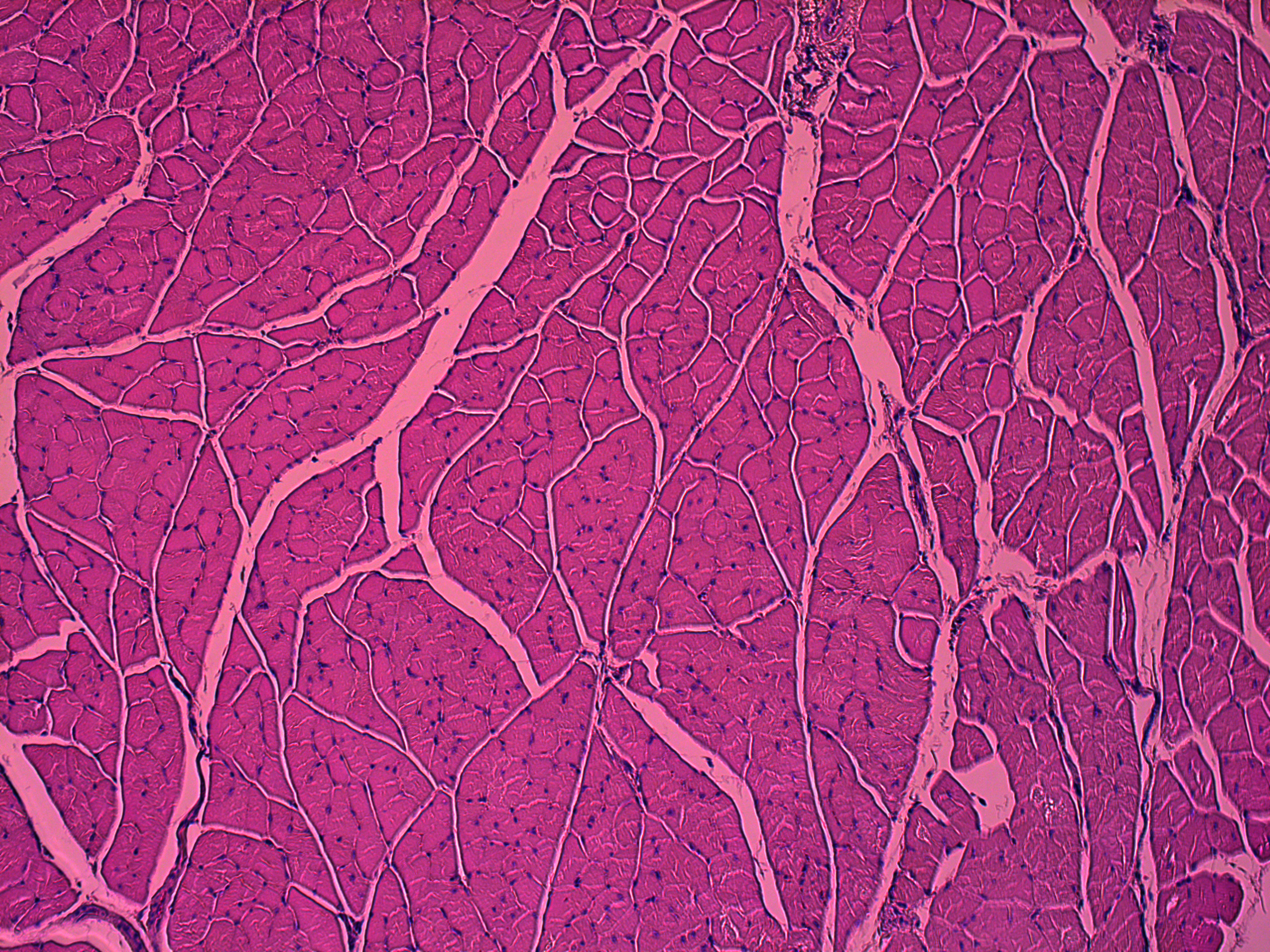

Supplement: Supplementary file 9 — Source data Fig. 7 [file 44321_2025_234_MOESM9_ESM.zip › Figure 7E/siCON2-1.tif]

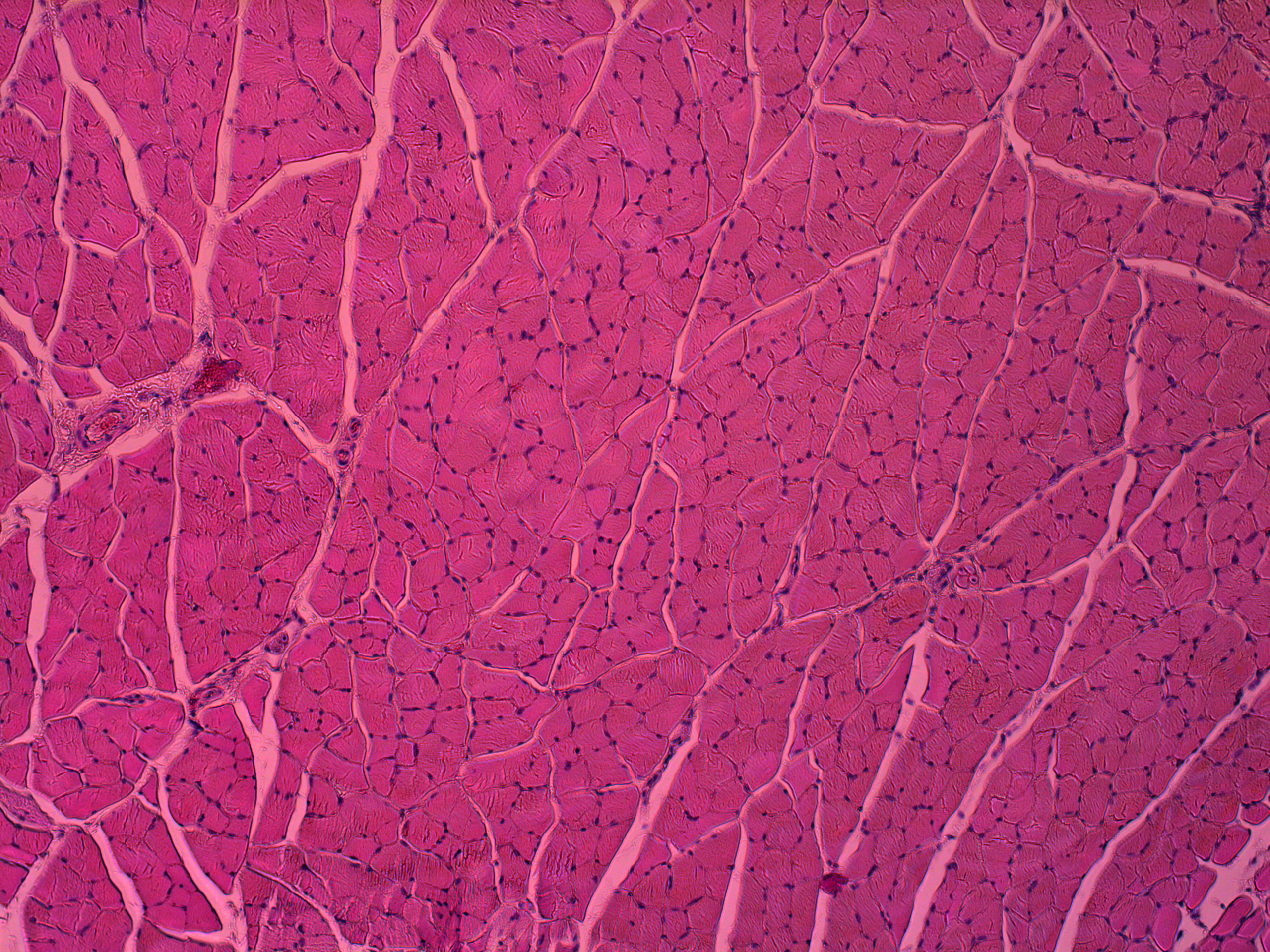

Supplement: Supplementary file 9 — Source data Fig. 7 [file 44321_2025_234_MOESM9_ESM.zip › Figure 7E/siCON3-1.tif]

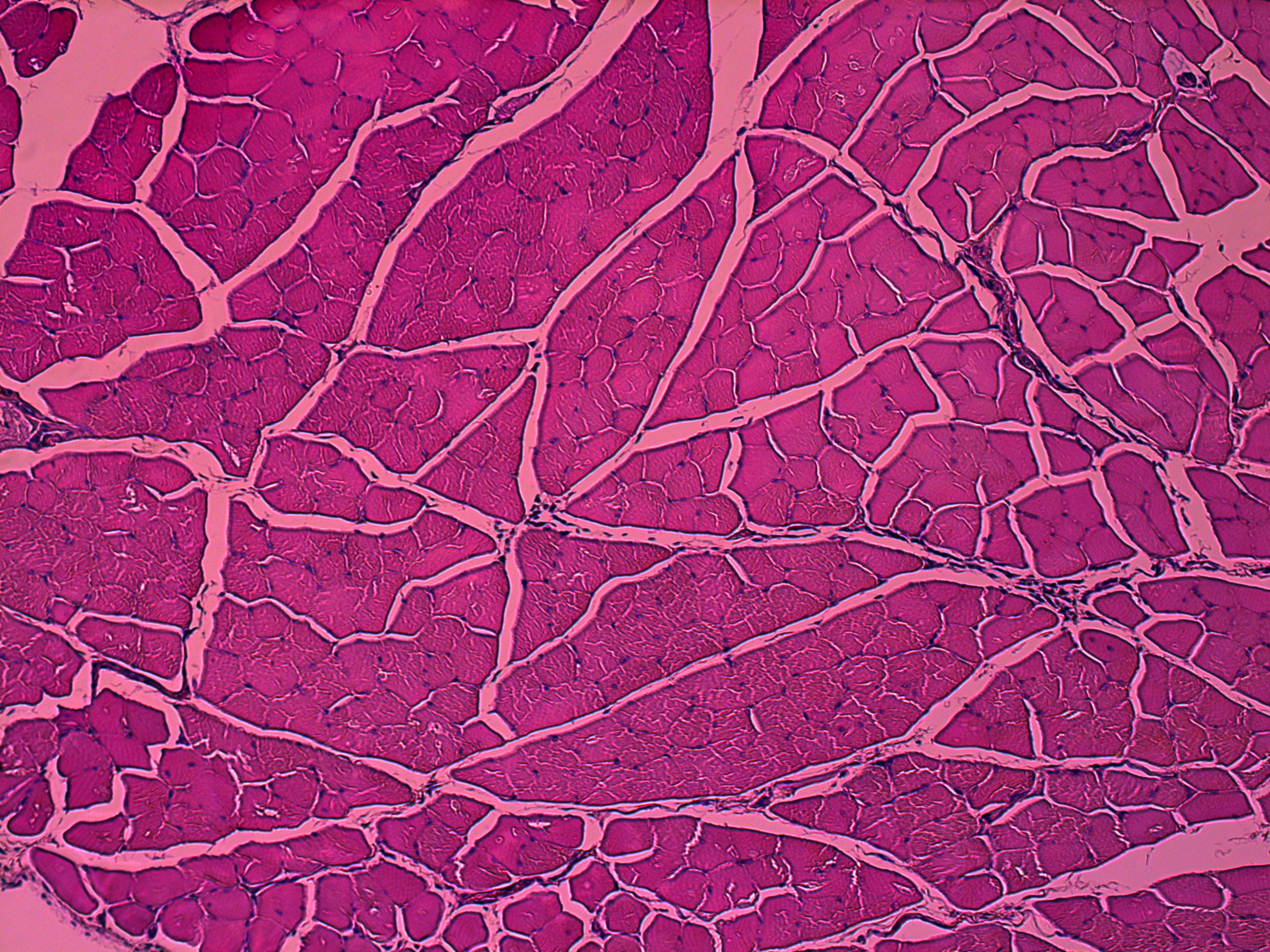

Supplement: Supplementary file 9 — Source data Fig. 7 [file 44321_2025_234_MOESM9_ESM.zip › Figure 7E/siCON4-1.tif]

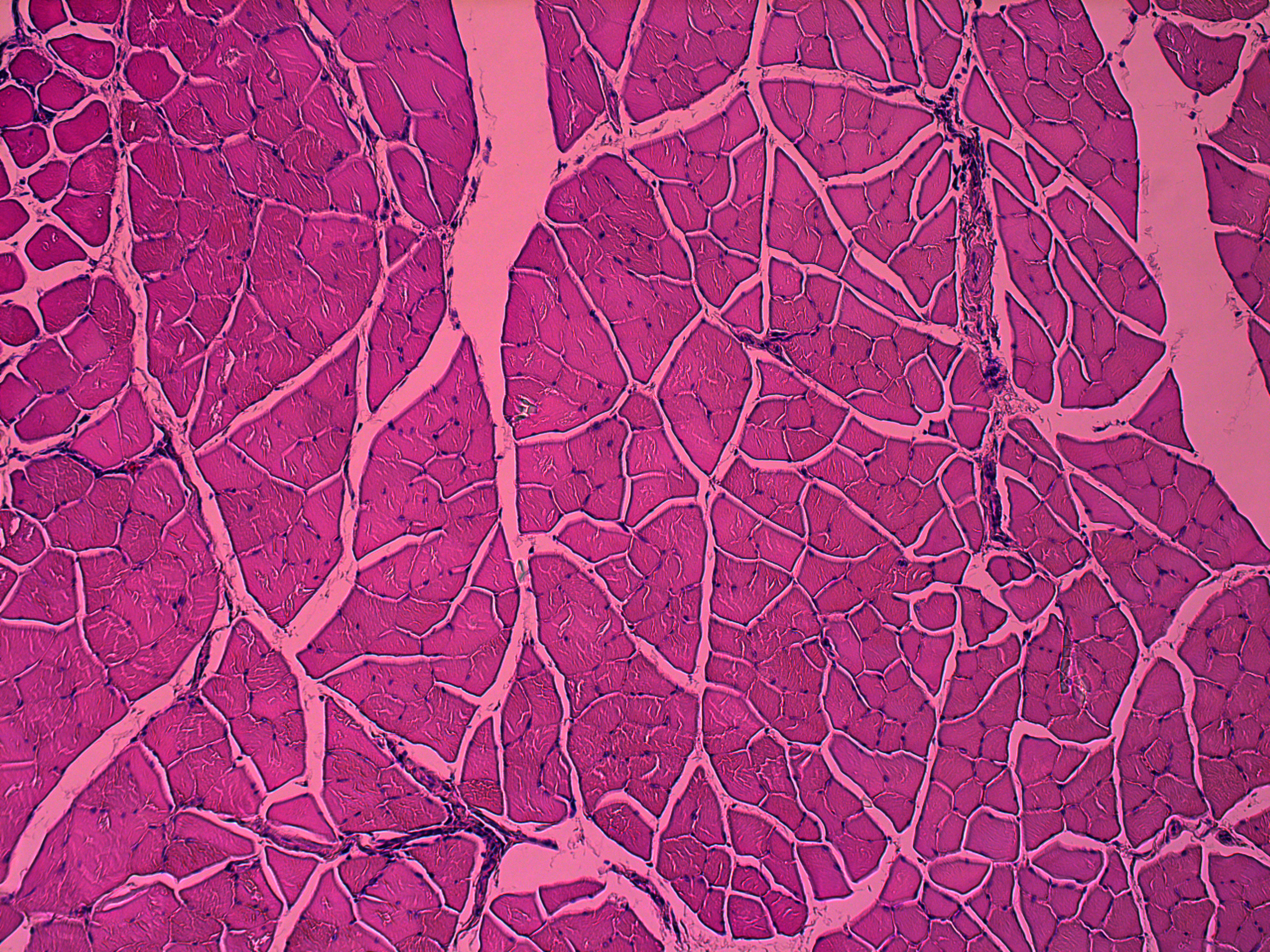

Supplement: Supplementary file 9 — Source data Fig. 7 [file 44321_2025_234_MOESM9_ESM.zip › Figure 7E/siCON5-1.tif]

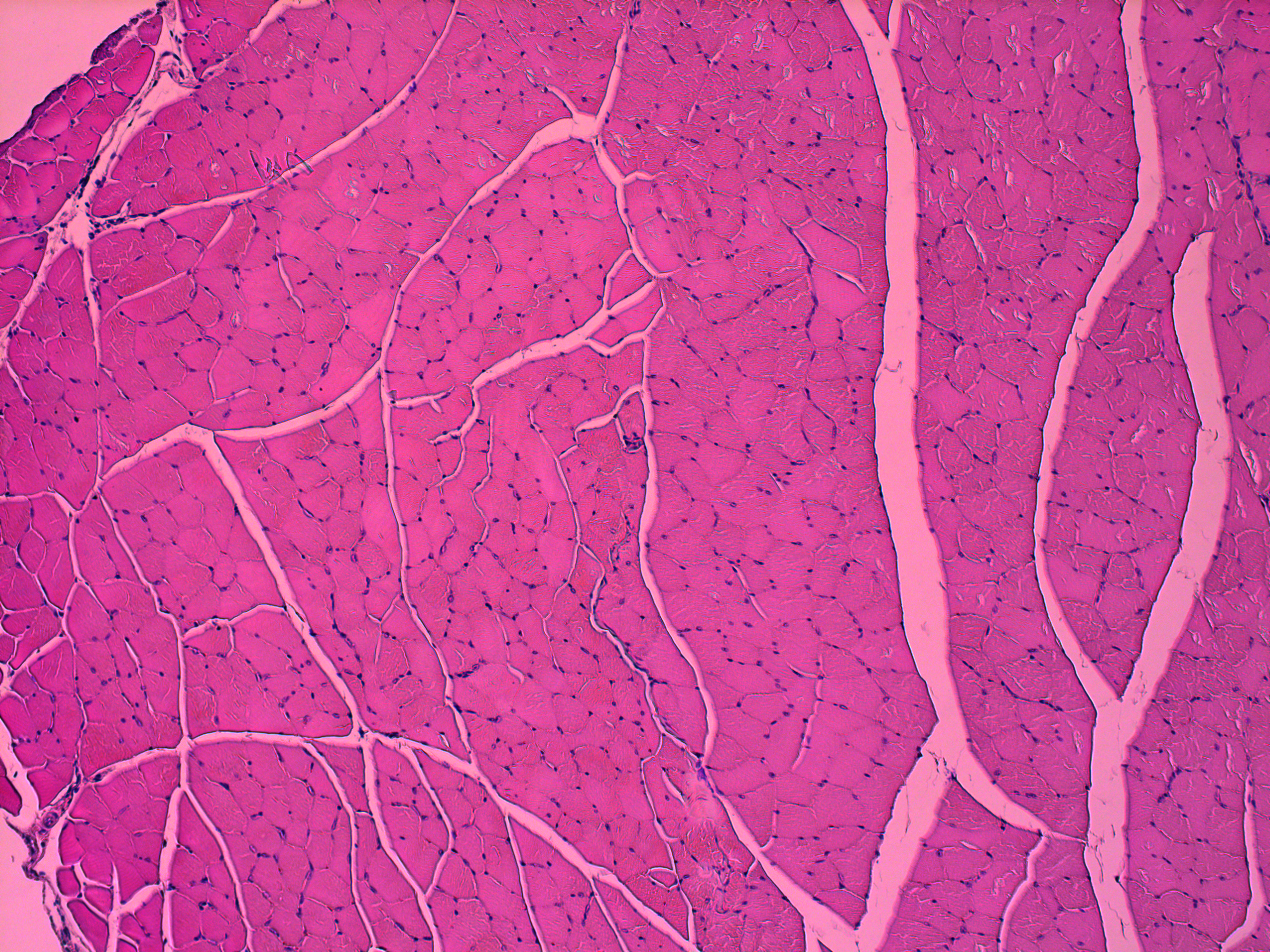

Supplement: Supplementary file 9 — Source data Fig. 7 [file 44321_2025_234_MOESM9_ESM.zip › Figure 7E/siDUSP22 1-1.tif]

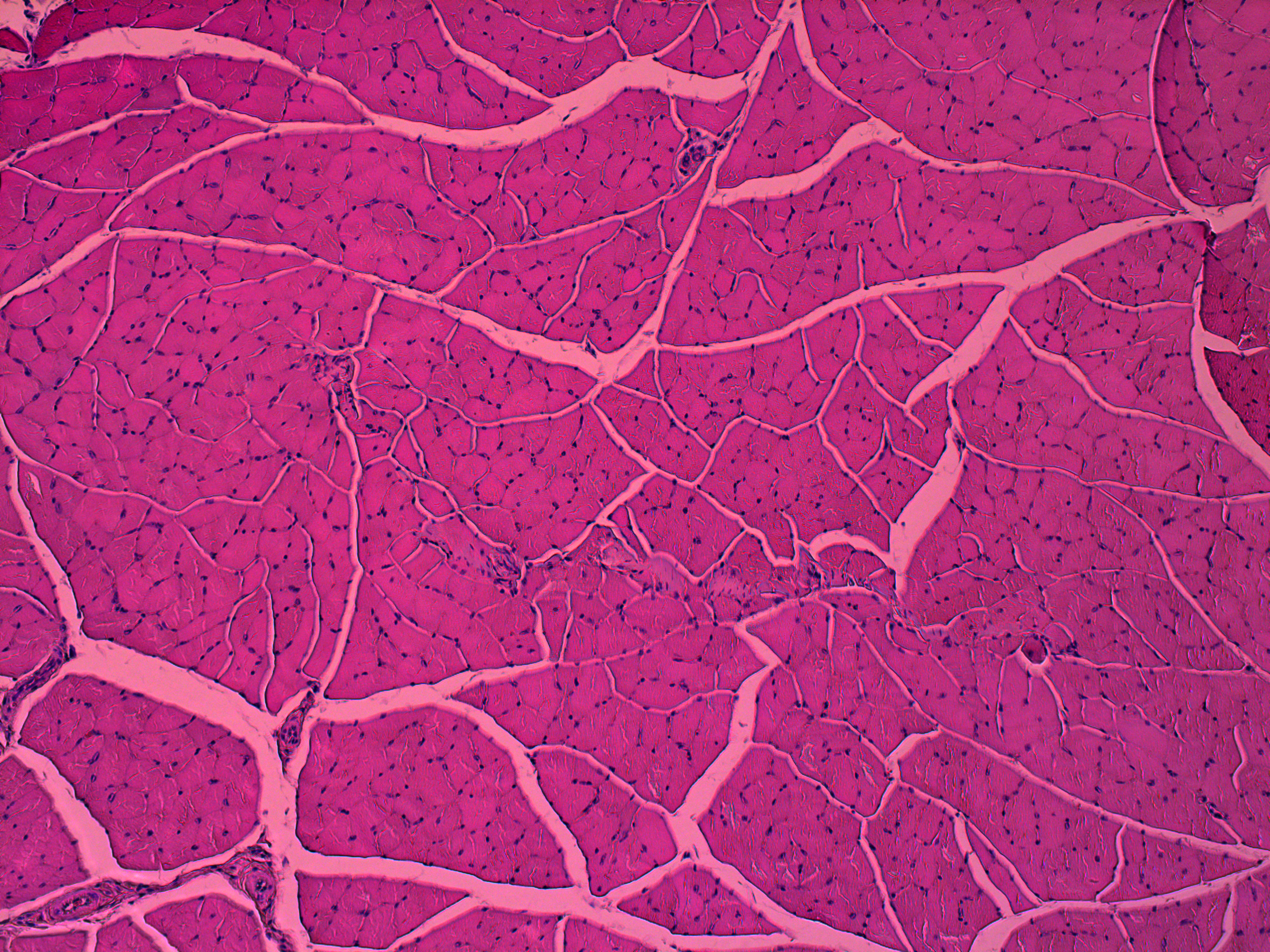

Supplement: Supplementary file 9 — Source data Fig. 7 [file 44321_2025_234_MOESM9_ESM.zip › Figure 7E/siDUSP22 2-1.tif]

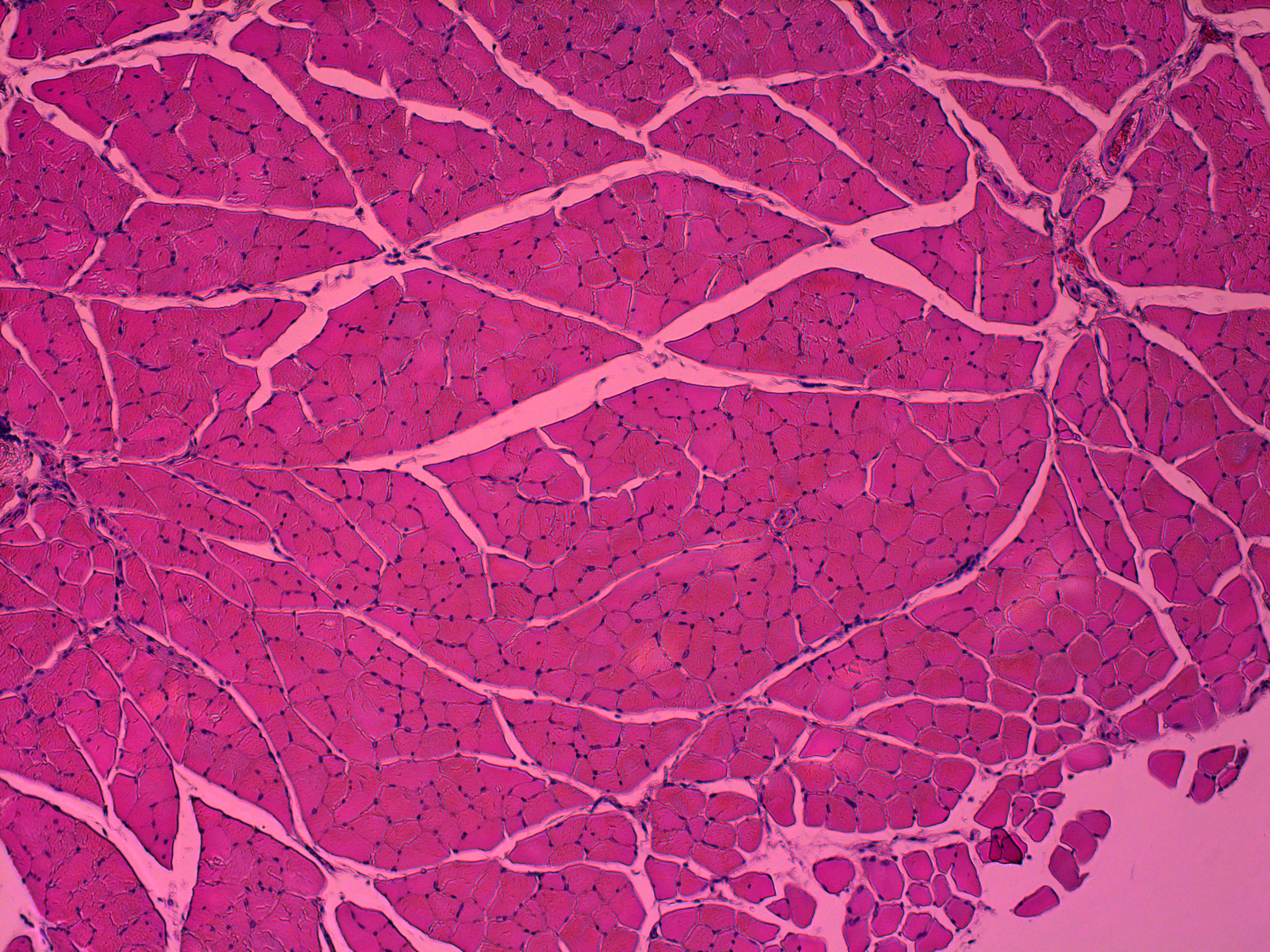

Supplement: Supplementary file 9 — Source data Fig. 7 [file 44321_2025_234_MOESM9_ESM.zip › Figure 7E/siDUSP22 3-1.tif]

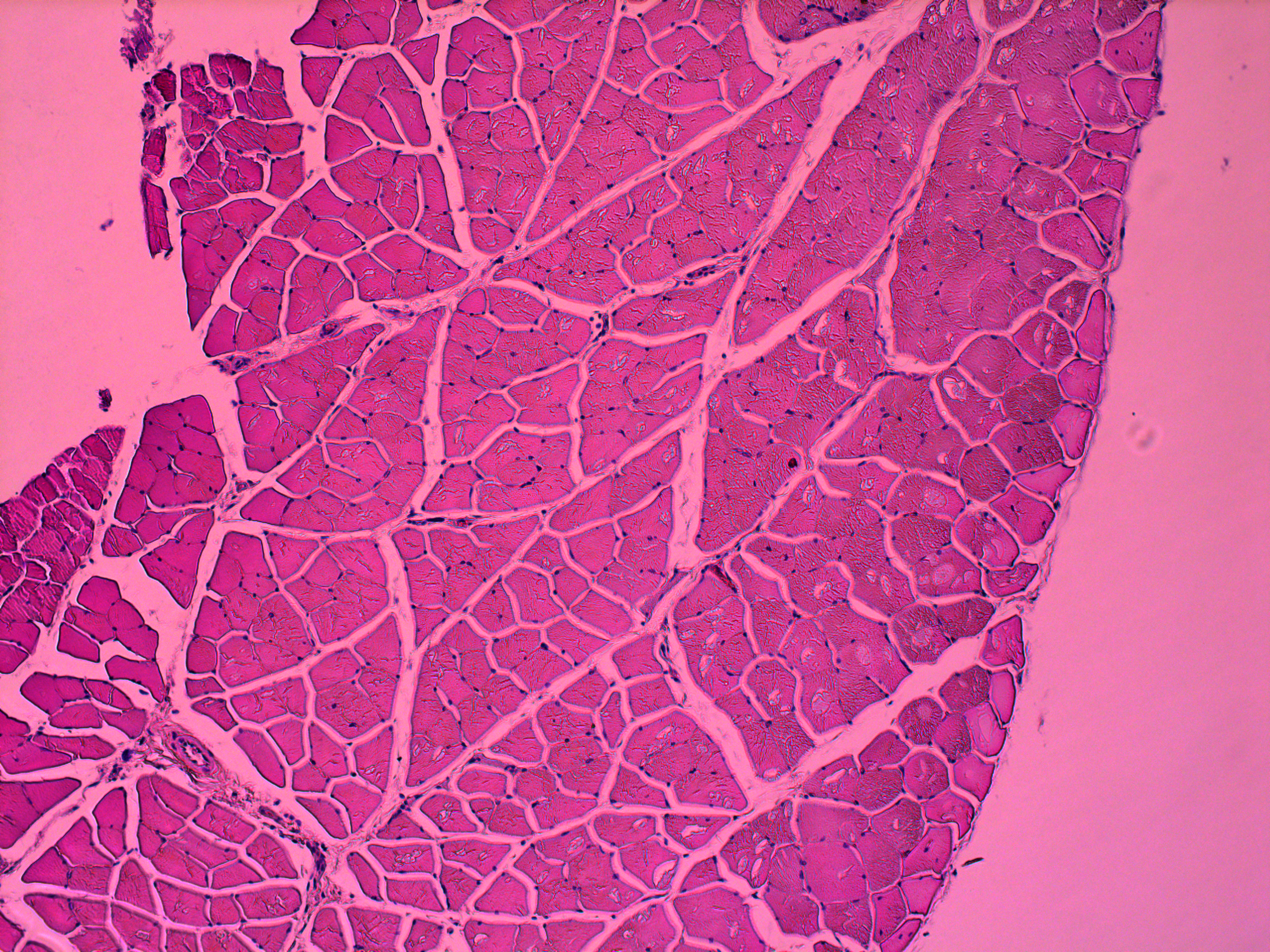

Supplement: Supplementary file 9 — Source data Fig. 7 [file 44321_2025_234_MOESM9_ESM.zip › Figure 7E/siDUSP22 4-1.tif]

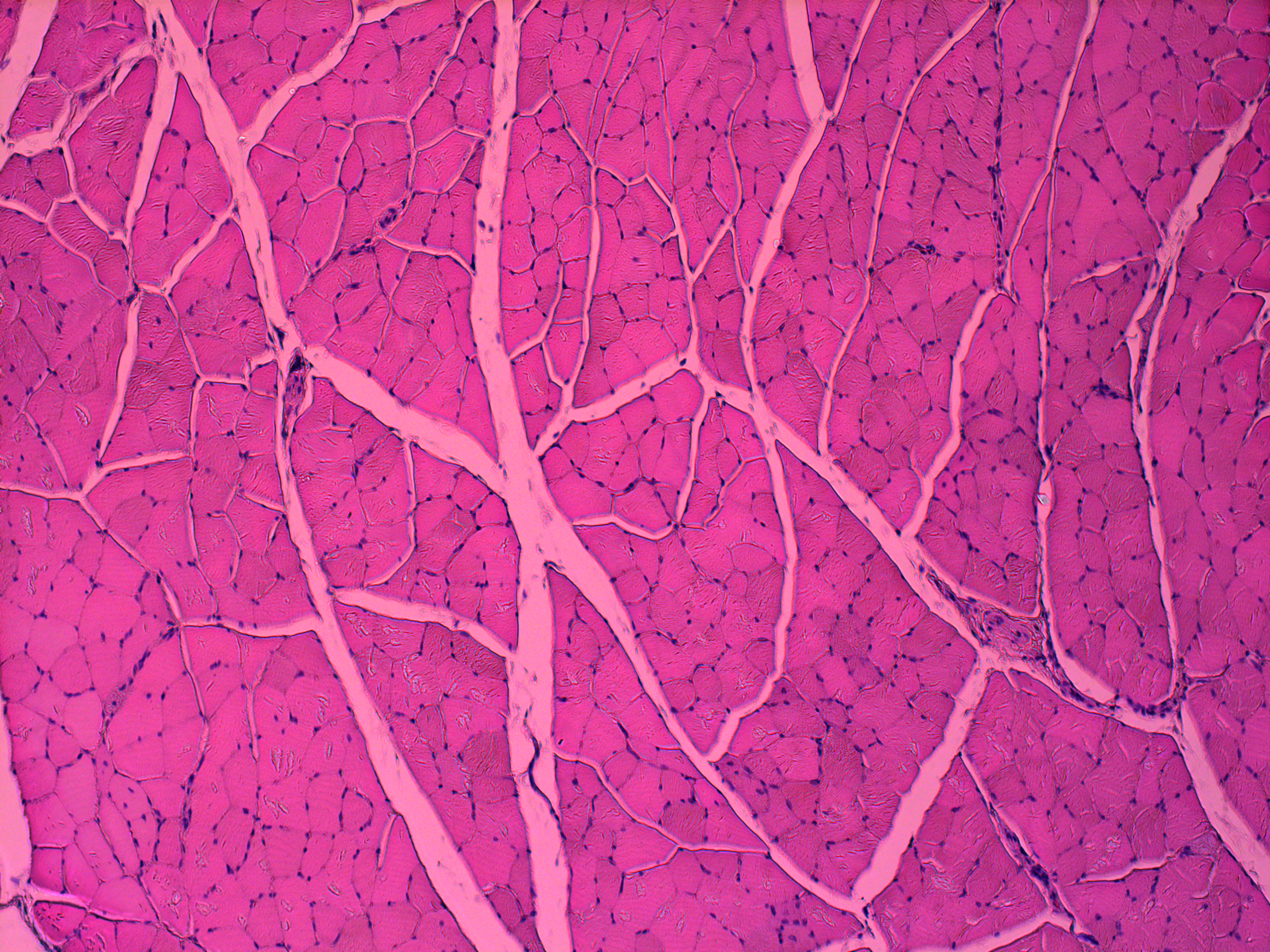

Supplement: Supplementary file 9 — Source data Fig. 7 [file 44321_2025_234_MOESM9_ESM.zip › Figure 7E/siDUSP22 5-1.tif]

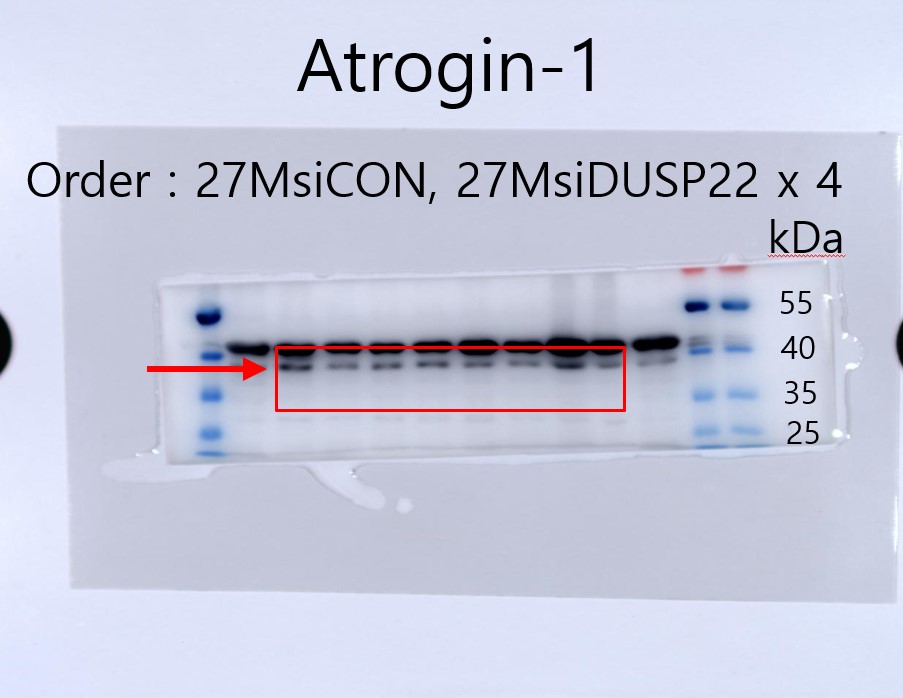

Supplement: Supplementary file 9 — Source data Fig. 7 [file 44321_2025_234_MOESM9_ESM.zip › Figure 7G/Figure 7G Atrogin-1.jpg]

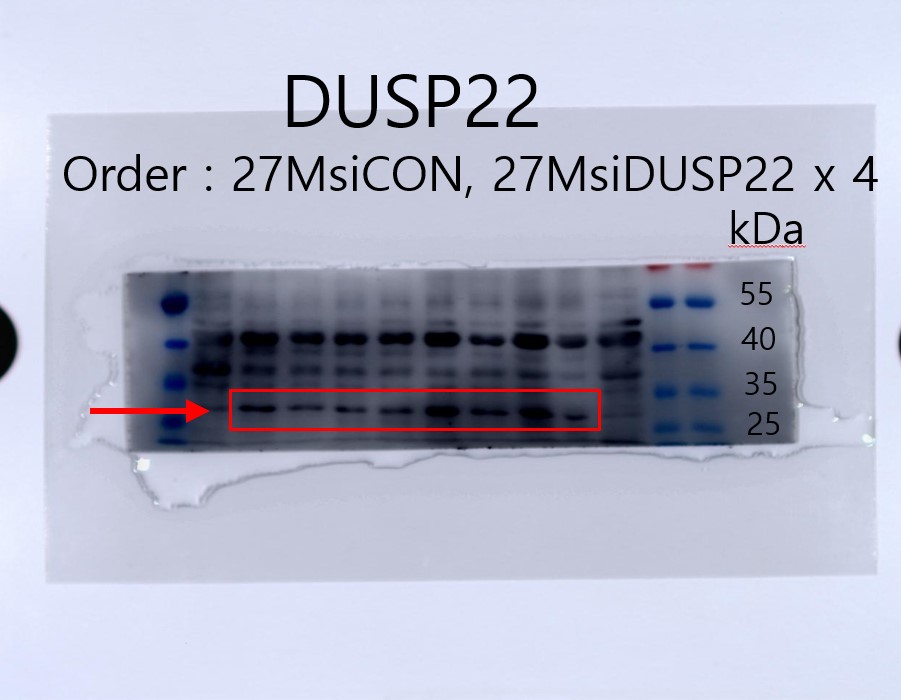

Supplement: Supplementary file 9 — Source data Fig. 7 [file 44321_2025_234_MOESM9_ESM.zip › Figure 7G/Figure 7G DUSP22.jpg]

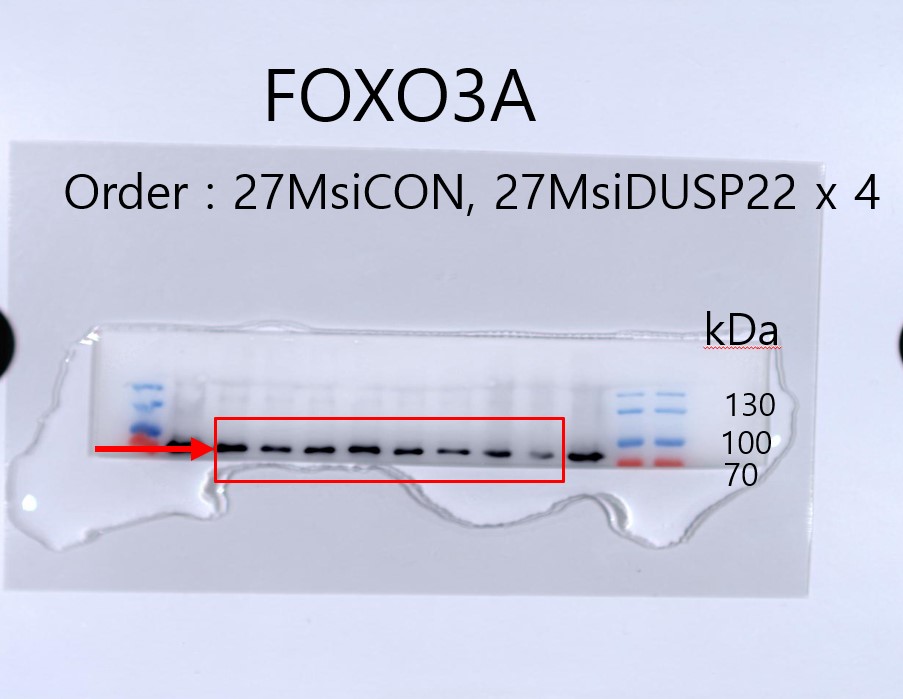

Supplement: Supplementary file 9 — Source data Fig. 7 [file 44321_2025_234_MOESM9_ESM.zip › Figure 7G/Figure 7G FOXO3a.jpg]

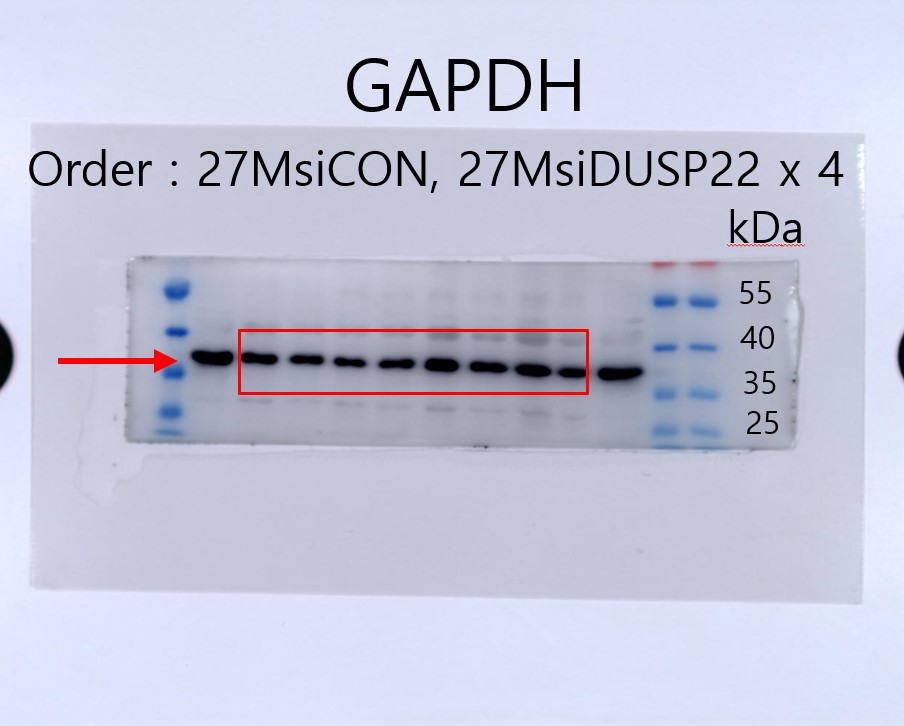

Supplement: Supplementary file 9 — Source data Fig. 7 [file 44321_2025_234_MOESM9_ESM.zip › Figure 7G/Figure 7G GAPDH.jpg]

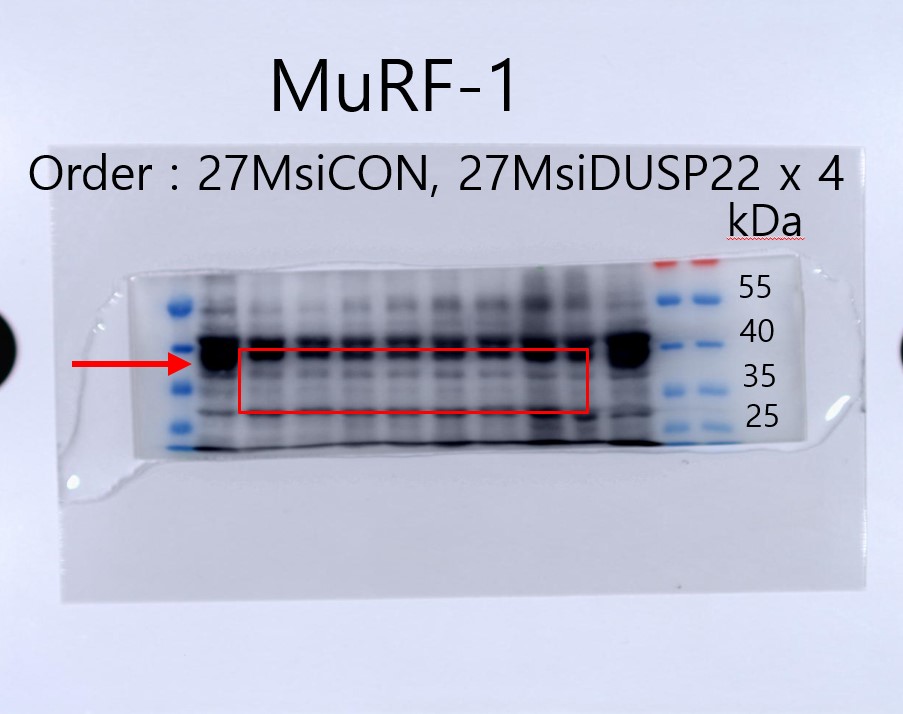

Supplement: Supplementary file 9 — Source data Fig. 7 [file 44321_2025_234_MOESM9_ESM.zip › Figure 7G/Figure 7G MuRF-1.jpg]

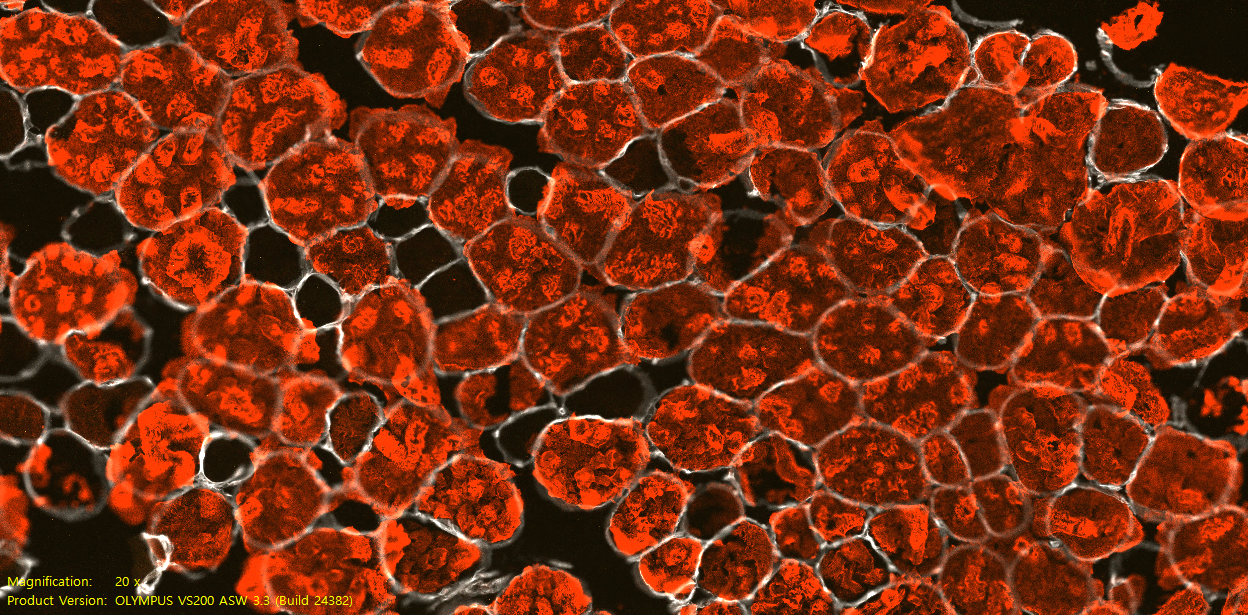

Supplement: Supplementary file 9 — Source data Fig. 7 [file 44321_2025_234_MOESM9_ESM.zip › Figure 7O/5months/5M 1.tif]

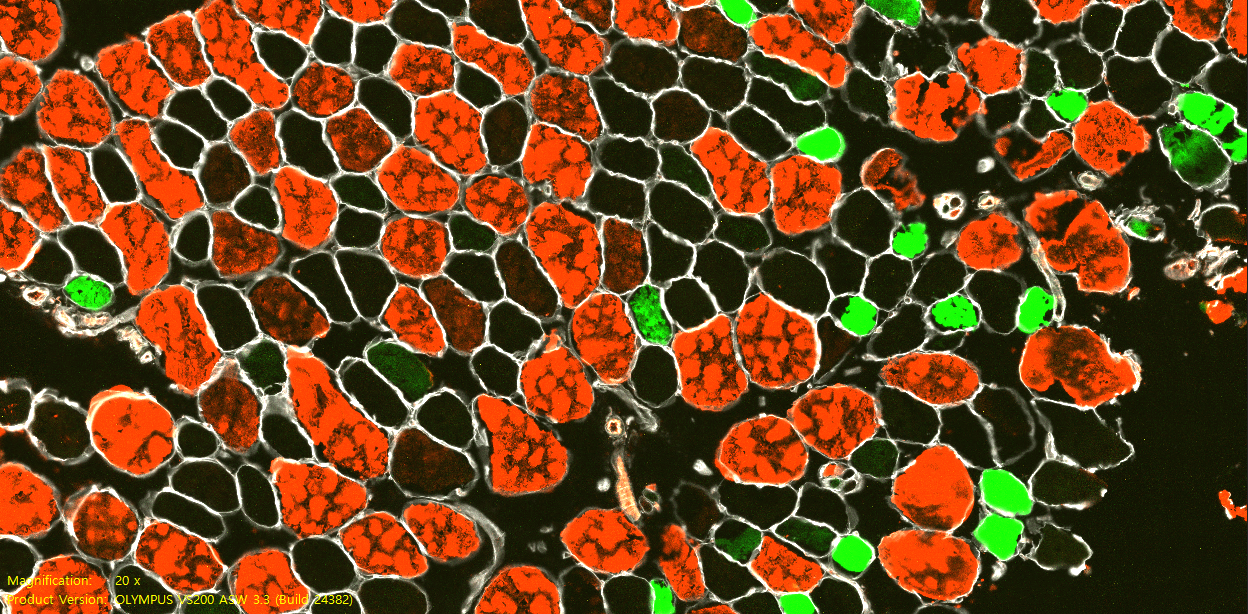

Supplement: Supplementary file 9 — Source data Fig. 7 [file 44321_2025_234_MOESM9_ESM.zip › Figure 7O/5months/5M 2.tif]

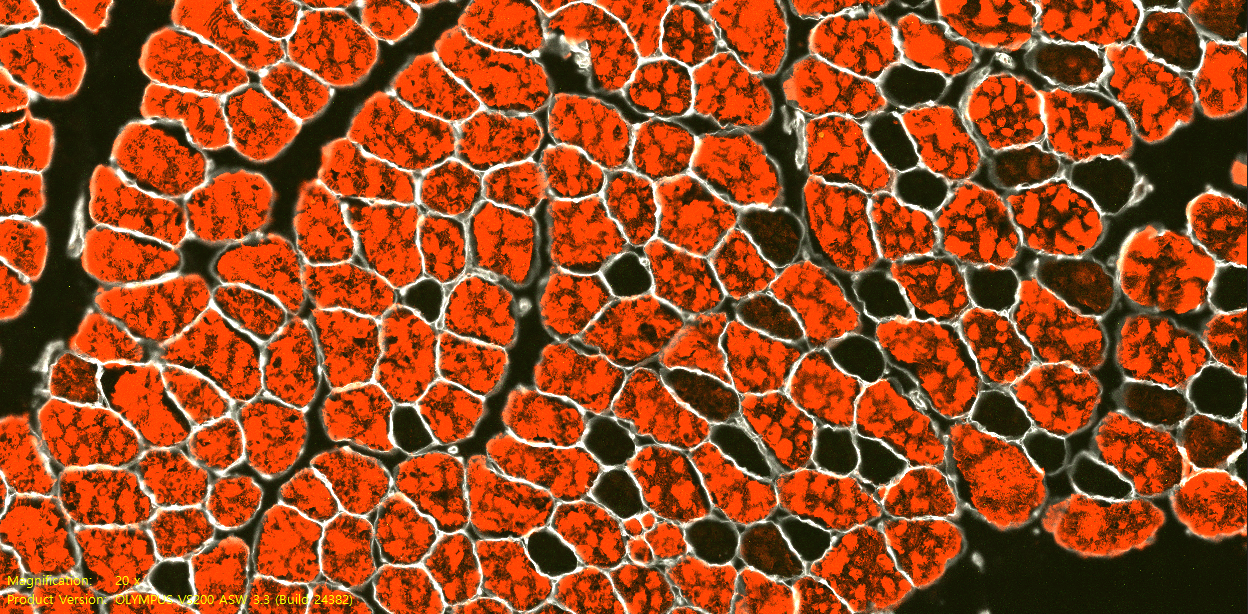

Supplement: Supplementary file 9 — Source data Fig. 7 [file 44321_2025_234_MOESM9_ESM.zip › Figure 7O/5months/5M 3.tif]

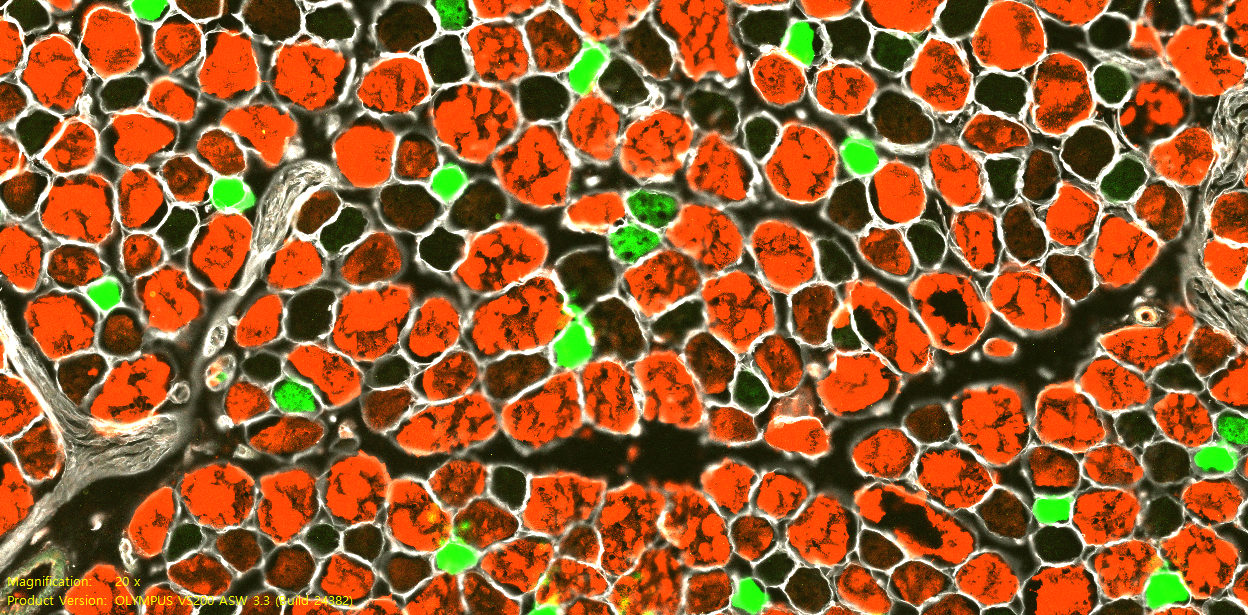

Supplement: Supplementary file 9 — Source data Fig. 7 [file 44321_2025_234_MOESM9_ESM.zip › Figure 7O/5months/5M 4.tif]

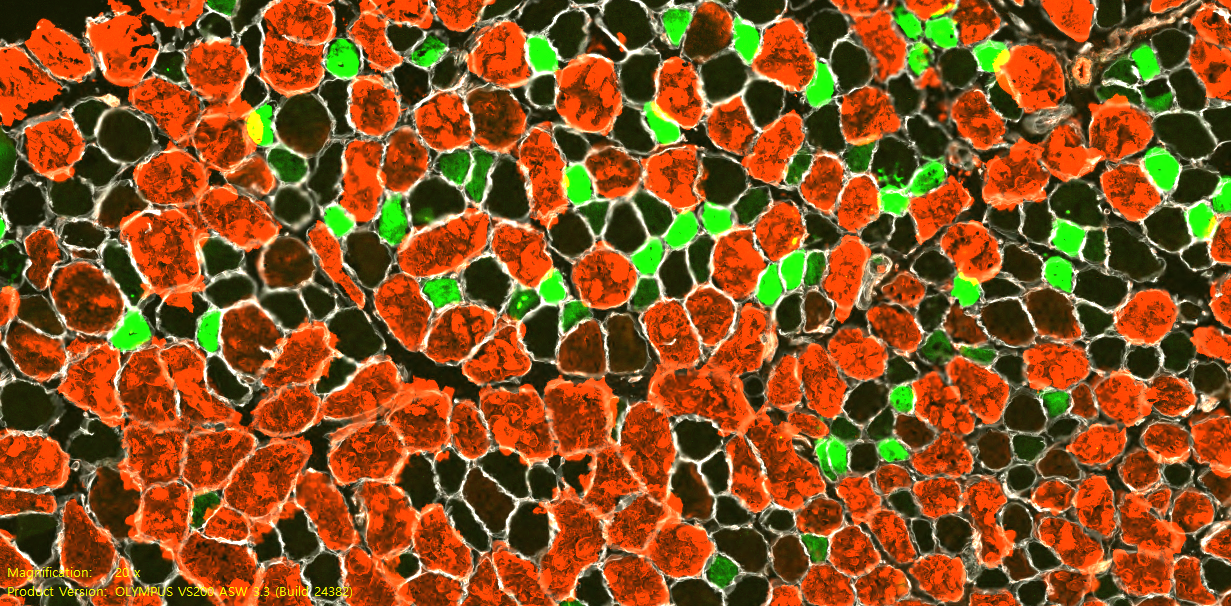

Supplement: Supplementary file 9 — Source data Fig. 7 [file 44321_2025_234_MOESM9_ESM.zip › Figure 7O/Aged/Aged 1.tif]

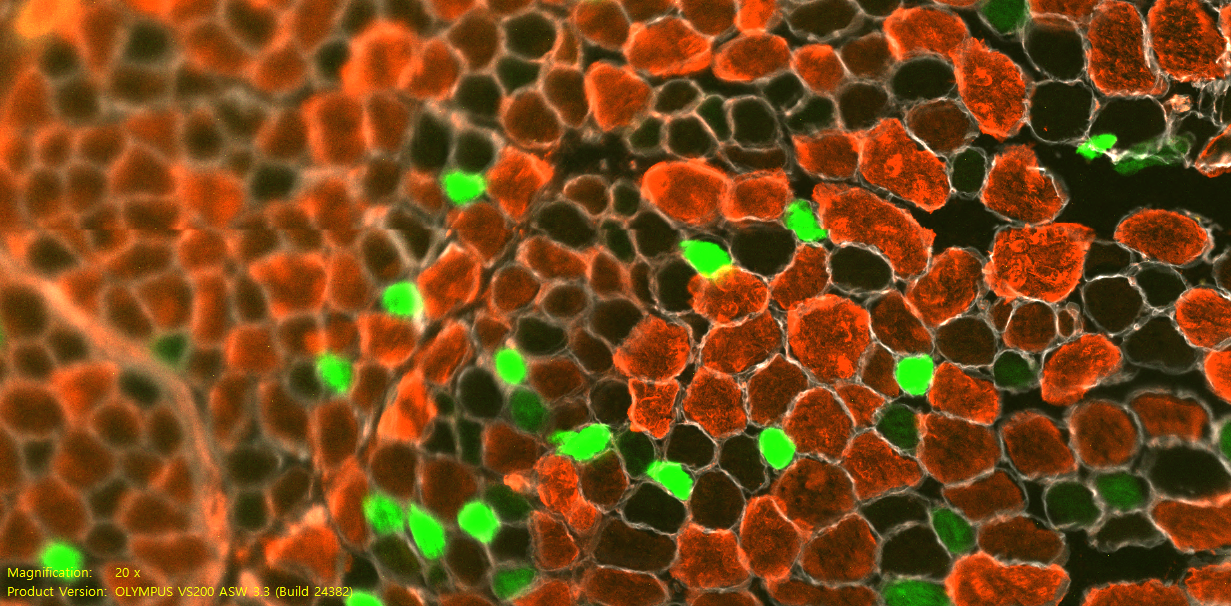

Supplement: Supplementary file 9 — Source data Fig. 7 [file 44321_2025_234_MOESM9_ESM.zip › Figure 7O/Aged/Aged 2.tif]

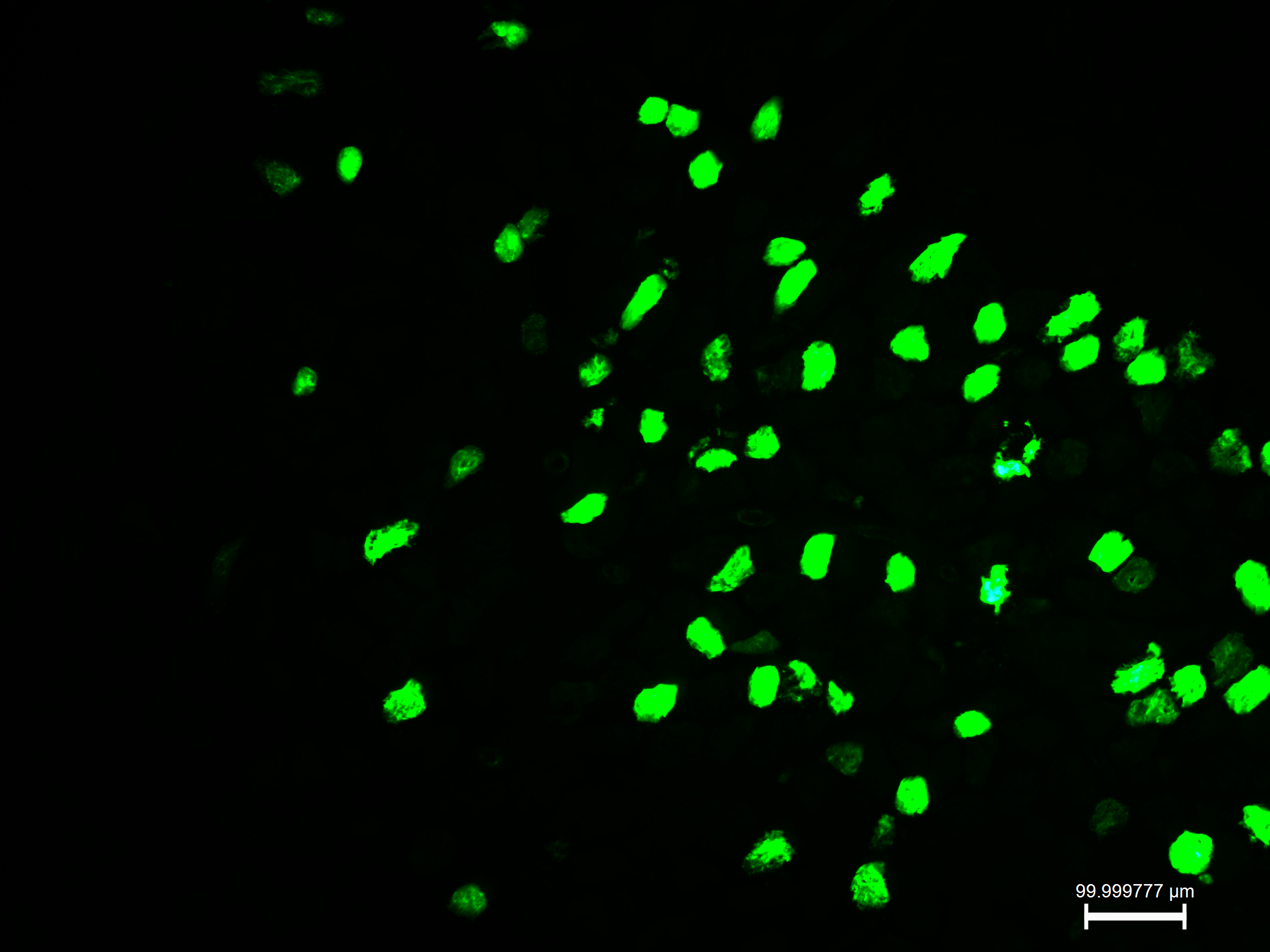

Supplement: Supplementary file 9 — Source data Fig. 7 [file 44321_2025_234_MOESM9_ESM.zip › Figure 7O/Aged/Aged 3 2A.tif]

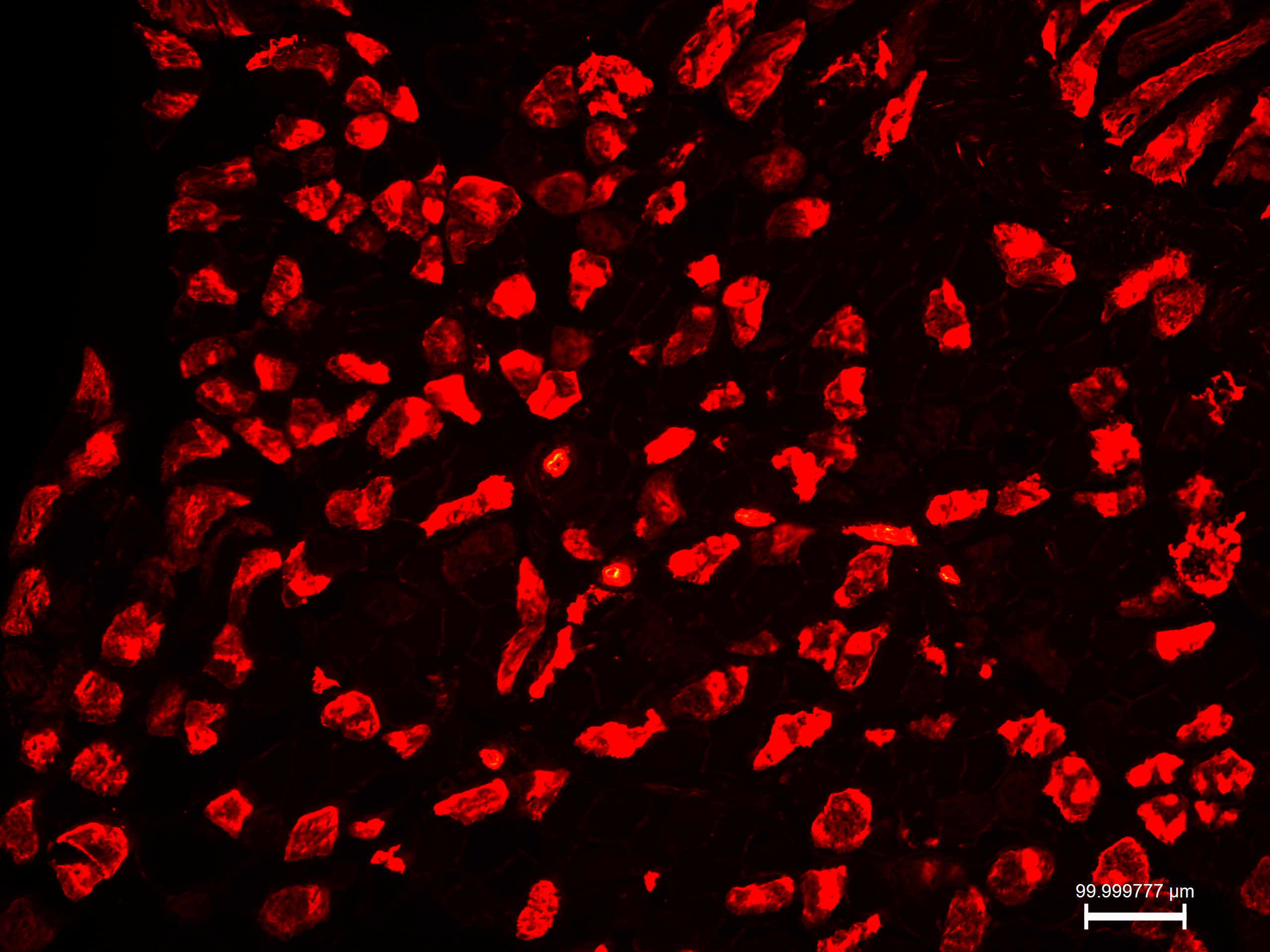

Supplement: Supplementary file 9 — Source data Fig. 7 [file 44321_2025_234_MOESM9_ESM.zip › Figure 7O/Aged/Aged 3 2B.tif]

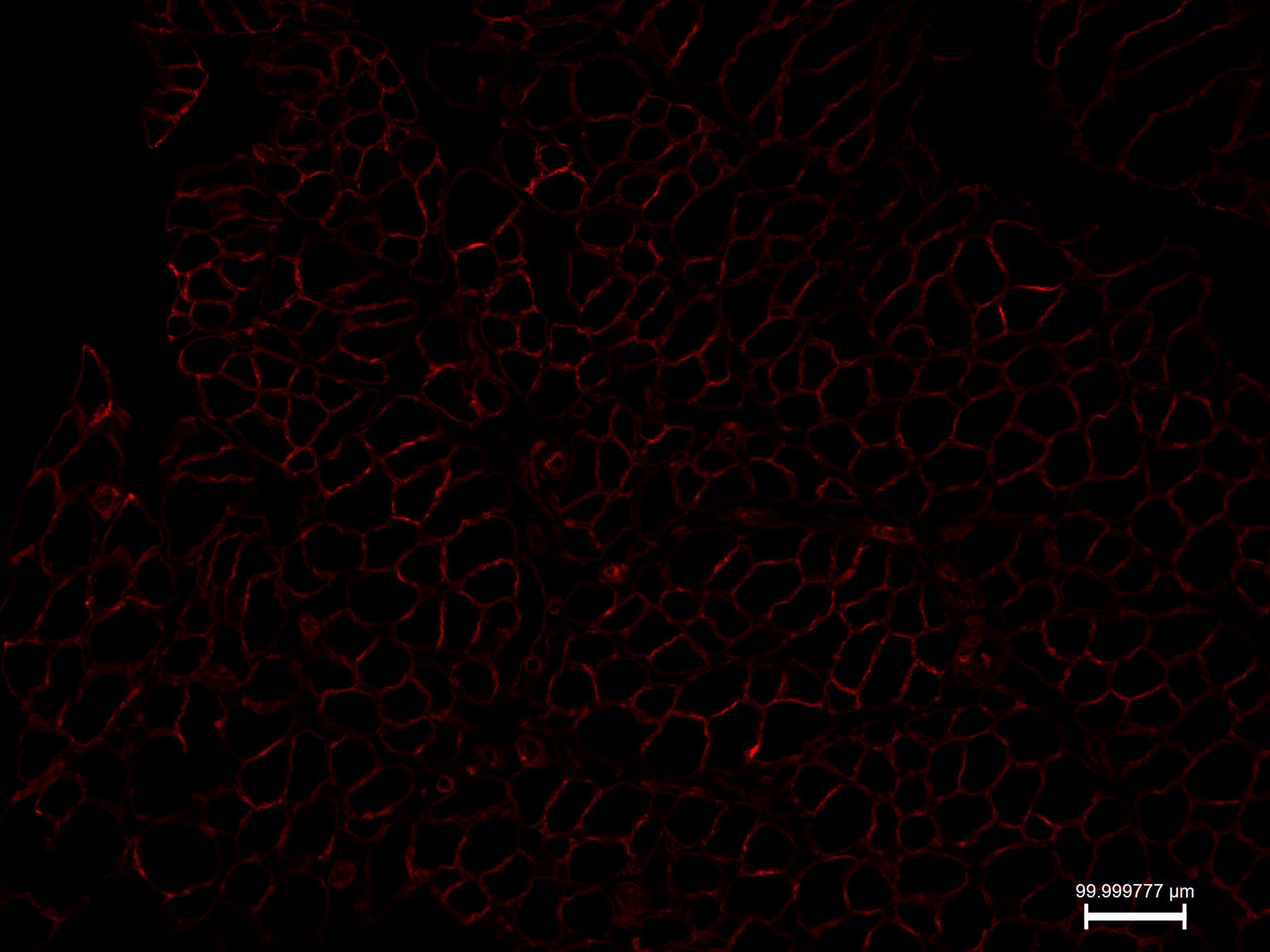

Supplement: Supplementary file 9 — Source data Fig. 7 [file 44321_2025_234_MOESM9_ESM.zip › Figure 7O/Aged/Aged 3 Laminin.tif]

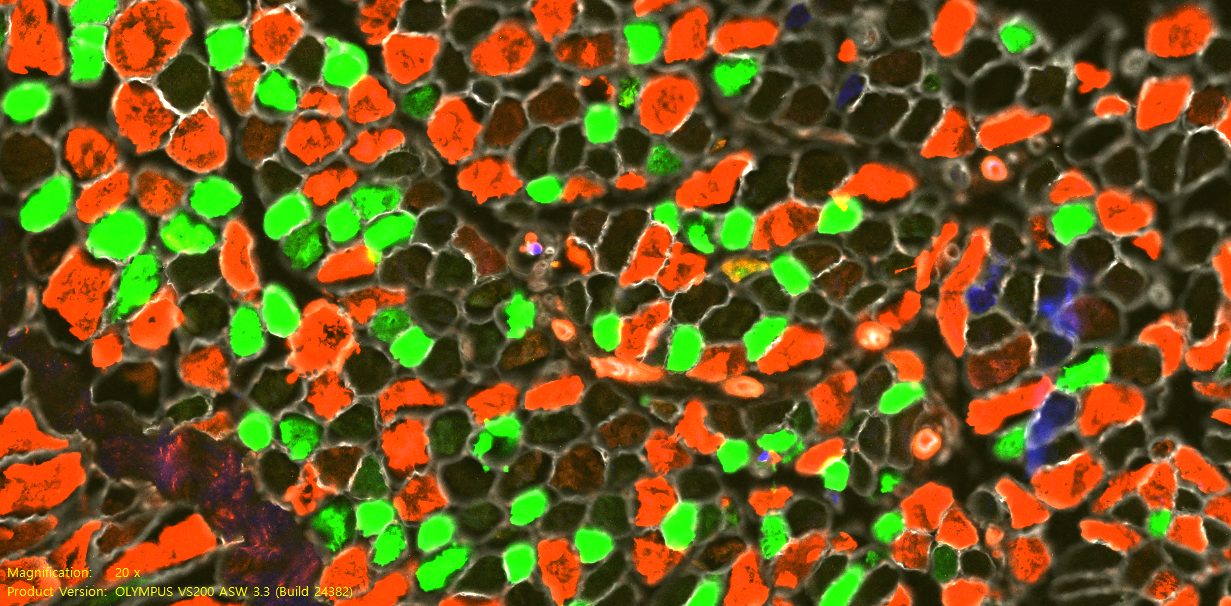

Supplement: Supplementary file 9 — Source data Fig. 7 [file 44321_2025_234_MOESM9_ESM.zip › Figure 7O/Aged/Aged 4.tif]

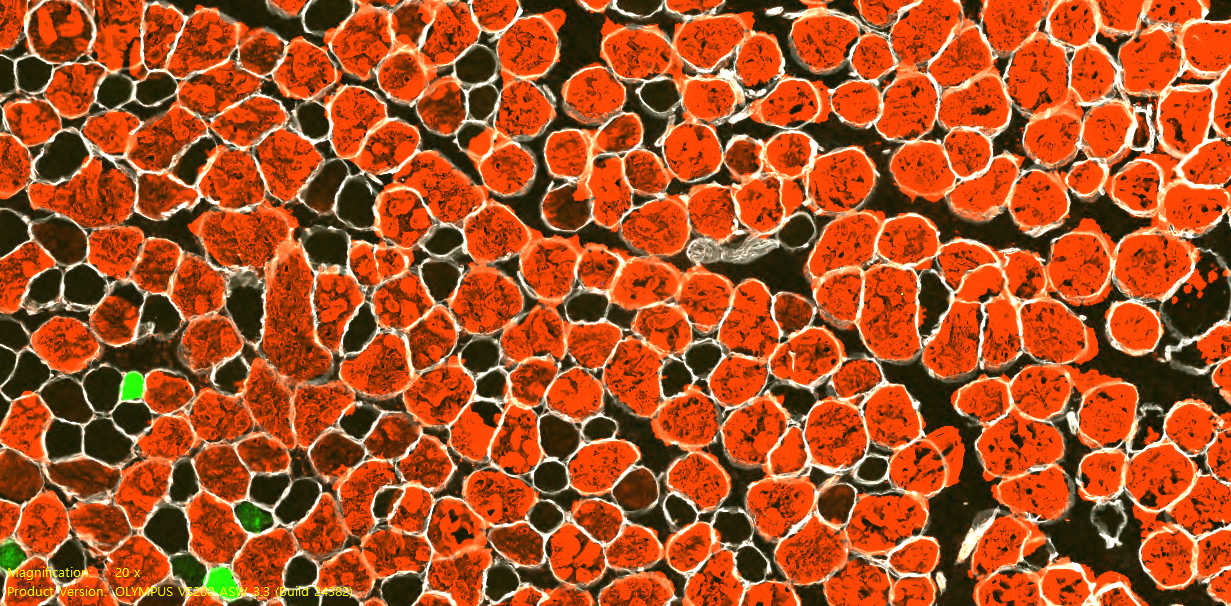

Supplement: Supplementary file 9 — Source data Fig. 7 [file 44321_2025_234_MOESM9_ESM.zip › Figure 7O/Aged+ BML260/BML-260 1.tif]

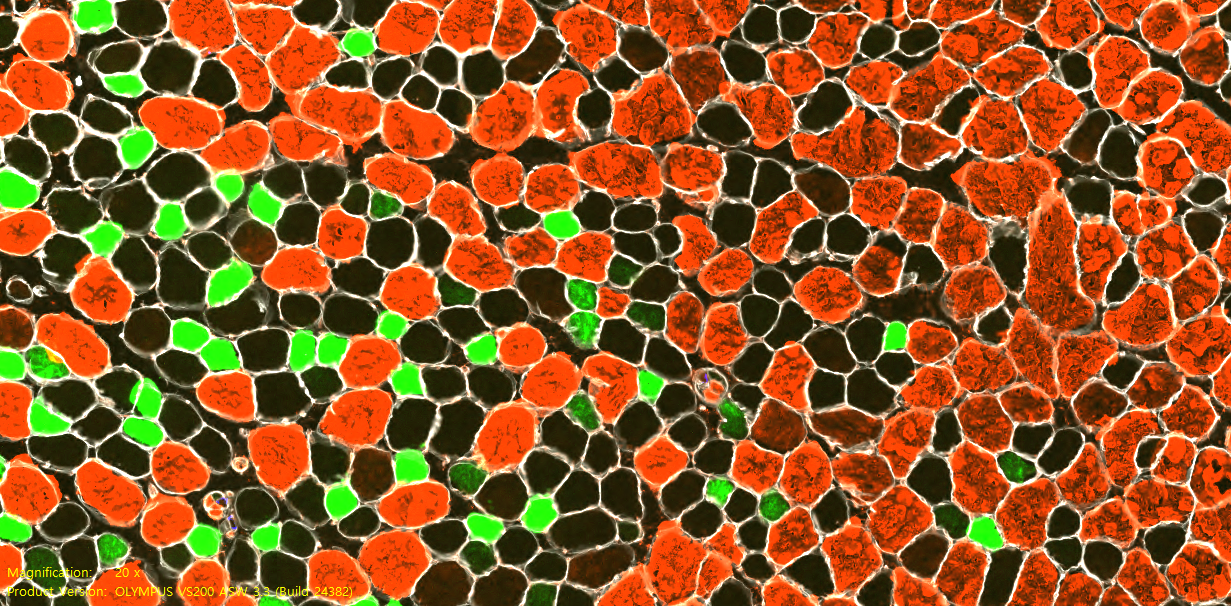

Supplement: Supplementary file 9 — Source data Fig. 7 [file 44321_2025_234_MOESM9_ESM.zip › Figure 7O/Aged+ BML260/BML-260 2.tif]

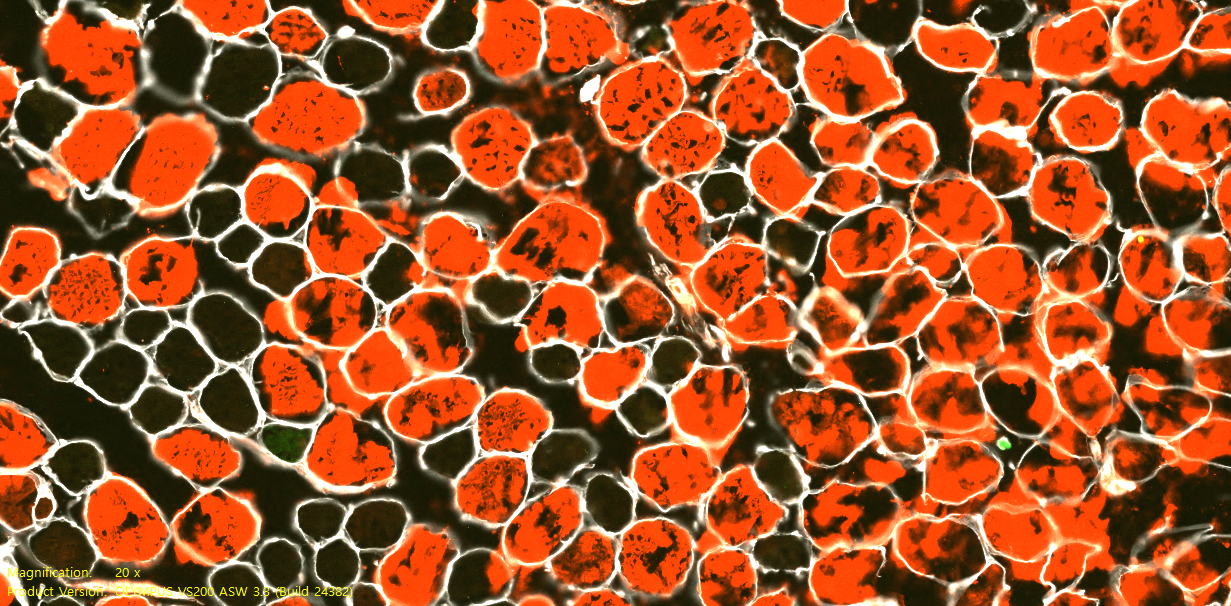

Supplement: Supplementary file 9 — Source data Fig. 7 [file 44321_2025_234_MOESM9_ESM.zip › Figure 7O/Aged+ BML260/BML-260 3.tif]

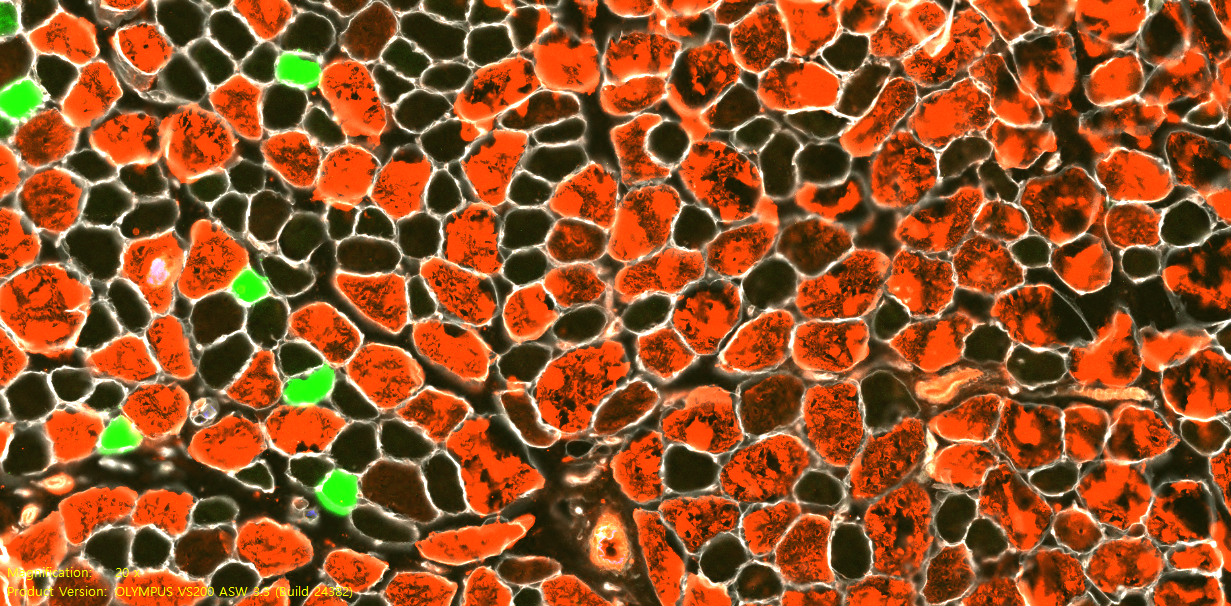

Supplement: Supplementary file 9 — Source data Fig. 7 [file 44321_2025_234_MOESM9_ESM.zip › Figure 7O/Aged+ BML260/BML-260 4.tif]

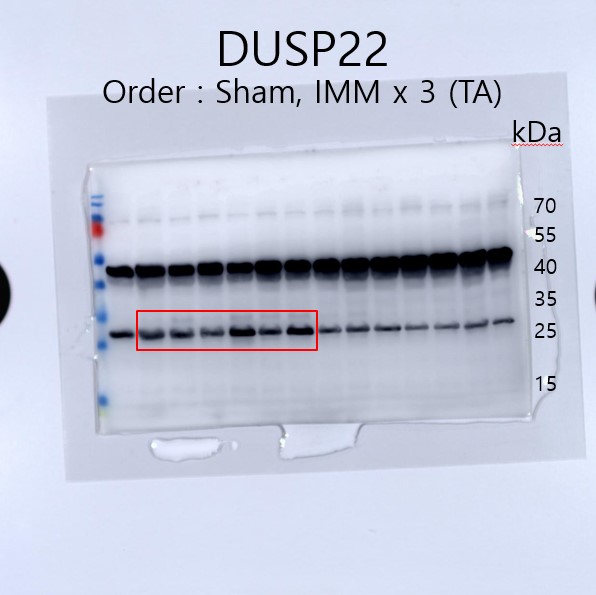

Supplement: Supplementary file 11 — Source data Fig. 9 [file 44321_2025_234_MOESM11_ESM.zip › Figure 9D/Figure 9D DUSP22.jpg]

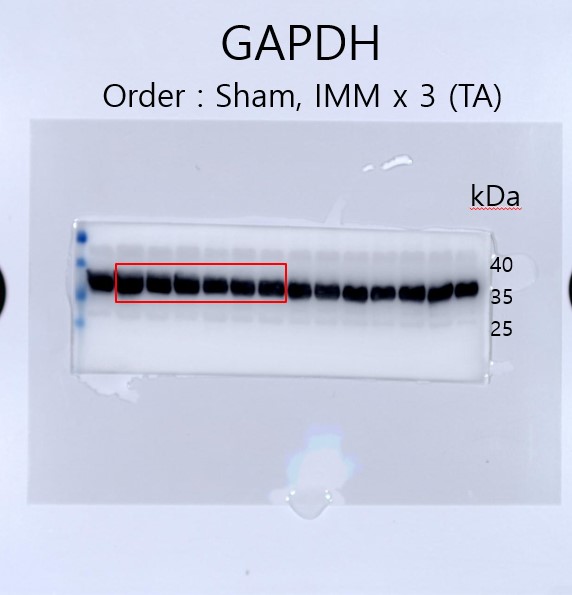

Supplement: Supplementary file 11 — Source data Fig. 9 [file 44321_2025_234_MOESM11_ESM.zip › Figure 9D/Figure 9D GAPDH.jpg]

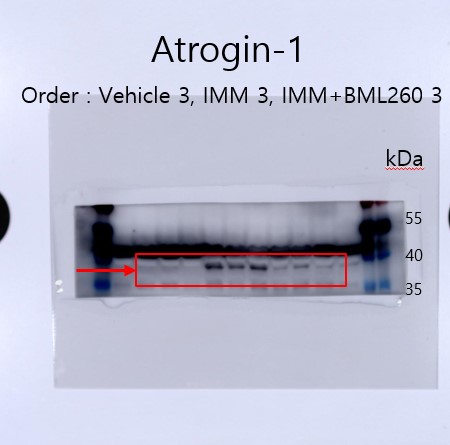

Supplement: Supplementary file 11 — Source data Fig. 9 [file 44321_2025_234_MOESM11_ESM.zip › Figure 9F/Figure 9F Atrogin-1.jpg]

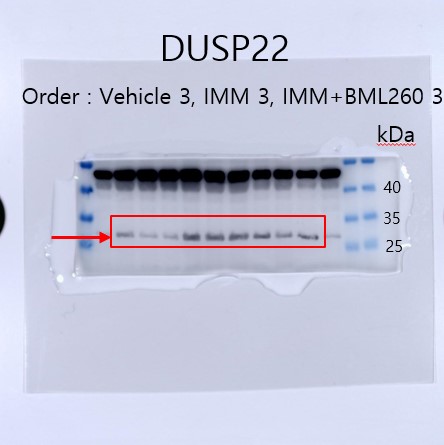

Supplement: Supplementary file 11 — Source data Fig. 9 [file 44321_2025_234_MOESM11_ESM.zip › Figure 9F/Figure 9F DUSP22.jpg]

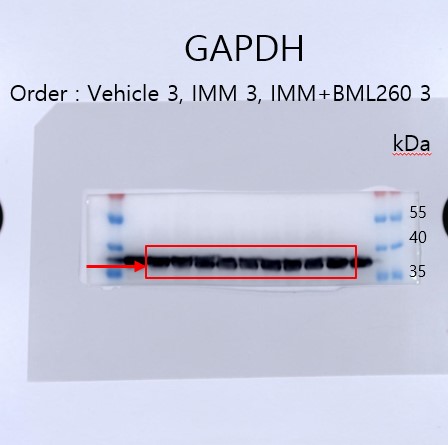

Supplement: Supplementary file 11 — Source data Fig. 9 [file 44321_2025_234_MOESM11_ESM.zip › Figure 9F/Figure 9F GAPDH.jpg]

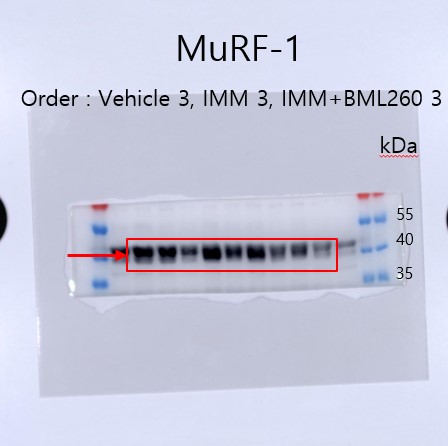

Supplement: Supplementary file 11 — Source data Fig. 9 [file 44321_2025_234_MOESM11_ESM.zip › Figure 9F/Figure 9F MuRF-1.jpg]

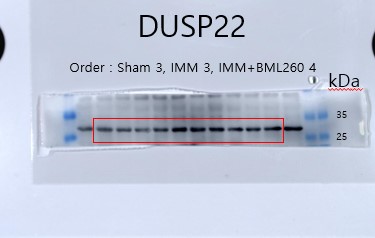

Supplement: Supplementary file 12 — EV Figure Source Data [file 44321_2025_234_MOESM12_ESM.zip › EV5/EV5/EV5/Figure EV5 DUSP22.jpg]

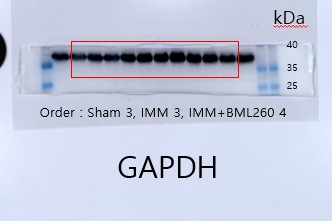

Supplement: Supplementary file 12 — EV Figure Source Data [file 44321_2025_234_MOESM12_ESM.zip › EV5/EV5/EV5/Figure EV5 GAPDH.jpg]

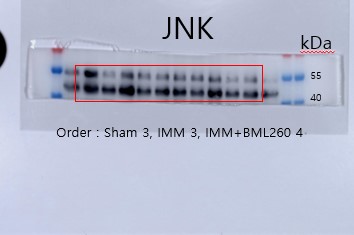

Supplement: Supplementary file 12 — EV Figure Source Data [file 44321_2025_234_MOESM12_ESM.zip › EV5/EV5/EV5/Figure EV5 JNK.jpg]

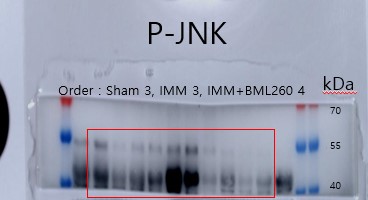

Supplement: Supplementary file 12 — EV Figure Source Data [file 44321_2025_234_MOESM12_ESM.zip › EV5/EV5/EV5/Figure EV5 p-JNK.jpg]

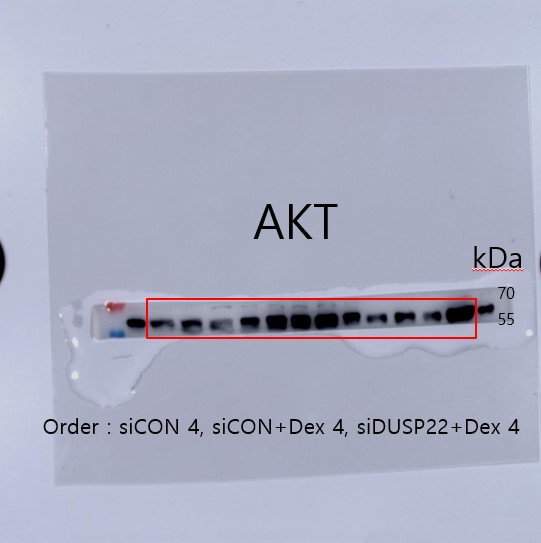

Supplement: Supplementary file 12 — EV Figure Source Data [file 44321_2025_234_MOESM12_ESM.zip › EV1/EV1/FIgure EV1/Figure S7 AKT.jpg]

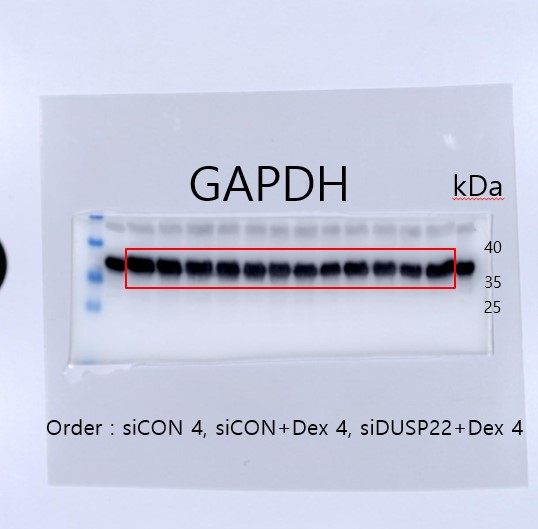

Supplement: Supplementary file 12 — EV Figure Source Data [file 44321_2025_234_MOESM12_ESM.zip › EV1/EV1/FIgure EV1/Figure S7 GAPDH.jpg]

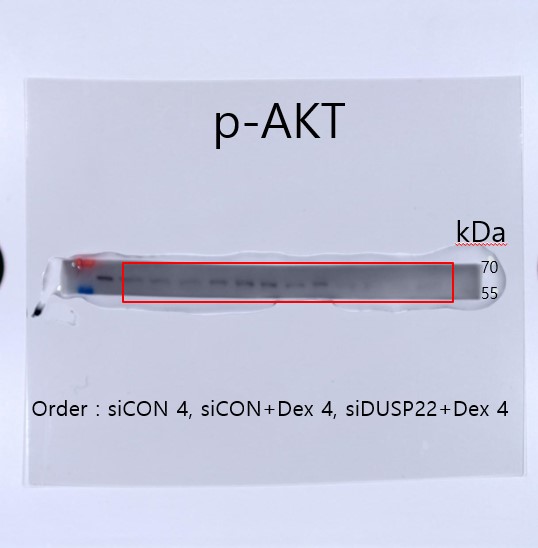

Supplement: Supplementary file 12 — EV Figure Source Data [file 44321_2025_234_MOESM12_ESM.zip › EV1/EV1/FIgure EV1/Figure S7 p-AKT.jpg]

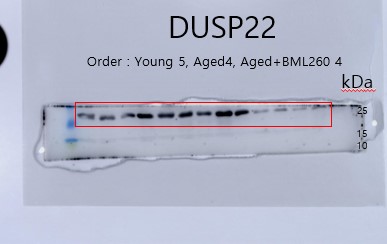

Supplement: Supplementary file 12 — EV Figure Source Data [file 44321_2025_234_MOESM12_ESM.zip › EV4/EV4/EV4/EV4A/Figure EV4 DUSP22 aged.jpg]

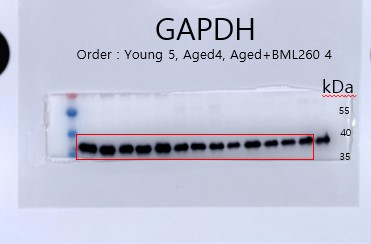

Supplement: Supplementary file 12 — EV Figure Source Data [file 44321_2025_234_MOESM12_ESM.zip › EV4/EV4/EV4/EV4A/Figure EV4 GAPDH aged.jpg]

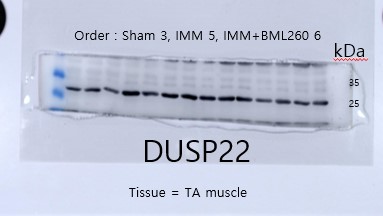

Supplement: Supplementary file 12 — EV Figure Source Data [file 44321_2025_234_MOESM12_ESM.zip › EV4/EV4/EV4/EV4B/Figure EV4 DUSP22 TA.jpg]

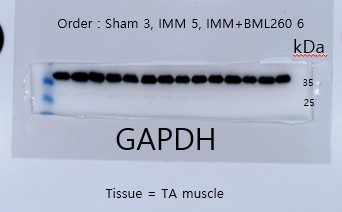

Supplement: Supplementary file 12 — EV Figure Source Data [file 44321_2025_234_MOESM12_ESM.zip › EV4/EV4/EV4/EV4B/Figure EV4 GAPDH TA.jpg]

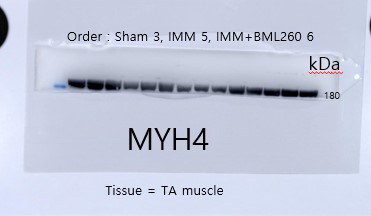

Supplement: Supplementary file 12 — EV Figure Source Data [file 44321_2025_234_MOESM12_ESM.zip › EV4/EV4/EV4/EV4B/Figure EV4 MYH4 TA.jpg]

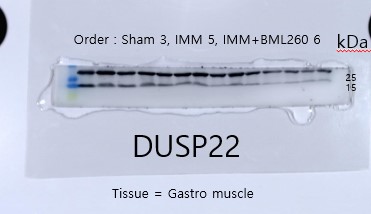

Supplement: Supplementary file 12 — EV Figure Source Data [file 44321_2025_234_MOESM12_ESM.zip › EV4/EV4/EV4/EV4C/Figure EV4 DUSP22 GA.jpg]

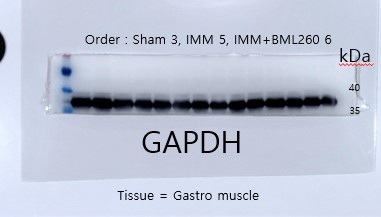

Supplement: Supplementary file 12 — EV Figure Source Data [file 44321_2025_234_MOESM12_ESM.zip › EV4/EV4/EV4/EV4C/Figure EV4 GAPDH GA.jpg]

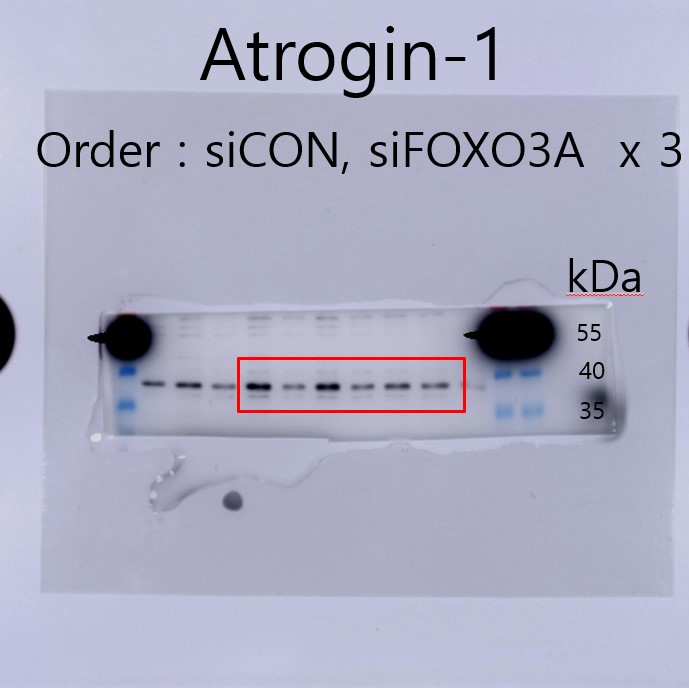

Supplement: Supplementary file 13 — Appendix Figure Source Data S2-S6 [file 44321_2025_234_MOESM13_ESM.zip › S3/Figure S3/FIgure S3 Atrogin-1.jpg]

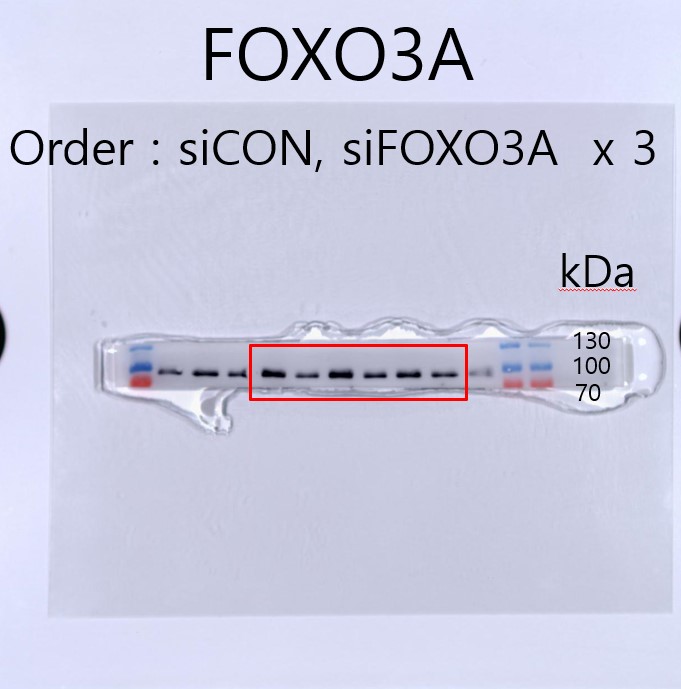

Supplement: Supplementary file 13 — Appendix Figure Source Data S2-S6 [file 44321_2025_234_MOESM13_ESM.zip › S3/Figure S3/Figure S3 FOXO3a.jpg]

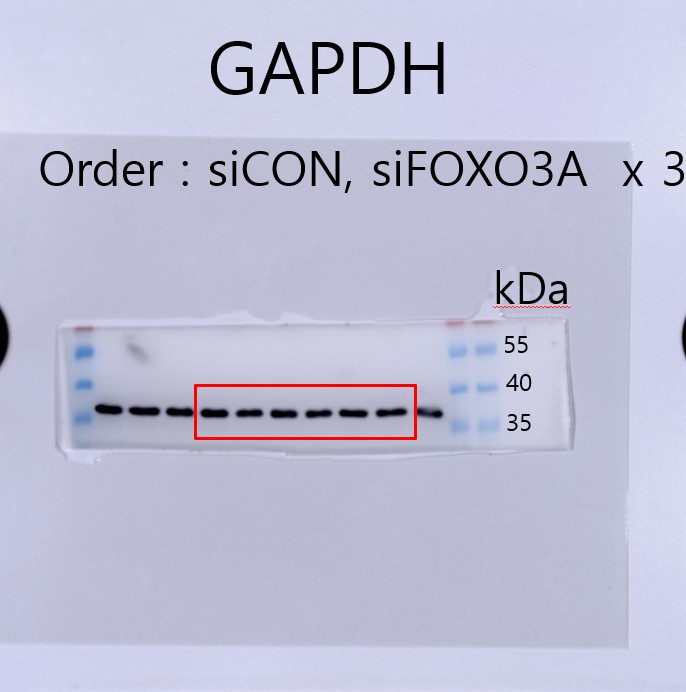

Supplement: Supplementary file 13 — Appendix Figure Source Data S2-S6 [file 44321_2025_234_MOESM13_ESM.zip › S3/Figure S3/Figure S3 GAPDH.jpg]

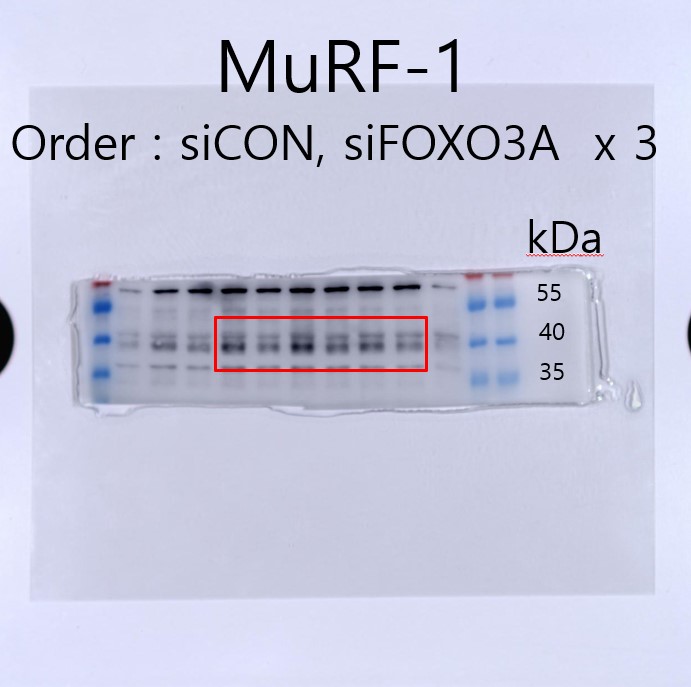

Supplement: Supplementary file 13 — Appendix Figure Source Data S2-S6 [file 44321_2025_234_MOESM13_ESM.zip › S3/Figure S3/Figure S3 MuRF-1.jpg]
