# Supplementary figures and images for: Modulating phosphatase DUSP22 with BML-260 ameliorates skeletal muscle wasting via Akt independent JNK-FOXO3a repression (part 3 of 3)
Source: EMBO Mol Med. 2025 Apr 22;17(6):1259–88. doi: 10.1038/s44321-025-00234-2 (PMC12162873; doi:10.1038/s44321-025-00234-2)

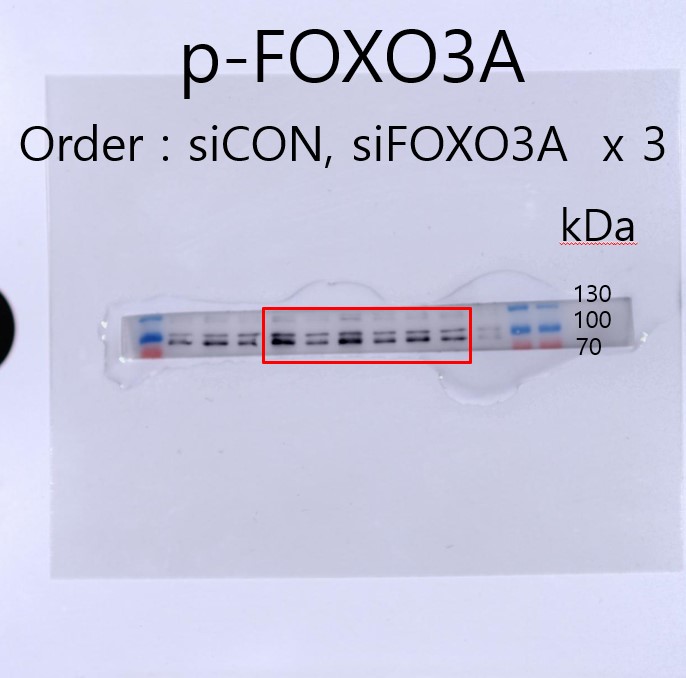

Supplement: Supplementary file 13 — Appendix Figure Source Data S2-S6 [file 44321_2025_234_MOESM13_ESM.zip › S3/Figure S3/Figure S3 p-FOXO3a.jpg]

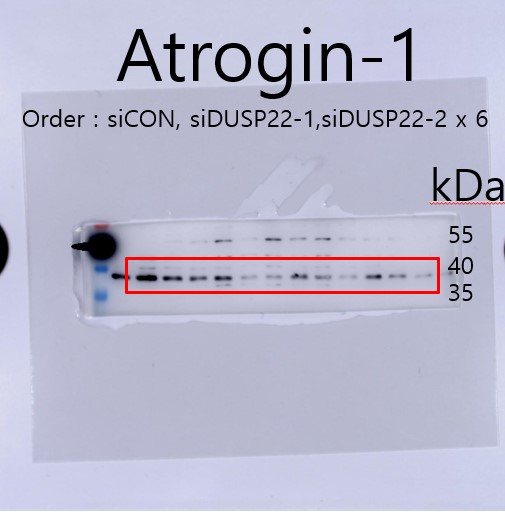

Supplement: Supplementary file 13 — Appendix Figure Source Data S2-S6 [file 44321_2025_234_MOESM13_ESM.zip › S4/Figure S4/Figure S4 Atrogin-1 1.jpg]

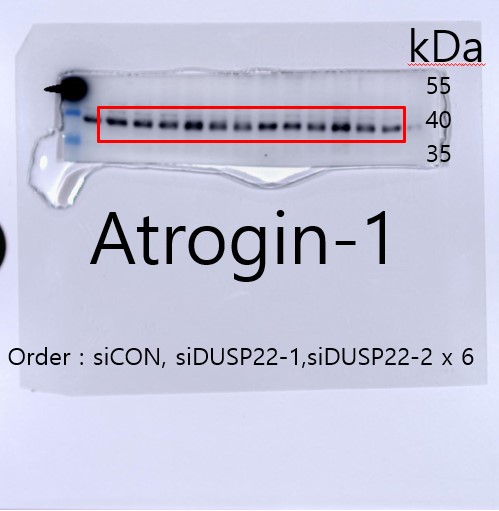

Supplement: Supplementary file 13 — Appendix Figure Source Data S2-S6 [file 44321_2025_234_MOESM13_ESM.zip › S4/Figure S4/Figure S4 Atrogin-1 2.jpg]

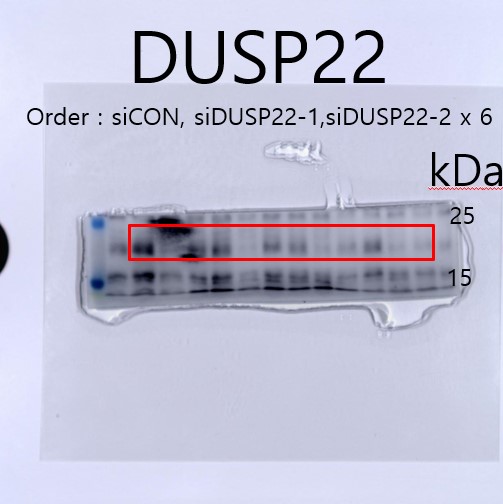

Supplement: Supplementary file 13 — Appendix Figure Source Data S2-S6 [file 44321_2025_234_MOESM13_ESM.zip › S4/Figure S4/Figure S4 DUSP22 1.jpg]

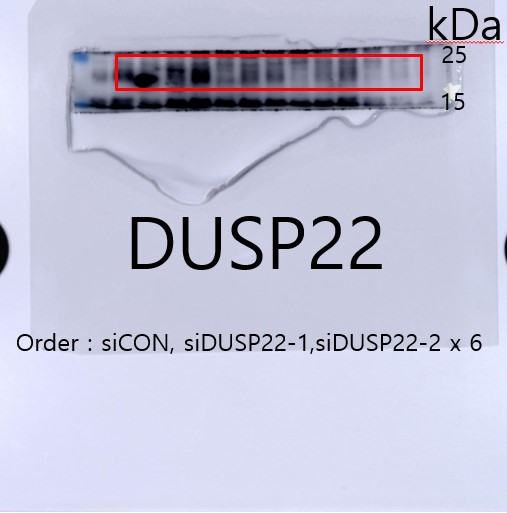

Supplement: Supplementary file 13 — Appendix Figure Source Data S2-S6 [file 44321_2025_234_MOESM13_ESM.zip › S4/Figure S4/Figure S4 DUSP22 2.jpg]

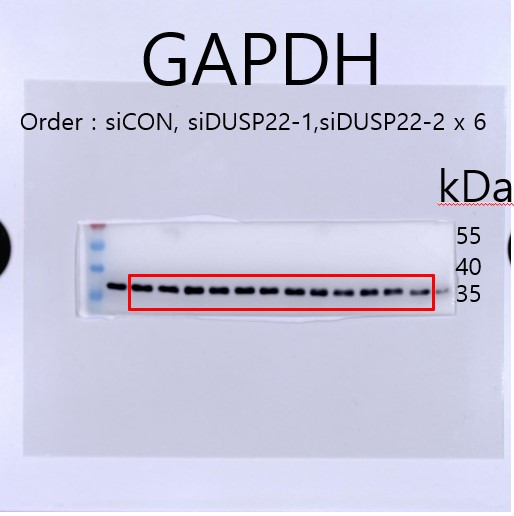

Supplement: Supplementary file 13 — Appendix Figure Source Data S2-S6 [file 44321_2025_234_MOESM13_ESM.zip › S4/Figure S4/Figure S4 GAPDH 1.jpg]

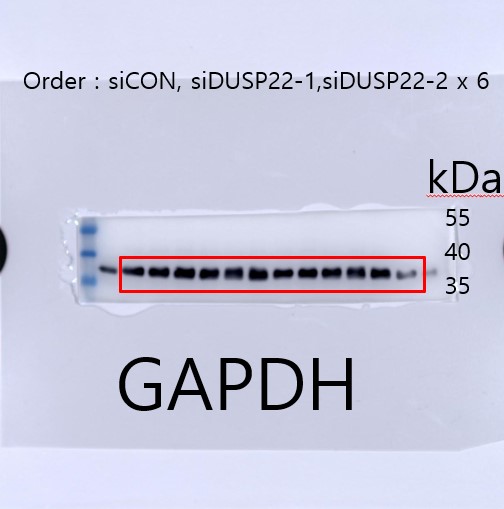

Supplement: Supplementary file 13 — Appendix Figure Source Data S2-S6 [file 44321_2025_234_MOESM13_ESM.zip › S4/Figure S4/Figure S4 GAPDH 2.jpg]

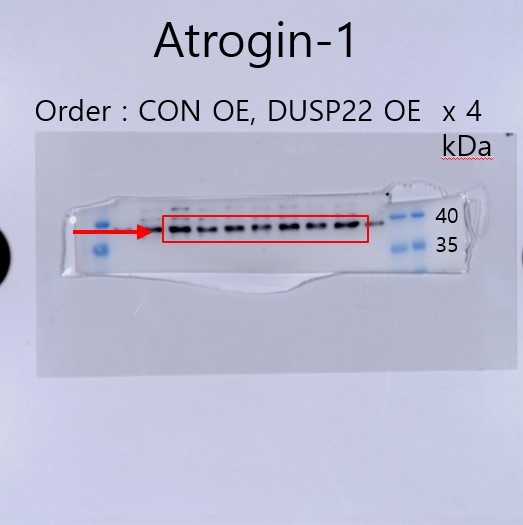

Supplement: Supplementary file 13 — Appendix Figure Source Data S2-S6 [file 44321_2025_234_MOESM13_ESM.zip › S2/Figure S2/Figure S2 Atrogin-1.jpg]

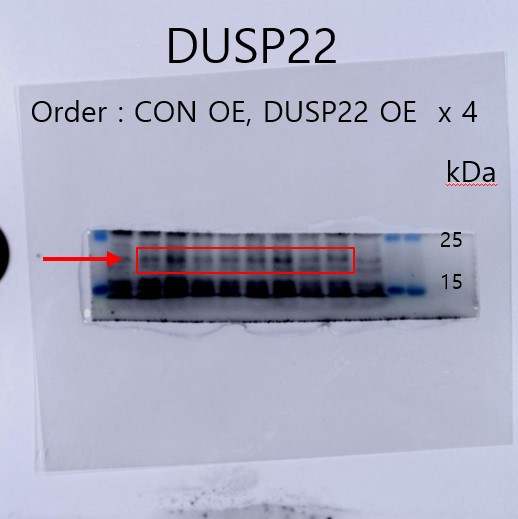

Supplement: Supplementary file 13 — Appendix Figure Source Data S2-S6 [file 44321_2025_234_MOESM13_ESM.zip › S2/Figure S2/Figure S2 DUSP22.jpg]

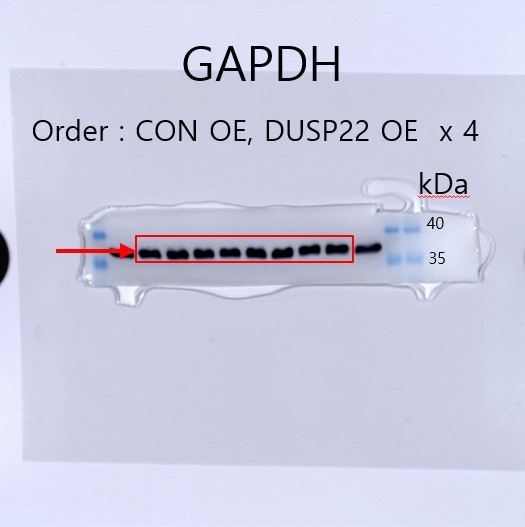

Supplement: Supplementary file 13 — Appendix Figure Source Data S2-S6 [file 44321_2025_234_MOESM13_ESM.zip › S2/Figure S2/Figure S2 GAPDH.jpg]

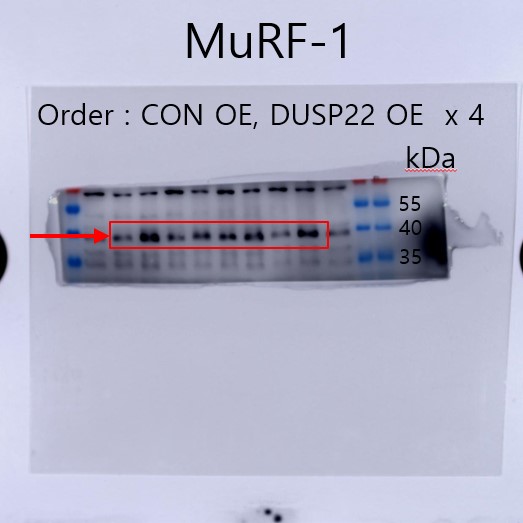

Supplement: Supplementary file 13 — Appendix Figure Source Data S2-S6 [file 44321_2025_234_MOESM13_ESM.zip › S2/Figure S2/Figure S2 MuRF-1.jpg]

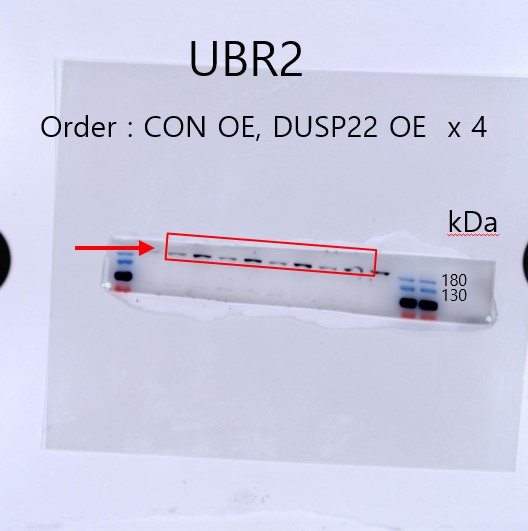

Supplement: Supplementary file 13 — Appendix Figure Source Data S2-S6 [file 44321_2025_234_MOESM13_ESM.zip › S2/Figure S2/Figure S2 UBR2.jpg]
