# Supplementary material for: Modulating phosphatase DUSP22 with BML-260 ameliorates skeletal muscle wasting via Akt independent JNK-FOXO3a repression
Source: EMBO Mol Med. 2025 Apr 22;17(6):1259–88. doi: 10.1038/s44321-025-00234-2 (PMC12162873; doi:10.1038/s44321-025-00234-2)
Supplement: Supplementary file 15 — Expanded View Figures [file 44321_2025_234_MOESM15_ESM.pdf]

Expanded View Figures

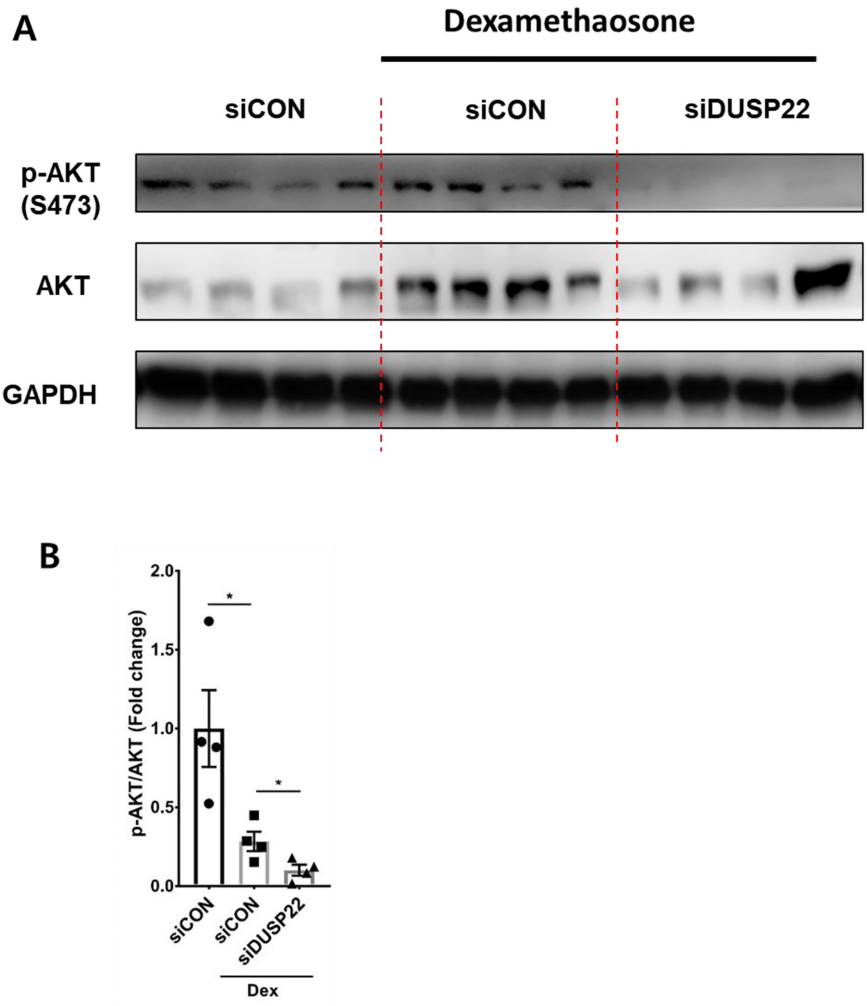

Figure EV1. DUSP22 siRNA reduces the phosphorylation of AKT.

(**A**, **B**) Western blot and densitometry analysis of AKT phosphorylation in the Dex-treated tibialis anterior (TA) muscle 3 d after delivery of control or DUSP22 siRNA ($n = 4$) ($p =$ (siCON = 0.029, siDUSP22+Dex = 0.0412)). GAPDH was used for normalization of expression. $^*p < 0.05$ indicate significantly increased or decreased. n represents biological replicates analyzed by Student's t test. Error bars represent the standard error of the mean (SEM). Source data are available online for this figure.

© The Author(s)

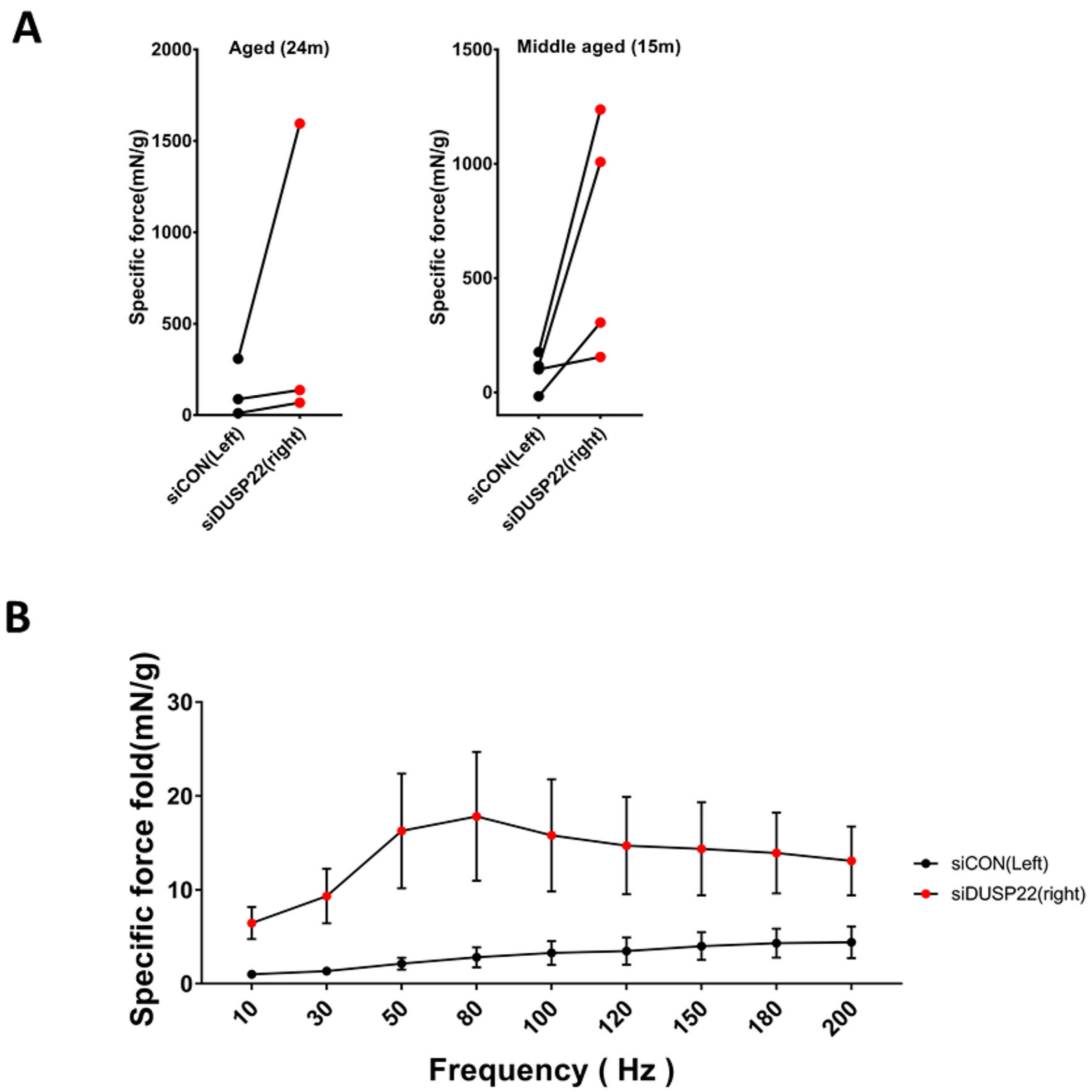

Figure EV2. DUSP22 siRNA enhances tetanic and twitch muscle force in the tibialis anterior muscle.

(**A**) Tetanic muscle contraction measurement in the TA muscle of aged ($n = 3$) ($p = 0.3974$) or middle aged mice ($n = 4$) ($p = 0.0807$) 3 d after the delivery of control or DUSP22 siRNA. (**B**) Twitch force measure in 15-month-old mice 3 d after the delivery of control or DUSP22 siRNA ($n = 4$) ($p = 0.1054$, 0.1677, 0.1964, 0.1858, 0.2118, 0.234, 0.2489, 0.2684, 0.2947). *n* represents biological replicates analyzed by Student's t test. Error bars represent the standard error of the mean (SEM). Source data are available online for this figure.

BML-260 detection : Plasma

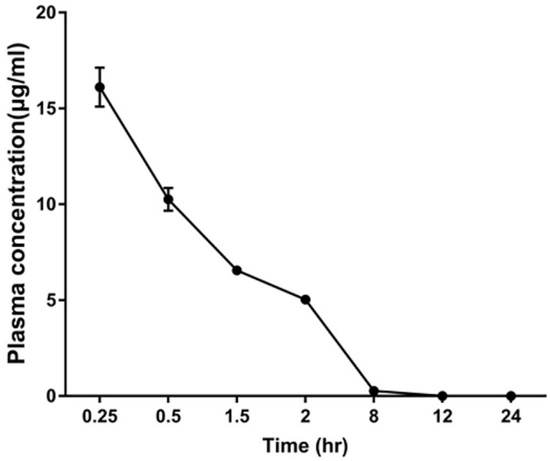

PK Parameters	Unit	BML-260
		IP
Dose	mg/kg	5.00
AUClast	h*ug/mL	26.14
AUCinf	h*ug/mL	26.70
Cmax	ug/mL	16.11
Tmax	h	0.25
Vz/F	L/kg	0.38
Cl/F	L/h/kg	0.19
$t_{1/2}$	h	1.42

BML-260 detection : Skeletal muscle (tibialis anterior)

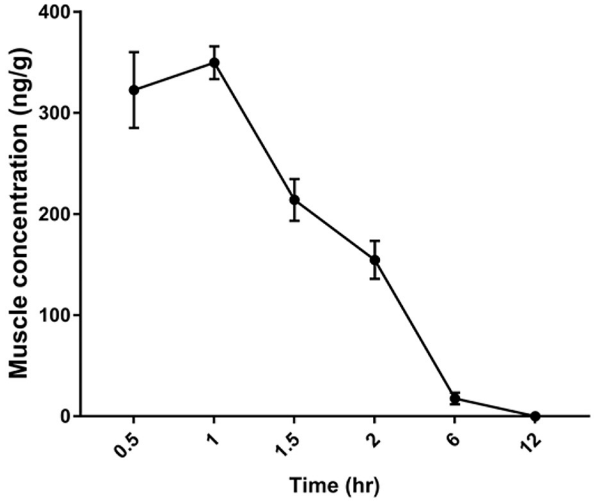

PK Parameters	Unit	BML-260
		IP
Dose	mg/kg	5.00
AUClast	h*ug/g	0.73
AUCinf	h*ug/g	0.76
Cmax	ug/g	0.35
Tmax	h	1.00
Vz/F	g/kg	11871.11
Cl/F	g/h/kg	6569.42
$t_{1/2}$	h	1.25

Figure EV3. Pharmacokinetic analysis of BML-260 in 6-week-old male C57BL/6J mice.

Pharmacokinetic analysis of BML-260 in 6-week-old male C57BL/6J mice (5 mg/kg delivered via IP injection) ($n = 3$). BML-260 detection was assessed in the plasma and tibialis anterior muscle. AUClast: Area under the concentration-time curve from time of dosing to the last measurable concentration. AUCinf: Area under the concentration-time curve extrapolated to infinity based on the last measurable concentration. Cmax: Maximum plasma concentration. Tmax: Time to reach the maximum plasma concentration. VZ/F: Apparent volume of distribution during the terminal phase. Cl/F: Apparent total body clearance after extravascular administration. t1/2: Terminal half-life. *n* represents biological replicates. Error bars represent the standard error of the mean (SEM). Source data are available online for this figure.

© The Author(s)

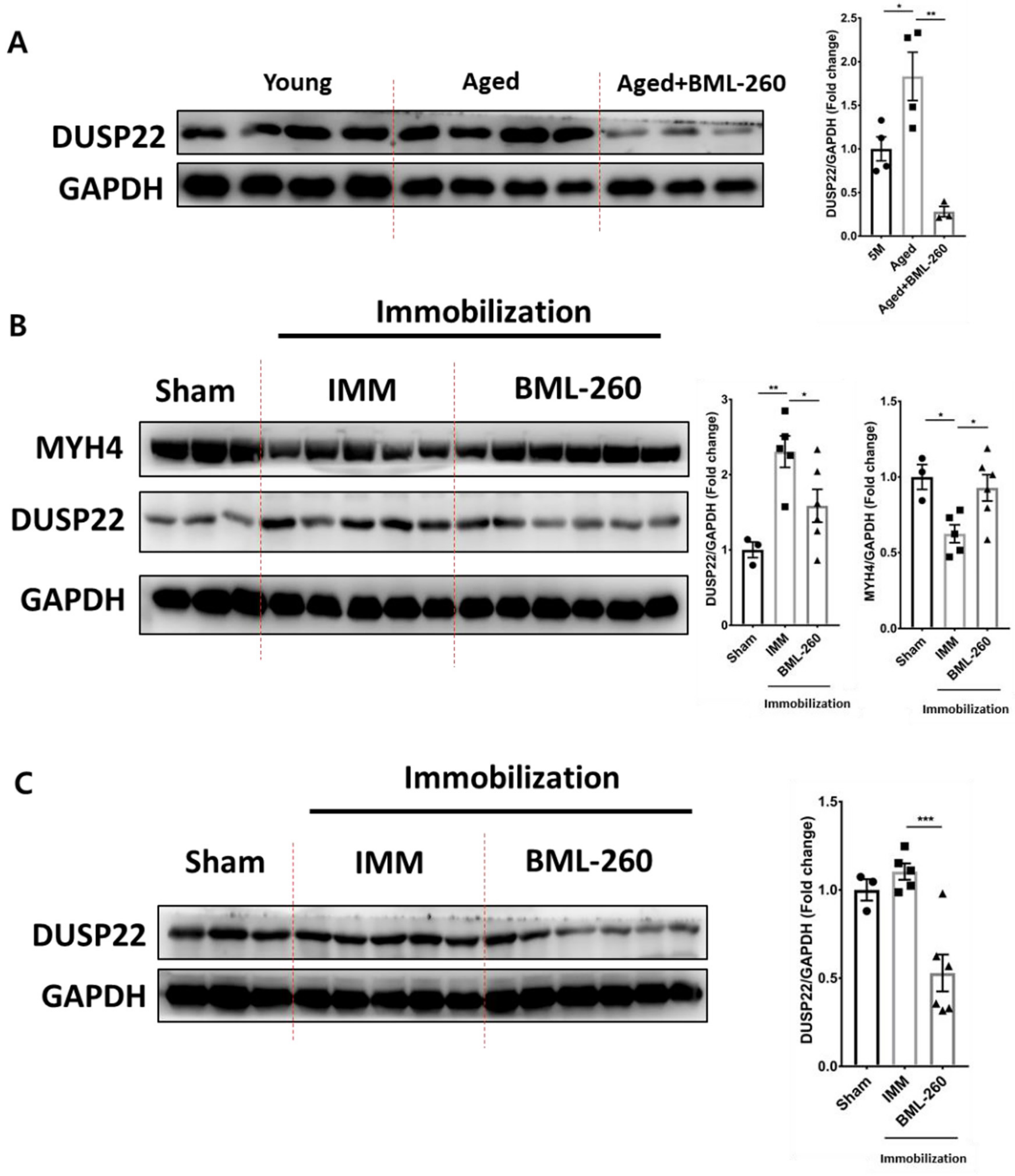

Figure EV4. DUSP22 and MYH4 levels in skeletal muscles of aged or immobilized mice treated with BML-260.

(**A**) Western blot analysis of DUSP22 expression in the TA of aged mice treated with BML-260 ($n = 4,3$) ($p =$ (Young = 0.0362, Aged+BML-260 = 0.0054)). GAPDH was used for normalization of expression. *$p < 0.05$ and **$p < 0.01$ indicate significantly increased or decreased. (**B**, **C**) Western blot analysis of DUSP22 expression in the TA ($n = 3,5,6$) ($p =$ (Sham = 0.0051, BML-260 = 0.0488)) (**B**) and gastrocnemius muscle ($n = 3,5,6$) ($p =$ (Sham=0.6845, BML-260 = 0.0008)) (**C**) of immobilized (IMM) mice treated with BML-260. For the TA muscle, MYH4 (myosin heavy chain 2B) ($p =$ (Sham=0.0253, BML-260 = 0.0288)) levels are also shown. GAPDH was used for normalization of expression. *$p < 0.05$, **$p < 0.01$, and ***$p < 0.001$ indicate significantly increased or decreased. n represents biological replicates analyzed by Student's t test. Error bars represent the standard error of the mean (SEM). Source data are available online for this figure.

© The Author(s)

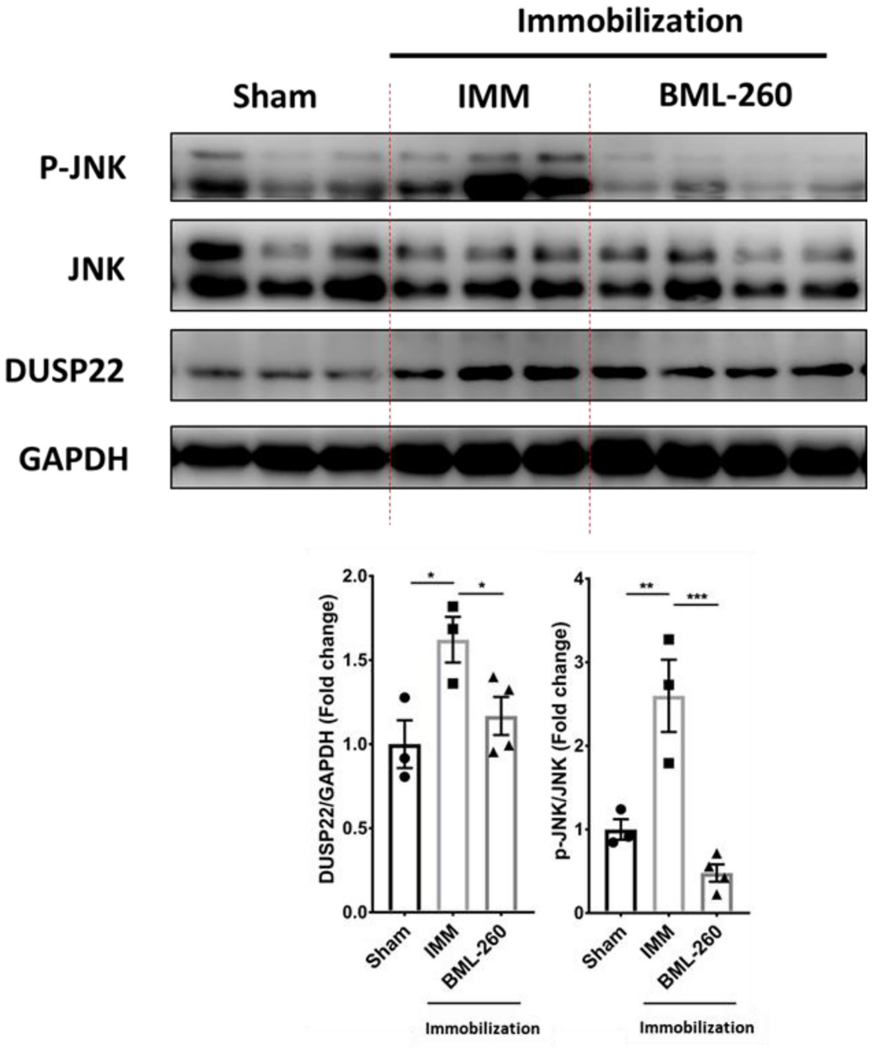

Figure EV5. DUSP22, JNK and phosphorylated JNK levels in the tibialis anterior of immobilized mice treated with BML-260.

Western blot analysis of DUSP22 (p = (Sham = 0.0341, BML-260 = 0.0494)), JNK and phosphorylated JNK (JNK-P) (p = (Sham = 0.0052, BML-260 = 0.0007)) levels in the TA muscle of immobilized mice (n = 3,4). GAPDH was used for normalization of expression. $^{*}p < 0.05$, $^{**}p < 0.01$, and $^{***}p < 0.001$ indicate significantly increased or decreased. n represents biological replicates analyzed by Student's t test. Error bars represent the standard error of the mean (SEM). Source data are available online for this figure.

 © The Author(s)
